# *In silico* analyses of mitochondrial ORFans in freshwater mussels (Bivalvia: Unionoida) provide a framework for future studies of their origin and function

**DOI:** 10.1186/s12864-016-2986-6

**Published:** 2016-08-09

**Authors:** Alyssa Mitchell, Davide Guerra, Donald Stewart, Sophie Breton

**Affiliations:** 1 Department of Biological Sciences, Université de Montréal, CP 6128, Succursale Centre-Ville, Montréal, QC H3C 3J7 Canada; 2 Department of Biology, Acadia University, Wolfville, NS B4P 2R6 Canada

**Keywords:** Mitochondrial DNA, Mitochondrial ORFans, Mitochondrial inheritance, Doubly uniparental inheritance of mitochondria, Bivalvia, Unionoida

## Abstract

**Background:**

Many species of bivalves exhibit a unique system of mtDNA transmission named Doubly Uniparental Inheritance (DUI). Under this system, species have two distinct, sex-linked mitochondrial genomes: the M-type mtDNA, which is transmitted by males to male offspring and found in spermatozoa, and the F-type mtDNA, which is transmitted by females to all offspring, and found in all tissues of females and in somatic tissues of males. Bivalves with DUI also have sex-specific mitochondrial ORFan genes, (M-*orf* in the M mtDNA, F-*orf* in the F mtDNA), which are open reading frames having no detectable homology and no known function. DUI ORFan proteins have previously been characterized *in silico* in a taxonomically broad array of bivalves including four mytiloid, one veneroid and one unionoid species. However, the large evolutionary distance among these taxa prevented a meaningful comparison of ORFan properties among these divergent lineages. The present *in silico* study focuses on a suite of more closely-related Unionoid freshwater mussel species to provide more reliably interpretable information on patterns of conservation and properties of DUI ORFans. Unionoid species typically have separate sexes, but hermaphroditism also occurs, and hermaphroditic species lack the M-type mtDNA and possess a highly mutated version of the F-*orf* in their maternally transmitted mtDNA (named H*-orf* in these taxa). In this study, H*-orf*s and their respective proteins are analysed for the first time.

**Results:**

Despite a rapid rate of evolution, strong structural and functional similarities were found for M-*ORF* proteins compared among species, and among the F*-ORF* and H*-ORF* proteins across the studied species. *In silico* analyses suggest that M-*ORF*s have a role in transport and cellular processes such as signalling, cell cycle and division, and cytoskeleton organisation, and that F-*ORF*s may be involved in cellular traffic and transport, and in immune response. H-*ORF*s appear to be structural glycoproteins, which may be involved in signalling, transport and transcription. Our results also support either a viral or a mitochondrial origin for the ORFans.

**Conclusions:**

Our findings reveal striking structural and functional similarities among proteins encoded by mitochondrial ORFans in freshwater mussels, and strongly support a role for these genes in the DUI mechanism. Our analyses also support the possibility of DUI systems with elements of different sources/origins and different mechanisms of action in the distantly-related DUI taxa. Parallel situations to the novel mitochondrially-encoded functions of freshwater mussel ORFans present in some other eukaryotes are also discussed.

**Electronic supplementary material:**

The online version of this article (doi:10.1186/s12864-016-2986-6) contains supplementary material, which is available to authorized users.

## Background

Metazoan mitochondrial genomes (mtDNAs) are typically small, circular genomes without introns that encode two ribosomal RNAs, 22 transfer RNAs, and 13 proteins involved in ATP production [1, 2]. Strict maternal inheritance (SMI) of mtDNA is predominant among animals with limited or no paternal contribution [3]. There are, however, many exceptions to these characteristics (e.g. [4–6]). Anomalous gene contents have been found among metazoan mtDNAs, particularly in invertebrates (reviewed in [6]). For example, duplications of typical protein-coding genes have been reported in several mollusc species, including cephalopods, aplacophorans, and bivalves. Additional ‘atypical’ protein-coding genes with non-OXPHOS functions have been reported in cnidarians, sponges, and placozoans (e.g. *dnaB*, *tatC*); and mitochondrial ORFans, i.e. genes with unknown function, have been identified in cnidarians, and also in bivalves with doubly uniparental inheritance of mtDNA (DUI), which is the only known exception to SMI in animals [6].

DUI has been reported in marine and freshwater bivalves, specifically the orders Mytiloida, Nuculanoida, Unionoida, and Veneroida [7–10]. Species with DUI possess mitochondrial genomes that are transmitted in a sex-specific manner (known as a female-transmitted F-type and a male-transmitted M-type mtDNA, respectively). Haploid eggs typically contain mitochondria with only F-type mtDNA (but see [11, 12]), while sperm mitochondria, which enter the egg when fertilization occurs, only contain the M-type [10]. If the embryo develops as a female, sperm mitochondria are dispersed and/or destroyed, leading to homoplasmic females (similar to what happens under SMI) [10]. However, if the embryo develops as a male, sperm mitochondria remain grouped together, and are eventually sequestered in the germ line, which becomes homoplasmic for the M mtDNA [13, 14]. Males are therefore heteroplasmic individuals, with mitochondria inherited from their mother containing the F-type mtDNA present throughout their soma, and mitochondria inherited from their father containing the M-type mtDNA in their germ line cells (in males M mtDNA can also be found in variable proportions in somatic tissues [9, 10]). DNA divergence between conspecific M- vs. F-type mitochondrial genomes over 40 % has been found in many species [10].

**Fig. 1:**
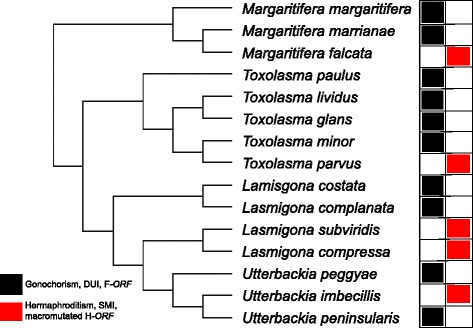
Simplified phylogeny of some gonochoric and hermaphroditic unionoid bivalves redrawn after Breton et al. [16]. The presence of DUI (Doubly Uniparental Inheritance) with F- and M-type mtDNAs in gonochoric species is indicated in black, whereas hermaphroditism with SMI (Strict Maternal Inheritance) is indicated in red. Species in black have F*-ORF*s containing one predicted transmembrane (TM) helix in their N-terminal portion, whereas hermaphroditic species have macromutated H*-ORF*s with repeat units and sometimes more than one predicted TM helix [16]

The mitochondrial genomes of bivalve species with DUI also contain additional, sex-specific protein-coding genes known as mitochondrial ORFans - F*-orf*s and M*-orf*s in the F- and M-type mtDNAs, respectively - whose products are exported from the organelle and may be involved in functions other than energy production [15–20]. For example, in freshwater mussels, species typically have separate sexes (gonochorism or dioecy), but hermaphroditic species also occur rarely [21, 22]. In gonochoric species, an absolute correlation has been observed between the presence of DUI and novel sex-specific proteins encoded by the F- and M-type mtDNAs (F-*ORF* and M-*ORF*), whereas hermaphroditic species lack the M-type altogether [16]. Hermaphroditic species appear to follow the SMI rule of mitochondrial transmission and individual mussels have only one type of mtDNA, called H-type [16]. The H-type is remarkably similar to (and evolutionarily derived from) the F-type mtDNA of closely-related gonochoric species except for the novel ORFan gene (named H*-orf* in these species), which is a highly mutated version of the F-*orf* in their sister taxa [16] (Fig. 1)*.* For these reasons, Breton et al. [16] proposed a connection between DUI and the maintenance of separate sexes in freshwater mussels. However, the link between DUI and sex determination, and the cause of deviation from the “SMI rule” in bivalves remain open questions.

The first in-depth bioinformatic analysis of the structures and potential functions of F-*ORF* and M*-ORF* proteins was performed by Milani et al. [18] on the following DUI bivalve species: the marine mussels *Musculista senhousia*, *Mytilus edulis*, *Mytilus galloprovincialis*, *Mytilus trossulus* and *Mytilus californianus* (Mytiloida), the marine clam *Ruditapes philippinarum* (Veneroida), and the freshwater mussel *Venustaconcha ellipsiformis* (Unionoida). M*-orf* and F*-orf* nucleotide sequences were found to be highly variable, with mostly non-synonymous mutations, indicating rapid evolution and supporting previous claims that these protein-coding genes are the fastest-evolving mitochondrial genes in bivalves with DUI [16–18]. Despite this fast rate of evolution, structural similarities in their translated amino acid sequences were observed among species and ORFan proteins were predicted to share similar functions. For example, F-*ORF*s were largely predicted to bind and interact with nucleic acids, associate with membranes for cell adhesion and/or signalling, or play a role in immune response. M-*ORF*s were also predicted to be membrane-associated and interact with nucleic acids, primarily for signalling, cell differentiation and development, and also for cytoskeleton formation and dynamics, ubiquitination, apoptosis, and immune response [18]. Even if hit probabilities in the proteins were sometimes low and the regions of similarity were of short lengths, several clues suggested that the respective novel ORFans originated from endogenization of viral DNA [18, 19]. However, obtaining satisfactory alignments including F-*ORF*s and M-*ORF*s from all species was impossible due to the highly divergent nature of these proteins [18]. This indicated either that their fast rate of evolution erased any evidence of ORFan sequence similarities (homology) among species or that the ORFans originated from independent virus endogenization events [18]. It is also conceivable that the ORFans originated from different sources/processes but evolved similar function(s) in these distantly related DUI species, particularly if DUI evolved independently more than once [18]. Other than a viral origin, there are at least three other possibilities for the source of these mitochondrial ORFans: they may have originated from (i) a duplicated and diverged mitochondrial gene, (ii) a gene composed from previously non-coding mitochondrial sequences, or (iii) a gene transferred from the nucleus to the mitochondrion (e.g. [17]).

Unfortunately, it is not currently possible to confirm whether or not the mitochondrial ORFans in phylogenetically unrelated DUI species are homologous because of their high divergence and our incomplete knowledge regarding their distribution in bivalves. One option to better understand the origin(s) and function(s) of a subset of these ORFans is to compare a suite of more closely related sequences within a single order of bivalves. Freshwater mussels (Unionoida) offer an excellent opportunity for this for at least two reasons: (1) they are an evolutionarily old group of bivalves, suggesting that their ORFans have an ancient origin and that DUI in this group might be one of the first examples of this phenomenon in bivalves [23], and (2) complete F and M genomes or F*-orf*, M*-orf* and H*-orf* sequences are available for several gonochoric species and five independently evolved hermaphroditic species (e.g. [16, 23, 24]). All of these taxa belong to the family Unionidae (except for *Margaritifera falcata* [Margaritiferidae]), but recently we have sequenced the F and M mtDNAs from *Cumberlandia monodonta* (Margaritiferidae) and *Hyridella menziesii* (Hyriidae) (these genomes have been sequenced at the sequencing platform of McGill University [Montreal, Canada] using the genome sequencer FLX sequencing service), and these genomes possess an F*-orf* and an M*-orf*, suggesting that these unique genes have been present and functioning continuously for >200 million years in this group ([16, 23]; Guerra et al. unpublished).

The present study aims to predict the origin, structure, and function of the F*-ORF* and M*-ORF* protein sequences in Unionoida, and to analyze the H*-ORF*s for the first time. Our results confirm that they are encoded by the fastest evolving genes in unionoid mitochondrial genomes, that they share structural and functional similarities, and that their respective ORFans could have a viral or a mitochondrial origin, leading us to revisit the evolutionary scenario of multiple origins of DUI [18, 19].

## Methods

### Sequences used in the analyses

ORFan, *cox1*, and *atp8* nucleotide sequences of unionoid bivalve species were either obtained from the National Center for Biotechnology Information (NCBI) or from newly sequenced mitochondrial genomes (i.e. *H. menziesii* and *C. monodonta*; Guerra et al. unpublished). All species and GenBank entries used in this study are listed in Table 1 (note that M*-orf* sequences for *Lasmigona complanata*, *Margaritifera margaritifera* and *Toxolasma lividus* have not been obtained; Additional file 1: Table S1). The sequences were translated with ORF Finder [25] using the invertebrate mitochondrial genetic code and analyzed at the nucleotide and/or amino acid level (see below). Because M*-ORF* and F*-ORF* protein sequences vary little within a species, only one sequence was used for each gonochoric species. H*-ORF* sequences are highly variable within species [16], and so multiple sequences were analyzed per species to provide a more complete picture of intraspecific H*-ORF* evolution and potential functionality.

**Table 1:** Sequences analyzed in the present study for gonochoric species with DUI and hermaphroditic species with SMI

Species	mtDNA type	Accession number	ORF names
Subfamiliy Ambleminae			
*Quadrula quadrula*	M	FJ809751.1	Qqu-M*orf*
	M	FJ809751.1	Qqu-M*cox1*
	M	FJ809751.1	Qqu-M*atp8*
	F	FJ809750.1	Qqu-F*orf*
	F	FJ809750.1	Qqu-F*cox1*
	F	FJ809750.1	Qqu-F*atp8*
*Toxolasma lividus*	F	HM849457.1	Tli-F*orf*
*Toxolasma parvum*	H	KU728097	Tpa-H*orf*
*Venustaconcha ellipsiformis*	M	FJ809752.1	Vel-M*orf*
	M	FJ809752.1	Vel-M*cox1*
	M	FJ809752.1	Vel-M*atp8*
	F	FJ809753.1	Vel-F*orf*
	F	FJ809753.1	Vel-F*cox1*
	F	FJ809753.1	Vel-F*atp8*
Subfamiliy Anodontinae			
*Anodonta anatina*	M	KF030962.1	Aan-M*orf*
	F	KF030964.1	Aan-F*orf*
Subfamiliy Gonideinae			
*Inversidens japanensis*	M	AB055624.1	Ija-M*orf*
	M	AB055624.1	Ija-M*cox1*
	M	AB055624.1	Ija-M*atp8*
	F	AB055625.1	Ija-F*orf*
	F	AB055625.1	Ija-F*cox1*
	F	AB055625.1	Ija-F*atp8*
*Solenaia carinatus*	M	KC848655.1	Sca-M*orf*
	M	KC848655.1	Sca-M*cox1*
	M	KC848655.1	Sca-M*atp8*
	F	KC848654.1	Sca-F*orf*
	F	KC848654.1	Sca-F*cox1*
	F	KC848654.1	Sca-F*atp8*
Subfamiliy Hyriidae			
*Hyridella menziesii*	M	KU728093	Hme-M*orf*
	M	KU728094	Hme-M*cox1*
	F	KU728092	Hme-F*orf*
	F	AY785394.1	Hme-F*cox1*
Subfamiliy Margaritiferinae			
*Cumberlandia monodonta*	M	KU728095	Cmo-M*orf*
	M	KU728096	Cmo-M*cox1*
	F	HM849375.1	Cmo-F*orf*
	F	KF647374.1	Cmo-F*cox1*
*Margaritifera falcata*	H	HM849545.1	Mfa-H*orf* (top- bottom 1–4)
	H	HM856634.1	
	H	HM849547.1	
	H	HM849548.1	
	H	HM856634.1	Mfa-H*cox1* (top-bottom 1–2)
	H	NC_015476.1	
*Margaritifera margaritifera*	F	HM849399.1	Mma-F*orf*
	F	HM849095.1	Mma-F*cox1*
Subfamiliy Unioninae			
*Lasmigona complanata*	F	HM849393.1	Lco-F*orf*
*Lasmigona compressa*	H	HM849534.1	Lco-H*orf* (top-bottom 1–2)
	H	HM849535.1	
	H	HM856638.1	Lco-H*cox1* (top-bottom 1–2)
	H	NC_015481.1	
*Lasmigona subviridis*	H	HM849542.1	Lsu-H*orf* (top-bottom 1–2)
	H	HM849543.1	
*Pyganodon grandis*	M	FJ809755.1	Pgr-M*orf*
	M	FJ809755.1	Pgr-M*cox1*
	M	FJ809755.1	Pgr-M*atp8*
	F	FJ809754.1	Pgr-F*orf*
	F	FJ809754.1	Pgr-F*cox1*
	F	FJ809754.1	Pgr-F*atp8*
*Utterbackia imbecillis*	H	HM849591.1	Uim-H*orf* (top-bottom 1–7)
	H	HM849595.1	
	H	HM849594.1	
	H	HM849601.1	
	H	HM849606.1	
	H	HM849597.1	
	H	HM849584.1	
	H	NC_015479	Uim-H*cox1* (top-bottom 1–2)
	H	HM856637.1	
*Utterbackia peninsularis*	M	HM856635.1	Upe-M*orf*
	M	HM856635.1	Upe-M*cox1*
	M	HM856635.1	Upe-M*atp8*
	F	HM856636.1	Upe-F*orf*
	F	HM856636.1	Upe-F*cox1*
	F	HM856636.1	Upe-F*atp8*

Note: *M* M mtDNA in a DUI gonochoric breeding system, *F* F mtDNA in a DUI gonochoric breeding system, *H* H mtDNA in a non-DUI hermaphroditic breeding system

### Analyses of ORFan sequences and protein secondary structures

Alignments of ORFan, *cox1*, and *atp8* nucleotide and translated protein sequences were performed with M-COFFEE (DNA) and PSI-COFFEE (proteins) [26]. Nucleotide and amino acid p-distances, as well as a codon-based test of positive selection using the Nei-Gojobori method [27], were calculated using MEGA6 [28] with variance estimated using 500 bootstrap repetitions. The program VISTA [29] was used to display the level of sequence conservation between M vs. M, F vs. F, and F vs. H complete mitochondrial genomes. M- and F-type mtDNAs were not compared due to their previous characterization that showed extreme intraspecific sequence divergences [16, 23]. Hydropathy profiles of each amino acid sequence were calculated with the ProtScale tool at ExPASy [30] using the method of Kyte and Doolittle [31]. Putative transmembrane (TM) helices were identified using a variety of protein signature recognition methods implemented by the following programs: Phobius [32], InterProScan (TMHMM) [33], TMPred [34], TOPCONS [35], and Predict Protein [36].

### Functional analyses of ORFan proteins

Evidence of signal peptides (SPs) was sought using Phobius [26], InterProScan [33], PrediSi [37], and SignalP [38]. Motif Scan [39] and HHpred [40] were used to search for known functional sequence motifs and domains. TPRpred [41] was used to search for potential tetratricopeptide repeat (TPR) or pentatricopeptide repeat (PPR) motifs. The following procedures were used to predict the function of ORFan proteins: (1) we performed BLASTp, tBLASTx, and PSI-BLAST searches against NCBI entire non-redundant protein database (NRDB) and against mitochondrial proteins only (last accessed July, 2015) with default parameters [42], as well as FASTA and PSI-BLAST searches against UniProt (release 2015_05) with default parameters, at the EBI websites [43] and [42], respectively; (2) we used hmmbuild (v3.1b2; downloaded from http://hmmer.janelia.org) [44] to generate two HMM profiles from both the F-*ORF* and M-*ORF* protein alignments (four profiles in total; see below) (H-*ORF*s were not considered given their scattered phylogenetic distribution and independent evolutionary histories) using default and custom parameters (for the latter procedure, the options --fast --symfrac 0 --fragthresh 0 --wnone --enone were used), and performed profile HMM – sequence comparison against UniProtKB, Swissprot, PDB, QfO, and Pfamseq databases using HMMER hmmsearch [44] with default parameters (E-value cutoff = 0.001); (3) for profile HMM – profile HMM comparisons, we used HHpred, which compares HMM profiles with databases of HMMs representing proteins with known structure (e.g. PDB, SCOP) or annotated protein families (e.g. PFAM, SMART, CDD, COGs, KOGs); and (4) the following programs were also used to predict the function of ORFan proteins: @tome2, which predicts tertiary structure and searches for similarity to proteins with structures solved [45]; I-TASSER, which uses a hierarchical protein structure modeling approach that is based on the secondary-structure enhanced profile–profile threading alignment [46]; and PredictProtein, which predicts aspects of protein structure (secondary structure, solvent accessibility, transmembrane helices [TMSEG] and strands, coiled-coil regions, disulfide bonds and disordered regions) and function (identification of functional regions, homology-based inference of Gene Ontology terms, comprehensive subcellular localization prediction, protein-protein binding sites, protein-polynucleotide binding sites and predictions of the effect of point mutations [non-synonymous SNPs] on protein function) [36]. For BLASTp and PredictProtein all matches with E-values <1.0 were kept, while for position-specific iterative or PSI-BLAST all matches with E-values <0.01 were kept as recommended by the program (except for PSI-BLAST analyses against NCBI mitochondrial genes only, where E-values <1.0 were kept, see below). For I-TASSER, all top templates and structural analogs were recorded. All @tome2 results were kept. Motif Scan results not marked as “questionable” or “weak” were kept. Hits described as “uncharacterized,” “putative,” “unknown,” or “predicted” were not kept.

## Results

### Rate of evolution of ORFan genes and proteins

**Fig. 2:**
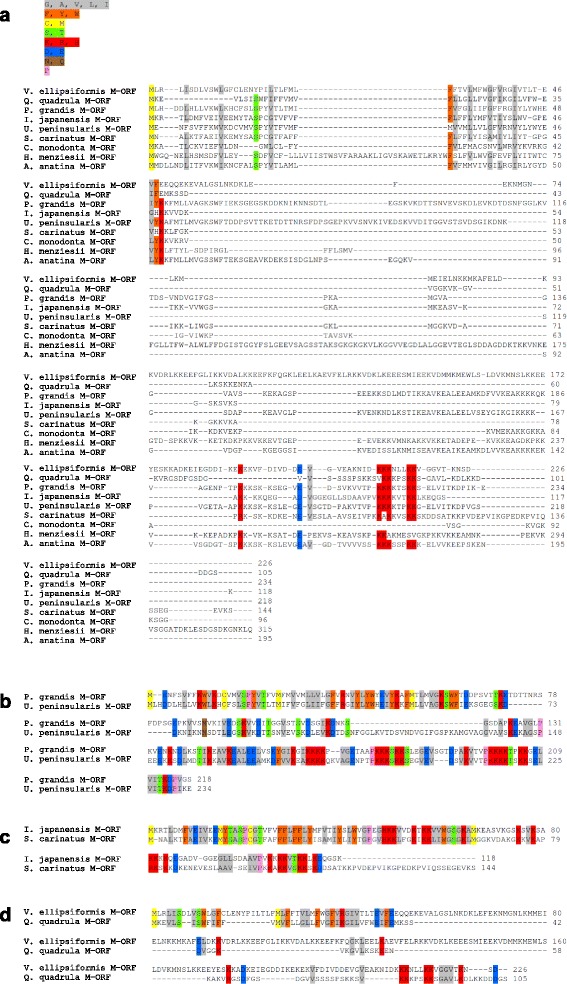
Alignments of M*-ORF* protein sequences. Global alignments and alignments for each subfamily are shown. **a** All M*-ORF* sequences, **b** M*-ORFs* from the subfamily Unioninae, **c** M*-ORFs* from the subfamily Gonideinae, **d** M*-ORFs* from the subfamily Ambleminae. Colour coding is applied to amino acid groups conserved in ≥70 % of sequences. Grey, aliphatic amino acids; orange, aromatic amino acids; yellow, sulfur amino acids; green, amino acids bearing a hydroxyl group; red, basic amino acids; blue, acidic amino acids; brown, amino acids with an amide group; pink, cyclic amino acids

**Fig. 3:**
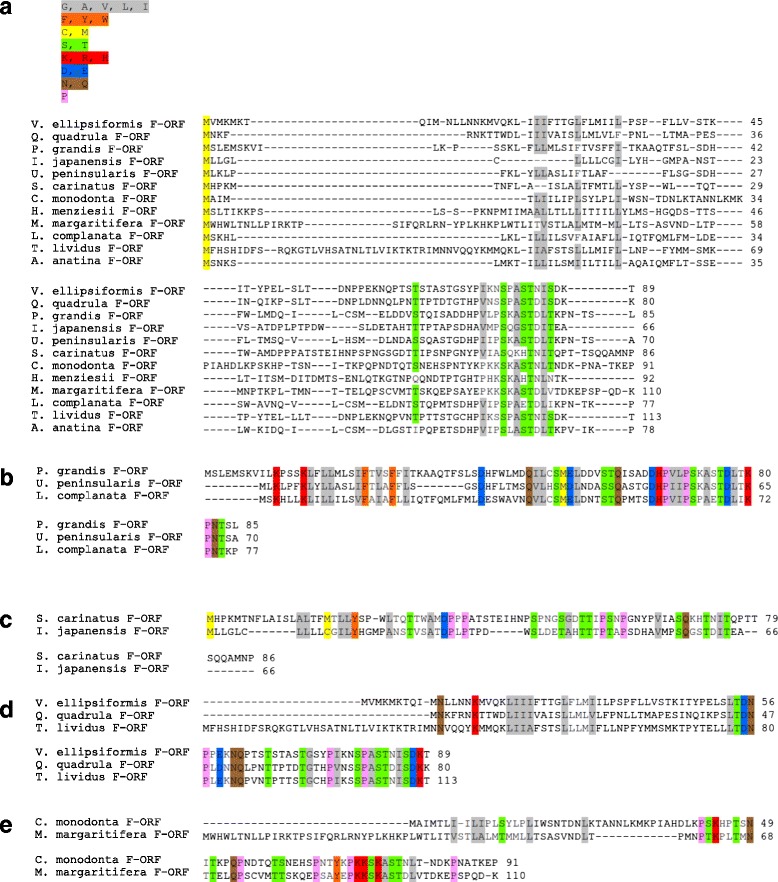
Alignments of F*-ORF* protein sequences. Global alignments and alignments for each subfamily are shown. **a** all F*-ORF* sequences, **b** F*-ORFs* from the subfamily Unioninae, **c** F*-ORFs* from the subfamily Gonideinae, **d** F*-ORFs* from the subfamily Ambleminae (**e**) F*-ORFs* from the subfamily Margaritiferidae. Colour coding is applied to amino acid groups conserved in ≥70 % of sequences. Grey, aliphatic amino acids; orange, aromatic amino acids; yellow, sulfur amino acids; green, amino acids bearing a hydroxyl group; red, basic amino acids; blue, acidic amino acids; brown, amino acids with an amide group; pink, cyclic amino acids

The amino acid sequences of ORFans were generally not well conserved among unionoid species. As seen in Fig. 2, a good comprehensive alignment including all M*-ORF* sequences was not possible due to their high divergence, however, sequences from the same subfamily produced good alignments (Fig. 2b–d). A common feature of M*-ORFs* is that they are all lysine-rich proteins frequently with poly-K strings, a characteristic that is apparently absent in F*-ORF* and H*-ORF* amino acid sequences. Similar to M*-ORF* sequences, F*-ORF* sequences from the same subfamily or family produced better alignments than for all species (Fig. 3). Finally, because phylogenetic analysis indicates that the H*-ORF*s were formed by five independent evolutionary events [15], interspecific alignment is not possible for hermaphrodite ORFans, and alignments between hermaphrodite H*-ORF*s and closely-related gonochoric species F*-ORF*s were mainly of low quality (Additional file 2: Figure S1). In instances where multiple H-*ORF*s were available for a given species of hermaphrodite, these protein sequences were only aligned intraspecifically.

The p-distances for nucleotide and amino acid ORFan sequences as well as the outcome of the test of positive selection are reported in Table 2 (M*-ORF*s and F*-ORF*s) and Table 3 (H*-ORF*s), along with the values for *cox1* and *atp8* sequences taken from the same sex-specific mtDNAs. Table 4 shows the p-distances for within-genus comparisons of F*-ORF*s versus H*-ORF*s. In all cases, the novel ORFs have interspecific p-distances several times higher than *cox1* and higher than *atp8*, which typically represent the slowest- and fastest- evolving mitochondrial protein-coding genes, respectively, in both freshwater mussels and in animals in general [16, 47]. For all groups of sequences, we observed no significant probability of rejecting the null hypothesis of neutral selection in favor of the alternative hypothesis of positive selection. The level of sequence conservation between M vs. M, F vs. F, and F vs. H complete mitochondrial genomes also confirmed that mitochondrial ORFans are the fastest evolving genes in the mtDNA of freshwater mussels with DUI (Additional file 3: Figure S2).

**Table 2:** p-distances (p-D) and standard error (SE) values for mitochondrial M*-orf*s, F*-orf*s, *cox1* and *atp8* in freshwater mussel subfamilies

Subfamily	Gene (N)	Nucleotide		Amino acid		*p*
		p-D	SE	p-D	SE	
Unioninae	F-*orf* (3)	0.355	0.023	0.467	0.047	1.000
	F-*cox1* (2)	0.103	0.007	0.014	0.005	1.000
	F-*atp8* (2)	0.300	0.011	0.333	0.015	1.000
	M-*orf* (2)	0.350	0.018	0.502	0.034	1.000
	M-*cox1* (2)	0.165	0.010	0.094	0.012	1.000
	M-*atp8* (2)	0.250	0.010	0.267	0.013	1.000
Gonideinae	F-*orf* (2)	0.469	0.033	0.692	0.058	1.000
	F-*cox1* (2)	0.132	0.008	0.033	0.008	1.000
	F-*atp8* (2)	0.400	0.025	0.222	0.010	1.000
	M-*orf* (2)	0.384	0.025	0.552	0.044	1.000
	M-*cox1* (2)	0.175	0.009	0.130	0.015	1.000
	M-*atp8* (2)	0.301	0.019	0.421	0.039	1.000
Ambleminae	F-*orf* (3)	0.351	0.024	0.508	0.041	1.000
	F-*cox1* (2)	0.128	0.009	0.033	0.007	1.000
	F-*atp8* (2)	0.278	0.018	0.370	0.031	1.000
	M-*orf* (2)	0.421	0.027	0.687	0.047	1.000
	M-*cox1* (2)	0.179	0.010	0.145	0.015	1.000
	M-*atp8* (2)	0.211	0.012	0.233	0.017	1.000
Margaritiferinae	F-*orf* (2)	0.393	0.029	0.705	0.050	1.000
	F-*cox1* (2)	0.164	0.009	0.068	0.009	1.000

Note: *N* number of sequences used. The probability of rejecting the null hypothesis of strict-neutrality (*d*_N_ = *d*_S_) in favor of the alternative hypothesis (*d*_N_ > *d*_S_) (in the p column) is shown. *d*_S_ and *d*_N_ are the numbers of synonymous and nonsynonymous substitutions per site, respectively

**Table 3:** p-distances (p-D) and standard error (SE) values of mitochondrial H-*orf*s and *cox1* in hermaphroditic freshwater mussels

Species	Gene (N)	Nucleotide		Amino acid		*p*
		p-D	SE	p-D	SE	
*Utterbackia imbecillis*	H-*orf* (7)	0.070	0.008	0.181	0.022	1.000
	*cox1* (2)	0.000	0.000	0.000	0.000	1.000
*Margaritifera falcata*	H-*orf* (4)	0.003	0.002	0.004	0.004	1.000
	*cox1* (2)	0.000	0.000	0.000	0.000	1.000
*Lasmigona compressa*	H-*orf* (2)	0.029	0.007	0.065	0.017	1.000
	*cox1* (2)	0.000	0.000	0.000	0.000	1.000
*Lasmigona subviridis*	H-*orf* (2)	0.016	0.005	0.021	0.010	1.000

Note: *N* number of sequences used. Multiple *cox1* sequences were not available for *L. subviridis.* The probability of rejecting the null hypothesis of strict-neutrality (*d*_N_ = *d*_S_) in favor of the alternative hypothesis (*d*_N_ > *d*_S_) (in the p column) is shown. *d*_S_ and *d*_N_ are the numbers of synonymous and nonsynonymous substitutions per site, respectively

**Table 4:** p-distances (p-D) and standard error (SE) values of mitochondrial F-*orf*s vs H-*orf*s and F*cox1* vs H*cox1* in comparisons between gonochoric vs. closely related hermaphroditic freshwater mussel species

Species	Genes	Nucleotide		Amino acid	
		p-D	SE	p-D	SE
*Utterbackia peninsularis* vs *U. imbecillis*					
	F*-ORF* vs. H*-ORF1*	0.338	0.034	0.691	0.055
	F*-ORF* vs. H*-ORF2*	0.310	0.032	0.721	0.054
	F*-ORF* vs. H*-ORF3*	0.343	0.031	0.743	0.051
	F*-ORF* vs. H*-ORF4*	0.335	0.034	0.729	0.054
	F*-ORF* vs. H*-ORF5*	0.333	0.031	0.714	0.052
	F*-ORF* vs. H*-ORF6*	0.333	0.031	0.714	0.052
	F*-ORF* vs. H*-ORF7*	0.310	0.030	0.739	0.055
	**Mean**	**0.329**	**0.030**	**0.722**	**0.052**
	F*-COX1* vs. H*-COX1-1*	0.547	0.012	0.020	0.006
	F*-COX1* vs. H*-COX1-2*	0.547	0.012	0.020	0.006
	**Mean**	**0.547**	**0.0012**	**0.020**	**0.006**
*Margaritifera margaritifera* vs *M. falcata*					
	F*-ORF* vs. H*-ORF1*	0.339	0.025	0.491	0.048
	F*-ORF* vs. H*-ORF2*	0.336	0.026	0.491	0.049
	F*-ORF* vs. H*-ORF3*	0.358	0.024	0.491	0.049
	F*-ORF* vs. H*-ORF4*	0.336	0.026	0.491	0.049
	**Mean**	**0.342**	**0.025**	**0.491**	**0.049**
	F*-COX1* vs. H*-COX1-1*	0.469	0.022	0.000	0.000
	F*-COX1* vs. H*-COX1-2*	0.469	0.021	0.000	0.000
	**Mean**	**0.469**	**0.021**	**0.000**	**0.000**
*Lasmigona complanata* vs *L. compressa*					
	F*-ORF* vs. H*-ORF1*	0.218	0.028	0.394	0.059
	F*-ORF* vs. H*-ORF2*	0.255	0.027	0.395	0.055
	**Mean**	**0.237**	**0.027**	**0.395**	**0.057**
*Lasmigona complanata* vs *L. subviridis*					
	F*-ORF* vs. H*-ORF1*	0.269	0.029	0.429	0.054
	F*-ORF* vs. H*-ORF2*	0.295	0.029	0.442	0.055
	**Mean**	**0.282**	**0.029**	**0.436**	**0.054**
*Toxolasma lividus* vs *T. parvum*	F*-ORF* vs. H*-ORF*	0.443	0.027	0.736	0.044

Note: Bold numbers indicate mean values

### Conserved structures in ORFan protein sequences

**Fig. 4:**
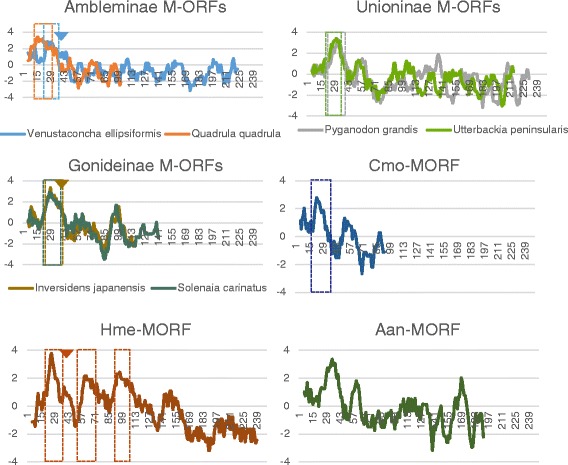
Hydrophobicity profiles of M*-ORFs*. Boxes indicate predicted TM helices, arrowheads indicate the end of predicted SPs. X-axis is amino acid position, Y-axis is hydrophobicity. Aan, *Anodonta anatina*; Cmo, *Cumberlandia monodonta*; Hme, *Hyridella menziesii*

**Fig. 5:**
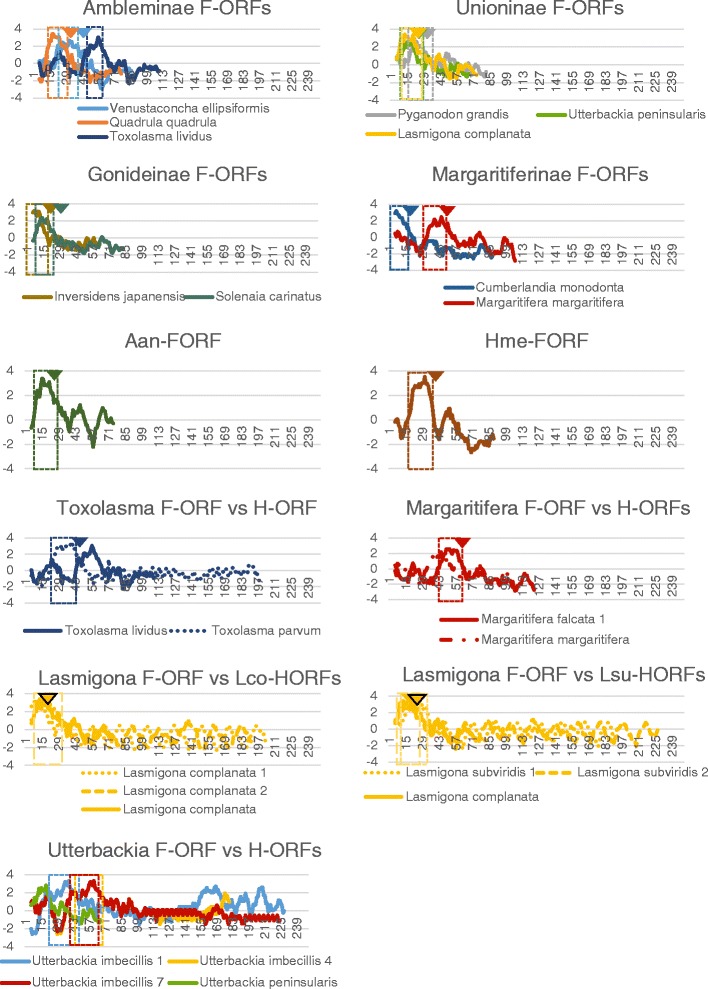
Hydrophobicity profiles of F*-ORFs* (*top*) and H*-ORFs* vs. F-*ORFs* (*bottom*). Boxes indicate predicted TM helices, arrowheads indicate the end of predicted SPs. X-axis is amino acid position, Y-axis is hydrophobicity. Aan, *Anodonta anatina*; Cmo, *Cumberlandia monodonta*; Hme, *Hyridella menziesii;* Lco-HORFs, *Lasmigona compressa* H-*ORFs*; Lsu-HORFs, *Lasmigona subviridis* H-*ORFs*. For hermaphroditic species, only sequences with different hydrophobicity profiles are shown

One TM helix was predicted near the N-terminus of all M*-ORF*s (Fig. 4 and Additional file 1: Table S2), except for *H. menziesii* M*-ORF* sequence, for which one N-terminal and two additional TM helices were predicted. PrediSi and SignalP both returned predicted SPs for all M*-ORF* sequences, however, the programs rarely agreed about the length of the predicted SP (Additional file 1: Table S3). One TM helix near the N-terminus was also predicted in all F*-ORF* sequences, with an SP predicted to overlap with this TM structure, except in the case of the *T. lividus* F*-ORF* for which the location of the SP was uncertain (Fig. 5 and Additional file 1: Tables S2 and S3). All H*-ORF*s contained one predicted TM helix near the N-terminus as well, except for *U. imbecillis* H*-ORF*s that contained multiple predicted TM helices, but only the location of the first TM helix (closest to the N-terminus) was predicted with high confidence (Fig. 5 and Additional file 1: Table S4). *U. imbecillis* H*-ORF*s also returned variable SP predictions, whereas all other H*-ORF* sequences contain one predicted SP overlapping with the N-terminal TM helix (Additional file 1: Table S5). Although they could not be confidently aligned (see Additional file 3: Figure S2), F*-ORF*s and H*-ORF*s of closely related species showed some structural similarities in the localization of the TM helices and SPs (Fig. 5). Importantly, all H*-ORF*s contain tandem repeats (*L. compressa* possesses between 3 to 7 tandemly repeated sequence motifs of 20 or 21aa; *L. subviridis* 7 to 9 repeats of 17aa; *T. parvum* 2 to 3 repeats of 47aa; *M. falcata* 2 to 3 repeats of 11aa; and *U. imbecillis* 2 to 4 repeats of 11 or 21aa), which are not found in F*-ORF*s and account for most of the difference in length between F*-ORF*s and H*-ORF*s of closely related species (Additional file 3: Figure S2).

### Motif and functional domain scans: frequently recurring HHpred hits and potential ligand-binding sites

**Fig. 6:**
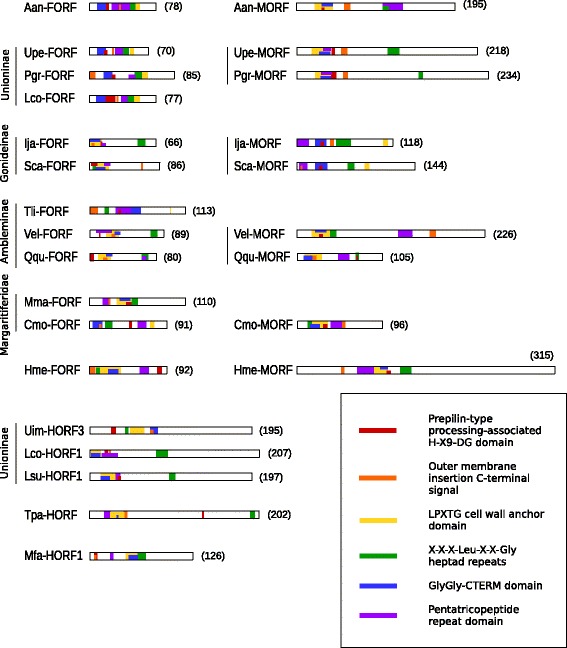
Position of motifs frequently recurring in HHpred hits. Protein length in amino acids is indicated in parentheses. One representative sequence was chosen for each hermaphroditic species

Six HHpred hits consistently appeared highly ranked in the results of M*-ORF*s, F*-ORF*s and H*-ORF*s: (1) prepilin-type processing-associated H-X9-DG domain, (2) outer membrane insertion C-terminal signal, (3) LPXTG cell wall anchor domain, (4) X-X-X-Leu-X-X-Gly heptad repeats, (5) GlyGly-CTERM domain, and (6) a pentatricopeptide repeat (PPR) domain. Probabilities were all >92 % (which the developers state can be interpreted literally [40]), and ranks were typically 1–6 in variable order, with very few of these hits falling outside of the top 10 (Additional file 1: Tables S6 and S7). Fig. 6 shows the position of these six hits in the protein sequences analyzed. Other less recurring motifs and domains are presented in detail in Additional file 1: Table S8 and S9.

Inferred homologies and prediction of binding sites both indicated that ORFan proteins may bind several ligands (Table 5). All M*-ORF*s returned hits to protein-binding, DNA-binding and RNA-binding proteins, all F*-ORF*s returned hits to protein-binding and RNA-binding proteins, and all H*-ORF* sequences returned hits to protein-binding, DNA-binding, RNA-binding and carbohydrates-binding proteins.

**Table 5:** Summary of hits to ligand-binding sites in M-*ORF*s, F-*ORF*s and H-*ORF*s

Protein	DNA	RNA	Protein	Carbohydrate	Ion	Lipid	ATP
Vel-MORF	X	X	X	X		X	X
Qqu-MORF	X	X	X		X		
Pgr-MORF	X	X	X	X	X		X
Ija-MORF	X	X	X		X		X
Upe-MORF	X	X	X		X		X
Sca-MORF	X	X	X		X		X
Cmo-MORF	X	X	X		X	X	
Hme-MORF	X	X	X		X	X	X
Aan-MORF	X	X	X	X	X		
**Total**	**9**	**9**	**9**	**3**	**8**	**3**	**6**
Vel-FORF	X	X	X	X	X		X
Qqu-FORF	X	X	X	X	X	X	X
Pgr-FORF	X	X	X	X	X		X
Ija-FORF	X	X	X	X	X		
Upe-FORF	X	X	X		X		X
Sca-FORF	X	X	X		X	X	X
Cmo-FORF		X	X	X			
Hme-FORF	X	X	X	X			X
Lco-FORF	X	X	X		X	X	X
Tli-FORF	X	X	X	X	X		X
Mma-FORF	X	X	X		X		
Aan-FORF	X	X	X		X	X	
**Total**	**11**	**12**	**12**	**7**	**10**	**4**	**8**
Uim-HORF1 - 3	X	X	X	X	X		X
Uim-HORF4 - 7	X	X	X	X	X		
Mma-HORF1, 2, 4	X	X	X		X		X
Mma-HORF3	X	X	X		X		
Tpa-HORF	X	X	X	X	X	X	X
Lco-HORF1	X	X	X		X		
Lco-HORF2	X	X	X	X	X	X	
Lsu-HORF1 - 2	X	X	X	X	X		
**Total**	**14**	**14**	**14**	**10**	**14**	**2**	**6**

Note: Bold numbers indicate mean values

### Prediction of molecular function: hits to viral proteins

As mentioned above, a recent study proposed a viral origin for the mitochondrial ORFans in DUI bivalves [18]. Therefore, we first scanned our results obtained with all programs for protein function prediction, i.e. BLAST, HMMER, HHpred, @tome2, I-TASSER, and PredictProtein, for supported hits to viral proteins (Table 6). For H*-ORF*s, *M. falcata* primarily returned envelope proteins, *L. subviridis* returned capsid and envelope proteins, *L. compressa* returned proteins that interact with receptors, *T. parvum* returned a protein that regulates the degradation of a receptor, and *U. imbecillis* returned capsid proteins and other structural proteins. M*-ORF*s returned nucleoproteins (*A. anatina* and *H. menziesii*), membrane proteins (*I. japanensis* and *S. carinatus*), and proteins with a role in replication, life cycle, and apoptosis (*A. anatina, U. peninsularis, I. japanensis* and *V. ellipsiformis*). F*-ORF* hits were mostly parts of the viral capsid and viral envelope (*S. carinatus, T. lividus* and *M. margaritifera*), receptors/fibre proteins (*M. margaritifera* and *C. monodonta*), or proteins involved in cell cycle and translation (*P. grandis* and *I. japanensis*).

**Table 6:** Hits to viral proteins from structural prediction analyses

Gene	Hit	Function	Position
Aan-MORF	Nucleoprotein, *Andes virus* [Atome 2; 41.16]	Nucleoprotein	NA
	Regulatory protein MNT, *Enterobacteria phage P22* [Atome 2; 21.14]	Gene regulation	NA
Upe-MORF	Uncharacterized protein 56B, *Sulfolobus islandicus* [Atome 2; 27.96]	Transcription repressor	NA
Pgr-MORF	Matrix protein 1, *Influenza A virus* [Atome 2; 39.16]	Matrix protein	NA
	Helix-destabilizing protein, *Enterobacteria phage T7* [Atome 2; 18.55]	DNA binding protein	NA
Ija-MORF	Nonstructural protein 5A, *Bovine viral diarrhea virus 1-CP7* [Atome 2; 33.37]	Membrane protein	NA
	Functional anti-apoptotic factor vBCL-2 homolog, *Human herpesvirus 8* [Atome 2; 27.14]	Apoptosis	NA
Sca-MORF	Nonstructural protein 5A, *Bovine viral diarrhea virus 1-CP7* [Atome 2; 22.35]	Membrane protein	NA
Vel-MORF	Macrophage galactose N-acetyl-galactosamine specific lectin 2 [Hhpred; 93.40]	C-type lectin	20–171
	RhUL123, *Macacine herpesvirus 3* [I-TASSER; TM score 0.671]	Viral life cycle	NA
	Phosphoprotein, *Measels virus* [Atome 2; 49.33]	Unknown function	NA
	Tail needle protein gp26, *Enterobacteria phage P22* [Atome 2; 48.96]	Fibrous protein	NA
Qqu-MORF	Virion RNA polymerase, *Bacteriophage n4* [I-TASSER; TM score 0.542]	Transferase	NA
Cmo-MORF	No hits to viral proteins		
Hme-MORF	Nucleoprotein, *Andes virus* [Atome 2; 63.91]	Nucleoprotein	NA
Aan-FORF	No hits to viral proteins		
Upe-FORF	BM2 protein, *Influenza B virus (B/Taiwan/70061/2006)* [Atome 2; 42.29]	Transport protein	NA
Pgr-FORF	V-cyclin, *Human herpesvirus 8* [I-TASSER; norm. TM score 0.517]	Cell cycle	NA
Lco-FORF	Herpes simplex virus protein ICP47, *Herpes simplex virus (type 1/strain 17)* [Atome 2; 46.61]	Membrane protein	NA
Ija-FORF	Non-structural RNA-binding protein 34, *Simian rotavirus A/SA11* (2) [Atome 2; 48.04, 28.60]	Translation	NA
Sca-FORF	Major capsid protein (protein P3), *Enterobacteria phage PRD1* [Atome 2; 80.01]	Capsid protein	NA
Tli-FORF	Envelope protein E, *Dengue virus 2 Thailand/16681/84* [Atome 2; 46.45]	Envelope protein	NA
Vel-FORF	V1V2 region of HIV-1 on 1FD6 scaffold, *Human immunodeficiency virus 1* [Atome 2; 57.65]	Immune system	NA
Qqu-FORF	HIV-1 matrix protein, *Human immunodeficiency virus 1* (2) [Atome 2; 83.13, 72.79]	Matrix protein	NA
Mma-FORF	ODV-E18: Occlusion-derived virus envelope protein ODV-E18 (2) [Hhpred; 72.05, 62.79]	Envelope protein	21–62
	Adenovirus fibre, *Human adenovirus 2* [Atome 2; 27.29]	Fibre protein	23–55
	Fibre protein 2 (receptor-binding domain), *Human adenovirus 41* [I-TASSER; 18.06]	Fibre protein, receptor binding	NA
			NA
Cmo-FORF	Virus attachment protein globular domain (49835) SCOP seed sequence: d1h7za [Hhpred; 21.78]	Viral attachment, entry into host cell	50–68
	Adenovirus fibre protein; cell receptor recognition, receptor, *Human adenovirus type 3* [Hhpred; 21.71]	Fibre protein, Cell receptor recognition	44–68
	Fibre protein, *Human adenovirus 37* [Atome 2; 31.21]		NA
	Fibre protein, *Human adenovirus 2* [Atome 2; 30.90]		NA
	Type 5 fibre protein, *Human adenovirus 5* [Atome 2; 30.46]		NA
	Fibre protein, *Human adenovirus 41* [Atome 2; 24.60]		NA
Hme-FORF	Nucleoprotein, *Influenza A virus* [Atome 2; 80.49]	RNA binding protein	NA
Uim-HORFs	HIV-1 capsid, *Human immunodeficiency virus 1* [I-TASSER; TM score 0.513]	Capsid protein	NA
	Gag Polyprotein, *Human immunodeficiency virus 1* [I-TASSER; TM score 0.510]	Precursor protein	NA
	Capsid protein P24, *Human immunodeficiency virus type 2* [I-TASSER; TM score 0.504]	Capsid protein	NA
	Nucleoprotein, *Andes virus* [Atome 2; 44.18]	Nucleoprotein	NA
	Protein ICP47, *Herpes simplex virus* [Atome 2; 37.48]	Membrane protein	NA
	LdOrf-129 peptide, *Lymantria dispar multiple nucleopolyhedrovirus* (2) [BLASTP, PSIBLAST; 2e-06, 7e-10]	Structual protein	74–144
	ORF-132 protein, *Lymantria dispar multiple nucleopolyhedrovirus* (2) [BLASTP, PSIBLAST; 4e-06, 2e-09]	Unknown	74–131
	orf-126 protein, *Lymantria dispar multiple nucleopolyhedrovirus* [PSIBLAST; 4e-08]	Unknown	72–140
	Central variable region protein, *African swine fever virus* [PSIBLAST; 6e-08, 7e-07]	Unknown	60–154
	Central variable region protein, *African swine fever virus* [PSIBLAST; 7e-08]	Unknown	60–130
	pB602L, *African swine fever virus tick/South Africa/Pretoriuskop Pr4/1996* [PSIBLAST; 8e-08]	Structural capsid protein, chaperone in capsid assembly *(several hits)*	65–153
	U1, *Hyposoter didymator ichnovirus* [PSIBLAST; 3e-07]	Spliceosomal RNA	65–137
	gp7, *Salmonella phage epsilon15* [I-TASSER; norm. Z-score 1.32]	DNA transfer protein	NA
	Long tail fibre protein p37, *Enterobacteria phage T4* [I-TASSER; norm. Z-score 1.30]	Fibre protein	88–166
	RhUL123, *Macacine herpesvirus 3* [I-TASSER; TM score 0.617]	Viral life cycle	NA
	Nucleoprotein, *Andes virus* [Atome 2; 39.59]	Nucleoporin *(several hits)*	NA
	LdOrf-129 peptide, *Lymantria dispar multiple nucleopolyhedrovirus* [PSIBLAST; 8e-10]	Structual protein	NA
	ORF-132 protein, *Lymantria dispar multiple nucleopolyhedrovirus* [PSIBLAST; 5e-09]	Unknown	NA
	DNA stabilization protein, *Salmonella phage HK620* [I-TASSER; Z-score 1.09]	DNA binding & stabilization	87–188
	Hexon protein, *Human adenovirus 5* [I-TASSER; Z-score 1.01]	Major coat protein	139–223
	Human T-cell leukemia virus type II matrix protein, *Human T-lymphotropic virus 2* [I-TASSER; Z-score 1.00]	Matrix protein	NA
	Herpes simplex virus protein ICP47, *Herpes simplex virus (type 1/strain 17)* [Atome 2; 1.72]	Blocks the major histocompatibility complex class I antigen presentation pathway	NA
Lco-HORFs	Long tail fiber protein P37, *Enterobacteria phage T4* [I-TASSER; Z-score 1.01]	Receptor binding viral protein	NA
	Capsid protein, *Rubella virus strain M33* [Atome 2; 83.05]	Capsid component	NA
	VPU protein, *Human immunodeficiency virus 1* [Atome 2; 43.79]	Regulates degradation of receptor molecule CD4 *(several hits)*	NA
Lsu-HORFs	Major capsid protein, *Synechococcus phage Syn5* [I-TASSER; Z-score 1.66]	Capsid component	NA
	RhUL123, *Macacine herpesvirus 3* [I-TASSER; TM score 0.547]	Viral life cycle	69–195
	Herpes virus major outer envelope glycoprotein (BLLF1) [BLASTP/PSIBLAST; 2.73e-03]	Envelope protein	NA
	Short tail fiber protein, *Enterobacteria phage T4* [I-TASSER; Z-score 2.14]	Structural protein	NA
	Major capsid protein, *Synechococcus phage Syn5* [I-TASSER; Z-score 2.19]	Capsid component *(several hits*)	NA
	Coat protein, *Enterobacteria phage P22* [I-TASSER; TM score 0.520]	Coat component	NA
	Herpes virus major outer envelope glycoprotein (BLLF1) [BLASTP/PSIBLAST; 4.85e-04]	Envelope protein	NA
Tpa-HORF	VPU protein (Trans-membrane domain), *Human immunodeficiency virus 1* [Atome 2; 33.16]	Regulates degradation of receptor molecule CD4 *(several hits)*	NA
Mfa-HORFs	ODV-E18: Occlusion-derived virus envelope protein ODV-E18 [Hhpred; 74.97]	Envelope protein *(several hits)*	33–73
	Herpes_TK_C: Thymidine kinase from Herpesvirus C-terminal, *Herpesvirus* (2) [Hhpred; 48.70, 48.13]	ATP binding, thymidine kinase *(several hits)*	33–73
	Adenovirus fibre, *Human adenovirus 2* [Atome 2; 34.11]	Fibre protein, receptor binding *(several hits)*	NA

Note: I-TASSER: Norm. Z-score > 1 indicates a good alignment; TM-score > 0.5 indicates a similar fold with query [46]; position = amino acid position in the query sequence; *NA* not applicable

### Prediction of molecular function: hits to mitochondrial proteins

Besides viral hits, most of the sequences analyzed also returned hits to proteins involved in energy production, including proteins of the mitochondrial electron transport system, so we tested the similarity of the ORFan proteins to standard mtDNA-encoded ones with BLAST searches. Our analyses predicted M*-ORF*s mostly as subunit 5 of the NADH-Ubiquinone Oxidoreductase complex I of the mitochondrial electron transport chain (*NAD5*) for 5 species out of 9, and/or *ATP8* of the ATP synthase complex V for 5 species, but only with very low support (i.e. E-values ranged between 6e-04 and <1.0, the limit chosen for this analysis) (see Table 7). This latter result was also supported by a moderately significant domain hit identified in *C. monodonta*, i.e. pfam02326 or Mt_ATP-synt_B, a superfamily that corresponds to the subunit 8 of the F0 complex of plants (E-value 4e-03). Specifically, *C. monodonta* M-*ORF* shares similarities in its N-terminal amino-acid sequence with *ATP8* sequences from plant but also from non-plant species (Additional file 4: Figure S3). However, similar results were not found for other M-*ORF* protein sequences (data not shown).

**Table 7:** List of BLAST hits for mitochondrial ORFans in freshwater mussels searched against NCBI NRDB mitochondrial proteins

Species Name	M*-ORF*s	F-*ORF*s	H-*ORF*s
*Anodonta anatina*	NAD7 (0.61)	---	
	---	*atp9 (0.19)*	
*Cumberlandia monodonta*	ATP8 (0.81)	---	
	---	*nad2 (6e-08)*	
*Hyridella menziesi*	ATP8 (0.61)	NAD2 (0.33)	
	*nad4 (6e-04)*	*nad2 (0.022)*	
*Lasmigona complanata*		---	
		*nad2 (0.094)*	
*Lasmigona compressa*			F-ORF (4e-05)
			*f-orf (2e-05)*
*Lasmigona subviridis*			F-ORF (6e-09)
			*f-orf (2e-05)*
			*nad1 (0.64)*
*Inversidens japanensis*	ATP8 (0.62)	---	
	*nad5 (0.001)*	*nad2 (0.22)*	
	*atp8 (0.048)*		
	*cox1 (0.15)*		
*Margaritifera falcata*			COX1 (0.94)
			---
*Margaritifera margaritifera*		NAD5 (0.093)	
		NAD2 (0.23)	
		*nad2 (0.15)*	
*Pyganodon grandis*	NAD5 (0.046)	---	
	*atp9 (0.30)*	*cytb (0.13)*	
*Quadrula quadrula*	NAD5 (0.026)	NAD5 (0.31)	
	ATP8 (0.070)	*nad2 (0.56)*	
	*atp9 (0.30)*		
*Solenaia carinatus*	COX1 (0.41)	---	
	NAD5 (0.99)	*nad2 (0.018)*	
	*nad5 (0.33)*		
*Toxolasma lividus*		---	
		---	
*Toxolasma parvum*			F-ORF (0.020)
			---
*Utterbackia imbecillis*			---
			*nad2 (0.061)*
*Utterbackia peninsularis*	NAD5 (0.38)	---	
	*nad2 (0.31)*	*cox1 (0.056)*	
*Venustaconcha ellipsiformis*	NAD4 (0.19)	NAD4 (0.55)	
	CYTB (0.21)	*nad2 (0.14)*	
	ATP8 (0.94)		
	*nad4 (0.15)*		

Note: Protein name and (e-values <1.0) identified using PSI-BLAST and *tBLASTx* are indicated above in capital letters and below in italics, respectively. Hits to freshwater mussel mitochondrial ORF homologs are not presented, except for the highly divergent H-*ORF*s

For F*-ORF*s, the most recurring hit (8 species out of 12) was subunit 2 of the mitochondrial complex I (*NAD2*), again with quite low support (E-values ranged between 6e-08 and <1.0). The lowest E-value was obtained with the F*-ORF* sequence of *C. monodonta*, but only for a short fragment of 20 amino acids sharing similarities with the *NAD2* protein of the trematode *Fasciola* sp. The alignment of *C. monodonta* F*-ORF* and *NAD2* protein sequences revealed poor similarities (Additional file 5: Figure S4), and identical results were also obtained in other studied gonochoric species (data not shown). Finally, BLAST searches of H*-ORF*s principally identified F*-ORF*s (3 species out of 5), with moderate E-values (Table 7).

### Profile HMM – sequence comparisons for F-*ORF*s and M-*ORF*s

The hmmsearch analyses with HMM profiles for F-*ORF* and M-*ORF* alignments gave different numbers of hits for default vs. custom profiles. In general, the custom profiles were more “stringent” in terms of hit yield among all databases analysed, giving fewer total results than the default ones. Except for one hit for the M-*ORF* profiles, freshwater mussel ORFan sequences were the only significant hits (E-value <0.001) returned for all profiles, and they will not be considered. Therefore, we will describe all the hits other than unionoids ORFans (even those with E-values higher than the cutoff) in terms of functional recurrence. Results are presented in Additional file 1: Table S10 and S11.

Overall, F-*ORF* hits for both profiles are related to membrane association, virus life cycle, and interaction with nucleic acids (Additional file 1: Table S10 and S11). The M-*ORF* default profile frequently returned hits associated with membranes, related to energy production in bacteria or eukaryotes, transport or movement, or other functions related to membranes (Additional file 1: Table S10 and S11). The Excalibur domain protein, predicted two times with borderline significance (E-values 0.0011 and 0.0018), also has functions in DNA binding and repair and transcription regulation. Other recurring predicted functions are interaction with RNA (pre-rRNA processing, translation initiation, tRNA modification, poly-(A) RNA binding for nuclear import, posttranscriptional expression regulation) and with amino acids and proteins (protein transport, protein modification, or involvement in cytoskeleton rearrangements). Some hits suggest the possible insertion of DNA from foreign sources such as viruses (e.g. hits to viral delta antigens of hepatitis delta virus that are related to viral life cycle, i.e. invasion in host cell and nucleus, replication) and bacteria (a transposition protein gene from *E. coli* Tn7 transposon). The M-*ORF* custom profile returned four additional results, all involved in protein and/or membrane interactions.

### Prediction of molecular function (all sequences, all programs except hmmsearch)

**Fig. 7:**
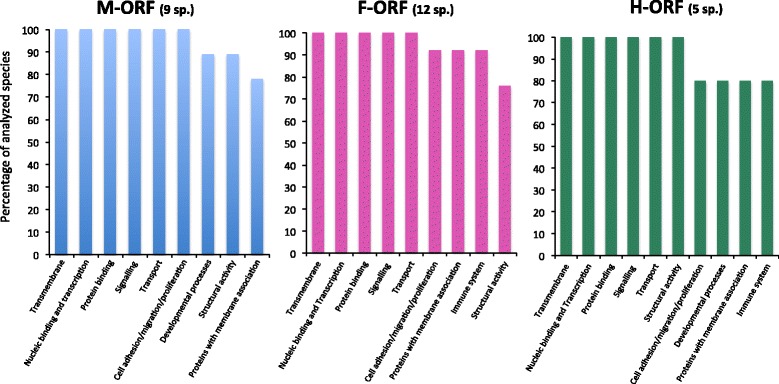
Most frequent categories of hits for biological processes or molecular functions for freshwater mussel mitochondrial ORFans. Categories presented are those returned for over 75 % of all analyzed species for each ‘sex’ (the number of analyzed species for each sex is indicated in parentheses). Blue, M-*ORF*; pink, F-*ORF*; green, H-*ORF*

Finally, we compiled the results obtained for all ORFans with all other programs for protein function prediction (i.e. BLAST, HHpred, @tome2, I-TASSER, and PredictProtein). Fig. 7 summarizes the most frequent categories of hits for biological processes or molecular functions for freshwater mussel mitochondrial ORFans (i.e. those returned for over 75 % of all analyzed species for each ‘sex’) and Additional file 6: Figure S5 and Additional file 1: Table S12-S37 contain detailed hits and recurring functions (i.e. biological processes, cellular components/subcellular localizations and molecular functions). Overall, the most common hits for all M*-ORF*s, F*-ORF*s and H*-ORF*s were transmembrane proteins, proteins involved in nucleic acid binding and transcription, protein binding proteins, and proteins involved in cellular signalling and transport (Fig. 7). In particular, all M*-ORF*s returned hits to proteins involved in cell adhesion, migration and proliferation, and the predicted subcellular localizations for M*-ORF*s were membranes and mostly organelles (endoplasmic reticulum, mitochondria, Golgi and nucleus). Other hits for M*-ORF*s included proteins related to developmental processes (e.g. embryonic development) and structural activity (Figs. 6 and 7 and Additional file 1: Table S10-S37).

The most common hits for F*-ORF*s included proteins with membrane association (e.g. proteins involved in trafficking and transport functions such as SNAP receptors and kinases). Many hits also pointed to a role in immune response. The mitochondria, Golgi, and ER were predicted subcellular localizations for F*-ORF*s (an extracellular localization was also suggested) (Fig. 7 and Additional file 1: Tables S10-S37). For H*-ORF*s, structural proteins, particularly collagen and collagen-like proteins were the most common categories, closely followed by membrane-associated proteins, proteins involved in developmental processes and immune response. An extracellular localisation was also suggested for H*-ORF*s (Fig. 7 and Additional file 1: Tables S10-S37).

## Discussion

### Evolution of freshwater mussel ORFan sequences and protein structures

One general feature observed in mitochondrial ORFan sequences of marine [18] and freshwater bivalves with DUI (present study) is their higher p-distance values at the amino acid level compared to their own nucleotide sequences, suggesting a rapid rate of evolution. However, the null hypothesis of strict-neutrality (*d*_N_ = *d*_S_) was not rejected in favor of the alternative hypothesis of positive selection (*d*_N_ > *d*_S_) (although *d*_N_ > *d*_S_ is an extremely conservative test that may miss instances in which positive selection is happening [48]). Despite low sequence conservation, M*-ORF* and F*-ORF* proteins appear structurally conserved among species, suggesting that their biological functions might be conserved as well.

Compared to F-*ORF*s from gonochoric species, H*-ORF*s from hermaphroditic unionoids contain repeat units and sometimes different hydropathy profiles (e.g. *U. imbecillis* vs. *U. peninsularis*). One possible mechanism for the duplication of repeats independently in the H*-orf* sequences is slippage due to DNA hairpins, a common mechanism implicated in the creation of short protein repeats [49, 50]. These distinctive features of the five H*-ORF*s could indicate changes of function from that of the homologous F*-ORF*s in gonochoric species. The high level of amino acid sequence and structural similarities of the H*-ORF* protein within species, as well as its recent detection in the transcriptome of the hermaphroditic species *U. imbecillis* (Capt et al. unpublished) suggest that it is functional.

Proteins that contain tandem repeats frequently interact with other proteins or ligands such as DNA or RNA (e.g. [50, 51]). A classic example in organelles is the pentatricopeptide repeat (PPR) protein family, and PPR hits were found in all ORFan protein sequences using HHpred. PPR proteins contain variable numbers of tandem repeats and function in transcription, RNA processing, splicing, stability, editing, and translation [51]. Interestingly, PPR proteins are key elements of the only known sex determination system in which the mitochondrial DNA is involved, i.e. in hermaphroditic plants exhibiting cytoplasmic male sterility (CMS) [51]. PPR proteins appear to function as nuclear-encoded restorers of fertility in CMS plants, which suppress mtDNA-encoded factors that inhibit the production of viable pollen [51]. It has been hypothesized that in unionids with DUI the loss of the M mitochondrial genome and macromutations in the F*-orf* gene (i.e. acquisition of tandem repeats) could enable an individual to produce both sperm and eggs leading to hermaphroditism [16].

### Conserved motifs and domains: mitochondrial export of ORFan proteins

In this unionoid-specific study, we found the same pattern of homology detection hits for M*-ORF*s and F*-ORF*s as presented in Milani et al. [18], i.e. motifs and domains involved in cell membrane/surface anchoring, transcription and post-transcriptional processes. Two notable differences involved hits involved in cleavage/methylation and protein transport.

So far, the protein products of the F*-orf* and M*-orf* genes in unionoids have been studied only in the species *Venustaconcha ellipsiformis* [16]. Using immunoelectron microscopy, the F-*ORF* protein has been localized not only to egg mitochondria, but also to the nuclear envelope and the egg nucleoplasm [16]. Interestingly, the F*-ORF* protein was also found on the inner mitochondrial membrane of some sperm mitochondria [52], which are thought to contain only M mtDNA [53]. Because small proteins may diffuse into the nucleus without a specific targeting signal, the nuclear localization in eggs may not be specific, however, mitochondrial localization depends on an N-terminus signal peptide [54, 55]. Because the F mtDNA is not present in DUI bivalve sperm mitochondria [53], either there is a version of the F*-orf* gene in the nuclear genome (or another nuclear-encoded gene product is capable of reacting with the antibody), or the mtDNA-encoded F*-ORF* protein is exported from F-type mitochondria and imported via an N-terminal signal peptide into sperm mitochondria. Examination of a freshwater mussel nuclear genome (currently underway in our laboratory) will help test these hypotheses.

Subcellular localization of the M*-ORF* protein has not yet been studied, but our *in silico* detection of nuclear localization signals in several M*-ORF* sequences, and of hits related to protein movement, are consistent with the hypothesis that this protein is exported from the organelle. Such results have been observed in the venerid clam *Ruditapes philippinarum*, in which the M*-ORF* protein was immunolocalized in both mitochondria and the nucleus of sperm [19]. Hence, mitochondrial ORFan proteins in DUI bivalves likely have multiple roles in different cellular compartments ([16, 18, 19], present study), explaining the existence of functional domains for interacting with diverse cellular elements.

The process for mitochondrial exporting of F*-ORF* or M*-ORF* proteins remains unexplained. In fact, while mitochondrial import of proteins is well-studied in eukaryotes [56], the process of mitochondrial export is still obscure (e.g. [57]). The export of cell death effectors [58], retrograde signals *humanin* and MOTS-c [59], and small peptides to trigger retrograde nuclear signalling in mitochondrial unfolded protein response in mammals are all partially characterized, but mitochondrial protein export of larger molecules is relatively unstudied (e.g. [57, 60]). Further work is needed to better understand mitochondrial export in animals.

### Putative origin for freshwater mussel mitochondrial ORFans

As mentioned, prior *in silico* analyses pointed to a possible viral origin of bivalve mitochondrial ORFans, although the probability of some hits were low and the regions of similarity were short [18]. Except for the M*-ORF* of *C. monodonta* and the F*-ORF* of *A. anatina*, our results revealed the presence of at least one viral hit for each sequence analyzed (consistent with the viral hypothesis), but with low probability values and short regions of similarity. We also consistently obtained hits with stronger probability values for bacterial or metazoan genes (Table 6 and Additional file 1: Tables S12-S37). Consequently, we cannot exclude other organisms or other processes [61–63] as the source of these ORFan genes. For example, gene duplication is thought to be the mechanism underlying the origin of most novel genes, and thus represents one of the most important processes for functional innovation during evolution [62]. Interestingly, several sequences returned hits to proteins involved in mitochondrial energy production, including proteins of the electron transport system, suggesting that duplication and neofunctionalization of a mitochondrial gene could be the source of freshwater mussel mitochondrial ORFans. Several M*-ORF* sequences returned hits to the subunit *ATP8* of the mitochondrial ATP synthase complex V (Table 7), and M-*ORF* profiles to subunit b of bacterial ATP synthase. These results are interesting for two reasons. First, the *atp8* and M*-orf* genes occur beside one another in a region corresponding to one of the three gene order rearrangements observed between female and male mtDNAs of freshwater mussels [16]. Second, the *atp8* gene is highly modified or reported as missing in other bivalve species with DUI due to its short length and rapid evolution causing difficulties in annotation (e.g. [64–66]). It is conceivable that a duplication event (as described in several other animal mtDNAs [67]) of the region containing the *atp8* gene occurred in an ancestral freshwater mussel species with DUI. One of the two duplicate *atp8* copies could have evolved new male-specific functional properties, giving rise to the M*-orf* gene. The identification of a conserved domain of the Mt_ATP-synt_B superfamily in the M-*ORF* protein sequence of *C. monodonta*, i.e. a domain found at the N terminus of subunit 8 of the F0 complex of mitochondrial ATP synthases from plants and algae, also provides further support for the above scenario (Additional file 4: Figure S3). In a variety of plant species, this N-terminal conserved domain is not only found in *ATP8* but also in CMS proteins (coupled to novel C-terminus domains as a result of mt genome rearrangements) that are associated with reduction in ATPase activity in male-sterile lines (e.g. [68, 69]). Considering this, both mitochondrial *ATP8* and bacterial subunit b hits for M*-ORF* protein sequences may indicate a mitochondrial localization for M-*ORF* in the F0 subunit of complex V, the region of ATP synthase where protons pass through the inner membrane from the intermembrane space to the matrix. Examples of mtDNA-encoded non-canonical subunits of the F0 complex are already known from studies on protists [70] and plants [68, 69], and unionoid M-*ORF*s might be a metazoan version of this scenario. Questions for future studies include whether (1) the M-*ORF* in these species forms part of complex V thereby altering mitochondrial membrane potential, and (2) whether sperm mitochondrial inheritance could be effected by such a mechanism (as proposed by [71]).

Individual F*-ORF* sequences also returned many hits pointing to mitochondrial membrane proteins, often *NAD2*, although with relatively low E-values. Nonetheless, this is interesting because *nad2* and the F*-orf* genes are also typically localized beside one another in a region corresponding to the only gene order rearrangement observed among F mtDNAs in freshwater mussels with DUI [15]. It is plausible that this region was duplicated with subsequent adaptation of one of the two copies of *nad2*. The *nad2* gene is also localized beside the F*-orf* gene in the marine clam *Ruditapes philippinarum* [66] (but this is not the case for all species with DUI). Finally, and not surprisingly, all H*-ORF* sequences returned hits to F*-ORF* sequences (Table 7), and many hits for F-*ORF* profiles are annotated H-*ORF*s, supporting previous results that H-*orf* genes are derived from F*-orf* genes [16]. With a rapid rate of evolution, the mitochondrial ORFans would have rapidly lost their resemblance to the highly conserved mitochondrial genes from which they evolved. Our results do not refute the hypothesis that these ORFans originated from viral sequences, but they open up the possibility of a mitochondrial origin for these genes, specifically *ATP8* and *NAD2* for the M-*ORF* and F-*ORF* in unionoids, respectively.

### Predicted functions for freshwater mussel mitochondrial ORFans

The absolute linkage of a hermaphroditic breeding system, the absence of an M genome and highly modified F-*ORF*s (i.e. H-*ORF*s in hermaphrodites) has led to the hypothesis that the F-*ORF* and M-*ORF* proteins likely have coordinated roles in maintaining gonochorism in freshwater mussels [16]. Furthermore, these roles must be concordantly modified to produce a hermaphroditic individual [16]. Milani et al. [18, 19] suggested that the M-*ORF* protein might play a role in aggregating sperm-derived mitochondria in early-stage male embryos. Our analysis of M*-ORF* sequences indicated connections with cytoskeleton proteins involved in microtubule-binding and actin-binding (e.g. ankyrin). With their predicted SPs and TM helices, M*-ORF*s may target sites outside sperm mitochondria and be responsible for their cellular positioning in developing embryos. It has been suggested that mitochondrial dynamics (e.g., motility, fusion, etc.) must include “signalling” from the respective individual mitochondrion [72]. Although no protein of the dynamics machinery has been identified in bivalves yet, the mtDNA-encoded M*-ORF* in bivalves with DUI is an ideal candidate for direct control of sperm mitochondria. As hypothesized by Milani et al. [18], the M-*ORF* protein could be a masculinizing factor and sperm from males with high amounts of transcript and/or protein would shift embryo development toward maleness. Yusa et al. [73], in their DUI sex-determination model, predicted the existence of such secondary or minor sex-determining mitochondrial factors. Like the M*-ORF*, if the F*-ORF* is a feminizing factor, and because macromutational modifications to the F-*orf* gene are always associated with hermaphroditism, it is tempting to speculate that the F*-ORF* protein could participate in the inhibition of testicular development in embryos that will become females, and the extreme modifications seen in H*-ORF*s could explain why development of some testicular tissue is not completely inhibited in hermaphrodites.

## Conclusions

Because the evolutionary distance among mytilids, venerids, and unionids did not allow for a meaningful comparison of mitochondrial ORFans [18], we decided to perform *in silico* analyses on more closely related ORFan sequences within the order Unionoida. Our findings, in agreement with previous data by Milani et al. [18, 19], reveal high levels of sequence divergence among ORFans, yet with conserved predicted structures, motifs and domains. These ORFans might have originated either from viral horizontal gene transfers or mitochondrial gene duplications but they have evolved rapidly to the point that a clear signature of their origin is not easily recognizable. Our study, which also strongly supports a role for these ORFans in the DUI mechanism, is in line with the growing body of literature extending our understanding of metazoan mitochondrial genome function beyond exclusively OXPHOS related roles (e.g. [18, 59, 74, 75]. DUI as well as other intriguing systems like the recently discovered maternally transmitted sex distortion in booklice that is associated with extremely divergent mitochondria [76], represent interesting cases to look for and better understand antagonistic interactions between distorting mitochondria and nuclear suppressors similar to CMS in plants. If the F*-ORF* and M*-ORF* proteins in bivalves with DUI are indeed antagonistic molecules, i.e. with the F*-ORF* participating in the inhibition of testicular development in female developing embryos and the M*-ORF* participating in the inhibition of ovarian development in male developing embryos, this could explain why macromutations in the F*-ORF* protein (that turns it into a H*-ORF*) would allow for testis development in otherwise female gonads (i.e. hermaphroditism). However, the precise mechanisms underlying DUI and sex determination in bivalves remain to be elucidated.

## Abbreviations

ATP, adenosine triphosphate; *atp8,* ATP synthase subunit 8; CMS, cytoplasmic male sterility; *cox1,* cytochrome c oxidase subunit 1; CTERM, C-terminal; DNA, deoxyribonucleic acid; *dnaB,* DNA helicase; DUI, doubly uniparental inheritance; ER, endoplasmic reticulum; HMM, hidden Markov model; mtDNA, mitochondrial DNA; MY, million years; *nad2,* NADH dehydrogenase subunit 2; *nad5,* NADH dehydrogenase subunit 5; NADH, nicotiamide adenine dinucleotide, reduced form; NCBI, national Center for Biotechnology Information; NRDB, non-redundant protein database; ORF, open reading frame; ORFan, open reading frame without homology to a known protein; PPR, pentatricopeptide repeat; RNA, ribonucleic Acid; SMI, strict Maternal Inheritance; SP, signal peptide; *tatC,* twin-arginine translocase, subunit C; TM, transmembrane; TPR, tetratricopeptide repeat

## Additional files

Additional file 1: Tables SI-S37. Results of *in silico* analyses. (DOCX 368 kb) Additional file 2: Figure S1. Alignments of F*-ORF*s and H*-ORF*s of closely related species. Colour coding is applied to amino acid groups conserved in ≥70 % of sequences. Grey, aliphatic amino acids; orange, aromatic amino acids; yellow, sulfur amino acids; green, amino acids bearing a hydroxyl group; red, basic amino acids; blue, acidic amino acids; brown, amino acids with an amide group; pink, cyclic amino acids. Green box: conserved C-terminal domain identified in [16]; blue underlining: repetitive sequences. UpeFORF, *U. peninsularis* F*-ORF*; UimHORF, *U. imbecillis* H*-ORF*; TliFORF, *T. lividus* F*-ORF*; TpaHORF, *T. parvum* H*-ORF*; MmaFORF, *M. margaritifera* F*-ORF*; MfaHORF, *M. falcata* H*-ORF*; LcoFORF, *L. complanata* F*-ORF*; LcoHORF, *L. compressa* H*-ORF*; LsuHORF, *L. subviridus* H*-ORF*. (PDF 1185 kb) Additional file 3: Figure S2. Percentage of similarity between complete mitochondrial genomes of freshwater mussels with DUI. Each graph shows the percent of conservation between genomes at any given coordinate. The top and bottom percentage bounds are shown to the right of every row. The pink regions are conserved non-protein-coding sequences, the dark blue regions are protein-coding genes, the white regions are non-coding sequences. (A) M vs. M genome comparison between two closely related species (*Utterbackia peninsularis* and *Pyganodon grandis*, GenBank accession numbers HM856635 and FJ809754, respectively) showing that the M*-ORF* gene shows low level of sequence conservation compared to other protein-coding genes. (B) F vs. F genome comparison between two closely related species (*U. peninsularis* and *P. grandis*, GenBank accession numbers HM856636 and FJ809755, respectively) showing that the F*-ORF* gene shows low level of sequence conservation compared to other protein-coding genes. (C) F vs. H genome comparison between two closely related species (*Utterbackia peninsularis* and *U. imbecillis*, GenBank accession numbers HM856636 and HM856637, respectively) showing that the F*-ORF*/H*-ORF* gene region shows low level of sequence conservation compared to other protein-coding genes. (PDF 160 kb) Additional file 4: Figure S3. Protein sequence alignment of *Cumberlandia monodonta* M*-ORF* and *ATP8*, along with *ATP8* from the most diverse members of the Mt_ATP-synt_B superfamily (pfam02326). *Homo sapiens ATP8* has also been included for comparison. The alignment was generated using T-COFFEE. The most conserved N-terminal domain, i.e. the best aligned portion, is in red; the rest of the sequences are rather badly aligned (in green). Consensus is shown and indicates good (red), intermediate (yellow), and bad alignment (green), and insertion/deletion (in blue). Cumberland, *Cumberlandia monodonta*; H_sapiens, *Homo sapiens*; Malawimonas, *Malawimonas* sp. (Excavate); Thraustoch, *Thraustochytrium* sp. (Stramenopiles); Mesostigma, *Mesostigma* sp. (Streptophyta); Reclinomon, *Reclinomonas* sp. (Protozoa); Porphyra, *Porphyra* sp. (Rhodophyta); Cyanidiosc, *Cyanidioschyzon* sp. (Rhodophyta); Pseudendoc, *Pseudendoclonium* sp. (Chlorophyta); Acanthamoe, *Acanthamoeba* sp. (Amoebozoa); Nephroselm, *Nephroselmis* sp. (Streptophyta). (PDF 236 kb) Additional file 5: Figure S4. Protein sequence alignment of *Cumberlandia monodonta* F*-ORF* and *NAD2*. The alignment was generated using T-COFFEE. Consensus is shown and indicates identical (*) and similar (: and.) amino acids. Description of the data: Protein sequence alignment of *Cumberlandia monodonta* F*-ORF* and *NAD2*. (PDF 141 kb) Additional file 6: Figure S5. Position of frequently recurring functions in HHpred and BLAST hits for (a) M*-ORF*s, (b) F*-ORF*s, and (c) and (d) H*-ORF*s. Hits with positions were grouped into categories and traced together, showing hot spots of functionality. Protein length in amino acids is indicated in parentheses. (PDF 301 kb)
