# Supplementary material for: In silico analyses of mitochondrial ORFans in freshwater mussels (Bivalvia: Unionoida) provide a framework for future studies of their origin and function
Source: BMC Genomics. 2016 Aug 9;17:597. doi: 10.1186/s12864-016-2986-6 (PMC4979158; doi:10.1186/s12864-016-2986-6)
Supplement: Additional file 1: Tables SI-S37. — Results of in silico analyses. (DOCX 368 kb) [file 12864_2016_2986_MOESM1_ESM.docx]

Table S1. Information on ORFan sequences used in this study.

| **Family** | **Subfamily** | **Species** | **mtDNA**  **type** | **Accession**  **number** | **Complete**  **genome** | **ORF name** | **Start** | **End** | **Length**  **nt** | **Start**  **codon** | **Stop**  **codon** | **Length**  **aa** |
| --- | --- | --- | --- | --- | --- | --- | --- | --- | --- | --- | --- | --- |
| Hyriidae |  | *Hyridella menziesii* | F | KU728092 | no | Hme-Forf | 1 | 279 | 279 | GTG | TAG | 92 |
|  |  |  | M | KU728093 | no | Hme-Morf | 1 | 948 | 948 | ATG | TAG | 315 |
| Margaritiferidae | Margaritiferinae | *Cumberlandia monodonta* | F | HM849375.1 | no | Cmo-Forf | 1 | 276 | 276 | ATT | TAA | 91 |
|  |  |  | M | KU728095 | no | Cmo-Morf | 1 | 291 | 291 | ATG | TAG | 96 |
|  |  | *Margaritifera falcata* | H | HM849545.1 | no | Mfa-Horf-1 | 1 | 381 | 381 | ATA | TAA | 126 |
|  |  |  |  | HM856634.1 | yes | Mfa-Horf-2 | 3151 | 3531 | 381 | ATA | TAA | 126 |
|  |  |  |  | HM849547.1 | no | Mfa-Horf-3 | 1 | 414 | 414 | ATA | TAA | 137 |
|  |  |  |  | HM849548.1 | no | Mfa-Horf-4 | 1 | 381 | 381 | ATA | TAA | 126 |
|  |  | *Margaritifera margaritifera* | F | HM849399.1 | no | Mma-Forf | 1 | 333 | 333 | ATT | TAG | 110 |
| Unionidae | Ambleminae | *Quadrula quadrula* | F | FJ809750.1 | yes | Qqu-Forf | 3303 | 3545 | 243 | ATA | TAA | 80 |
|  |  |  | M | FJ809751.1 | yes | Qqu-Morf | 14965 | 14648 | 318 | GTG | TAG | 105 |
|  |  | *Toxolasma lividus* | F | HM849457.1 | no | Tli-Forf | 1 | 342 | 342 | GTG | TAA | 113 |
|  |  | *Toxolasma parvum* | H | KU728097 | no | Tpa-Horf | 1 | 609 | 609 | ATT | TAA | 202 |
|  |  | *Venustaconcha ellipsiformis* | F | FJ809753.1 | yes | Vel-Forf | 3233 | 3502 | 270 | TTG | TAA | 89 |
|  |  |  | M | FJ809752.1 | yes | Vel-Morf | 15279 | 14599 | 681 | ATC | TAA | 226 |
|  | Anodontinae | *Anodonta anatina* | F | KF030964.1 | yes | Aan-Forf | 8428 | 8664 | 237 | ATT | TAA | 78 |
|  |  |  | M | KF030962.1 | yes | Aan-Morf | 4483 | 3896 | 588 | ATA | TAA | 195 |
|  | Gonideinae | *Inversidens japanensis* | F | AB055625.1 | no | Ija-Forf | 6556 | 6356 | 201 | ATT | TAA | 66 |
|  |  |  | M | AB055624.1 | no | Ija-Morf | 1826 | 2182 | 357 | ATG | TAA | 118 |
|  |  | *Solenaia carinatus* | F | KC848654.1 | yes | Sca-Forf | 4403 | 4663 | 261 | ATA | TAA | 86 |
|  |  |  | M | KC848655.1 | yes | Sca-Morf | 15266 | 14832 | 435 | ATA | TAA | 144 |
|  | Unioninae | *Lasmigona complanata* | F | HM849393.1 | no | Lco-Forf | 1 | 234 | 234 | ATC | TAA | 77 |
|  |  | *Lasmigona compressa* | H | HM849534.1 | no | Lco-Horf-1 | 1 | 624 | 624 | ATC | TAA | 207 |
|  |  |  |  | HM849535.1 | no | Lco-Horf-2 | 1 | 585 | 585 | ATA | TAA | 194 |
|  |  | *Lasmigona subviridis* | H | HM849542.1 | no | Lsu-Horf-1 | 1 | 594 | 594 | TTG | TAA | 197 |
| Unionidae | Unioninae | *Lasmigona subviridis* | H | HM849543.1 | no | Lsu-Horf-2 | 1 | 690 | 690 | ATG | na | 229 |
|  |  | *Pyganodon grandis* | F | FJ809754.1 | yes | Pgr-Forf | 3087 | 3344 | 258 | ATA | TAA | 85 |
| Unionidae | Unioninae | *Pyganodon grandis* | M | FJ809755.1 | yes | Pgr-Morf | 15226 | 14522 | 705 | TTG | TAA | 234 |
|  |  | *Utterbackia imbecillis* | H | HM849591.1 | no | Uim-Horf-1 | 1 | 729 | 729 | ATG | TAA | 242 |
|  |  |  |  | HM849595.1 | no | Uim-Horf-2 | 1 | 849 | 849 | ATC | TAA | 282 |
|  |  |  |  | HM849594.1 | no | Uim-Horf-3 | 1 | 1113 | 1113 | ATC | TAA | 370 |
|  |  |  |  | HM849601.1 | no | Uim-Horf-4 | 1 | 789 | 789 | ATC | TAA | 262 |
|  |  |  |  | HM849606.1 | no | Uim-Horf-5 | 1 | 921 | 921 | ATC | TAA | 306 |
|  |  |  |  | HM849597.1 | no | Uim-Horf-6 | 1 | 849 | 849 | ATC | TAA | 282 |
|  |  |  |  | HM849584.1 | no | Uim-Horf-7 | 1 | 1017 | 1017 | ATC | TAA | 338 |
|  |  | *Utterbackia peninsularis* | F | HM856636.1 | yes | Upe-Forf | 3125 | 3337 | 213 | ATT | TAA | 70 |
|  |  |  | M | HM856635.1 | yes | Upe-Morf | 14942 | 14286 | 657 | ATA | TAA | 218 |

NOTE. – M = M mtDNA in a DUI gonochoric breeding system, F = F mtDNA in a DUI gonochoric breeding system, H = H mtDNA in a non-DUI hermaphroditic breeding system. For each GenBank accession number, it is specified if the sequence is a complete mt genome or not. Stop codon is not available for *Lasmigona subviridis* Lsu-Horf-2 as this sequence is truncated in its 3’ end.

Table S2. Predicted transmembrane (TM) helices in M*-ORFs* and F*-ORFs.*

| **TM Helices** | | | | | | | | | | | | |
| --- | --- | --- | --- | --- | --- | --- | --- | --- | --- | --- | --- | --- |
|  | *Aan* | *Upe* | *Pgr* | *Lco* | *Ija* | *Sca* | *Tli* | *Vel* | *Qqu* | *Mma* | *Cmo* | *Hme* |
| **M*-ORF*** |  | | | | | | | | | | | |
| Phobius | 20-44 | 20-45 | 20-46 |  | *21-41* | 23-41 |  | 20-38 | 6-34 |  | 20-37 | *20-42,*  54-77,  *89-109* |
| InterProScan (TMHMM) | 24-46 | 20-42 | 22-44 |  | 21-43 | 21-43 |  | 20-42 | 5-27 |  | 15-37 | 13-35,  55-77,  90-109 |
| TMPred | 23-41 | 21-38 | *24-45* |  | *24-41* | *23-40* |  | *21-39* | 7-27 |  | *16-34* | *20-36*  54-73  *90-112* |
| TOPCONS | *24-44* | *18-38* | *22-42* |  | 25-45 | 24-44 |  | *15-35* | 17-37 |  | 17-37 | 2-22,  *69-89* |
| Predict Protein | 26-43 | 22-39 | 24-44 |  | 19-42 | 22-39 |  | 21-38 | 17-32 |  | 17-33 | 21-38 |
| Consensus | ~23-44 | ~20-38 | ~22-44 |  | ~24-42 | ~22-41 |  | ~20-38 | ~10-30 |  | ~17-35 | ~19-34, 54-72,  90-110 |
| **F*-ORF*** |  | | | | | | | | | | | |
| Phobius | - | - | - | - | - | - | *45-65* | *21-42* | *12-30* | 31-53 | - | - |
| InterProScan (TMHMM) | 9-31 | 7-29 | 16-38 | 5-27 | - | 7-26 | 45-67 | 21-43 | 12-24 | 31-53 | - | 15-37 |
| TMPred | *9-27* | *6-25* | *16-40* | *8-26* | *1-18* | *7-23* | 45-68 | *21-42* | 12-30 | *32-49* | *2-18* | *18-37* |
| TOPCONS | *9-29* | *8-28* | *16-36* | *8-28* | *2-22* | *6-26* | *41-61* | *21-41* | *10-30* | *31-51* | 2-22 | *17-37* |
| Predict Protein | 9-26 | 8-25 | 14-31 | 8-25 | 1-18 | 8-25 | 44-66 | 20-42 | 16-33 | 32-49 | 1-18 | 17-31 |
| Consensus | *~9-28* | *~7-27* | *~16-35* | *~8-26* | *~1-19* | *~7-25* | ~45-66 | *~21-42* | ~12-29 | ~31-51 | ~2-19 | *~17-36* |

Note. – All structures listed here were statistically supported by the programs used (Phobius posterior label probability>0.5; PrediSi score>0.5; SignalP score>D-cutoff 0.5; TMpred score>500; significance test not provided by the other programs). Numbers in italics represent TMHs predicted to be oriented from inside to outside, those underlined represent TMHs predicted to be oriented from outside to inside.

Table S3. Predicted signal peptides in M*-ORFs* and F*-ORFs.*

| **Signal Peptides** | | | | | | | | | | | | |
| --- | --- | --- | --- | --- | --- | --- | --- | --- | --- | --- | --- | --- |
|  | *Aan* | *Upe* | *Pgr* | *Lco* | *Ija* | *Sca* | *Tli* | *Vel* | *Qqu* | *Mma* | *Cmo* | *Hme* |
| **M*-ORF*** |  | | | | | | | | | | | |
| Phobius | - | - | - |  | - | - |  | - | - |  | - | - |
| InterProScan |  | - | - |  | - | - |  | - | - |  | - | - |
| PrediSi | CP43 | CP 42* | CP 44 |  | CP 40* | CP 35 |  | CP 40* | CP 29* |  | CP 34 | CP38* |
| SignalP | 1-20 | 1-10 | 1-10 |  | 1-40 | 1-16 |  | 1-40 | 1-10 |  | 1-10 | 1-37 |
| Consensus | - | - | - |  | 1-40 | - |  | 1-40 | - |  | - | 1-38 |
| **F*-ORF*** |  | | | | | | | | | | |  |
| Phobius | 1-26* | 1-25* | 1-33* | 1-37* | 1-26* | 1-32* | - | - | - | - | 1-20* | 1-40* |
| InterProScan | - | - | - | - | - | - | - | - | - | - | - | - |
| PrediSi | CP26* | CP 25* | CP 33* | CP 25* | CP 17* | CP 32* | CP67 | CP44 | CP 32* | CP 51 | CP 20* | CP 40* |
| SignalP | 1-26* | 1-19 | 1-36 | 1-37 | 1-20* | 1-32* | 1-18 | 1-44 | 1-32 | 1-51 | 1-20* | 1-40 |
| Consensus | 1-26 | ~1-25 | 1-34 | ~1-33 | ~1-23 | 1-32 | - | 1-44 | 1-32 | 1-51 | 1-20 | 1-40 |

Note. – All structures marked by an asterisk were statistically supported by the programs used. Those not marked with an asterisk were not statistically supported, but were predicted by multiple programs. (Phobius posterior label probability>0.5; PrediSi score>0.5; SignalP score>D-cutoff 0.5; TMpred score>500; significance test not provided by the other programs).

Table S4. Predicted transmembrane (TM) helices in H*-ORFs.*

| **TM Helix** | | | | | | | | | | | | | | |
| --- | --- | --- | --- | --- | --- | --- | --- | --- | --- | --- | --- | --- | --- | --- |
|  | *Uim1* | *Uim2* | *Uim3* | *Uim4* | *Uim5&6* | *Uim7* | *Lsu1* | *Lsu2* | *Lco1* | *Lco2* | *Tpa* | *Mfa1* | *Mfa2&4* | *Mfa3* |
| **H*-ORF*** |  | | | | | | | | | | | | | |
| Phobius | *21-46,*  52-73, *149-170,*  190-209 | *37-61,*  67-84 | *37-61,*  67-84 | *44-68,*  74-95 | *40-61,*  67-84 | *44-68,*  74-94 | *12-36* | - | - | *7-31* | - | - | - | - |
| InterProScan (TMHMM) | 17-39 | 39-61 | 39-61 | 39-61 | 39-61 | 39-61 | 12-34 | 7-29 | - | 7-29 | 22-44 | 44-61 | 44-61 | 44-61 |
| TMPred | 23-50,  *153-171* | 54-72 | 54-72 | *45-72* | *44-72* | *45-72* | *14-32* | *8-26* | *2-20* | *7-31* | *22-42* | *44-62* | *44-62* | *44-62* |
| TOPCONS | *150-170,*  189-209 | *29-49* | - | 52-72 | *59-79* | *20-40,*  42-62 | - | - | - | - | *22-42* | *44-64* | *44-64* | *44-64* |
| Predict Protein | 22-41,  46-63,  195-212 | 41-65 | 43-67 | 46-64 | 51-65 | 42-66 | 16-33 | 11-29 | 1-18 | 10-28 | 26-44 | 43-61 | 43-61 | 43-60 |
| Consensus | ~22-46 | ~40-62 | ~44-65 | ~45-68 | ~45-65 | ~42-64 | ~13-33 | ~9-28 | - | ~7-30 | ~22-43 | ~44-62 | ~44-62 | ~44-62 |

Note. – All structures listed here were statistically supported by the programs used (Phobius posterior label probability>0.5; PrediSi score>0.5; SignalP score>D-cutoff 0.5; TMpred score>500; significance test not provided by the other programs). Numbers in italics represent TMHs predicted to be oriented from inside to outside, those underlined represent TMHs predicted to be oriented from outside to inside.

Table S5. Predicted signal peptides in H*-ORFs.*

| **Signal Peptides** | | | | | | | | | | | | | | |
| --- | --- | --- | --- | --- | --- | --- | --- | --- | --- | --- | --- | --- | --- | --- |
|  | *Uim1* | *Uim2* | *Uim3* | *Uim4* | *Uim5&6* | *Uim7* | *Lsu1* | *Lsu2* | *Lco1* | *Lco2* | *Tpa* | *Mfa1* | *Mfa2&4* | *Mfa3* |
| **H*-ORF*** |  | | | | | | | | | | | | | |
| Phobius | - | - | - | - | - | - | 1-25* | 1-19* | 1-19* | - | 1-47* | 1-61* | 1-61* | 1-61* |
| InterProScan | - | - | - | - | - | - | - | - | - | - | - | - | - | - |
| PrediSi | CP 168* | CP 69 | CP 69 | CP 69 | CP 69 | CP 69 | CP 25* | CP 19* | CP 17 | CP 18 | CP 49* | CP 64* | CP 64* | CP 64* |
| SignalP | 1-15 | 1-24 | 1-24 | 1-24 | 1-24 | 1-24 | 1-10 | 1-19 | 1-17 | 1-10 | 1-48 | 1-29 | 1-29 | 1-29 |
| Consensus | - | - | - | - | - | - | 1-25 | 1-19 | 1-18 | - | ~1-49 | 1-62 | 1-62 | 1-62 |

Note. – All structures marked by an asterisk were statistically supported by the programs used. Those not marked with an asterisk were not statistically supported, but were predicted by multiple programs. (Phobius posterior label probability>0.5; PrediSi score>0.5; SignalP score>D-cutoff 0.5; TMpred score>500; significance test not provided by the other programs).

Table S6. Frequently recurring HHpred hits in F*-ORFs* and M*-ORFs*

|  | ***Aan*** | ***Upe*** | ***Pgr*** | ***Lco*** | ***Ija*** | ***Sca*** | ***Tli*** | ***Vel*** | ***Qqu*** | ***Mma*** | ***Cmo*** | ***Hme*** |
| --- | --- | --- | --- | --- | --- | --- | --- | --- | --- | --- | --- | --- |
| **F*-ORF* – probability (rank)** | | | | | | | | | | | | |
| Prepilin-type processing-associated H-X9-DG domain | 99.31 (2) | 99.34 (2) | 99.27 (3) | 99.32 (2) | 99.23 (3) | 99.37 (1) | 99.23 (1) | 99.30 (1) | 99.37 (1) | 99.11 (2) | 99.04 (2) | 99.25 (1) |
| Outer membrane insertion C-terminal signal | 99.24 (3) | 99.28 (3) | 99.34 (2) | 99.27 (3) | 99.36 (2) | 99.06 (2) | 99.14  (2) | 99.16 (2) | 99.21 (2) | 99.14 (1) | 99.21 (1) | 99.05 (3) |
| LPXTG cell wall anchor domain | 99.47 (1) | 99.47 (1) | 99.45 (1) | 99.46 (1) | 99.44 (1) | 98.91 (3) | 98.81 (3) | 98.97 (3) | 99.02 (3) | 98.87 (3) | 98.83 (3) | 99.10 (2) |
| X-X-X-Leu-X-X-Gly heptad repeats | 98.03 (4) | 98.08 (4) | 97.97 (4) | 98.05 (4) | 97.91 (4) | 97.69 (4) | 97.99 (4) | 97.97 (4) | 97.98 (4) | 97.75 (4) | 97.70 (4) | 97.66 (4) |
| GlyGly-CTERM domain | 97.33 (5) | 97.39 (5) | 97.22 (5) | 97.32 (5) | 97.58 (5) | 96.90 (5) | 97.08 (5) | 97.29 (5) | 97.38 (5) | 97.15 (5) | 96.86 (5) | 96.93 (5) |
| Pentatricopeptide repeat domain | 94.32 (6) | 94.79 (6) | 94.27 (7) | 94.58 (6) | 94.24 (6) | 93.28 (6) | 94.54  (6) | 95.33 (6) | 94.93 (6) | 93.83 (6) | 93.47 (6) | 93.52 (6) |
| **M*-ORF* – probability (rank)** | | | | | | | | | | | | |
| Prepilin-type processing-associated H-X9-DG domain | 99.04 (2) | 99.06 (2) | 99.10 (1) |  | 99.21 (2) | 99.15 (1) |  | 99.01 (2) | 99.14 (2) |  | 99.52 (1) | 99.58 (1) |
| Outer membrane insertion C-terminal signal | 99.24  (1) | 96.16 (1) | 98.89 (3) |  | 99.25 (1) | 98.75 (3) |  | 99.11 (1) | 99.19 (1) |  | 99.20 (2) | 99.19 (2) |
| LPXTG cell wall anchor domain | 98.89 (3) | 98.89 (3) | 99.05 (2) |  | 98.80 (3) | 98.88 (2) |  | 98.99 (3) | 99.12 (3) |  | 98.77 (3) | 98.67 (3) |
| X-X-X-Leu-X-X-Gly heptad repeats | 97.70 (4) | 97.32 (4) | 97.48 (4) |  | 97.73 (4) | 97.95 (4) |  | 97.60 (4) | 97.91 (4) |  | 97.81 (4) | 97.91 (4) |
| GlyGly-CTERM domain | 97.26 (5) | 97.24 (5) | 97.15 (5) |  | 96.74 (5) | 97.04 (5) |  | 96.47 (14) | 97.57 (5) |  | 97.14 (5) | 96.67 (8) |
| Pentatricopeptide repeat domain | 92.98 (6) | 92.61 (6) | 92.96 (6) |  | 94.79 (6) | 94.60 (6) |  | 92.71 (48) | 93.60 (6) |  | 94.35 (6) | 93.94 (47) |
| **F*-ORF* – amino acid position** | | | | | | | | | | | | |
| Prepilin-type processing-associated H-X9-DG domain | 19-22 | 18-21 | 26-29 | 18-29 | 13-15 | 2-9 | 34-36 | 11-13 | 1-4 | 44-49 | 48-50 | 80-85 |
| Outer membrane insertion C-terminal signal | 35-36 | 27-28 | 1-6 | 34-35 | 3-5 | 62-63 | 1-8 | 25-29 | 16-20 | 23-24 | 12-14 | 1-6 |
| LPXTG cell wall anchor domain | 55-60 | 47-52 | 62-67 | 54-59 | 1-15 | 4-22 | 95-96 | 19-34 | 10-25 | 32-48 | 72-76 | 8-35 |
| X-X-X-Leu-X-X-Gly heptad repeats | 47-54 | 39-46 | 54-61 | 46-53 | 57-65 | 4-7 | 18-22 | 71-78 | 62-69 | 49-56 | 18-27 | 8-12 |
| GlyGly-CTERM domain | 9-19 | 8-18 | 16-26 | 8-18 | 2-13 | 7-17 | 50-60 | 28-35 | 19-26 | 36-48 | 4-15 | 23-35 |
| Pentatricopeptide repeat domain | 26-46 | 31-38 | 46-53 | 38-45 | 14-18 | 16-23 | 30-49 | 7-25 | 63-66 | 16-23 | 59-68 | 60-69 |
| **M*-ORF* – amino acid position** | | | | | | | | | | | | |
| Prepilin-type processing-associated H-X9-DG domain | 29-32 | 41-44 | 40-46 |  | 28-31 | 27-30 |  | 26-29 | 70-71 |  | 30-34 | 107-111 |
| Outer membrane insertion C-terminal signal | 57-64 | 53-60 | 55-59 |  | 40-44 | 6-7 |  | 158-164 | 19-21 |  | 51-56 | 53-56 |
| LPXTG cell wall anchor domain | 22-42 | 18-38 | 20-40 |  | 103-107 | 80-85 |  | 17-39 | 8-28 |  | 14-35 | 93-109 |
| X-X-X-Leu-X-X-Gly heptad repeats | 102-108 | 107-121 | 144-149 |  | 46-64 | 60-67 |  | 40-46 | 69-72 |  | 13-16 | 123-137 |
| GlyGly-CTERM domain | 30-42 | 26-38 | 28-40 |  | 22-35 | 21-36 |  | 22-35 | 6-18 |  | 16-26 | 98-109 |
| Pentatricopeptide repeat domain | 102-125 | 32-39 | 29-41 |  | 1-14 | 3-13 |  | 120-136 | 48-60 |  | 39-51 | 71-90 |

Table S7. Frequently recurring HHpred hits in H*-ORFs*

|  | ***Uim1*** | ***Uim2*** | ***Uim3*** | ***Uim4*** | ***Uim5&6*** | ***Uim7*** | ***Lsu1*** | ***Lsu2*** | ***Lco1*** | ***Lco2*** | ***Tpa*** | ***Mfa1*** | ***Mf2&4*** | ***Mfa3*** |
| --- | --- | --- | --- | --- | --- | --- | --- | --- | --- | --- | --- | --- | --- | --- |
| **H*-ORF* – probability (rank)** | | | | | | | | | | | | | | |
| Prepilin-type processing-associated H-X9-DG domain | 99.30 (1) | 99.28 (1) | 99.27 (2) | 99.14 (2) | 99.14 (2) | 99.16 (2) | 99.14 (2) | 99.07 (1) | 99.15 (1) | 99.17 (1) | 98.98 (2) | 99.16 (2) | 99.16 (2) | 99.14 (2) |
| Outer membrane insertion C-terminal signal | 99.27 (2) | 99.22 (2) | 99.33 (1) | 99.28 (1) | 99.19 (1) | 99.28 (1) | 99.24 (1) | 98.86 (3) | 98.77 (3) | 98.82 (3) | 98.46 (3) | 99.23 (1) | 99.23 (1) | 99.19 (1) |
| LPXTG cell wall anchor domain | 98.89 (3) | 98.94 (3) | 98.98 (3) | 98.89 (3) | 98.86 (3) | 98.89 (3) | 99.03 (3) | 98.98 (2) | 98.90 (2) | 99.09 (2) | 99.01 (1) | 98.88 (3) | 98.93 (3) | 98.94 (3) |
| X-X-X-Leu-X-X-Gly heptad repeats | 97.49 (4) | 97.63 (7) | 97.70 (21) | 97.50 (11) | 97.45 (6) | 97.53 (4) | 97.61 (4) | 97.52 (4) | 97.64 (4) | 97.62 (4) | 97.44 (4) | 97.66 (4) | 97.50 (4) | 97.43 (4) |
| GlyGly-CTERM domain | 96.92 (7) | 97.09 (19) | 97.05 (45) | 96.95 (15) | 96.72 (7) | 96.97 (5) | 96.98 (5) | 96.88 (5) | 96.49 (5) | 97.02 (5) | 96.69 (5) | 96.99 (5) | 96.99 (5) | 96.95 (5) |
| Pentatricopeptide repeat domain | 94.13 (23) | - | - | 94.09 (30) | 94.26 (8) | 94.00 (8) | 92.73 (8) | 92.04 (8) | 93.03 (6) | 92.77 (8) | 93.27 (6) | 93.25 (6) | 93.25 (6) | 93.37 (6) |
| **H*-ORF* – amino acid position** | | | | | | | | | | | | | | |
| Prepilin-type processing-associated H-X9-DG domain | 201-204 | 45-50 | 25-30 | 25-30 | 13-15 | 13-15 | 34-37 | 28-31 | 17-20 | 27-30 | 134-135 | 5-8 | 5-8 | 5-8 |
| Outer membrane insertion C-terminal signal | 49-52 | 75-81 | 71-74 | 71-74 | 71-74 | 71-74 | 1-5 | 91-93 | 23-24 | 33-34 | 40-44 | 5-7 | 5-7 | 5-7 |
| LPXTG cell wall anchor domain | 75-77 | 56-72 | 49-64 | 49-64 | 97-99 | 49-64 | 14-30 | 8-24 | 2-13 | 7-23 | 24-41 | 43-59 | 43-60 | 43-60 |
| X-X-X-Leu-X-X-Gly heptad repeats | 21-23 | 227-231 | 43-45 | 43-45 | 43-45 | 43-45 | 95-101 | 91-95 | 79-92 | 112-125 | 189-194 | 60-67 | 61-67 | 61-67 |
| GlyGly-CTERM domain | 49-57 | 58-71 | 71-79 | 71-79 | 55-70 | 96-97 | 14-24 | 8-18 | 2-12 | 7-17 | 33-44 | 47-59 | 47-59 | 47-60 |
| Pentatricopeptide repeat domain | 14-21 | - | - | 36-43 | 48-57 | 87-98 | 31-35 | 25-45 | 14-34 | 22-44 | 17-23 | 23-27 | 23-27 | 23-27 |

Table S8. Hits to other motifs and domains in M*-ORFs* and F*-ORFs*

| **Motif or domain** | | ***Aan*** | ***Upe*** | ***Pgr*** | ***Ija*** | ***Sca*** | ***Tli*** | ***Vel*** | ***Qqu*** | ***Cmo*** | ***Hme*** |
| --- | --- | --- | --- | --- | --- | --- | --- | --- | --- | --- | --- |
| Lysine-rich region profile | M  F | X | X | X | X | X |  | X |  | X | X |
| Bipartite nuclear localization signal profile | M  F |  | X | X |  |  |  |  |  |  | X |
| RNA recognition motif in regulators of calcineurin and similar proteins | M  F |  | X |  |  |  |  |  |  |  |  |
| Prokaryotic membrane lipoprotein lipid attachment site profile | M  F |  |  |  |  |  |  |  |  | X |  |
| HIG1 domain family member | M  F | X | X |  |  |  |  |  |  |  | X |
| Telomerase reverse transcriptase (TEN domain) | M  F | X |  |  |  |  |  |  |  |  |  |
| EGF-like-domain | M  F | X | X | X |  |  |  |  |  |  | X |
| Voltage-dependent anion channel | M  F |  |  |  |  |  |  |  |  |  | X |
| Histone H1-like protein Hc1 | M  F |  |  |  |  |  |  |  |  |  | X |
| Microtubule-binding protein MIP-T3 | M  F |  |  |  |  |  |  |  |  |  | X |
| Periplasmic protein TonB, links inner and outer membranes | M  F |  |  |  |  |  |  |  |  |  | X |
| Cell division protein FtsN | M  F |  |  |  |  |  |  |  |  |  | X |
| Plant ATP synthase F0 | M  F |  |  |  |  |  |  |  |  | X |  |
| DUF4381 Domain of unknown function | M  F |  |  |  |  |  |  |  |  | X |  |
| E set domains | M  F |  |  |  |  |  |  |  |  | X |  |
| Homeodomain-like | M  F |  |  |  |  |  |  |  |  | X |  |
| PELOTA RNA binding domain | M  F |  | X |  |  |  |  |  |  |  |  |
| Trigger factor ribosome-binding domain | M  F |  | X |  |  |  |  |  |  |  |  |
| DNaJ domain family member | M  F |  |  |  | X |  |  |  |  |  |  |
| Autophagy protein Apg6 | M  F |  |  |  |  |  |  | X |  |  |  |
| Chromosome segregation ATPases | M  F |  |  |  |  |  |  | X |  |  |  |
| chromosome segregation protein SMC, common bacterial type | M  F |  |  |  |  |  |  | X |  |  |  |
| TIGR03778 VPDSG-CTERM protein sorting domain | M  F |  |  |  | X |  |  |  |  |  |  |
| Bifunctional 2',3'-cyclic nucleotide 2'-phosphodiesterase/3'-nucleotidase precursor protein | M  F |  |  |  |  | X |  |  |  |  |  |
| TIGR04288 CGP-CTERM domain | M  F |  |  |  |  |  |  |  |  | X |  |
| Homodimeric domain of signal transducing histidine kinase | M  F |  |  |  |  |  |  |  |  | X |  |
| Virus attachment protein globular domain | M  F |  |  |  |  |  |  |  |  | X |  |
| Opacity-associated protein A N-terminal motif | M  F |  |  |  |  |  | X | X | X |  |  |

Note. – *Lco* and *Mma* M*-ORFs* and F*-ORFs* did not return any motifs or domains other than the frequently recurring HHpred hits.

Table S9. Hits to other motifs and domains in H*-ORFs*

| **Motif or domain** | ***Uim*** | | | | | | ***Lsu*** | | ***Mfa*** | | |
| --- | --- | --- | --- | --- | --- | --- | --- | --- | --- | --- | --- |
|  | **1** | **2** | **3** | **4** | **5&6** | **7** | **1** | **2** | **1** | **2&4** | **3** |
| Response regulator receiver domain protein (CheY-like) | X |  |  |  |  |  |  |  |  |  |  |
| Mitochondria Localisation Sequence |  | X | X |  |  |  |  |  |  |  |  |
| ribonuclease E |  | X | X |  |  | X |  |  |  |  |  |
| Ehrlichia tandem repeat |  | X | X |  |  | X |  |  |  |  |  |
| Terminal organelle assembly protein TopJ |  | X | X |  |  |  |  |  |  |  |  |
| Bifunctional 2',3'-cyclic nucleotide 2'-phosphodiesterase/3'-nucleotidase precursor protein |  |  |  | X |  |  |  |  |  |  |  |
| TIGR03544 DivIVA domain |  |  |  |  |  |  |  |  | X | X | X |
| EGF-like-domain |  |  |  |  |  |  |  |  | X |  | X |
| Herpes virus major outer envelope glycoprotein (BLLF1) |  |  |  |  |  |  | X | X |  |  |  |

Note – *Lco* and *Tpa* did not return any motifs or domains other than the frequently recurring HHpred hits.

Table S10. Filtered hmmsearch output for the M-*ORF* and F-*ORF* HMM profiles built using default parameters with hmmbuild.

| *Profile* | *Database* | *Target* | *Description* | *Kingdom* | *Species* | *# hits* | *# significant hits* | *Bit Score* | *E-value* |
| --- | --- | --- | --- | --- | --- | --- | --- | --- | --- |
| M-*ORF* | UniProtKB | F4ZG80_9BIVA | M-specific morf protein | Eukaryota | Utterbackia peninsularis | 1 | 1 | **174.6** | **5.20E-48** |
|  |  | V9PBU4_9BIVA | M-ORF | Eukaryota | Solenaia carinatus | 1 | 1 | **94.8** | **1.70E-23** |
|  |  | A0A023I1E9_ANOAN | M-ORF | Eukaryota | Anodonta anatina | 1 | 1 | **88.8** | **1.20E-21** |
|  |  | A0A023I1I6_ANOAN | M-ORF | Eukaryota | Anodonta anatina | 1 | 1 | **88.6** | **1.40E-21** |
|  |  | A0A0F4GXW8_9PEZI | DUF221-domain-containing protein | Eukaryota | Zymoseptoria brevis | 1 | 1 | **30.5** | **0.001** |
|  |  | G2RQY3_BACME | Excalibur domain protein | Bacteria | Bacillus megaterium WSH-002 | 1 | 1 | **30.3** | 0.0011 |
|  |  | A0A068N778_BACCE | Group-specific protein | Bacteria | Bacillus cereus | 1 | 1 | **29.0** | 0.0029 |
|  |  | A0A0D0GUL4_BACTM | Bacillus thuringiensis serovar morrisoni strain HD 600 BG10.Contig244, whole genome shotgun sequence | Bacteria | Bacillus thuringiensis subsp. morrisoni | 1 | 1 | **28.2** | 0.0048 |
|  |  | G3H659_CRIGR | CKLF-like MARVEL transmembrane domain-containing protein 2B | Eukaryota | Cricetulus griseus | 1 | 1 | **27.4** | 0.0087 |
|  |  | K2G8H3_9BACT | RNA binding S1 protein | Bacteria | uncultured bacterium (gcode 4) | 1 | 1 | **27.4** | 0.0089 |
|  |  | R7N780_9FIRM | Electron transport complex subunit E | Bacteria | Firmicutes bacterium CAG:95 | 1 | 0 | **26.6** | 0.016 |
|  |  | A0A0E0W0K4_BACAN | Group-specific protein | Bacteria | Bacillus anthracis str. H9401 | 1 | 0 | **26.3** | 0.019 |
|  |  | Q63BB7_BACCZ | Group-specific protein | Bacteria | Bacillus cereus (strain ZK / E33L) | 1 | 0 | **26.3** | 0.019 |
|  |  | C1H9F4_PARBA | Nucleolar protein NOP56 | unclassified sequences | unclassified | 1 | 0 | **25.9** | 0.025 |
|  |  | A0A0D6M554_9BILA | SnoRNA binding domain protein | Eukaryota | Ancylostoma ceylanicum | 1 | 0 | **25.6** | 0.031 |
|  |  | A5KSC4_9BACT | ATP synthase subunit b | Bacteria | candidate division TM7 genomosp. GTL1 | 1 | 0 | **24.9** | 0.052 |
|  |  | A0A061B2Y9_CYBFA | CYFA0S08e02300g1_1 | Eukaryota | Cyberlindnera fabianii | 1 | 0 | **24.6** | 0.061 |
|  |  | Q8EWL2_MYCPE | Putative uncharacterized protein MYPE1910 | Bacteria | Mycoplasma penetrans (strain HF-2) | 1 | 0 | **24.4** | 0.076 |
|  |  | A0A098DB54_GIBZA | Fusarium graminearum chromosome 1, complete genome | Eukaryota | Gibberella zeae | 1 | 0 | **24.3** | 0.079 |
|  |  | H2J4N9_MARPK | ATP synthase subunit b | Bacteria | Marinitoga piezophila (strain DSM 14283 / JCM 11233 / KA3) | 1 | 0 | **24.3** | 0.081 |
|  |  | A0A061CBD1_LACDL | Hypothetical membrane protein | Bacteria | Lactobacillus delbrueckii subsp. lactis | 1 | 0 | **23.7** | 0.12 |
| M-*ORF* | UniProtKB | H2B2B1_KAZAF | KAFR0L01510 protein | Eukaryota | Kazachstania africana (strain ATCC 22294 / BCRC 22015 / CBS 2517 / CECT 1963 / NBRC 1671 / NRRL Y-8276) | 1 | 0 | **23.6** | 0.12 |
|  |  | A0A023FMS2_9ACAR | Putative ribosome bioproteinsis protein | Eukaryota | Amblyomma cajennense | 1 | 0 | **23.4** | 0.15 |
|  |  | S9UXZ4_9TRYP | Cellular retinaldehyde-binding protein/triple function domain-containing protein | Eukaryota | Strigomonas culicis | 1 | 0 | **23.3** | 0.16 |
|  |  | C4L4A3_EXISA | Glycosyl transferase family 51 | Bacteria | Exiguobacterium sp. (strain ATCC BAA-1283 / AT1b) | 1 | 0 | **23.1** | 0.18 |
|  |  | A0A085C9C0_BACIU | Membrane protein | Bacteria | Bacillus subtilis | 1 | 0 | **22.9** | 0.21 |
|  |  | YTTA_BACSU | Uncharacterized membrane protein YttA | Bacteria | Bacillus subtilis (strain 168) | 1 | 0 | **22.9** | 0.21 |
|  |  | A6TVR5_ALKMQ | Integral membrane sensor signal transduction histidine kinase | Bacteria | Alkaliphilus metalliredigens (strain QYMF) | 1 | 0 | **22.8** | 0.22 |
|  |  | Q28264_CANFA | Junctional sarcoplasmic reticulum protein | Eukaryota | Canis familiaris | 1 | 0 | **22.8** | 0.23 |
|  |  | L7MMA2_OESDE | RIC-3 | Eukaryota | Oesophagostomum dentatum | 1 | 0 | **22.4** | 0.3 |
|  |  | C6H7B9_AJECH | Sec14 cytosolic factor | Eukaryota | Ajellomyces capsulatus (strain H143) | 1 | 0 | **22.3** | 0.33 |
|  |  | L9VVP2_9EURY | ATPase AAA containing von Willebrand factor type A (VWA) domain-like protein | Archaea | Natronorubrum tibetense GA33 | 1 | 0 | **22.2** | 0.35 |
|  |  | B5RV46_DEBHA | Vacuolar protein sorting-associated protein 29 | Eukaryota | Debaryomyces hansenii (strain ATCC 36239 / CBS 767 / JCM 1990 / NBRC 0083 / IGC 2968) | 1 | 0 | **22.0** | 0.39 |
|  |  | K2HQK2_ENTNP | Major facilitator superfamily protein | Eukaryota | Entamoeba nuttalli (strain P19) | 1 | 0 | **21.7** | 0.48 |
|  |  | W4GC94_9STRA | tRNA pseudouridine(55) synthase | Eukaryota | Aphanomyces astaci | 1 | 0 | **21.5** | 0.57 |
|  |  | F0U682_AJEC8 | SEC14 cytosolic factor | Eukaryota | Ajellomyces capsulatus (strain H88) | 1 | 0 | **21.5** | 0.58 |
|  |  | A0A078J4T8_BRANA | BnaCnng34340D protein | Eukaryota | Brassica napus | 1 | 0 | **21.4** | 0.6 |
|  |  | I2H4F9_TETBL | TBLA0E02080 protein | Eukaryota | Tetrapisispora blattae (strain ATCC 34711 / CBS 6284 / DSM 70876 / NBRC 10599 / NRRL Y-10934 / UCD 77-7) | 1 | 0 | **21.4** | 0.61 |
|  |  | H0DG78_9STAP | Nuclease-like protein | Bacteria | Staphylococcus pettenkoferi VCU012 | 1 | 0 | **21.4** | 0.62 |
| M-*ORF* | UniProtKB | M3UR84_ENTHI | Major facilitator superfamily protein | Eukaryota | Entamoeba histolytica HM-1:IMSS-B | 1 | 0 | **21.1** | 0.74 |
|  |  | K0NZP8_9LACO | Hypothetical membrane protein | Bacteria | Lactobacillus equicursoris DSM 19284 = JCM 14600 = CIP 110162 | 1 | 0 | **21.1** | 0.75 |
|  |  | A0A090BR33_KLUMA | Nucleolar protein 56 | Eukaryota | Kluyveromyces marxianus | 1 | 0 | **21.1** | 0.75 |
|  |  | W0TC29_KLUMA | Nucleolar protein 56 | Eukaryota | Kluyveromyces marxianus DMKU3-1042 | 1 | 0 | **21.1** | 0.75 |
|  |  | C5D4P5_GEOSW | Penicillin-binding protein transpeptidase | Bacteria | Geobacillus sp. (strain WCH70) | 1 | 0 | **21.0** | 0.8 |
|  |  | R6HQS4_9PROT | Putative uncharacterized membrane protein | Bacteria | Azospirillum sp. CAG:260 | 1 | 0 | **21.0** | 0.8 |
|  |  | F9N4N5_9FIRM | ATP synthase subunit b | Bacteria | Veillonella sp. oral taxon 780 str. F0422 | 1 | 0 | **20.8** | 0.96 |
|  |  | H2B1U5_KAZAF | KAFR0K02390 protein | Eukaryota | Kazachstania africana (strain ATCC 22294 / BCRC 22015 / CBS 2517 / CECT 1963 / NBRC 1671 / NRRL Y-8276) | 1 | 0 | **20.7** | 0.97 |
|  | SwissProt | YTTA_BACSU | Uncharacterized membrane protein YttA | Bacteria | Bacillus subtilis (strain 168) | 1 | 1 | **22.9** | 0.0023 |
|  |  | YZVL_CAEEL | Uncharacterized NOP5 family protein K07C5.4 | Eukaryota | Caenorhabditis elegans | 1 | 0 | 18.7 | 0.046 |
|  |  | PROQ_VIBF1 | RNA chaperone ProQ | Bacteria | Vibrio fischeri (strain ATCC 700601 / ES114) | 1 | 0 | 17.9 | 0.08 |
|  |  | PROQ_VIBFM | RNA chaperone ProQ | Bacteria | Vibrio fischeri (strain MJ11) | 1 | 0 | 17.9 | 0.08 |
|  |  | HDAC1_CHICK | Histone deacetylase 1 | Eukaryota | Gallus gallus | 1 | 0 | 17.4 | 0.12 |
|  |  | PROQ_PSYIN | RNA chaperone ProQ | Bacteria | Psychromonas ingrahamii (strain 37) | 1 | 0 | 17.0 | 0.15 |
|  |  | YYAB_BACSU | Uncharacterized protein YyaB | Bacteria | Bacillus subtilis (strain 168) | 1 | 0 | 16.9 | 0.17 |
|  |  | NU3M_YARLI | NADH-ubiquinone oxidoreductase chain 3 | Eukaryota | Yarrowia lipolytica (strain CLIB 122 / E 150) | 2 | 0 | 16.2 | 0.27 |
|  |  | LRC59_RAT | Leucine-rich repeat-containing protein 59 | Eukaryota | Rattus norvegicus | 1 | 0 | 16.1 | 0.28 |
|  |  | CT47A_HUMAN | Cancer/testis antigen 47A | Eukaryota | Homo sapiens | 1 | 0 | 15.9 | 0.33 |
|  |  | PROQ_ALISL | RNA chaperone ProQ | Bacteria | Aliivibrio salmonicida LFI1238 | 1 | 0 | 15.8 | 0.34 |
|  |  | LRC59_HUMAN | Leucine-rich repeat-containing protein 59 | Eukaryota | Homo sapiens | 1 | 0 | 15.8 | 0.36 |
| M-*ORF* | SwissProt | TNSB_ECOLX | Transposon Tn7 transposition protein TnsB | Bacteria | Escherichia coli | 1 | 0 | 15.7 | 0.37 |
|  |  | RPN2_CANGA | 26S proteasome regulatory subunit RPN2 | Eukaryota | Candida glabrata (strain ATCC 2001 / CBS 138 / JCM 3761 / NBRC 0622 / NRRL Y-65) | 1 | 0 | 15.5 | 0.44 |
|  |  | Y377_MYCGE | Uncharacterized protein MG377 | Bacteria | Mycoplasma genitalium (strain ATCC 33530 / G-37 / NCTC 10195) | 1 | 0 | 15.3 | 0.49 |
|  |  | ATPF_XYLFT | ATP synthase subunit b | Bacteria | Xylella fastidiosa (strain Temecula1 / ATCC 700964) | 1 | 0 | 14.9 | 0.67 |
|  |  | ATPF_XYLF2 | ATP synthase subunit b | Bacteria | Xylella fastidiosa (strain M23) | 1 | 0 | 14.9 | 0.67 |
|  |  | ATPF_BURVG | ATP synthase subunit b | Bacteria | Burkholderia vietnamiensis (strain G4 / LMG 22486) | 1 | 0 | 14.8 | 0.72 |
|  |  | ATPF_BURCC | ATP synthase subunit b | Bacteria | Burkholderia cenocepacia (strain MC0-3) | 1 | 0 | 14.8 | 0.74 |
|  |  | ATPF_BURCA | ATP synthase subunit b | Bacteria | Burkholderia cenocepacia (strain AU 1054) | 1 | 0 | 14.8 | 0.74 |
|  |  | ATPF_BURCH | ATP synthase subunit b | Bacteria | Burkholderia cenocepacia (strain HI2424) | 1 | 0 | 14.8 | 0.74 |
|  |  | ATPF_BURCJ | ATP synthase subunit b | Bacteria | Burkholderia cenocepacia (strain ATCC BAA-245 / DSM 16553 / LMG 16656 / NCTC 13227 / J2315 / CF5610) | 1 | 0 | 14.8 | 0.74 |
|  |  | ATPF_BURM1 | ATP synthase subunit b | Bacteria | Burkholderia multivorans | 1 | 0 | 14.7 | 0.75 |
|  |  | ATPF_BURA4 | ATP synthase subunit b | Bacteria | Burkholderia ambifaria (strain MC40-6) | 1 | 0 | 14.6 | 0.83 |
|  |  | ATPF_BURCM | ATP synthase subunit b | Bacteria | Burkholderia ambifaria (strain ATCC BAA-244 / AMMD) | 1 | 0 | 14.6 | 0.83 |
|  |  | SHDAG_HDVAM | Small delta antigen | Viruses | Hepatitis delta virus genotype I (isolate American) | 2 | 0 | 14.6 | 0.84 |
|  |  | OTCC_TREDE | Ornithine carbamoyltransferase, catabolic | Bacteria | Treponema denticola (strain ATCC 35405 / CIP 103919 / DSM 14222) | 1 | 0 | 14.4 | 0.93 |
|  | PDB | 3x1l_C | Cmr4 | Archaea | Archaeoglobus fulgidus DSM 4304 | 2 | 0 | 16.0 | 0.15 |
|  |  | 1a92_A | DELTA ANTIGEN | Viruses | Hepatitis delta virus | 1 | 0 | 14.8 | 0.36 |
| M-*ORF* | QfO | YTTA_BACSU | Uncharacterized membrane protein YttA | Bacteria | Bacillus subtilis (strain 168) | 2 | 1 | **22.9** | 0.0032 |
|  |  | YZVL_CAEEL | Uncharacterized NOP5 family protein K07C5.4 | Eukaryota | Caenorhabditis elegans | 1 | 0 | 18.7 | 0.064 |
|  |  | Q9LTV0_ARATH | NOP56-like pre RNA processing ribonucleoprotein | Eukaryota | Arabidopsis thaliana | 1 | 0 | 18.4 | 0.078 |
|  |  | HDAC1_CHICK | Histone deacetylase 1 | Eukaryota | Gallus gallus | 1 | 0 | 17.4 | 0.16 |
|  |  | YYAB_BACSU | Uncharacterized protein YyaB | Bacteria | Bacillus subtilis (strain 168) | 1 | 0 | 16.9 | 0.23 |
|  |  | Q7S9Y2_NEUCR | NMDA receptor-regulated protein 1 | Eukaryota | Neurospora crassa (strain ATCC 24698 / 74-OR23-1A / CBS 708.71 / DSM 1257 / FGSC 987) | 1 | 0 | 16.6 | 0.27 |
|  |  | Q9LJA1_ARATH | Expressed protein | Eukaryota | Arabidopsis thaliana | 1 | 0 | 16.3 | 0.35 |
|  |  | NU3M_YARLI | NADH-ubiquinone oxidoreductase chain 3 | Eukaryota | Yarrowia lipolytica (strain CLIB 122 / E 150) | 2 | 0 | 16.2 | 0.38 |
|  |  | LRC59_RAT | Leucine-rich repeat-containing protein 59 | Eukaryota | Rattus norvegicus | 1 | 0 | 16.1 | 0.39 |
|  |  | CT47A_HUMAN | Cancer/testis antigen 47A | Eukaryota | Homo sapiens | 1 | 0 | 15.9 | 0.46 |
|  |  | H2QDF0_PANTR | Leucine rich repeat containing 59 | Eukaryota | Pan troglodytes | 1 | 0 | 15.8 | 0.5 |
|  |  | LRC59_HUMAN | Leucine-rich repeat-containing protein 59 | Eukaryota | Homo sapiens | 1 | 0 | 15.8 | 0.5 |
|  | Pfamseq | G2RQY3_BACME | Excalibur domain protein | Bacteria | Bacillus megaterium WSH-002 | 1 | 1 | **30.3** | 0.0018 |
|  |  | G3H659_CRIGR | CKLF-like MARVEL transmembrane domain-containing protein 2B | Eukaryota | Cricetulus griseus | 1 | 0 | **27.4** | 0.014 |
|  |  | K2G8H3_9BACT | RNA binding S1 protein | Bacteria | uncultured bacterium (gcode 4) | 1 | 0 | **27.4** | 0.014 |
|  |  | R7N780_9FIRM | Electron transport complex subunit E | Bacteria | Firmicutes bacterium CAG:95 | 1 | 0 | **26.6** | 0.026 |
|  |  | J6PCC7_BACAN | Group-specific protein | Bacteria | Bacillus anthracis str. BF1 | 1 | 0 | **26.3** | 0.03 |
|  |  | W8HQB3_BACAN | Group-specific protein | Bacteria | Bacillus anthracis str. SVA11 | 1 | 0 | **26.3** | 0.03 |
|  |  | J6DVY5_BACAN | Group-specific protein | Bacteria | Bacillus anthracis str. UR-1 | 1 | 0 | **26.3** | 0.03 |
|  |  | I0D291_BACAN | Group-specific protein | Bacteria | Bacillus anthracis str. H9401 | 1 | 0 | **26.3** | 0.03 |
|  |  | Q63BB7_BACCZ | Group-specific protein | Bacteria | Bacillus cereus (strain ZK / E33L) | 1 | 0 | **26.3** | 0.03 |
|  |  | C1H9F4_PARBA | Nucleolar protein NOP56 | unclassified sequences | unclassified | 1 | 0 | **25.9** | 0.041 |
|  |  | E4SY79_LACDN | Hypothetical membrane protein | Bacteria | Lactobacillus delbrueckii subsp. bulgaricus ND02 | 1 | 0 | **25.8** | 0.043 |
|  |  | A5KSC4_9BACT | ATP synthase subunit b | Bacteria | candidate division TM7 genomosp. GTL1 | 1 | 0 | **24.9** | 0.083 |
| M-*ORF* | Pfamseq | Q8EWL2_MYCPE | Putative uncharacterized protein MYPE1910 | Bacteria | Mycoplasma penetrans (strain HF-2) | 1 | 0 | **24.4** | 0.12 |
|  |  | H2J4N9_MARPK | ATP synthase subunit b | Bacteria | Marinitoga piezophila (strain DSM 14283 / JCM 11233 / KA3) | 1 | 0 | **24.3** | 0.13 |
|  |  | A0A023FMS2_9ACAR | Putative ribosome bioproteinsis protein | Eukaryota | Amblyomma cajennense | 1 | 0 | **23.4** | 0.24 |
|  |  | S9UXZ4_9TRYP | Cellular retinaldehyde-binding protein/triple function domain-containing protein | Eukaryota | Strigomonas culicis | 1 | 0 | **23.3** | 0.26 |
|  |  | A0A031LCL5_ENTFC | MAEBL family membrane protein | Bacteria | Enterococcus faecium VRE0576 | 1 | 0 | **23.2** | 0.28 |
|  |  | C4L4A3_EXISA | Glycosyl transferase family 51 | Bacteria | Exiguobacterium sp. (strain ATCC BAA-1283 / AT1b) | 1 | 0 | **23.1** | 0.29 |
|  |  | C0SHR5_PARBP | Nucleolar protein 5A | Eukaryota | Paracoccidioides brasiliensis (strain Pb03) | 1 | 0 | **23.0** | 0.32 |
|  |  | V5MXR1_BACIU | Putative membrane protein yttA | Bacteria | Bacillus subtilis PY79 | 1 | 0 | **22.9** | 0.34 |
|  |  | YTTA_BACSU | Uncharacterized membrane protein YttA | Bacteria | Bacillus subtilis (strain 168) | 1 | 0 | **22.9** | 0.34 |
|  |  | M1U5M5_BACIU | YttA | Bacteria | Bacillus subtilis subsp. subtilis 6051-HGW | 1 | 0 | **22.9** | 0.34 |
|  |  | J7JVR5_BACIU | YttA | Bacteria | Bacillus subtilis QB928 | 1 | 0 | **22.9** | 0.34 |
|  |  | A6TVR5_ALKMQ | Integral membrane sensor signal transduction histidine kinase | Bacteria | Alkaliphilus metalliredigens (strain QYMF) | 1 | 0 | **22.8** | 0.36 |
|  |  | L7MMA2_OESDE | RIC-3 | Eukaryota | Oesophagostomum dentatum | 1 | 0 | **22.4** | 0.48 |
|  |  | C6H7B9_AJECH | Sec14 cytosolic factor | Eukaryota | Ajellomyces capsulatus (strain H143) (Darling's disease fungus) (Histoplasma capsulatum) | 1 | 0 | **22.3** | 0.53 |
|  |  | L9VVP2_9EURY | ATPase AAA containing von Willebrand factor type A (VWA) domain-like protein | Archaea | Natronorubrum tibetense GA33 | 1 | 0 | **22.2** | 0.57 |
|  |  | B5RV46_DEBHA | DEHA2G07304p | Eukaryota | Debaryomyces hansenii (strain ATCC 36239 / CBS 767 / JCM 1990 / NBRC 0083 / IGC 2968) | 1 | 0 | **22.0** | 0.62 |
|  |  | C5DTL9_ZYGRC | ZYRO0C09614p | Eukaryota | Zygosaccharomyces rouxii (strain ATCC 2623 / CBS 732 / NBRC 1130 / NCYC 568 / NRRL Y-229) | 1 | 0 | **21.8** | 0.76 |
| M-*ORF* | Pfamseq | K2HQK2_ENTNP | Major facilitator superfamily protein | Eukaryota | Entamoeba nuttalli (strain P19) (Amoeba) | 1 | 0 | **21.7** | 0.77 |
|  |  | W4GC94_9STRA | tRNA pseudouridine(55) synthase | Eukaryota | Aphanomyces astaci | 1 | 0 | **21.5** | 0.91 |
|  |  | F0U682_AJEC8 | SEC14 cytosolic factor | Eukaryota | Ajellomyces capsulatus (strain H88) (Darling's disease fungus) (Histoplasma capsulatum) | 1 | 0 | **21.5** | 0.94 |
|  |  | H0DG78_9STAP | Nuclease-like protein | Bacteria | Staphylococcus pettenkoferi VCU012 | 1 | 0 | **21.4** | 0.99 |
| F-*ORF* | UniProtKB | F4ZFW9_9BIVA | H-orf protein (Fragment) | Eukaryota | Lasmigona subviridis | 8 | 8 | **122.9** | **1.8E-32** |
|  |  | F4ZFN3_9BIVA | Female-specific orf protein | Eukaryota | Toxolasma lividus | 1 | 1 | **108.3** | **6.6E-28** |
|  |  | F4ZFH5_MARMG | Female-specific orf protein | Eukaryota | Margaritifera margaritifera | 1 | 1 | **101.6** | **7.9E-26** |
|  |  | F4ZFV5_VENEL | Female-specific orf protein | Eukaryota | Venustaconcha ellipsiformis | 1 | 1 | **92.4** | **5.9E-23** |
|  |  | F4ZFW3_LASCM | H-orf protein | Eukaryota | Lasmigona compressa | 5 | 5 | **92.1** | **7.6E-23** |
|  |  | F4ZFG1_LAMSI | Female-specific orf protein | Eukaryota | Lampsilis siliquoidea | 1 | 1 | **89.5** | **4.8E-22** |
|  |  | F4ZFG0_9BIVA | Female-specific orf protein | Eukaryota | Lampsilis powellii | 1 | 1 | **89.0** | **7.0E-22** |
|  |  | F4ZFE6_CUMMO | Female-specific orf protein | Eukaryota | Cumberlandia monodonta | 1 | 1 | **72.0** | **1.4E-16** |
|  |  | F4ZFF2_CYCTU | Female-specific orf protein | Eukaryota | Cyclonaias tuberculata | 1 | 1 | **70.8** | **3.3E-16** |
|  |  | V9PBQ9_9BIVA | F-ORF | Eukaryota | Solenaia carinatus | 1 | 1 | **70.6** | **3.9E-16** |
|  |  | F4ZFH4_LEMRI | Female-specific orf protein | Eukaryota | Lemiox rimosus | 1 | 1 | **69.4** | **9.0E-16** |
|  |  | F4ZFL6_9BIVA | Female-specific orf protein | Eukaryota | Quadrula houstonensis | 1 | 1 | **69.0** | **1.2E-15** |
|  |  | F4ZFF9_9BIVA | Female-specific orf protein | Eukaryota | Echyridella menziesii | 1 | 1 | **68.9** | **1.2E-15** |
|  |  | F4ZFF3_CYCTU | Female-specific orf protein | Eukaryota | Cyclonaias tuberculata | 1 | 1 | **68.4** | **1.8E-15** |
|  |  | F4ZFH7_9BIVA | Female-specific orf protein | Eukaryota | Margaritifera marrianae | 1 | 1 | **67.4** | **3.7E-15** |
|  |  | F4ZFF4_9BIVA | Female-specific orf protein | Eukaryota | Ellipsaria lineolata | 1 | 1 | **66.5** | **7.2E-15** |
|  |  | F4ZFK9_9BIVA | Female-specific orf protein | Eukaryota | Pyganodon lacustris | 1 | 1 | **66.1** | **9.8E-15** |
|  |  | F4ZFI6_PYGGR | Female-specific orf protein | Eukaryota | Pyganodon grandis | 1 | 1 | **66.0** | **1.0E-14** |
|  |  | F4ZFL2_9BIVA | Female-specific orf protein | Eukaryota | Pyganodon lacustris | 1 | 1 | **65.3** | **1.6E-14** |
|  |  | F4ZFW1_LASCM | H-orf protein | Eukaryota | Lasmigona compressa | 6 | 4 | **64.8** | **2.4E-14** |
|  |  | F4ZFI1_9BIVA | Female-specific orf protein | Eukaryota | Potamilus metnecktayi | 1 | 1 | **64.7** | **2.6E-14** |
| F-*ORF* | UniProtKB | F4ZFF6_FUSFL | Female-specific orf protein | Eukaryota | Fusconaia flava | 1 | 1 | **64.6** | **2.9E-14** |
|  |  | F4ZFE3_9BIVA | Female-specific orf protein | Eukaryota | Alasmidonta undulata | 1 | 1 | **63.3** | **6.9E-14** |
|  |  | F4ZFW6_LASCM | H-orf protein | Eukaryota | Lasmigona compressa | 3 | 3 | **63.2** | **7.7E-14** |
|  |  | X2CT99_9BIVA | H open reading frame | Eukaryota | Dahurinaia dahurica | 1 | 1 | **62.6** | **1.2E-13** |
|  |  | F4ZFN5_9BIVA | Female-specific orf protein | Eukaryota | Toxolasma paulus | 1 | 1 | **60.5** | **5.4E-13** |
|  |  | F4ZFF5_9BIVA | Female-specific orf protein | Eukaryota | Fusconaia ebenus | 1 | 1 | **58.5** | **2.2E-12** |
|  |  | F4ZFQ2_9BIVA | Female-specific orf protein | Eukaryota | Toxolasma texasiensis | 1 | 1 | **57.8** | **3.7E-12** |
|  |  | F4ZFE2_ALAMA | Female-specific orf protein | Eukaryota | Alasmidonta marginata | 1 | 1 | **57.5** | **4.6E-12** |
|  |  | F4ZFH0_9BIVA | Female-specific orf protein | Eukaryota | Lasmigona costata | 1 | 1 | **57.5** | **4.6E-12** |
|  |  | F4ZFP9_9BIVA | Female-specific orf protein | Eukaryota | Toxolasma paulus | 1 | 1 | **56.2** | **1.2E-11** |
|  |  | F4ZFW2_LASCM | H-orf protein | Eukaryota | Lasmigona compressa | 2 | 2 | **55.9** | **1.5E-11** |
|  |  | F4ZFY0_MARFC | H-orf protein | Eukaryota | Margaritifera falcata | 1 | 1 | **55.6** | **1.8E-11** |
|  |  | F4ZFT3_9BIVA | Female-specific orf protein | Eukaryota | Utterbackia peninsularis | 1 | 1 | **53.3** | **9.3E-11** |
|  |  | F4ZFI0_9BIVA | Female-specific orf protein | Eukaryota | Megalonaias nervosa | 1 | 1 | **51.6** | **3.3E-10** |
|  |  | F4ZFT2_9BIVA | Female-specific orf protein | Eukaryota | Utterbackia peggyae | 1 | 1 | **50.7** | **6.1E-10** |
|  |  | F4ZFL9_9BIVA | Female-specific orf protein | Eukaryota | Strophitus undulatus | 1 | 1 | **45.4** | **2.7E-08** |
|  |  | F4ZFM7_9BIVA | Female-specific orf protein | Eukaryota | Toxolasma glans | 1 | 1 | **44.5** | **5.4E-08** |
|  |  | F4ZFM0_9BIVA | Female-specific orf protein | Eukaryota | Strophitus undulatus | 1 | 1 | **43.4** | **1.2E-07** |
|  |  | F4ZFQ7_9BIVA | Female-specific orf protein | Eukaryota | Utterbackia peggyae | 1 | 1 | **42.3** | **2.5E-07** |
|  |  | U5KJG1_ANOAN | F-ORF | Eukaryota | Anodonta anatina | 1 | 1 | **42.0** | **3.0E-07** |
|  |  | F2WZ99_SINWO | F ORF | Eukaryota | Sinanodonta woodiana | 1 | 1 | **33.0** | **0.0002** |
|  |  | Q6D9Z7_PECAS | Putative membrane protein | Bacteria | Pectobacterium atrosepticum (strain SCRI 1043 / ATCC BAA-672) | 2 | 0 | **21.5** | 0.76 |
|  | PDB | 3tia_A | Neuraminidase | Viruses | Influenza A virus (A/RI/5+/1957(H2N2)) | 1 | 0 | 14.7 | 0.56 |
|  | QfO | Q59ZX2_CANAL | FTR1 family protein | Eukaryota | Candida albicans (strain SC5314 / ATCC MYA-2876) | 3 | 0 | 18.6 | 0.098 |
|  |  | A2ERK8_TRIVA | DNA polymerase epsilon. catalytic subunit, putative | Eukaryota | Trichomonas vaginalis | 2 | 0 | 16.1 | 0.59 |
| F-*ORF* | QfO | Q9VE38_DROME | CG14302 | Eukaryota | Drosophila melanogaster | 1 | 0 | 16.0 | 0.64 |
|  | Pfamseq | F4ZFW9_9BIVA | H-orf protein (Fragment) | Eukaryota | Lasmigona subviridis | 8 | 8 | **122.9** | **2.9E-32** |
|  |  | F4ZFN3_9BIVA | Female-specific orf protein | Eukaryota | Toxolasma lividus | 1 | 1 | **108.3** | **1.1E-27** |
|  |  | F4ZFH5_MARMG | Female-specific orf protein | Eukaryota | Margaritifera margaritifera | 1 | 1 | **101.6** | **1.3E-25** |
|  |  | F4ZFV5_VENEL | Female-specific orf protein | Eukaryota | Venustaconcha ellipsiformis | 1 | 1 | **92.4** | **9.5E-23** |
|  |  | F4ZFW3_LASCM | H-orf protein | Eukaryota | Lasmigona compressa | 5 | 5 | **92.1** | **1.2E-22** |
|  |  | F4ZFG1_LAMSI | Female-specific orf protein | Eukaryota | Lampsilis siliquoidea | 1 | 1 | **89.5** | **7.8E-22** |
|  |  | F4ZFV6_VENEL | Female-specific orf protein | Eukaryota | Venustaconcha ellipsiformis | 1 | 1 | **89.1** | **1.1E-21** |
|  |  | F4ZFG0_9BIVA | Female-specific orf protein | Eukaryota | Lampsilis powellii | 1 | 1 | **89.0** | **1.1E-21** |
|  |  | F4ZFW0_LASCM | H-orf protein (Fragment) | Eukaryota | Lasmigona compressa | 7 | 7 | **86.9** | **4.8E-21** |
|  |  | F4ZGB5_LASCM | H open reading frame | Eukaryota | Lasmigona compressa | 5 | 5 | **72.1** | **2.1E-16** |
|  |  | F4ZFE6_CUMMO | Female-specific orf protein | Eukaryota | Cumberlandia monodonta | 1 | 1 | **72.0** | **2.3E-16** |
|  |  | F4ZFF2_CYCTU | Female-specific orf protein | Eukaryota | Cyclonaias tuberculata | 1 | 1 | **70.8** | **5.3E-16** |
|  |  | V9PBQ9_9BIVA | F-ORF | Eukaryota | Solenaia carinatus | 1 | 1 | **70.6** | **6.3E-16** |
|  |  | F4ZFH4_LEMRI | Female-specific orf protein | Eukaryota | Lemiox rimosus | 1 | 1 | **69.4** | **1.4E-15** |
|  |  | F4ZFL6_9BIVA | Female-specific orf protein | Eukaryota | Quadrula houstonensis | 1 | 1 | **69.0** | **1.9E-15** |
|  |  | F4ZFF9_9BIVA | Female-specific orf protein | Eukaryota | Echyridella menziesii | 1 | 1 | **68.9** | **2.0E-15** |
|  |  | F4ZFF3_CYCTU | Female-specific orf protein | Eukaryota | Cyclonaias tuberculata | 1 | 1 | **68.4** | **3.0E-15** |
|  |  | F4ZFH7_9BIVA | Female-specific orf protein | Eukaryota | Margaritifera marrianae | 1 | 1 | **67.4** | **6.0E-15** |
|  |  | F4ZFF4_9BIVA | Female-specific orf protein | Eukaryota | Ellipsaria lineolata | 1 | 1 | **66.5** | **1.2E-14** |
|  |  | F4ZFK9_9BIVA | Female-specific orf protein | Eukaryota | Pyganodon lacustris | 1 | 1 | **66.1** | **1.6E-14** |
|  |  | F4ZFI6_PYGGR | Female-specific orf protein | Eukaryota | Pyganodon grandis | 1 | 1 | **66.0** | **1.7E-14** |
|  |  | F4ZFI1_9BIVA | Female-specific orf protein | Eukaryota | Potamilus metnecktayi | 1 | 1 | **64.7** | **4.1E-14** |
|  |  | F4ZFF6_FUSFL | Female-specific orf protein | Eukaryota | Fusconaia flava | 1 | 1 | **64.6** | **4.6E-14** |
|  |  | F4ZFE3_9BIVA | Female-specific orf protein | Eukaryota | Alasmidonta undulata | 1 | 1 | **63.3** | **1.1E-13** |
|  |  | F4ZFW6_LASCM | H-orf protein | Eukaryota | Lasmigona compressa | 3 | 3 | **63.2** | **1.2E-13** |
|  |  | X2CT99_9BIVA | H open reading frame | Eukaryota | Dahurinaia dahurica | 1 | 1 | **62.6** | **1.9E-13** |
| F-*ORF* | Pfamseq | F4ZFN5_9BIVA | Female-specific orf protein | Eukaryota | Toxolasma paulus | 1 | 1 | **60.5** | **8.6E-13** |
|  |  | F4ZFW5_LASCM | H-orf protein | Eukaryota | Lasmigona compressa | 3 | 3 | **60.4** | **9.1E-13** |
|  |  | F4ZFQ4_9BIVA | Female-specific orf protein | Eukaryota | Truncilla macrodon | 1 | 1 | **60.3** | **1.0E-12** |
|  |  | F4ZFF5_9BIVA | Female-specific orf protein | Eukaryota | Fusconaia ebenus | 1 | 1 | **58.5** | **3.5E-12** |
|  |  | F4ZFQ2_9BIVA | Female-specific orf protein | Eukaryota | Toxolasma texasiensis | 1 | 1 | **57.8** | **5.9E-12** |
|  |  | F4ZFE2_ALAMA | Female-specific orf protein | Eukaryota | Alasmidonta marginata | 1 | 1 | **57.5** | **7.4E-12** |
|  |  | F4ZFH0_9BIVA | Female-specific orf protein | Eukaryota | Lasmigona costata | 1 | 1 | **57.5** | **7.4E-12** |
|  |  | F4ZFI3_PYGGR | Female-specific orf protein | Eukaryota | Pyganodon grandis | 1 | 1 | **57.1** | **1.0E-11** |
|  |  | F4ZFY2_MARFC | H-orf protein | Eukaryota | Margaritifera falcata | 1 | 1 | **55.2** | **3.9E-11** |
|  |  | F4ZFU5_9BIVA | Female-specific orf protein | Eukaryota | Utterbackia peninsularis | 1 | 1 | **53.0** | **1.9E-10** |
|  |  | F4ZFP6_9BIVA | Female-specific orf protein | Eukaryota | Toxolasma paulus | 1 | 1 | **52.5** | **2.6E-10** |
|  |  | F4ZFI0_9BIVA | Female-specific orf protein | Eukaryota | Megalonaias nervosa | 1 | 1 | **51.6** | **5.3E-10** |
|  |  | F4ZFT2_9BIVA | Female-specific orf protein | Eukaryota | Utterbackia peggyae | 1 | 1 | **50.7** | **9.9E-10** |
|  |  | F4ZFQ5_9BIVA | Female-specific orf protein | Eukaryota | Utterbackia peggyae | 1 | 1 | **48.5** | **4.7E-09** |
|  |  | F4ZFU7_9BIVA | Female-specific orf protein | Eukaryota | Utterbackia peninsularis | 1 | 1 | **45.8** | **3.3E-08** |
|  |  | F4ZFL9_9BIVA | Female-specific orf protein | Eukaryota | Strophitus undulatus | 1 | 1 | **45.4** | **4.3E-08** |
|  |  | F4ZFM7_9BIVA | Female-specific orf protein | Eukaryota | Toxolasma glans | 1 | 1 | **44.5** | **8.7E-08** |
|  |  | F4ZFQ7_9BIVA | Female-specific orf protein | Eukaryota | Utterbackia peggyae | 1 | 1 | **42.3** | **4.0E-07** |
|  |  | U5KJG1_ANOAN | F-ORF | Eukaryota | Anodonta anatina | 1 | 1 | **42.0** | **4.9E-07** |
|  |  | F2WZ99_SINWO | F ORF | Eukaryota | Sinanodonta woodiana | 1 | 1 | **33.0** | **3.2E-04** |

note. – Proteins described only as “uncharacterized”, “putative”, or not annotated in general, have been removed since no information can be obtained. In bold are bit scores ≥20 and E-values ≤0.001. Results are ordered by profile, database, and E-value.

Table S11. Filtered hmmsearch output for the M-*ORF* and F-*ORF* HMM profiles built using custom parameters with hmmbuild (parameters: --fast --symfrac 0 --fragthresh 0 –-wnone --enone; see HMMER User’s Guide at ftp://selab.janelia.org/pub/software/hmmer/CURRENT/Userguide.pdf for details on the commands).

| *Profile* | *Database* | *Target* | *Description* | *Kingdom* | *Species* | *# hits* | *# significant hits* | *Bit Score* | *E-value* |
| --- | --- | --- | --- | --- | --- | --- | --- | --- | --- |
| M-*ORF* | UniProtKB | F4ZG80_9BIVA | M-specific morf protein | Eukaryota | Utterbackia peninsularis | 1 | 1 | **386.2** | **6.1E-112** |
|  |  | V9PBU4_9BIVA | M-ORF | Eukaryota | Solenaia carinatus | 1 | 1 | **190.5** | **8.2E-53** |
|  |  | A0A023I1I6_ANOAN | M-ORF | Eukaryota | Anodonta anatina | 2 | 2 | **117.7** | **8.2E-31** |
|  | SwissProt | OTCC_TREDE | Ornithine carbamoyltransferase, catabolic | Bacteria | Treponema denticola (strain ATCC 35405 / CIP 103919 / DSM 14222) | 2 | 0 | 13.4 | 0.28 |
|  |  | CDSA_DICDI | Probable phosphatidate cytidylyltransferase | Eukaryota | Dictyostelium discoideum | 1 | 0 | 12.5 | 0.51 |
|  | PDB | 2ml9_A | Yop proteins translocation protein U | Bacteria | Yersinia pseudotuberculosis IP 32953 | 1 | 0 | 14.1 | 0.085 |
|  | QfO | C3Z4U7_BRAFL | Putative uncharacterized protein | Eukaryota | Branchiostoma floridae | 1 | 0 | 14.7 | 0.16 |
|  |  | CDSA_DICDI | Probable phosphatidate cytidylyltransferase | Eukaryota | Dictyostelium discoideum | 1 | 0 | 12.5 | 0.71 |
|  | Pfamseq | F4ZG80_9BIVA | M-specific morf protein | Eukaryota | Utterbackia peninsularis | 1 | 1 | **386.2** | **9.8E-112** |
|  |  | V9PBU4_9BIVA | M-ORF | Eukaryota | Solenaia carinatus | 1 | 1 | **190.5** | **1.3E-52** |
|  |  | A0A023I1I6_ANOAN | M-ORF | Eukaryota | Anodonta anatina | 2 | 2 | **117.7** | **1.3E-30** |
| F-*ORF* | UniProtKB | F4ZFN3_9BIVA | Female-specific orf protein | Eukaryota | Toxolasma lividus | 1 | 1 | **216.9** | **1.3E-60** |
|  |  | F4ZFH5_MARMG | Female-specific orf protein | Eukaryota | Margaritifera margaritifera | 1 | 1 | **168.3** | **8.3E-46** |
|  |  | F4ZFW9_9BIVA | H-orf protein (Fragment) | Eukaryota | Lasmigona subviridis | 8 | 8 | **163.0** | **3.4E-44** |
|  |  | F4ZFV6_VENEL | Female-specific orf protein | Eukaryota | Venustaconcha ellipsiformis | 1 | 1 | **149.0** | **6.2E-40** |
|  |  | F4ZFX0_9BIVA | H-orf protein (Fragment) | Eukaryota | Lasmigona subviridis | 7 | 7 | **148.9** | **6.7E-40** |
|  |  | F4ZFW3_LASCM | H-orf protein | Eukaryota | Lasmigona compressa | 5 | 5 | **126.7** | **3.7E-33** |
|  |  | F4ZFH4_LEMRI | Female-specific orf protein | Eukaryota | Lemiox rimosus | 1 | 1 | **124.0** | **2.4E-32** |
|  |  | F4ZFI9_PYGGR | Female-specific orf protein | Eukaryota | Pyganodon grandis | 1 | 1 | **119.5** | **5.8E-31** |
|  |  | F4ZFG1_LAMSI | Female-specific orf protein | Eukaryota | Lampsilis siliquoidea | 1 | 1 | **119.2** | **7.2E-31** |
|  |  | F4ZFL2_9BIVA | Female-specific orf protein | Eukaryota | Pyganodon lacustris | 1 | 1 | **118.6** | **1.0E-30** |
|  |  | F4ZFG0_9BIVA | Female-specific orf protein | Eukaryota | Lampsilis powellii | 1 | 1 | **118.6** | **1.1E-30** |
| F-*ORF* | UniProtKB | V9PBQ9_9BIVA | F-ORF | Eukaryota | Solenaia carinatus | 1 | 1 | **117.2** | **3.0E-30** |
|  |  | F4ZFV7_9BIVA | Female-specific orf protein | Eukaryota | Villosa iris | 1 | 1 | **115.7** | **8.3E-30** |
|  |  | F4ZFI3_PYGGR | Female-specific orf protein | Eukaryota | Pyganodon grandis | 1 | 1 | **93.8** | **3.9E-23** |
|  |  | F4ZFF9_9BIVA | Female-specific orf protein | Eukaryota | Echyridella menziesii | 1 | 1 | **92.4** | **1.0E-22** |
|  |  | F4ZG87_9BIVA | F-specific orf protein | Eukaryota | Utterbackia peninsularis | 1 | 1 | **92.1** | **1.3E-22** |
|  |  | F4ZFF2_CYCTU | Female-specific orf protein | Eukaryota | Cyclonaias tuberculata | 1 | 1 | **91.1** | **2.5E-22** |
|  |  | F4ZFL6_9BIVA | Female-specific orf protein | Eukaryota | Quadrula houstonensis | 1 | 1 | **90.9** | **2.8E-22** |
|  |  | F4ZFT2_9BIVA | Female-specific orf protein | Eukaryota | Utterbackia peggyae | 1 | 1 | **90.0** | **5.3E-22** |
|  |  | F4ZFH3_9BIVA | Female-specific orf protein | Eukaryota | Lasmigona costata | 1 | 1 | **88.4** | **1.6E-21** |
|  |  | F4ZFF4_9BIVA | Female-specific orf protein | Eukaryota | Ellipsaria lineolata | 1 | 1 | **88.0** | **2.2E-21** |
|  |  | F4ZFW5_LASCM | H-orf protein | Eukaryota | Lasmigona compressa | 3 | 3 | **87.4** | **3.4E-21** |
|  |  | F4ZFS9_9BIVA | Female-specific orf protein | Eukaryota | Utterbackia peggyae | 1 | 1 | **85.6** | **1.2E-20** |
|  |  | F4ZFF6_FUSFL | Female-specific orf protein | Eukaryota | Fusconaia flava | 1 | 1 | **83.5** | **5.2E-20** |
|  |  | F4ZFQ2_9BIVA | Female-specific orf protein | Eukaryota | Toxolasma texasiensis | 1 | 1 | **82.8** | **8.1E-20** |
|  |  | F4ZFN5_9BIVA | Female-specific orf protein | Eukaryota | Toxolasma paulus | 1 | 1 | **80.6** | **3.9E-19** |
|  |  | F4ZFQ4_9BIVA | Female-specific orf protein | Eukaryota | Truncilla macrodon | 1 | 1 | **78.7** | **1.4E-18** |
|  |  | F4ZFN4_9BIVA | Female-specific orf protein | Eukaryota | Toxolasma paulus | 1 | 1 | **78.1** | **2.3E-18** |
|  |  | F4ZFI1_9BIVA | Female-specific orf protein | Eukaryota | Potamilus metnecktayi | 1 | 1 | **72.0** | **1.6E-16** |
|  |  | F4ZFP1_9BIVA | Female-specific orf protein | Eukaryota | Toxolasma paulus | 1 | 1 | **70.9** | **3.3E-16** |
|  |  | F4ZFF5_9BIVA | Female-specific orf protein | Eukaryota | Fusconaia ebenus | 1 | 1 | **68.5** | **1.8E-15** |
|  |  | F4ZFP9_9BIVA | Female-specific orf protein | Eukaryota | Toxolasma paulus | 1 | 1 | **67.7** | **3.3E-15** |
|  |  | F4ZFL9_9BIVA | Female-specific orf protein | Eukaryota | Strophitus undulatus | 1 | 1 | **65.0** | **2.1E-14** |
|  |  | F4ZFH7_9BIVA | Female-specific orf protein | Eukaryota | Margaritifera marrianae | 1 | 1 | **64.2** | **3.8E-14** |
|  |  | U5KJG1_ANOAN | F-ORF | Eukaryota | Anodonta anatina | 1 | 1 | **63.6** | **5.9E-14** |
|  |  | F4ZFL8_9BIVA | Female-specific orf protein | Eukaryota | Strophitus undulatus | 1 | 1 | **60.7** | **4.2E-13** |
|  |  | F4ZFX2_MARFC | H open reading frame | Eukaryota | Margaritifera falcata | 1 | 1 | **60.3** | **5.7E-13** |
|  |  | F4ZFM7_9BIVA | Female-specific orf protein | Eukaryota | Toxolasma glans | 1 | 1 | **59.2** | **1.2E-12** |
| F-*ORF* | UniProtKB | X2CT99_9BIVA | H open reading frame | Eukaryota | Dahurinaia dahurica | 1 | 1 | **59.2** | **1.2E-12** |
|  |  | F4ZFM0_9BIVA | Female-specific orf protein | Eukaryota | Strophitus undulatus | 1 | 1 | **58.4** | **2.2E-12** |
|  |  | F4ZFI0_9BIVA | Female-specific orf protein | Eukaryota | Megalonaias nervosa | 1 | 1 | **57.3** | **4.8E-12** |
|  |  | F2WZ99_SINWO | F ORF | Eukaryota | Sinanodonta woodiana | 1 | 1 | **39.2** | **1.5E-06** |
|  |  | A0A0C5RBW4_9MOLU | Strain ATCC 49782 genome | Bacteria | Ureaplasma diversum | 1 | 0 | **21.0** | 0.51 |
|  |  | A0A091H1Z3_BUCRH | Metalloreductase STEAP4 (Fragment) | Eukaryota | Buceros rhinoceros silvestris | 1 | 0 | **20.6** | 0.68 |
|  |  | A0A091Q5Q8_LEPDC | Metalloreductase STEAP4 (Fragment) | Eukaryota | Leptosomus discolor | 1 | 0 | **20.6** | 0.69 |
|  |  | C0F8Y4_9RICK | Efflux transporter, RND family, MFP subunit (Fragment) | Bacteria | Wolbachia endosymbiont of Muscidifurax uniraptor | 1 | 0 | **20.6** | 0.7 |
|  | SwissProt | NRAM_I68A6 | Neuraminidase | Viruses | Influenza A virus (A/Northern Territories/60-JY2/1968(H3N2)) | 1 | 0 | 16.1 | 0.17 |
|  |  | NRAM_I57A5 | Neuraminidase | Viruses | Influenza A virus (strain A/Singapore/1/1957 H2N2) | 1 | 0 | 14.9 | 0.4 |
|  |  | NRAM_I60A0 | Neuraminidase | Viruses | Influenza A virus (strain A/Ann Arbor/6/1960 H2N2) | 1 | 0 | 14.9 | 0.41 |
|  |  | NRAM_I66A1 | Neuraminidase | Viruses | Influenza A virus (strain A/Turkey/Wisconsin/1/1966 H9N2) | 1 | 0 | 14.3 | 0.6 |
|  |  | NRAM_I68A5 | Neuraminidase | Viruses | Influenza A virus (A/(Puerto Rico/8/1934-Korea/426/1968)(H2N2)) | 1 | 0 | 14.3 | 0.62 |
|  |  | NRAM_I67A0 | Neuraminidase | Viruses | Influenza A virus (strain A/Tokyo/3/1967 H2N2) | 1 | 0 | 14.3 | 0.62 |
|  | PDB | 3tia_A | Neuraminidase | Viruses | Influenza A virus (A/RI/5+/1957(H2N2)) | 1 | 0 | 14.9 | 0.2 |
|  | QfO | P73901_SYNY3 | 50S ribosomal protein L12 honologue | Bacteria | Synechocystis sp. (strain PCC 6803 / Kazusa) | 1 | 0 | 15.9 | 0.28 |
|  | Pfamseq | F4ZFN3_9BIVA | Female-specific orf protein | Eukaryota | Toxolasma lividus | 1 | 1 | **216.9** | **2.2E-60** |
|  |  | F4ZFH5_MARMG | Female-specific orf protein | Eukaryota | Margaritifera margaritifera | 1 | 1 | **168.3** | **1.3E-45** |
|  |  | F4ZFW9_9BIVA | H-orf protein (Fragment) | Eukaryota | Lasmigona subviridis | 8 | 8 | **163.0** | **5.4E-44** |
|  |  | F4ZFV6_VENEL | Female-specific orf protein | Eukaryota | Venustaconcha ellipsiformis | 1 | 1 | **149.0** | **1.0E-39** |
|  |  | F4ZFX0_9BIVA | H-orf protein (Fragment) | Eukaryota | Lasmigona subviridis | 7 | 7 | **148.9** | **1.1E-39** |
| F-*ORF* | Pfamseq | F4ZFV5_VENEL | Female-specific orf protein | Eukaryota | Venustaconcha ellipsiformis | 1 | 1 | **133.7** | **4.3E-35** |
|  |  | F4ZFW3_LASCM | H-orf protein | Eukaryota | Lasmigona compressa | 5 | 5 | **126.7** | **5.9E-33** |
|  |  | F4ZFH4_LEMRI | Female-specific orf protein | Eukaryota | Lemiox rimosus | 1 | 1 | **124.0** | **3.9E-32** |
|  |  | F4ZFI6_PYGGR | Female-specific orf protein | Eukaryota | Pyganodon grandis | 1 | 1 | **120.1** | **6.2E-31** |
|  |  | F4ZFG1_LAMSI | Female-specific orf protein | Eukaryota | Lampsilis siliquoidea | 1 | 1 | **119.2** | **1.2E-30** |
|  |  | F4ZFI8_PYGGR | Female-specific orf protein | Eukaryota | Pyganodon grandis | 1 | 1 | **118.6** | **1.7E-30** |
|  |  | F4ZFL2_9BIVA | Female-specific orf protein | Eukaryota | Pyganodon lacustris | 1 | 1 | **118.6** | **1.7E-30** |
|  |  | F4ZFG0_9BIVA | Female-specific orf protein | Eukaryota | Lampsilis powellii | 1 | 1 | **118.6** | **1.7E-30** |
|  |  | V9PBQ9_9BIVA | F-ORF | Eukaryota | Solenaia carinatus | 1 | 1 | **117.2** | **4.8E-30** |
|  |  | F4ZFV7_9BIVA | Female-specific orf protein | Eukaryota | Villosa iris | 1 | 1 | **115.7** | **1.3E-29** |
|  |  | F4ZFW0_LASCM | H-orf protein (Fragment) | Eukaryota | Lasmigona compressa | 7 | 7 | **115.1** | **2.0E-29** |
|  |  | F4ZFE6_CUMMO | Female-specific orf protein | Eukaryota | Cumberlandia monodonta | 1 | 1 | **113.0** | **8.8E-29** |
|  |  | F4ZFG2_LASCO | Female-specific orf protein | Eukaryota | Lasmigona complanata | 1 | 1 | **112.6** | **1.2E-28** |
|  |  | F4ZFK9_9BIVA | Female-specific orf protein | Eukaryota | Pyganodon lacustris | 1 | 1 | **110.3** | **5.6E-28** |
|  |  | F4ZFE8_CUMMO | Female-specific orf protein | Eukaryota | Cumberlandia monodonta | 1 | 1 | **108.8** | **1.7E-27** |
|  |  | F4ZFE3_9BIVA | Female-specific orf protein | Eukaryota | Alasmidonta undulata | 1 | 1 | **105.6** | **1.5E-26** |
|  |  | F4ZFL4_9BIVA | Female-specific orf protein | Eukaryota | Pyganodon lacustris | 1 | 1 | **103.7** | **5.7E-26** |
|  |  | F4ZFT3_9BIVA | Female-specific orf protein | Eukaryota | Utterbackia peninsularis | 1 | 1 | **96.8** | **7.5E-24** |
|  |  | F4ZFE2_ALAMA | Female-specific orf protein | Eukaryota | Alasmidonta marginata | 1 | 1 | **96.5** | **8.8E-24** |
|  |  | F4ZFH0_9BIVA | Female-specific orf protein | Eukaryota | Lasmigona costata | 1 | 1 | **96.5** | **8.8E-24** |
|  |  | F4ZFU5_9BIVA | Female-specific orf protein | Eukaryota | Utterbackia peninsularis | 1 | 1 | **95.9** | **1.4E-23** |
|  |  | F4ZFI3_PYGGR | Female-specific orf protein | Eukaryota | Pyganodon grandis | 1 | 1 | **93.8** | **6.2E-23** |
|  |  | F4ZFF9_9BIVA | Female-specific orf protein | Eukaryota | Echyridella menziesii | 1 | 1 | **92.4** | **1.6E-22** |
|  |  | F4ZFF2_CYCTU | Female-specific orf protein | Eukaryota | Cyclonaias tuberculata | 1 | 1 | **91.1** | **4.0E-22** |
|  |  | F4ZFL6_9BIVA | Female-specific orf protein | Eukaryota | Quadrula houstonensis | 1 | 1 | **90.9** | **4.5E-22** |
|  |  | F4ZFT2_9BIVA | Female-specific orf protein | Eukaryota | Utterbackia peggyae | 1 | 1 | **90.0** | **8.6E-22** |
|  |  | F4ZFU7_9BIVA | Female-specific orf protein | Eukaryota | Utterbackia peninsularis | 1 | 1 | **88.7** | **2.1E-21** |
| F-*ORF* | Pfamseq | F4ZFH3_9BIVA | Female-specific orf protein | Eukaryota | Lasmigona costata | 1 | 1 | **88.4** | **2.6E-21** |
|  |  | F4ZFF4_9BIVA | Female-specific orf protein | Eukaryota | Ellipsaria lineolata | 1 | 1 | **88.0** | **3.5E-21** |
|  |  | F4ZFW5_LASCM | H-orf protein | Eukaryota | Lasmigona compressa | 3 | 3 | **87.4** | **5.5E-21** |
|  |  | F4ZFF3_CYCTU | Female-specific orf protein | Eukaryota | Cyclonaias tuberculata | 1 | 1 | **87.0** | **6.8E-21** |
|  |  | F4ZFR5_9BIVA | Female-specific orf protein | Eukaryota | Utterbackia peggyae | 1 | 1 | **86.5** | **1.0E-20** |
|  |  | F4ZFQ4_9BIVA | Female-specific orf protein | Eukaryota | Truncilla macrodon | 1 | 1 | **78.7** | **2.3E-18** |
|  |  | F4ZFN4_9BIVA | Female-specific orf protein | Eukaryota | Toxolasma paulus | 1 | 1 | **78.1** | **3.6E-18** |
|  |  | F4ZFQ7_9BIVA | Female-specific orf protein | Eukaryota | Utterbackia peggyae | 1 | 1 | **74.3** | **5.0E-17** |
|  |  | F4ZFI1_9BIVA | Female-specific orf protein | Eukaryota | Potamilus metnecktayi | 1 | 1 | **72.0** | **2.6E-16** |
|  |  | F4ZFP1_9BIVA | Female-specific orf protein | Eukaryota | Toxolasma paulus | 1 | 1 | **70.9** | **5.4E-16** |
|  |  | F4ZFF5_9BIVA | Female-specific orf protein | Eukaryota | Fusconaia ebenus | 1 | 1 | **68.5** | **2.9E-15** |
|  |  | F4ZFH7_9BIVA | Female-specific orf protein | Eukaryota | Margaritifera marrianae | 1 | 1 | **64.2** | **6.1E-14** |
|  |  | U5KJG1_ANOAN | F-ORF | Eukaryota | Anodonta anatina | 1 | 1 | **63.6** | **9.5E-14** |
|  |  | F4ZFL8_9BIVA | Female-specific orf protein | Eukaryota | Strophitus undulatus | 1 | 1 | **60.7** | **6.8E-13** |
|  |  | F4ZFX2_MARFC | H-orf protein | Eukaryota | Margaritifera falcata | 1 | 1 | **60.3** | **9.2E-13** |
|  |  | F4ZFM7_9BIVA | Female-specific orf protein | Eukaryota | Toxolasma glans | 1 | 1 | **59.2** | **2.0E-12** |
|  |  | X2CT99_9BIVA | H open reading frame | Eukaryota | Dahurinaia dahurica | 1 | 1 | **59.2** | **2.0E-12** |
|  |  | F4ZFI0_9BIVA | Female-specific orf protein | Eukaryota | Megalonaias nervosa | 1 | 1 | **57.3** | **7.7E-12** |
|  |  | U5KJC3_ANOAN | F-ORF | Eukaryota | Anodonta anatina | 1 | 1 | **56.4** | **1.4E-11** |
|  |  | F2WZ99_SINWO | F ORF | Eukaryota | Sinanodonta woodiana | 1 | 1 | **39.2** | **2.4E-06** |

note. – Proteins described only as “uncharacterized”, “putative”, or not annotated in general, have been removed since no information can be obtained. In bold are bit scores ≥20 and E-values ≤0.001. Results are ordered by profile, database, and E-value.

Table S12. *Venustaconcha ellipsiformis* M*-ORF* function predictions

| **Hits** | **Species** | **Position** | **Probability** |
| --- | --- | --- | --- |
| **HHpred** | | | |
| TIGR03304 outer membrane insertion C-terminal signal |  | 158-164 | 99.11 |
| TIGR04294 prepilin-type processing-associated H-X9-DG domain |  | 26-29 | 99.01 |
| TIGR01167 LPXTG cell wall anchor domain |  | 17-39 | 98.99 |
| TIGR03057 X-X-X-Leu-X-X-Gly heptad repeats |  | 40-46 | 97.60 |
| Cutaneous T-cell lymphoma-associated antigen 1 isoform 1 | *Homo sapiens* | 21-171 | 97.99 |
| CTAGE family, member 5 isoform 2 | *Homo sapiens* | 13-171 | 97.92 |
| TIGR03501 GlyGly-CTERM domain (rank 14) |  | 22-35 | 96.47 |
| Nuclear Pore complex Protein family member (npp-11) | *Caenorhabditis elegans* | 62-217 | 97.08 |
| Essential subunit of the nuclear pore complex (NPC) |  | 87-218 | 96.25 |
| Subunit of the Nsp1p-Nup57p-Nup49p-Nic96p subcomplex of the nuclear pore complex (NPC) | *Saccharomyces cerevisiae* | 90-192 | 95.37 |
| Flagellar motor protein | *Agrobacterium tumefaciens* | 11-176 | 95.34 |
| Essential component of the nuclear pore complex | *Saccharomyces cerevisiae* | 44-170 | 95.24 |
| Collectin sub-family member 12 isoform II | *Homo sapiens* | 19-171 | 95.15 |
| CD207 antigen, langerin | *Mus musculus* | 19-149 | 94.92 |
| Peroxisomal membrane protein that is a central component of the peroxisomal protein import machinery | *Saccharomyces cerevisiae* | 27-167 | 94.76 |
| Structural constituent of nuclear pore | *Arabidopsis thaliana* | 61-217 | 94.64 |
| Fc fragment of IgE, low affinity II, receptor for (CD23A) | *Homo sapiens* | 24-157 | 94.54 |
| Structural constituent of nuclear pore | *Arabidopsis thaliana* | 44-172 | 94.40 |
| Scavenger receptor class A, member 3 | *Mus musculus* | 19-171 | 94.36 |
| Keratin 9 | *Homo sapiens* | 81-171 | 94.29 |
| Nucleoporin 62kDa | *Homo sapiens* | 90-218 | 93.89 |
| TIGR00756 pentatricopeptide repeat domain |  | 120-136 | 92.71 |
| F02E8.5 | *Caenorhabditis elegans* | 43-190 | 93.56 |
| CG4898-PF, isoform F | *Drosophila melanogaster* | 91-171 | 93.56 |
| CG4898-PK, isoform K | *Drosophila melanogaster* | 90-171 | 93.50 |
| Keratin complex 1, acidic, gene 9 | *Mus musculus* | 81-218 | 93.44 |
| Cancer susceptibility candidate 4 isoform b | *Homo sapiens* | 21-183 | 93.43 |
| Macrophage galactose N-acetyl-galactosamine specific lectin 2 |  | 20-171 | 93.40 |
| Laminin, beta 4 | *Homo sapiens* | 41-218 | 93.39 |
| Laminin, beta 1 precursor | *Homo sapiens* | 41-218 | 93.08 |
| Vacuolar protein sorting 37C | *Mus musculus* | 72-171 | 93.04 |
| PaREP5a | *Pyrobaculum aerophilum* | 89-171 | 92.83 |
| Laminin B1 subunit 1 | *Mus musculus* | 41-218 | 92.77 |
| Keratin 3 | *Homo sapiens* | 81-171 | 92.39 |
| Essential subunit of the nuclear pore complex (NPC) | *Saccharomyces cerevisiae* | 84-218 | 92.38 |
| B-cell receptor-associated protein BAP29 isoform c | *Homo sapiens* | 24-157 | 92.13 |
| Keratin 10 | *Homo sapiens* | 81-171 | 92.07 |
| Nuclear Pore complex Protein family member (npp-1) | *Caenorhabditis elegans* | 81-171 | 91.77 |
| Nucleotide binding | *Arabidopsis thaliana* | 90-213 | 91.70 |
| Collectin sub-family member 12 isoform I | *Homo sapiens* | 19-171 | 91.49 |
| CG16932-PC, isoform C | *Drosophila melanogaster* | 44-178 | 91.46 |
| PaREP5a | *Pyrobaculum aerophilum* | 79-171 | 91.21 |
| C27D6.4c | *Caenorhabditis elegans* | 81-293 | 91.19 |
| Type I hair keratin KA36 | *Homo sapiens* | 90-178 | 91.00 |
| Subunit of the Nsp1p-Nup57p-Nup49p-Nic96p subcomplex of the nuclear pore complex (NPC) | *Saccharomyces cerevisiae* | 107-345 | 90.77 |
| Shep3p Protein that acts as an adaptor between Myo4p and the She2p-mRNA complex | *Saccharomyces cerevisiae* | 50-171 | 90.76 |
| Keratin 1 | *Homo sapiens* | 81-177 | 90.64 |
| CG16932-PC, isoform C | *Drosophila melanogaster* | 44-155 | 90.63 |
| APG16 autophagy 16-like isoform 1 | *Homo sapiens* | 75-200 | 90.61 |
| Cortactin binding protein 2 | *Homo sapiens* | 46-183 | 90.59 |
| B-cell receptor-associated protein BAP29 isoform b | *Homo sapiens* | 24-155 | 90.58 |
| ATP synthase subunit I | *Aeropyrum pernix K1* | 54-171 | 90.51 |
| CG7123-PA, isoform A | *Drosophila melanogaster* | 44-214 | 90.39 |
| CG7123-PB, isoform B | *Drosophila melanogaster* | 44-214 | 90.39 |
| Nuclear Pore complex Protein family member (npp-1) | *Caenorhabditis elegans* | 45-182 | 90.32 |
| CG8831-PA | *Drosophila melanogaster* | 41-177 | 90.13 |
| **BLASTP** | | | |
| Chromosome segregation protein SMC |  | 38-196 | 3.45e-05 |
| Chromosome segregation ATPases |  | 44-212 | 2.02e-04 |
| Autophagy protein Apg6 |  | 67-180 | 1.77e-03 |
| RNA polymerase Rpb1 C-terminal repeat domain-containing protein | *Blastomyces dermatitidis* | 43-220 | 7e-05 |
| SMC domain-containing protein | *Thermodesulfatator indicus* | 72-162 | 0.008 |
| LPXTG-motif cell wall anchor domain | *Bacillus cytotoxicus* | 94-171 | 0.011 |
| Cell wall anchor protein | *Bacillus cytotoxicus* | 94-171 | 0.011 |
| Viral A-type inclusion protein | *Trichomonas vaginalis* | 53-111 | 0.031 |
| SMC1, partial | *Brachionus calyciflorus* | 52-111 | 0.089 |
| Intracellular protein transport protein USO1 | *Entamoeba dispar* | 74-123 | 0.099 |
| Chromosome segregation protein SMC | *Methanocaldococcus villosus* | 65-193 | 0.49 |
| **PSIBLAST** | | | |
| Chromosome segregation protein SMC, common bacterial type |  | 38-196 | 3.45e-05 |
| Chromosome segregation ATPases |  | 44-212 | 2.02e-04 |
| Autophagy protein Apg6 |  | 67-180 | 1.77e-03 |
| Ankyrin-3 | *Fukomys damarensis* | 51-218 | 4e-04 |
| Ankyrin-3 | *Heterocephalus glaber* | 51-218 | 5e-04 |
| Ankyrin-3 | *Pteropus alecto* | 51-218 | 0.001 |
| **Motif Scan** | | | |
| Lysine-rich region profile |  | 63-217 | 12.512 |
| **I-TASSER** |  |  |  |
| Tropomyosin | *Oryctolagus cuniculus* |  | 2.41 |
| Smooth muscle myosin heavy chain | *Gallus gallus* |  | 1.82 |
| Secreted 45kDa protein | *Streptococcus pneumoniae* |  | 1.75 |
| General control protein GCN4 and Tropomyosin 1 α chain | *Oryctolagus cuniculus* |  | 1.91 |
| RhUL123 | *Macacine herpesvirus 3* |  | 0.671 |
| Tyrosine-protein kinase Fes/Fps | *Homo sapiens* |  | 0.597 |
| SH3-containing GRB2-like protein 2 | *Homo sapiens* |  | 0.594 |
| Metastasis suppressor protein 1 | *Mus musculus* |  | 0.588 |
| Brain-specific angiogenesis inhibitor 1-associated protein 2-like protein 2 | *Mus musculus* |  | 0.588 |
| Formin-binding protein 1 | *Homo sapiens* |  | 0.582 |
| LEOA | *Escherichia coli* |  | 0.575 |
| ARF-GAP with coiled-coil, ANK repeat and PH domain-containing protein 1 | *Homo sapiens* |  | 0.574 |
| FCH domain only protein 2 | *Homo sapiens* |  | 0.569 |
| Brain-specific angiogenesis inhibitor 1-associated protein 2 | *Homo sapiens* |  | 0.568 |
| **Predict Protein** | |  |  |
| Protein binding |  | 1 |  |
| Cytoplasm |  |  |  |
| Ankyrin-3 | *Fukomys damarensis* |  | 9e-35, 0.06 |
| Coiled-coil domain-containing protein 6 | *Homo sapiens* |  | 1e-11 |
| Coiled-coil domain-containing protein 6 | *Mus musculus* |  | 1e-11 |
| Ankyrin-3 **(2)** | *Heterocephalus glaber* |  | 2e-33, 0.24 |
| Ankyrin-3 **(5)** | *Pteropus alecto* |  | 2e-33-0.5 |
| Myosin-6 **(3)** | *Mus musculus* |  | 6e-5- 0.77 |
| Myosin-6 **(3)** | *Rattus norvegicus* |  | 4e-5-0.54 |
| Myosin-7 **(3)** | *Canis familiaris* |  | 7e-5- 0.19 |
| Myosin-7 **(3)** | *Homo sapiens* |  | 5e-5-0.14 |
| Myosin-7 **(3)** | *Oryctolagus cuniculus* |  | 5e-5-0.061 |
| Unconventional myosin-Vc **(6)** | *Homo sapiens* |  | 1e-11- 0.71 |
| Myosin heavy chain, cardiac muscle isoform **(3)** | *Gallus gallus* |  | 2e-5- 0.58 |
| Myosin heavy chain, skeletal muscle **(2)** | *Oryctolagus cuniculus* |  | 3e-5, 8e-4 |
| Reticulocyte-binding protein 2 homolog a **(3)** | *Plasmodium falciparum* |  | 8e-5- 5e-4 |
| **Atome2** | | | |
| Myosin heavy chain, cardiac muscle beta isoform | *Homo sapiens* |  | 86.43 |
| Myosin-5A | *Gallus gallus* |  | 76.77 |
| Myosin heavy chain, cardiac muscle beta isoform | *Homo sapiens* |  | 73.37 |
| M protein | *Streptococcus pyogenes* |  | 60.89 |
| Protein Shroom | *Drosophila melanogaster* |  | 59.94 |
| Beclin-1 (Coiled Coil Domain) | *Rattus norvegicus* |  | 59.27 |
| Myosin-5A | *Gallus gallus* |  | 54.59 |
| ADP-ribosylation factor 6 (G domain, residues 13-175) | *Homo sapiens* |  | 53.93 |
| Rho-associated protein kinase 1 | *Homo sapiens* |  | 53.17, |
| Cell division protein ZAPB | *Escherichia coli* |  | 51.37 |
| Phosphoprotein | *Measles virus* |  | 49.33 |
| Tail needle protein gp26 | *Enterobacteria phage P22* |  | 48.96 |
| Ras-related protein SEC4 | *Saccharomyces cerevisiae* |  | 48.96 |
| C-JUN homodimer (leucine zipper domain | *Homo sapiens* |  | 38.90 |
| Secreted 45 kDa protein **(2)** | *Streptococcus pneumoniae* |  | 37.17, |
| HP0958 | *Helicobacter pylori* |  | 41.08 |

Table S13. *Quadrula quadrula* M*-ORF* function predictions

| **Hits** | **Species** | **Position** | **Probability** |
| --- | --- | --- | --- |
| **HHpred** | | | |
| TIGR03304 outer membrane insertion C-terminal signal |  | 19-21 | 99.19 |
| TIGR04294 prepilin-type processing-associated H-X9-DG domain |  | 70-71 | 99.14 |
| TIGR01167 LPXTG cell wall anchor domain |  | 8-28 | 99.12 |
| TIGR03057 X-X-X-Leu-X-X-Gly heptad repeats |  | 69-72 | 97.91 |
| TIGR03501 GlyGly-CTERM domain |  | 6-18 | 97.57 |
| TIGR00756 pentatricopeptide repeat domain |  | 48-60 | 93.60 |
| CG18146-PB, isoform B | *Drosophila melanogaster* | 9-36 | 89.69 |
| Syndecan 3 | *Mus musculus* | 2-45 | 88.44 |
| RCR |  | 9-27 | 87.35 |
| CG14181-PA | *Drosophila melanogaster* | 13-37 | 83.64 |
| CG18146-PA, isoform A | *Drosophila melanogaster* | 9-82 | 82.12 |
| BAS1 (PHYB activation tagged suppressor 1) | *Arabidopsis thaliana* | 3-34 | 81.33 |
| 4_hem_cytochrm_NapC |  | 18-38 | 80.73 |
| CG13461-PA | *Drosophila melanogaster* | 12-30 | 80.51 |
| Sso2p: Plasma membrane t-SNARE | *Saccharomyces cerevisiae* | 9-32 | 80.47 |
| Syndecan 3 | *Homo sapiens* | 10-45 | 80.23 |
| Syndecan 1 precursor | *Homo sapiens* | 8-45 | 79.98 |
| S-antigen | *Plasmodium falciparum* | 1-19 | 79.73 |
| Histidine kinase | *Nitrosopumilus maritimus* | 1-19 | 76.65 |
| Signal sequence receptor, alpha | *Homo sapiens* | 11-91 | 74.74 |
| RCR |  | 9-27 | 74.71 |
| COLlagen family member (col-36) | *Caenorhabditis elegans* | 2-42 | 73.61 |
| Signal sequence receptor, alpha | *Mus musculus* | 11-91 | 72.27 |
| UCP006158_SH3 |  | 9-27 | 72.08 |
| Maltose:maltodextrin transport system permease | *Haloferax volcanii DS2* | 9-38 | 71.87 |
| SYP124; t-SNARE | *Arabidopsis thaliana* | 9-37 | 70.40 |
| LCR19 | *Arabidopsis thaliana* | 1-21 | 69.65 |
| SYP121; t-SNARE | *Arabidopsis thaliana* | 9-46 | 68.98 |
| LCR59 | *Arabidopsis thaliana* | 1-21 | 68.58 |
| SYNtaxin family member (syn-2) | *Caenorhabditis elegans* | 9-34 | 67.79 |
| SYP121; t-SNARE | *Arabidopsis thaliana* | 9-46 | 67.78 |
| ZC190.8 | *Caenorhabditis elegans* | 3-33 | 67.59 |
| Y116A8C.41 | *Caenorhabditis elegans* | 5-39 | 66.98 |
| UCP006158_SH3 |  | 9-23 | 65.64 |
| Translocation associated membrane protein |  | 12-33 | 65.57 |
| SYNtaxin family member (syn-1) | *Caenorhabditis elegans* | 9-31 | 65.27 |
| T01B11.3 | *Caenorhabditis elegans* | 9-31 | 63.67 |
| Transmembrane protein | *Mycobacterium tuberculosis* |  |  |
| CG16707-PB, isoform B | *Drosophila melanogaster* | 10-27 | 63.06 |
| CG16707-PA, isoform A | *Drosophila melanogaster* | 10-27 | 63.06 |
| SQuaT family member (sqt-2) | *Caenorhabditis elegans* | 2-42 | 63.00 |
| CG12194-PA | *Drosophila melanogaster* | 12-67 | 62.94 |
| Copper ion binding / electron transporter | *Arabidopsis thaliana* | 16-39 | 62.72 |
| GGDEF family protein | *Beggiatoa sp. PS* | 1-35 | 62.40 |
| Y106G6E.2 | *Caenorhabditis elegans* | 10-28 | 62.07 |
| Lectin, mannose-binding 2 | *Mus musculus* | 1-33 | 61.96 |
| CG16707-PD, isoform D | *Drosophila melanogaster* | 11-34 | 61.28 |
| CG16707-PC, isoform C | *Drosophila melanogaster* | 11-34 | 61.28 |
| NHL25 (NDR1/HIN1-LIKE 25) | *Arabidopsis thaliana* | 10-37 | 60.76 |
| Serpentine Receptor, class X family member (srx-131) | *Caenorhabditis elegans* | 10-41 | 60.38 |
| C44H4.1 | *Caenorhabditis elegans* | 4-74 | 60.14 |
| TMEM171: Transmembrane protein family 171 |  | 10-34 | 60.03 |
| AC3.6 | *Caenorhabditis elegans* | 5-42 | 59.28 |
| Opsin 1, short-wave-sensitive | *Homo sapiens* | 8-41 | 59.09 |
| Metal ion binding | *Arabidopsis thaliana* | 9-37 | 58.54 |
| ATG27: Autophagy-related protein 27 |  | 6-32 | 57.82 |
| PsbI: Photosystem II reaction centre I protein |  | 8-22 | 57.67 |
| TcaA |  | 7-23 | 57.22 |
| **I-TASSER** | | | |
| Fumarate hydratase class II | *Mycobacterium tuberculosis* |  | 0.560 |
| Fumarate hydratase class II | *Mycobacterium smegmatis* |  | 0.559 |
| Fumarase Fum | *Mycobacterium marinum* |  | 0.558 |
| Adenylosuccinate lyase | *Mycobacterium smegmatis* |  | 0.548 |
| Argininosuccinate lyase | *Thermus thermophilus* |  | 0.548 |
| 3-carboxy-cis,cis-muconate cycloisomerase | *Pseudomonas putida* |  | 0.545 |
| Virion RNA polymerase | *Bacteriophage n4* |  | 0.542 |
| **Predict Protein** | | | |
| Protein binding |  | 42,66,105 |  |
| Nucleus |  |  |  |
| **Atome2** | | | |
| Flavocytochrome C sulfide dehydrogenase (Flavin-binding subunit) | *Allochromatium vinosum* |  | 62.97 |
| NADH-quinone oxidoreductase | *Thermus thermophilus HB8* |  | 60.23 |
| Voltage-gated sodium channel | *Caldalkalibacillus thermarum* |  | 42.44 |
| NADH-quinone oxidoreductase subunit L |  |  | 42.08 |
| Alpha-catenin (dimerization and beta-catenin binding region) | *Mus musculus* |  | 39.63 |
| Ion transport protein | *Arcobacter butzleri* |  | 36.63 |
| Collagen alpha 1 fragment 84-116 of NC1 | *Gallus gallus* |  | 36.49 |
| Apocytochrome f | *Chlamydomonas reinhardtii* |  | 31.45 |
| Disabled homolog 2 | *Homo sapiens* |  | 29.91 |
| Minicollagen-5 | *Hydra vulgaris* |  | 19.36 |
| Proline dehydrogenase | *Pseudomonas putida* |  | 17.32 |

Table S14. *Pyganodon grandis* M-*ORF* function predictions

| **Hits** | **Species** | **Position** | **Probability** |
| --- | --- | --- | --- |
| **HHpred** |  |  |  |
| TIGR04294 prepilin-type processing-associated H-X9-DG domain |  | 40-46 | 99.10 |
| TIGR01167 LPXTG cell wall anchor domain |  | 20-40 | 99.05 |
| TIGR03304 outer membrane insertion C-terminal signal |  | 55-59 | 98.89 |
| TIGR03057 X-X-X-Leu-X-X-Gly heptad repeats |  | 144-149 | 97.48 |
| TIGR03501 GlyGly-CTERM domain |  | 28-40 | 97.15 |
| TIGR00756 pentatricopeptide repeat domain |  | 29-41 | 92.96 |
| DYstrophin-like phenotype and CAPON related family member (dyc-1) | *Caenorhabditis elegans* | 164-232 | 89.28 |
| EGF-like-domain, multiple 5 | *Mus musculus* | 21-45 | 88.13 |
| CG32048-PA, isoform A | *Drosophila melanogaster* | 164-232 | 87.99 |
| CG2086-PB, isoform B | *Drosophila melanogaster* | 24-128 | 84.37 |
| Phosphoserine aminotransferase, PSAT | *Bacillus circulans,* | 104-140 | 79.56 |
| Negative regulator of septation ring formation | *Lactobacillus casei* | 25-60 | 78.48 |
| Golgi phosphoprotein 4 | *Mus musculus* | 25-47 | 78.23 |
| CG17213-PA | *Drosophila melanogaster* | 2-55 | 76.37 |
| Phosphoserine aminotransferase | *Beggiatoa sp. PS* | 104-140 | 76.27 |
| CG6124-PA | *Drosophila melanogaster* | 19-51 | 75.80 |
| F55C12.5c | *Caenorhabditis elegans* | 4-63 | 74.61 |
| Phosphoserine aminotransferase, PSAT | *Bacillus alcalophilus* | 104-140 | 74.12 |
| Cell division protein | *Yersinia pestis* | 20-47 | 73.99 |
| Phosphoserine_aminotransferase phosphoserine | Methanosarcina type | 91-140 | 73.60 |
| D2092.1a | *Caenorhabditis elegans* | 6-74 | 73.12 |
| Septation ring formation regulator EzrA | *Streptococcus pneumoniae* | 19-52 | 72.71 |
| ZK353.4 | *Caenorhabditis elegans* | 1-44 | 72.53 |
| PsbI: Photosystem II reaction centre I protein |  | 22-41 | 72.29 |
| Phosphoserine aminotransferase | *Beggiatoa sp. PS* | 46-140 | 70.86 |
| Photosystem II reaction center protein I, PsbI | *Thermosynechococcus vulcanus* | 22-41 | 69.88 |
| Cytochrome c oxidase, cbb3-type, CcoQ subunit |  | 22-52 | 69.88 |
| Photosystem II reaction center protein I, PsbI |  | 22-41 | 69.59 |
| Homoserine kinase ThrH | *Pseudomonas aeruginosa* | 116-163 | 69.41 |
| GtrA |  | 6-35 | 69.05 |
| CG2086-PA, isoform A | *Drosophila melanogaster* | 13-111 | 68.86 |
| Transmembrane protein | *Mycobacterium tuberculosis H37Rv* | 19-59 | 68.34 |
| Phosphoserine aminotransferase | *Mycobacterium tuberculosis H37Rv* | 104-183 | 68.17 |
| Podocalyxin-like precursor isoform 2 | *Homo sapiens* | 21-48 | 68.13 |
| ZK945.3 | *Caenorhabditis elegans* | 149-200 | 67.86 |
| Phosphoserine aminotransferase, PSAT | *Bacillus circulans,* | 104-140 | 67.51 |
| UBN_AB: Ubinuclein conserved middle domain |  | 149-217 | 67.29 |
| CG32177-PA (SD09769P) |  | 22-45 | 67.07 |
| Photosystem II reaction center I protein | *Synechococcus sp.* | 22-41 | 66.83 |
| Sec-independent translocase | *Agrobacterium tumefaciens* | 25-57 | 66.80 |
| Myc_target_1 Myc target protein 1. |  | 1-39 | 64.30 |
| Phosphoglycolate phosphatase, PGPase | *Pyrococcus horikoshii* | 116-176 | 64.22 |
| Sec-independent translocase | *Escherichia coli* | 25-53 | 63.02 |
| Essential cell division protein | *Escherichia coli* | 20-46 | 62.46 |
| Podocalyxin-like precursor isoform 1 | *Homo sapiens* | 21-48 | 61.69 |
| Nedd4 family interacting protein 2 | *Homo sapiens* | 20-45 | 61.47 |
| Phosphoserine aminotransferase | *Homo sapiens* | 104-140 | 61.38 |
| Phosphoserine aminotransferase (PSAT) family |  | 104-183 | 60.87 |
| F56H1.3 | *Caenorhabditis elegans* | 21-47 | 60.78 |
| Phosphoserine aminotransferase | *Salmonella enterica* | 104-140 | 60.66 |
| Penumbra | *Homo sapiens* | 10-59 | 60.60 |
| ACyLtransferase-like family member (acl-2) | *Caenorhabditis elegans* | 149-196 | 59.95 |
| Claudin domain containing 1 protein isoform a | *Homo sapiens* | 3-59 | 59.47 |
| Sec-independent protein translocase protein TatB | *Yersinia pestis CO92* | 25-57 | 59.28 |
| Phosphoserine aminotransferase, PSAT | *Bacillus alcalophilus* | 104-140 | 58.99 |
| Golgi phosphoprotein 4 | *Homo sapiens* | 29-47 | 58.44 |
| Membrane protein component of ABC phosphate transporter | *Pseudomonas aeruginosa* | 9-46 | 57.97 |
| Photosystem II reaction center protein I |  | 22-41 | 57.78 |
| Trans-golgi network protein 2 | *Homo sapiens* | 28-62 | 57.19 |
| Transmembrane protein | *Mycobacterium tuberculosis* | 6-42 | 55.77 |
| Phosphoserine aminotransferase, PSAT | *Escherichia coli* | 104-140 | 55.21 |
| Phosphoglycolate phosphatase | *Pyrococcus horikoshii* | 80-177 | 54.69 |
| CG7695-PA | *Drosophila melanogaster* | 20-37 | 54.45 |
| C18H2.4 | *Caenorhabditis elegans* | 21-60 | 54.21 |
| Claudin containing domain 1 | *Mus musculus* | 22-59 | 54.19 |
| Phosphomannomutase 1 | *Homo sapiens* | 116-160 | 54.15 |
| Myc_target_1: Myc target protein 1 |  | 1-39 | 54.06 |
| Translocation associated membrane protein |  | 173-220 | 54.03 |
| MiRP K channel accessory subunit family (mps-4) | *Caenorhabditis elegans* | 6-49 | 54.02 |
| MCTP-related |  | 9-75 | 53.90 |
| Sensor histidine kinase | *Bartonella henselae* | 9-47 | 53.80 |
| Phosphoserine aminotransferase | *Mycobacterium tuberculosis* | 91-140 | 53.25 |
| Phosphoserine aminotransferase | *Campylobacter jejuni* | 104-140 | 53.11 |
| Claudin domain containing 1 protein isoform b | *Homo sapiens* | 22-59 | 52.75 |
| Integral membrane protein | *Streptomyces coelicolor* | 26-54 | 52.57 |
| Y77E11A.12a | *Caenorhabditis elegans* | 9-62 | 52.43 |
| Twin arginine translocase protein A | *Frankia alni ACN14a* | 25-37 | 52.36 |
| **Motif Scan** | | | |
| Lysine-rich region profile |  | 142-233 | 12.082 |
| Bipartite nuclear localization signal profile |  | 183-199 | 4.000 |
| **I-TASSER** | | | |
| Type I hyperactive antifreeze protein | *Pseudopleuronectes americanus* |  | 1.64 |
| Antigen MTB48, Mycobacterial protein **(2)** | *Mycobacterium smegmatis* |  | 1.20, 1.37 |
| LEOA | *Escherichia coli* |  | 1.05 |
| Serine/threonine-protein kinase mTOR | *Homo sapiens* |  | 1.03 |
| Hemocyanin KLH1 | *Megathura crenulata* |  | 1.02 |
| Accumulation associated protein | *Staphylococcus epidermidis* |  | 1.33 |
| Flagellar hook-associated protein | *Burkholderia pseudomallei* |  | 0.572 |
| Type I hyperactive antifreeze protein | *Pseudopleuronectes americanus* |  | 0.563 |
| Flagellar hook-associated protein 1 | *Salmonella enterica* |  | 0.560 |
| Phospholipase C beta | *Meleagris gallopavo* |  | 0.546 |
| LEOA | *Escherichia coli* |  | 0.540 |
| 1-phosphatidylinositol 4,5-bisphosphate phosphodiesterase beta-3 | *Homo sapiens* |  | 0.532 |
| Multidrug resistance protein pgp-1 | *Caenorhabditis elegans* |  | 0.521 |
| Interferon-induced guanylate-binding protein 1 | *Homo sapiens* |  | 0.520 |
| **Predict Protein** | | | |
| Protein binding |  | 1 |  |
| Cytoplasm |  |  |  |
| Muscle M-line assembly protein unc-89 | *Harpegnathos saltator* |  | 0.19 |
| Conjugative transposon TraM protein | *Parabacteroides sp. 20_3* |  | 0.70 |
| **Atome2** | | | |
| Methly-coenzyme M reductase I alpha subunit | *Methanopyrus kandleri* |  | 85.07 |
| Dolichyl-diphosphooligosaccharide-protein glycosyltransferase subunit STT3 | *Saccharomyces cerevisiae* |  | 65.74 |
| Succinylglutamate desuccinylase | *Chromobacterium violaceum* |  | 55.83 |
| Matrix protein 1 | *Influenza A virus* |  | 39.16 |
| Receptor tyrosine-protein kinase erbB-2 | *Homo sapiens* |  | 38.24 |
| Cyclic nucleotide-gated cation channel alpha-3 | *Homo sapiens* |  | 37.26 |
| Talin-1 (F2F3 subdomain, UNP residues 206-405) | *Mus musculus* |  | 23.21 |
| Proteasome-associated ATPase (Coil coil domain) | *Mycobacterium tuberculosis* |  | 21.27 |
| Cytochrome c oxidase subunit 1 | *Thermus thermophilus* |  | 18.60 |
| Helix-destabilizing protein | *Enterobacteria phage T7* |  | 18.55 |
| Ion transport protein | *Magnetococcus marinus* |  | 18.17 |
| Myosin light chain | *Saccharomyces cerevisiae* |  | 16.20 |
| Soluble cytochrome b562, Smoothened homolog | *Homo sapiens* |  | 15.95 |
| Formaldehyde-activating enzyme fae | *Methylobacterium extorquens* |  | 13.89 |

Table S15. *Inversidens japanensis* M*-ORF* function predictions

| **Hits** | **Species** | **Position** | **Probability** |
| --- | --- | --- | --- |
| **HHpred** | | | |
| TIGR03304 outer membrane insertion C-terminal signal |  | 40-44 | 99.25 |
| TIGR04294 prepilin-type processing-associated H-X9-DG domain |  | 28-31 | 99.21 |
| TIGR01167 LPXTG cell wall anchor domain |  | 103-107 | 98.80 |
| TIGR03057 X-X-X-Leu-X-X-Gly heptad repeats |  | 46-64 | 97.73 |
| TIGR03501 GlyGly-CTERM domain |  | 22-35 | 96.74 |
| TIGR00756 pentatricopeptide repeat domain |  | 1-14 | 94.79 |
| CG32245-PB, isoform B | *Drosophila melanogaster* | 17-63 | 94.34 |
| Septum formation initiator | *Beggiatoa sp. PS* | 25-47 | 88.31 |
| CG32245-PA, isoform A | *Drosophila melanogaster* | 17-63 | 87.71 |
| CG32245-PC, isoform C | *Drosophila melanogaster* | 17-63 | 86.87 |
| T24B1.1 | *Caenorhabditis elegans* | 3-48 | 85.10 |
| SYP51 (SYNTAXIN OF PLANTS 51) **(2)** | *Arabidopsis thaliana* | 1-42 | 83.61 |
| STOmatin family member (sto-6) | *Caenorhabditis elegans* | 19-59 | 82.81 |
| Photosystem II reaction center protein PsbN | *Synechococcus sp.* | 15-44 | 79.61 |
| STOmatin family member (sto-4) | *Caenorhabditis elegans* | 21-59 | 79.10 |
| MEchanosensory abnormality family member (mec-2) | *Caenorhabditis elegans* | 21-63 | 78.75 |
| Stomatin isoform a | *Homo sapiens* | 20-63 | 78.31 |
| Podocin | *Homo sapiens* | 21-63 | 76.82 |
| Golgi autoantigen, golgin subfamily a, 5 | *Homo sapiens* | 3-48 | 76.72 |
| F0F1-type ATP synthase, subunit b | *Lactobacillus casei* | 20-45 | 76.70 |
| SYP52 (SYNTAXIN OF PLANTS 52) **(2)** | *Arabidopsis thaliana* | 1-41 | 76.63 |
| Stomatin (Epb7.2)-like 3 | *Mus musculus* | 21-59 | 76.25 |
| UNCoordinated family member (unc-1) | *Caenorhabditis elegans* | 19-63 | 74.89 |
| STOmatin family member (sto-2) | *Caenorhabditis elegans* | 21-63 | 74.40 |
| CG7635-PA | *Drosophila melanogaster* | 21-59 | 72.95 |
| Ring finger protein 183 | *Mus musculus* | 24-63 | 71.09 |
| CG14644-PA | *Drosophila melanogaster* | 21-63 | 70.71 |
| ATP synthase F0 B subunit | *Desulfitobacterium sp.* | 23-45 | 69.95 |
| Y45F3A.8 | *Caenorhabditis elegans* | 24-37 | 69.72 |
| F0F1 ATP synthase subunit B | *Escherichia coli* | 23-45 | 69.20 |
| Cell divison protein FtsB | *Escherichia coli* | 26-53 | 68.45 |
| Sec20 |  | 23-42 | 68.00 |
| ATP synthase (subunit b) | *Bacillus subtilis* | 22-45 | 67.79 |
| CG14736-PA, isoform A | *Drosophila melanogaster* | 21-59 | 66.71 |
| Cell divison protein FtsB | *Yersinia pestis CO92* | 26-47 | 65.60 |
| Tumor endothelial marker 8 isoform 2 precursor | *Homo sapiens* | 17-42 | 63.80 |
| MEChanosensory abnormality family member (mec-2) | *Caenorhabditis elegans* | 19-63 | 62.96 |
| DNA repair protein complementing XP-A cells |  | 59-114 | 62.85 |
| Pheromone-regulated protein, induced during cell integrity signalling | *Saccharomyces cerevisiae* | 15-42 | 62.69 |
| ATP synthase B/B' CF(0) |  | 23-45 | 62.51 |
| ATP synthase chain b'''' | *Synechococcus sp. CC9311* | 22-45 | 62.44 |
| S-antigen |  | 24-42 | 62.44 |
| PaTched Related family member (ptr-12) | *Caenorhabditis elegans* | 2-41 | 62.40 |
| Stomatin-prohibitin homolog, transmembrane | *Haloferax volcanii DS2* | 22-59 | 62.08 |
| D2085.6 | *Caenorhabditis elegans* | 2-45 | 62.01 |
| Endoplasmic Reticulum-Golgi Intermediate Compartment (ERGIC) |  | 3-41 | 60.13 |
| CG10737-PA, isoform A | *Drosophila melanogaster* | 26-94 | 59.87 |
| Translocation associated membrane protein |  | 67-106 | 59.79 |
| Golgi membrane protein, similar to mammalian CASP |  | 23-43 | 59.55 |
| DNaJ domain family member (dnj-26) | *Caenorhabditis elegans* | 4-42 | 59.53 |
| ATP synthase subunit B | *Corynebacterium diphtheria* | 19-45 | 59.33 |
| Stomatin-like 3 | *Homo sapiens* | 19-59 | 59.04 |
| Anthrax toxin receptor 1 | *Mus musculus* | 17-42 | 58.78 |
| CG10737-PB, isoform B | *Drosophila melanogaster* | 26-94 | 58.62 |
| STOmatin family member (sto-1) | *Caenorhabditis elegans* | 17-59 | 58.61 |
| ATBS14A; protein transporter | *Arabidopsis thaliana* | 4-41 | 57.72 |
| CG10737-PC, isoform C | *Drosophila melanogaster* | 26-94 | 57.24 |
| CG10737-PD, isoform D | *Drosophila melanogaster* | 26-94 | 57.24 |
| CG31358-PA | *Drosophila melanogaster* | 21-59 | 57.24 |
| Nephrosis 2 homolog, podocin | *Mus musculus* | 19-59 | 56.75 |
| SYP61 | *Arabidopsis thaliana* | 1-39 | 56.60 |
| Melanocortin 2 receptor accessory protein isoform alpha | *Homo sapiens* | 23-37 | 55.13 |
| ATP synthase subunit B | *Bartonella henselae* | 23-45 | 54.94 |
| ATP synthase F0, B subunit | *Streptococcus pneumoniae* | 22-45 | 54.79 |
| Melanocortin 2 receptor accessory protein isoform beta | *Homo sapiens* | 23-37 | 54.11 |
| C35D10.8 | *Caenorhabditis elegans* | 4-43 | 54.11 |
| CG13409-PA | *Drosophila melanogaster* | 14-46 | 53.62 |
| ATP synthase subunit B | *Streptomyces coelicolor* | 19-45 | 53.22 |
| Actin binding | *Arabidopsis thaliana* | 26-38 | 52.92 |
| AFH1 | *Arabidopsis thaliana* | 25-36 | 52.85 |
| **BLASTP** | | | |
| ATP synthase F0 subunit B | *Lachnospiraceae bacterium* | 18-116 |  |
| **Motif Scan** | | | |
| Lysine-rich region profile |  | 49-118 | 10.073 |
| **I-TASSER** | | | |
| Nucleotidyltransferase | *Agrobacterium fabrum* |  | 0.668 |
| Bacteriorhodopsin | *Halobacterium salinarum* |  | 0.666 |
| Halorhodopsin | *Natronomonas pharaonis* |  | 0.665 |
| Deltarhodopsin | *Haloterrigena sp.* |  | 0.663 |
| Archaerhodopsin-1 | *Halorubrum chaoviator* |  | 0.662 |
| Archaerhodopsin-2 | *Halobacterium sp. AUS-2* |  | 0.660 |
| Cruxrhodopsin-3 | *Haloarcula vallismortis* |  | 0.657 |
| Halorhodopsin | *Halobacterium salinarum* |  | 0.652 |
| **Predict Protein** | | | |
| Protein binding |  | 4,43,90,92 |  |
| Polynucleotide binding |  | 64 |  |
| Mitochondrion |  |  |  |
| **Atome2** | | | |
| Second mitochondria-derived activator of caspases | *Homo sapiens* |  | 76.53 |
| Guanine nucleotide exchange factor P115RHOGEF | *Homo sapiens* |  | 71.51 |
| Rep (DNA-bindig domain) | *Escherichia coli* |  | 69.62 |
| Nuclear factor of activated T-cells | *Homo sapiens* |  | 37.72 |
| Antifreeze peptide SS-3 | *Myoxocephalus scorpius* |  | 34.95 |
| Nonstructural protein 5A (BVDV NS5A) | *Bovine viral diarrhea virus* |  | 33.37 |
| Functional anti-apoptotic factor vBCL-2 homolog | *Human herpesvirus 8* |  | 27.14 |
| Ion transport protein (Pore and cytoplasmic domains) | *Alkalilimnicola ehrlichii* |  | 25.55 |
| Thymosin alpha-1 | *Homo sapiens* |  | 24.55 |
| Cytochrome c oxidase subunit 1 | *Thermus thermophilus* |  | 24.36 |
| Ion transport protein | *Magnetococcus marinus* |  | 23.40 |
| NHE1 isoform of Na+/H+ exchanger | *Meriones unguiculatus* |  | 23.11 |
| Apoptosis regulator BAK | *Homo sapiens* |  | 18.22 |

Table S16. *Utterbackia peninsularis* M-*ORF* function predictions

| **Hits** | **Species** | **Position** | **Probability** |
| --- | --- | --- | --- |
| **HHpred** | | | |
| TIGR03304 outer membrane insertion C-terminal signal |  | 53-60 | 99.16 |
| TIGR04294 prepilin-type processing-associated H-X9-DG domain |  | 41-44 | 99.06 |
| TIGR01167 LPXTG cell wall anchor domain |  | 18-38 | 98.89 |
| TIGR03057 X-X-X-Leu-X-X-Gly heptad repeats |  | 107-121 | 97.32 |
| TIGR03501 GlyGly-CTERM domain |  | 26-38 | 97.24 |
| TIGR00756 pentatricopeptide repeat domain |  | 32-39 | 92.61 |
| F58B4.4 | *Caenorhabditis elegans* | 59-97 | 84.64 |
| EGF-like-domain, multiple 5 | *Mus musculus* | 19-43 | 78.12 |
| F13D12.5 | *Caenorhabditis elegans* | 15-46 | 77.77 |
| T21B10.4 | *Caenorhabditis elegans* | 15-46 | 75.99 |
| Catenin alpha-1; four helix bundle, cell adhesion | *Mus musculus* | 145-158 | 75.96 |
| ZK945.3 | *Caenorhabditis elegans* | 132-183 | 72.81 |
| Cytochrome c oxidase |  | 20-50 | 72.15 |
| Transcriptional regulator | *Lactobacillus casei* | 134-163 | 71.49 |
| Pheromone-regulated protein, DUP240 gene family | *Saccharomyces cerevisiae* | 6-85 | 71.19 |
| Peptidase A24A prepilin type IV | *Candidatus Korarchaeum cryptofilum OPF8* | 10-63 | 69.06 |
| Mitochondrial ribosomal protein S23 |  | 44-51 | 65.64 |
| CG18146-PB, isoform B | *Drosophila melanogaster* | 21-47 | 65.59 |
| Catenin alpha-1; four helix bundle | *Mus musculus* | 145-158 | 61.01 |
| Beta-lactamase | *Pseudomonas fluorescens* | 143-163 | 60.65 |
| Phosphatidylserine decarboxylase | *Methanosarcina mazei Go1* | 19-102 | 60.03 |
| Septation ring formation regulator EzrA | *Streptococcus pneumoniae* | 16-42 | 59.30 |
| Reticulon 1 isoform A | *Homo sapiens* | 4-56 | 59.03 |
| Trigger factor ribosome-binding domain (102735) SCOP seed sequence: d1w26a2 |  | 141-167 | 58.82 |
| Hup-type Ni,Fe-hydrogenase cytochrome b subunit | *Desulfitobacterium hafniense Y51* | 4-78 | 57.93 |
| Transcriptional regulator, TetR family | *Staphylococcus aureus* | 140-163 | 57.48 |
| CG32048-PA, isoform A | *Drosophila melanogaster* | 147-216 | 57.23 |
| Essential cell division protein | *Escherichia coli K12* | 18-44 | 56.08 |
| Trigger factor ribosome-binding domain |  | 141-167 | 56.00 |
| F56H1.3 | *Caenorhabditis elegans* | 19-45 | 55.38 |
| YajQ-like (89963) SCOP seed sequence: d1in0a1 |  | 143-164 | 54.62 |
| Transmembrane protein | *Mycobacterium tuberculosis* | 4-40 | 54.42 |
| POTASSIUM VOLTAGE-GATED CHANNEL SUBFAMILY E MEMBER 1, 3. |  | 20-38 | 53.73 |
| Multi-sensor hybrid histidine kinase | *Nostoc punctiforme* | 8-62 | 53.52 |
| Acyltransferase | *Streptomyces coelicolor* | 126-160 | 53.51 |
| PELOTA_1 PELOTA RNA binding domain. |  | 131-172 | 53.49 |
| F55C12.5c | *Caenorhabditis elegans* | 2-61 | 51.82 |
| Y77E11A.12a | *Caenorhabditis elegans* | 4-60 | 50.58 |
| Transcriptional regulator MvaT, P16 subunit | *Pseudomonas aeruginosa* | 130-213 | 49.58 |
| Sensor histidine kinase | *Bartonella henselae* | 1-50 | 49.57 |
| Zinc finger, DHHC domain containing 5 | *Mus musculus* | 2-45 | 49.44 |
| D2092.1a | *Caenorhabditis elegans* | 4-84 | 49.18 |
| Olfactory receptor, family 6, subfamily C, member 6 | *Homo sapiens* | 1-44 | 48.98 |
| C18H2.4 | *Caenorhabditis elegans* | 20-45 | 48.86 |
| Secretory carrier-associated membrane protein |  | 26-53 | 48.67 |
| Thiamin diphosphate-binding fold (THDP-binding) |  | 91-152 | 48.59 |
| PELOTA RNA binding domain |  | 141-172 | 48.40 |
| AMPC beta-Lactamase, class C | *Citrobacter freundii* | 140-163 | 47.16 |
| Transcriptional regulator, TetR family protein | *Streptococcus pneumoniae* | 144-163 | 46.07 |
| Chromatin regulatory protein SIR2 |  | 97-119 | 45.86 |
| Toll-like receptor 7 | *Mus musculus* | 20-80 | 45.51 |
| K+_transpter_TRK |  | 20-48 | 45.40 |
| Sec-independent protein translocase protein TatB | *Yersinia pestis* | 23-55 | 44.37 |
| Zinc finger DHHC domain-containing protein |  | 10-51 | 44.23 |
| RNA recognition motif in regulators of calcineurin (RCANs) and similar proteins |  | 137-159 | 44.00 |
| Maltose: maltodextrin transport system permease | *Haloferax volcanii* | 20-53 | 43.91 |
| Phospholipid/glycerol acyltransferase | *Beggiatoa sp. PS* | 131-176 | 43.89 |
| Podocalyxin-like precursor isoform 2 | *Homo sapiens* | 19-46 | 43.69 |
| Alpha amylase catalytic domain |  | 129-164 | 43.63 |
| Beta-lactamase ACT-1 | *Klebsiella pneumoniae* | 140-163 | 43.59 |
| Metal ion binding | *Arabidopsis thaliana* | 2-43 | 43.14 |
| Neopullulanase, central domain | *Bacillus stearothermophilus* | 129-164 | 42.86 |
| Neopullulanase, central domain | *Bacillus stearothermophilus* | 129-164 | 42.86 |
| GH36 glycosyl hydrolase family 36 (GH36) |  | 143-164 | 42.83 |
| Trigger factor, TF; chaperone | *Thermotoga maritima* | 140-167 | 42.76 |
| T22E7.2 | *Caenorhabditis elegans* | 10-45 | 42.75 |
| Zinc finger, DHHC domain containing 5 | *Homo sapiens* | 2-45 | 42.71 |
| UCP016495 |  | 149-189 | 42.37 |
| Organic solute transporter-related |  | 3-36 | 41.59 |
| Trigger_N: Bacterial trigger factor protein (TF) |  | 140-167 | 41.54 |
| SUPFAM template c.1.8 (Trans) glycosidases |  | 141-164 | 41.40 |
| **Motif Scan** | | | |
| Lysine-rich region profile |  | 115-206 | 9.500 |
| Bipartite nuclear localization signal profile |  | 166-180 | 4.000 |
| **I-TASSER** | | | |
| 4-Hydroxybutyrate CoA-transferase | *Clostridium aminobutyricum* |  | 0.605 |
| 4-hydroxybutyrate coenzyme A transferase | *Shewanella oneidensis* |  | 0.604 |
| 4-hydroxybutyrate CoA-transferase | *Porphyromonas gingivalis* |  | 0.603 |
| Coenzyme A transferase | *Yersinia pestis* |  | 0.598 |
| 4-hydroxybutyrate CoA-transferase | *Porphyromonas gingivalis* |  | 0.565 |
| Succinyl-CoA:acetate coenzyme A transferase | *Acetobacter aceti* |  | 0.565 |
| Acetyl-CoA hydrolase/transferase family protein | *Porphyromonas gingivalis* |  | 0.553 |
| **Predict Protein** | | | |
| Protein binding |  | 41,77,92,98,118,127 |  |
| Cytoplasm |  |  |  |
| **Atome2** | | | |
| Vinculin | *Gallus gallus* |  | 50.72 |
| ADP-ribosylation factor binding protein GGA | *Homo sapiens* |  | 42.58 |
| 80 kDa MCM3-associated protein | *Homo sapiens* |  | 41.19 |
| LCoR protein | *Homo sapiens* |  | 36.28 |
| Transcriptional repressor COPG | *Streptococcus agalactiae* |  | 34.49 |
| HIG1 domain family member 1B | *Homo sapiens* |  | 29.87 |
| Uncharacterized protein 56B (transcription repressor) | *Sulfolobus islandicus rod-shaped virus 1* |  | 27.96 |
| Talin-1 (F2F3 subdomain, UNP residues 206-405) | *Mus musculus* |  | 26.08 |
| ScpA | *Geobacillus sp.* |  | 22.94 |
| HIG1 domain family member 1A | *Homo sapiens* |  | 21.45 |
| PlnE | *Lactobacillus plantarum* |  | 15.35 |

Table S17. *Solenaia carinatus* M*-ORF* function predictions

| **Hits** | **Species** | **Position** | **Probability** |
| --- | --- | --- | --- |
| **HHpred** | | | |
| TIGR04294 prepilin-type processing-associated H-X9-DG domain |  | 27-30 | 99.15 |
| TIGR01167 LPXTG cell wall anchor domain |  | 80-85 | 98.88 |
| TIGR03304 outer membrane insertion C-terminal signal |  | 6-7 | 98.75 |
| TIGR03057 X-X-X-Leu-X-X-Gly heptad repeats |  | 60-67 | 97.95 |
| TIGR03501 GlyGly-CTERM domain |  | 21-36 | 97.04 |
| TIGR00756 pentatricopeptide repeat domain |  | 3-13 | 94.60 |
| W06F12.2a | *Caenorhabditis elegans* | 20-59 | 90.96 |
| SAP |  | 38-58 | 90.80 |
| S-antigen |  | 23-41 | 90.41 |
| ZK973.11 | *Caenorhabditis elegans* | 11-43 | 80.48 |
| Thioredoxin domain containing 10 | *Homo sapiens* | 11-43 | 79.74 |
| UDP-Gal:betaGlcNAc beta 1,4- galactosyltransferase 5 | *Homo sapiens* | 12-51 | 77.75 |
| V-type ATPase 116 kDa subunit | *Nitrosopumilus maritimus* | 4-60 | 70.53 |
| Thioredoxin domain containing 10 | *Mus musculus* | 11-113 | 70.01 |
| Golgi autoantigen, golgin subfamily a, 5 | *Homo sapiens* | 22-47 | 69.90 |
| Photosystem II reaction center protein PsbN | *Synechococcus sp.* | 14-45 | 68.30 |
| CG10207-PA | *Drosophila melanogaster* | 23-131 | 67.76 |
| PRP38 pre-mRNA processing factor 38 (yeast) domain containing B | *Homo sapiens* | 4-58 | 64.40 |
| CG14084-PB, isoform B | *Drosophila melanogaster* | 5-40 | 63.61 |
| CG14084-PA, isoform A | *Drosophila melanogaster* | 5-40 | 63.61 |
| ZK757.4b | *Caenorhabditis elegans* | 23-45 | 61.92 |
| F12B6.2b | *Caenorhabditis elegans* | 22-124 | 59.84 |
| Binding | *Arabidopsis thaliana* | 3-58 | 59.77 |
| V-type ATP synthase subunit I | *Methanopyrus kandleri* | 4-60 | 59.58 |
| F12B6.2a | *Caenorhabditis elegans* | 10-141 | 59.54 |
| Golgi membrane protein with similarity to mammalian CASP | *Saccharomyces cerevisiae* | 22-41 | 59.34 |
| Hydrolase, hydrolyzing O-glycosyl compounds | *Arabidopsis thaliana* | 23-44 | 59.22 |
| CG1622-PA | *Drosophila melanogaster* | 4-44 | 58.83 |
| CG17287-PA | *Drosophila melanogaster* | 23-45 | 58.07 |
| Sec20 membrane glycoprotein associated with secretory pathway |  | 3-41 | 57.96 |
| CG14181-PA | *Drosophila melanogaster* | 1-56 | 57.83 |
| DNA-binding transcription factor required for the activation of the GAL genes in response to galactose | *Saccharomyces cerevisiae* | 1-41 | 57.24 |
| Y15E3A.4 | *Caenorhabditis elegans* | 22-89 | 56.55 |
| AC3.10 | *Caenorhabditis elegans* | 22-45 | 56.54 |
| ZK757.4a | *Caenorhabditis elegans* | 23-46 | 56.20 |
| Y116A8C.41 | *Caenorhabditis elegans* | 23-58 | 55.43 |
| Actin binding | *Arabidopsis thaliana* | 25-33 | 55.06 |
| CG30272-PA | *Drosophila melanogaster* | 22-119 | 54.95 |
| Similar to S. cerevisiae PKR1 | *Schizosaccharomyces pombe* | 7-61 | 54.74 |
| PRP38 pre-mRNA processing factor 38 (yeast) domain containing B | *Mus musculus* | 4-44 | 54.67 |
| Protein transporter | *Arabidopsis thaliana* | 2-41 | 54.37 |
| VAC_I2L |  | 4-42 | 54.08 |
| Y47H9C.2 | *Caenorhabditis elegans* | 14-46 | 53.55 |
| Metal ion binding | *Arabidopsis thaliana* | 22-46 | 53.52 |
| Metal ion binding | *Arabidopsis thaliana* | 22-46 | 53.13 |
| Photosystem II reaction center protein N | *Nostoc punctiforme* | 21-45 | 53.05 |
| ATBS14A; protein transporter | *Arabidopsis thaliana* | 2-41 | 52.99 |
| BCL2/adenovirus E1B 19kD interacting protein 1 isoform BNIP1 | *Homo sapiens* | 2-52 | 52.89 |
| Sarcolycans |  | 21-42 | 52.86 |
| Binding | *Arabidopsis thaliana* | 4-58 | 52.79 |
| T12G3.7 | *Caenorhabditis elegans* | 26-88 | 52.77 |
| Membrane associated histidine-rich protein, MAHRP-1 |  | 1-41 | 52.62 |
| Integral membrane protein | *Streptomyces coelicolor* | 22-49 | 52.09 |
| CG6627-PA | *Drosophila melanogaster* | 21-46 | 51.18 |
| Zinc finger, DHHC domain containing 15 | *Mus musculus* | 22-45 | 51.04 |
| CG32245-PB, isoform B | *Drosophila melanogaster* | 16-43 | 50.50 |
| CHL00020 psbN photosystem II protein N |  | 21-45 | 50.47 |
| ZC190.8 | *Caenorhabditis elegans* | 21-41 | 50.24 |
| PsbN: Photosystem II reaction centre N protein (psbN) |  | 21-45 | 49.96 |
| Y51F10.4b | *Caenorhabditis elegans* | 5-43 | 49.92 |
| ATP synthase subunit I | *Sulfolobus solfataricus* | 4-60 | 48.84 |
| Erf4: Golgin subfamily A member 7/ERF4 family |  | 5-58 | 48.74 |
| Photosystem I subunit III | *Synechocystis sp.* | 13-42 | 48.61 |
| PsbN Photosystem II reaction centre N protein (psbN) |  | 21-45 | 47.86 |
| CG8421-PB, isoform B | *Drosophila melanogaster* | 20-46 | 47.40 |
| Rab5ip Rab5-interacting protein (Rab5ip) |  | 23-61 | 47.18 |
| Subunit III of photosystem I reaction centre, PsaF | *Synechococcus elongatus* | 13-42 | 47.18 |
| Stomatin-like 3 | *Homo sapiens* | 18-43 | 47.17 |
| **BLASTP** | | | |
| Transmembrane protein 72, partial | *Anas platyrhynchos* | 47-129 | 0.95 |
| **Motif Scan** |  |  |  |
| Lysine-rich region profile |  | 48-119 | 11.508 |
| **Predict Protein** | | | |
| Protein binding |  | 17,19,43,45,66 |  |
| Polynucleotide binding |  | 49, 63 |  |
| Mitochondrion |  |  |  |
| **Atome2** | | | |
| RAD50 ABC-ATPase (N-terminal domain) | *Pyrococcus furiosus* |  | 84.72 |
| Cytoplasmic FMR1-interacting protein 1 | *Homo sapiens* |  | 65.70 |
| Chromosomal replication initiator protein dnaA | *Mycoplasma genitalium* |  | 62.22 |
| KIAA0380 (RGS-like domain (residues 281-490)) | *Homo sapiens* |  | 59.65 |
| Autophagy protein 1 (coiled-coil domain) | *Saccharomyces cerevisiae* |  | 52.37 |
| V-type ATP synthase subunit E | *Methanocaldococcus jannaschii* |  | 51.34 |
| Nuclear pore complex protein Nup54 (UNP residues 346-407) | *Rattus norvegicus* |  | 51.32 |
| B-cell receptor-associated protein 31 | *Homo sapiens* |  | 46.96 |
| Protein NRD1 (CTD-interacting domain, unp residues 6-151) | *Saccharomyces cerevisiae* |  | 45.58 |
| Photosystem I P700 chlorophyll a apoprotein A1 | *Synechococcus elongatus* |  | 42.88 |
| Transcription factor ATF-4 | *Homo sapiens* |  | 38.86 |
| Cytochrome c oxidase subunit 1 | *Thermus thermophilus* |  | 27.52 |
| Bcl-2-like protein 2 (UNP residues 2-171) | *Bos taurus* |  | 24.75 |
| Lmo2059 protein (KVLM pore module, truncated C-terminus (UNP residues 98-233)) | *Listeria monocytogenes* |  | 24.64 |
| Nonstructural protein 5A (BVDV NS5A) | *Bovine viral diarrhea virus* |  | 22.35 |
| DNA-(apurinic or apyrimidinic site) lyase | *Homo sapiens* |  | 4.84 |

Table S18. *Cumberlandia monodonta* M*-ORF* function predictions

| **Hits** | **Species** | **Position** | **Prob-**  **ability** |
| --- | --- | --- | --- |
| **HHpred** | | | |
| TIGR04294 prepilin-type processing-associated H-X9-DG domain |  | 30-34 | 99.52 |
| TIGR03304 outer membrane insertion C-terminal signal |  | 51-56 | 99.20 |
| TIGR01167 LPXTG cell wall anchor domain |  | 14-35 | 98.77 |
| TIGR03057 X-X-X-Leu-X-X-Gly heptad repeats |  | 13-16 | 97.81 |
| TIGR03501 GlyGly-CTERM domain |  | 16-26 | 97.14 |
| TIGR00756 pentatricopeptide repeat domain |  | 39-51 | 94.35 |
| CG9552-PA | *Drosophila melanogaster* | 18-60 | 87.49 |
| YMF19 Plant ATP synthase F0 |  | 17-43 | 84.86 |
| Secretory carrier-associated membrane protein |  | 18-38 | 84.05 |
| Protein binding | *Arabidopsis thaliana* | 16-54 | 81.54 |
| F01E11.3 | *Caenorhabditis elegans* | 16-24 | 80.16 |
| YMF19: Plant ATP synthase F0 |  | 17-46 | 69.49 |
| ATP synthase subunit B | *Agrobacterium tumefaciens* | 17-50 | 67.80 |
| Selenoprotein_S: Selenoprotein S (SelS) |  | 8-42 | 66.63 |
| VP35_FiloV |  | 13-21 | 63.95 |
| CYTOCHROME C1 |  | 21-48 | 63.67 |
| F56F4.7 | *Caenorhabditis elegans* | 57-95 | 62.52 |
| Carrier | *Arabidopsis thaliana* | 18-38 | 62.02 |
| Syndecan |  | 16-41 | 59.22 |
| CG18146-PB, isoform B | *Drosophila melanogaster* | 20-51 | 55.88 |
| CG30389-PA, isoform A | *Drosophila melanogaster* | 18-37 | 55.74 |
| CG30389-PC, isoform C | *Drosophila melanogaster* | 18-37 | 55.74 |
| CG2023-PA | *Drosophila melanogaster* | 17-38 | 55.68 |
| CG30415-PB, isoform B | *Drosophila melanogaster* | 9-39 | 54.50 |
| CG30415-PA, isoform A | *Drosophila melanogaster* | 9-39 | 54.50 |
| SC3 (secretory carrier 3) | *Arabidopsis thaliana* | 18-49 | 53.74 |
| Y46G5A.26b | *Caenorhabditis elegans* | 18-26 | 53.07 |
| CD4.1 | *Caenorhabditis elegans* | 9-46 | 52.82 |
| F0F1 ATP synthase subunit B' |  | 17-50 | 50.26 |
| SCAMP homolog family member (scm-1) | *Caenorhabditis elegans* | 18-38 | 50.18 |
| Protein binding | *Arabidopsis thaliana* | 16-48 | 48.42 |
| Agal |  | 11-18 | 48.41 |
| CG16707-PD, isoform D | *Drosophila melanogaster* | 20-42 | 47.94 |
| CG16707-PC, isoform C | *Drosophila melanogaster* | 20-42 | 47.94 |
| Sperm-associated cation channel 2 | *Mus musculus* | 15-33 | 47.25 |
| BCL2/adenovirus E1B 19kD interacting protein 1 isoform BNIP1-c | *Homo sapiens* | 17-38 | 46.86 |
| Transmembrane protein 57 | *Mus musculus* | 18-37 | 46.30 |
| Syntaxin 7 | *Homo sapiens* | 17-39 | 44.74 |
| Carrier | *Arabidopsis thaliana* | 18-34 | 44.59 |
| Syntaxin 7 | *Mus musculus* | 17-39 | 44.47 |
| Carrier | *Arabidopsis thaliana* | 18-34 | 43.51 |
| S-antigen | *Plasmodium falciparum* | 18-32 | 42.94 |
| Y47D7A.13 | *Caenorhabditis elegans* | 7-26 | 42.68 |
| G-protein-linked Acetylcholine Receptor family member (gar-1) | *Caenorhabditis elegans* | 18-49 | 41.32 |
| Transmembrane protein 57 | *Homo sapiens* | 18-37 | 41.20 |
| ATP synthase subunit B | *Streptomyces coelicolor* | 17-50 | 40.92 |
| ATP synthase subunit B | *Bartonella henselae* | 17-49 | 40.84 |
| Homeodomain-like (46689) SCOP seed sequence: d1hlva2; InterPro: GO: 0003677 DNA binding. |  | 10-21 | 40.78 |
| Related to Secretory carrier-associated membrane protein 2 |  | 18-34 | 40.31 |
| C15A7.2 | *Caenorhabditis elegans* | 20-59 | 40.10 |
| Zinc beta-ribbon (57783) SCOP seed sequence |  | 25-31 | 39.90 |
| Carrier | *Arabidopsis thaliana* | 18-34 | 39.62 |
| Secretory carrier membrane protein 1 isoform 1 | *Homo sapiens* | 18-34 | 38.98 |
| PeRoxireDoXin family member (prdx-6) | *Caenorhabditis elegans* | 21-45 | 38.74 |
| E set domains (81296) SCOP seed sequence: d1eh9a1 |  | 54-64 | 38.39 |
| High affinity copper uptake protein 1; HCTR1 TMDS, oligomerization, metal transport | *Homo sapiens* | 15-24 | 37.97 |
| F11G11.10 | *Caenorhabditis elegans* | 14-24 | 37.43 |
| CG32177-PA | *Drosophila melanogaster* | 14-42 | 37.06 |
| NK inhibitory receptor precursor | *Homo sapiens* | 18-41 | 36.95 |
| DUF4381 Domain of unknown function (DUF4381) |  | 16-46 | 36.93 |
| CG3268-PA | *Drosophila melanogaster* | 18-40 | 36.73 |
| UCP014405 |  | 33-54 | 36.16 |
| CG9195-PA, isoform A | *Drosophila melanogaster* | 18-34 | 35.18 |
| CG15673-PA | *Drosophila melanogaster* | 17-41 | 35.13 |
| Metal ion binding | *Arabidopsis thaliana* | 41-93 | 34.53 |
| Metal ion binding | *Arabidopsis thaliana* | 41-93 | 34.53 |
| ATP synthase subunit B | *Bartonella henselae str. Houston-1* | 17-35 | 34.36 |
| SRF-like (55455) SCOP seed sequence: d1mnma |  | 36-50 | 34.35 |
| Secretory carrier membrane protein 2 | *Mus musculus* | 18-34 | 34.20 |
| BCL2/adenovirus E1B 19kD interacting protein 1 isoform BNIP1-b | *Homo sapiens* | 17-38 | 33.55 |
| **BLASTP** | | | |
| Plant ATP synthase F0 |  | 17-46 |  |
| **Motif Scan** | | | |
| Lysine-rich region profile |  | 38-93 | 10.360 |
| Prokaryotic membrane lipoprotein lipid attachment site profile |  | 1-29 | 6.000 |
| **I-TASSER** | | | |
| Enoyl-CoA hydratase EchA1 | *Mycobacterium marinum* |  | 1.76 |
| 40S ribosomal protein S4, X isoform | *Homo sapiens* |  | 1.22 |
| Enoyl-coA hydratase/isomerase | *Mycobacterium abscessus* |  | 1.23 |
| Enoyl-CoA hydratase EchA17 | *Mycobacterium marinum* |  | 1.20 |
| Carnitinyl-CoA dehydrates | *Mycobacterium avium* |  | 1.19 |
| Enoyl-CoA hydratase, EchA12_1 | *Mycobacterium marinum* |  | 1.19 |
| Enoyl-CoA hydratase/carnithine racemase | *Magnetospirillum magneticum* |  | 1.50 |
| Enoyl-CoA hydratase/isomerase family protein | *Bacillus anthracis* |  | 0.882 |
| Enoyl-CoA hydratase | *Mycobacterium smegmatis* |  | 0.876 |
| Methylglutaconyl-CoA hydratase | *Homo sapiens* |  | 0.875 |
| Enoyl-CoA hydratase echA8 | *Mycobacterium tuberculosis* |  | 0.874 |
| Enoyl-CoA hydratase | *Mycobacterium smegmatis* |  | 0.874 |
| Naphthoate synthase | *Staphylococcus aureus* |  | 0.873 |
| 2,3-dehydroadipyl-CoA hydratase | *Escherichia coli* |  | 0.872 |
| **Predict Protein** | | | |
| Protein binding |  | 1,18,29,33,36,46 |  |
| Polynucleotide binding |  | 92 |  |
| Nucleus |  |  |  |
| **Atome2** | | | |
| SVP1-like protein 2 | *Kluyveromyces lactis* |  | 78.90 |
| Rhomboid Intramembrane Protease | *Pseudomonas aeruginosa* |  | 63.14 |
| U3 small nucleolar RNA-associated protein 22 | *Saccharomyces cerevisiae* |  | 43.16 |
| DNA-directed RNA polymerase subunit alpha | *Escherichia coli* |  | 42.29 |
| Myosin-X (MyTH4-FERM tandem) | *Homo sapiens* |  | 33.40 |
| Multidrug transporter emrE | *Escherichia coli* |  | 28.36 |
| Actin, alpha skeletal muscle | *Oryctolagus cuniculus* |  | 24.15 |
| Calcium-activated potassium channel RSK2 | *Rattus norvegicus* |  | 19.69 |
| Calmodulin | *Rattus norvegicus* |  | 19.51 |
| Ion transport protein | *Magnetococcus marinus* |  | 16.41 |
| Calmodulin | *Homo sapiens* |  | 15.33 |
| High affinity copper uptake protein 1 | *Homo sapiens* |  | 8.26 |
| 14-3-3 protein beta/alpha | *Mus musculus* |  | 7.34 |

Table S19. *Hyridella menziesii* M*-ORF* function predictions

| **Hits** | **Species** | **Position** | **Prob-**  **ability** |
| --- | --- | --- | --- |
| **HHpred** | | | |
| TIGR04294 prepilin-type processing-associated H-X9-DG domain |  | 107-111 | 99.58 |
| TIGR03304 outer membrane insertion C-terminal signal |  | 53-56 | 99.19 |
| TIGR01167 LPXTG cell wall anchor domain |  | 93-109 | 98.67 |
| TIGR03057 X-X-X-Leu-X-X-Gly heptad repeats |  | 123-137 | 97.91 |
| Apolipoprotein Apolipoprotein A1/A4/E domain |  | 170-296 | 97.84 |
| Apolipoprotein Apolipoprotein A1/A4/E domain |  | 169-296 | 97.83 |
| Apolipoprotein A-I; lipid transport; NMR | *Mus musculus* | 169-296 | 97.66 |
| TIGR03501 GlyGly-CTERM domain (rank 8) |  | 98-109 | 96.67 |
| a.24.1 Apolipoprotein (47162) SCOP seed sequence |  | 182-286 | 97.42 |
| Apolipoprotein A-I; lipid transport | *Mus musculus* | 169-289 | 97.29 |
| Apolipoprotein E **(2)** | *Homo sapiens* | 167-288 | 97.27 |
| Apolipoprotein A-I preproprotein | *Homo sapiens* | 170-296 | 97.12 |
| Y51F10.4a | *Caenorhabditis elegans* | 20-296 | 96.48 |
| Apolipoprotein E, APO-E | *Homo sapiens* | 170-296 | 96.44 |
| Y51F10.4a | *Caenorhabditis elegans* | 20-289 | 96.35 |
| Y51F10.4b | *Caenorhabditis elegans* | 17-298 | 95.84 |
| Apolipoprotein E, APO-E | *Homo sapiens* | 169-264 | 95.77 |
| abortive infection protein family | *Staphylococcus aureus* | 11-313 | 95.21 |
| TIGR00756 pentatricopeptide repeat domain |  | 71-90 | 93.94 |
| Apolipoprotein: Apolipoprotein A1/A4/E domain |  | 167-296 | 94.83 |
| Apolipoprotein: Apolipoprotein A1/A4/E domain |  | 167-295 | 94.49 |
| CG3576-PA, isoform A | *Drosophila melanogaster* | 21-171 | 93.82 |
| CG3576-PB, isoform B | *Drosophila melanogaster* | 21-171 | 93.82 |
| Apolipoprotein E, APOE4 | *Homo sapiens* | 169-253 | 93.35 |
| apolipoprotein A-V | *Mus musculus* | 168-296 | 93.30 |
| Homolog of Yeast Longevity gene family member (hyl-1) | *Caenorhabditis elegans* | 21-109 | 93.29 |
| Homolog of Yeast Longevity gene family member (hyl-2) | *Caenorhabditis elegans* | 21-174 | 93.24 |
| apolipoprotein AV | *Homo sapiens* | 168-263 | 93.12 |
| W06F12.2a | *Caenorhabditis elegans* | 20-109 | 92.95 |
| Apolipoprotein E, APOE4 | *Homo sapiens* | 171-264 | 92.62 |
| CG30394-PB, isoform B | *Drosophila melanogaster* | 20-306 | 92.56 |
| CG30394-PA, isoform A | *Drosophila melanogaster* | 20-306 | 92.56 |
| Longevity assurance homolog 4 | *Mus musculus* | 21-109 | 92.48 |
| Apolipoprotein AV | *Homo sapiens* | 169-295 | 92.06 |
| Apolipoprotein A-V | *Mus musculus* | 173-296 | 91.63 |
| Autosomal Highly Conserved Protein | *Homo sapiens* | 10-106 | 91.54 |
| Longevity assurance factor 1 (LAG1) |  | 7-109 | 91.38 |
| CG30394-PB, isoform B | *Drosophila melanogaster* | 20-128 | 90.82 |
| CG30394-PA, isoform A | *Drosophila melanogaster* | 20-128 | 90.82 |
| LAG1 longevity assurance homolog 4 | *Homo sapiens* | 21-109 | 90.77 |
| Proline-rich transmembrane protein 2 | *Homo sapiens* | 17-82 | 90.76 |
| Translocation protein 1 | *Mus musculus* | 50-275 | 90.69 |
| Integral membrane protein | *Streptomyces coelicolor* | 20-110 | 90.69 |
| Late embryogenesis abundant (plants) LEA-related |  | 171-298 | 90.44 |
| Apolipoprotein A-IV precursor | *Homo sapiens* | 169-296 | 90.32 |
| **BLASTP/PSIBLAST** | | | |
| Voltage-dependent anion channel |  | 31-117 | 1.05e-04 |
| Histone H1-like protein Hc1 |  | 213-284 | 3.78e-03 |
| Microtubule-binding protein MIP-T3 |  | 166-313 | 8.12e-06 |
| Periplasmic protein TonB links inner & outer membranes |  | 201-300 | 7.59e-04 |
| Cell division protein FtsN |  | 155-286 | 1.31e-03 |
| fam-a protein | *Plasmodium chabaudi* | 170-294 | 1e-06 |
| Cyclin related protein | *Plasmodium chabaudi* | 168-294 | 4.00E-05 |
| fam-a protein | *Plasmodium chabaudi* | 168-294 | 7e-05 |
| Choline-binding protein A **(2)** | *Streptococcus pneumoniae* | 179-238 | 7e-05 |
| Choline-binding protein A **(2)** | *Streptococcus pneumoniae* | 180-239 | 7e-05 |
| Surface protein PspC **(2)** | *Streptococcus pneumoniae* | 179-238 | 1e-04 |
| Surface protein PspC | *Streptococcus pneumoniae* | 175-309 | 1e-04 |
| LPXTG-motif cell wall anchor domain protein | *Streptococcus pneumoniae* | 179-238 | 2e-04 |
| Choline-binding protein A | *Streptococcus pneumoniae* | 182-241 | 2e-04 |
| Surface protein PspC | *Streptococcus pneumoniae* | 180-239 | 9e-04 |
| Peptidase | *Streptococcus pneumoniae* | 182-241 | 0.004 |
| **BLASTP** | | | |
| Surface protein PspC | *Streptococcus pneumoniae* | 179-238 | 0.007 |
| igA FC receptor | *Streptococcus pneumoniae* | 182-241 | 0.012 |
| Surface protein PspC | *Streptococcus pneumoniae* | 180-302 | 0.12 |
| Cyclin related protein | *Plasmodium chabaudi chabaudi* | 191-250 | 0.91 |
| **Motif Scan** | | | |
| Lysine-rich region profile |  | 168-294 | 24.274 |
| Bipartite nuclear localization signal profile |  | 170-185  236-251  269-284 | 4.000 |
| **I-TASSER** | | | |
| DNA polymerase subunit gamma-1 | *Homo sapiens* |  | 1.15 |
| Survival motor neuron protein | *Homo sapiens* |  | 1.79, 2.63 |
| SHERP | *Leishmania major* |  | 1.41, 1.40 |
| Septation ring formation regulator EZRA | *Bacillus subtilis* |  | 1.30 |
| Vascular apoptosis-inducing protein 1 | *Crotalus atrox* |  | 1.01 |
| DNA (cytosine-5)-methyltransferase 1 | *Zea mays* |  | 1.15 |
| Accumulation associated protein | *Staphylococcus sp.* |  | 1.54 |
| Tropomyosin | *Oryctolagus cuniculus* |  | 1.28 |
| Septation ring formation regulator EZRA | *Bacillus subtilis* |  | 0.943 |
| **Predict Protein** | | | |
| Protein binding |  | 1-4 |  |
| Cytoplasm |  |  |  |
| Pneumococcal surface protein C **(2)** | *Streptococcus pneumoniae* |  | 6e-32, 2e-08 |
| IgA-binding beta antigen **(4)** | *Streptococcus pneumoniae* |  | 4e-08 |
| Surface protein PcpC **(8)** | *Streptococcus pneumoniae* |  | 4e-34- 0.22 |
| Surface protein PspC **(10)** | *Streptococcus pneumoniae* |  | 4e-34- 0.22 |
| Titin **(15)** | *Mus musculus* |  | 2e-09 |
| Muscle M-line assembly protein unc-89 **(16)** | *Caenorhabditis elegans* |  | 1e-12 |
| **Atome2** | | | |
| Nucleoprotein (N-terminal domain (residues 1-74)) | *Andes virus* |  | 63.91 |
| Nuclear distribution protein NUDE-like 1 | *Homo sapiens* |  | 61.83 |
| Alpha-synuclein | *Homo sapiens* |  | 60.92 |
| Beclin-1 | *Rattus norvegicus* |  | 59.76 |
| Bud site selection protein 6 | *Saccharomyces cerevisiae* |  | 56.98 |
| Spindle and kinetochore-associated protein 3 | *Homo sapiens* |  | 48.52 |
| Actin-related protein 7 | *Saccharomyces cerevisiae* |  | 38.22 |
| Synaptobrevin 2 | *Rattus norvegicus* |  | 33.50 |
| Talin-1 (Vbs2b domain, residues 787-91) | *Mus musculus* |  | 11.89 |

Table S20. *Anodonta anatina* M*-ORF* function predictions

| **Hits** | **Species** | **Position** | **Prob-**  **ability** |
| --- | --- | --- | --- |
| **HHpred** | | | |
| TIGR03304 outer membrane insertion C-terminal signal |  | 57-64 | 99.24 |
| TIGR04294 prepilin-type processing-associated H-X9-DG domain |  | 29-32 | 99.04 |
| TIGR01167 LPXTG cell wall anchor domain |  | 22-42 | 98.89 |
| TIGR03057 X-X-X-Leu-X-X-Gly heptad repeats |  | 102-108 | 97.70 |
| TIGR03501 GlyGly-CTERM domain |  | 30-42 | 97.26 |
| TIGR00756 pentatricopeptide repeat domain |  | 102-125 | 92.98 |
| D2092.1a | *Caenorhabditis elegans* | 1-92 | 92.91 |
| D2092.1b | *Caenorhabditis elegans* | 1-123 | 87.46 |
| Y77E11A.12a | *Caenorhabditis elegans* | 2-64 | 86.20 |
| SaPosin-like Protein family member (spp-14) | *Caenorhabditis elegans* | 19-57 | 81.56 |
| Multiple C2-domains with two transmembrane regions 1 isoform L | *Homo sapiens* | 1-132 | 80.62 |
| Transmembrane protein | *Mycobacterium tuberculosis* | 1-61 | 79.32 |
| SaPosin-like Protein family member (spp-14) | *Caenorhabditis elegans* | 19-57 | 79.01 |
| F55C12.5c | *Caenorhabditis elegans* | 11-65 | 78.50 |
| Mitochondrial ribosomal protein S23 |  | 48-55 | 78.12 |
| Auxilin; four helix bundle, protein binding; NMR | *Bos taurus* | 60-112 | 77.88 |
| Multiple C2-domains with two transmembrane regions 1 isoform S | *Homo sapien* | 1-64 | 76.81 |
| Y77E11A.12b | *Caenorhabditis elegans* | 2-64 | 76.38 |
| EGF-like-domain, multiple 5 | *Mus musculus* | 23-47 | 76.05 |
| Vacuolar H ATPase family member (vha-7) | *Caenorhabditis elegans* | 21-55 | 74.03 |
| Oxidoreductase | *Arabidopsis thaliana* | 1-47 | 73.17 |
| PadR-like family transcriptional regulator | *Nostoc punctiforme* | 4-55 | 72.42 |
| C01F6.2 | *Caenorhabditis elegans* | 5-98 | 71.70 |
| CG4832-PE, isoform E | *Drosophila melanogaster* | 61-122 | 71.57 |
| "Winged helix" DNA-binding domain |  | 20-64 | 71.19 |
| GtrA |  | 8-41 | 69.48 |
| CG33146-PA | *Drosophila melanogaster* | 1-64 | 68.60 |
| ZK353.4 | *Caenorhabditis elegans* | 13-46 | 67.88 |
| Proline-rich cyclin A1-interacting protein |  | 132-184 | 67.61 |
| YbaB-like (82607) SCOP seed sequence: d1j8ba_ |  | 107-127 | 67.13 |
| paREP7 | *Pyrobaculum aerophilum* | 108-130 | 65.89 |
| Integral membrane protein | *Streptomyces coelicolor* | 28-56 | 65.74 |
| CG33171-PE, isoform E | *Drosophila melanogaster* | 19-104 | 65.66 |
| Chemokine-like factor superfamily 3 isoform a **(3)** | *Homo sapiens* | 1-82 | 65.45 |
| SPFH_like core domain of the SPFH superfamily |  | 98-134 | 65.28 |
| V-type ATPase 116 kDa subunit | *Nitrosopumilus maritimus* | 10-55 | 64.89 |
| Isopentenyl pyrophosphate isomerase | *Thermoplasma acidophilum* | 47-148 | 63.71 |
| Transcriptional regulator, PadR-like family | *Eggerthella lenta* | 32-55 | 63.05 |
| UCP004555 |  | 107-127 | 62.33 |
| Peptidase A24A prepilin type IV | *Candidatus Korarchaeum cryptofilum OPF8* | 14-67 | 62.23 |
| Metal ion binding | *Arabidopsis thaliana* | 14-52 | 61.57 |
| YbaB-like (82607) SCOP seed sequence: d1puga_ |  | 108-127 | 61.33 |
| Vacuolar H ATPase family member (vha-6) | *Caenorhabditis elegans* | 1-55 | 60.37 |
| ZK945.3 | *Caenorhabditis elegans* | 104-163 | 59.46 |
| A-type ATP synthase subunit I | *Haloferax volcanii* | 9-55 | 59.20 |
| NADH dehydrogenase I, A subunit | *Neisseria meningitidis* | 20-92 | 59.19 |
| Sensor histidine kinase | *Bartonella henselae* | 11-49 | 58.76 |
| Integral transmembrane protein 2 |  | 24-49 | 58.48 |
| Membrane-associated phospholipid phosphatase | *Methanopyrus kandleri* | 1-87 | 58.13 |
| CG31247-PB, isoform B | *Drosophila melanogaster* | 19-56 | 57.68 |
| CG31247-PC, isoform C | *Drosophila melanogaster* | 19-56 | 57.45 |
| CG31247-PA, isoform A | *Drosophila melanogaster* | 19-56 | 57.22 |
| CG31247-PD, isoform D | *Drosophila melanogaster* | 19-56 | 57.22 |
| Zinc finger, DHHC domain containing 5 | *Mus musculus* | 14-49 | 57.19 |
| Multiple C2-domains with two transmembrane regions 2 | *Homo sapiens* | 1-64 | 56.00 |
| UNCoordinated family member (unc-32) | *Caenorhabditis elegans* | 7-55 | 55.98 |
| PadR-like family transcriptional regulator | *Thermofilum pendens* | 35-70 | 55.76 |
| **BLASTP** | | | |
| Mucolipin-2 | *Echinococcus granulosus* | 97-188 | 0.22 |
| **Motif Scan** | | | |
| Lysine-rich region profile |  | 121-193 | 9.643 |
| **I-TASSER** | | | |
| HAT1-interacting factor 1 | *Saccharomyces cerevisiae* |  | 1.11 |
| HAT1-interacting factor 1 | *Saccharomyces cerevisiae* |  | 0.670 |
| Superkiller protein 3 | *Saccharomyces cerevisiae* |  | 0.565 |
| SusD-like carbohydrate binding protein | *Bacteroides vulgatus* |  | 0.564 |
| G-protein-signaling modulator 2 | *Mus musculus* |  | 0.562 |
| Partner of inscuteable | *Drosophila melanogaster* |  | 0.553 |
| 14-3-3 protein | *Cryptosporidium parvum* |  | 0.551 |
| SusD superfamily protein | *Bacteroides vulgatus* |  | 0.548 |
| **Predict Protein** | | | |
| Protein binding |  | 2,45,67,68 | 0.548 |
| Nucleus |  |  |  |
| **Atome2** | | | |
| Telomerase reverse transcriptase (TEN domain) | *Tetrahymena thermophila* |  | 68.12 |
| Guanine nucleotide exchange factor P115RHOGEF | *Homo sapiens* |  | 59.68 |
| Vinculin | *Gallus gallus* |  | 44.56 |
| Nucleoprotein | *Andes virus* |  | 41.16 |
| rRNA methyltransferase | *Streptomyces sp.* |  | 39.21 |
| Ubiquinol cytochrome c oxidoreductase | *Gallus gallus* |  | 36.83 |
| Delta-sleep-inducing peptide immunoreactive peptide | *Sus scrofa* |  | 36.46 |
| HIG1 domain family member 1B | *Homo sapiens* |  | 29.71 |
| VicH protein | *Vibrio cholera* |  | 29.49 |
| Antifreeze protein type 1 analogue | *Pseudopleuronectes americanus* |  | 28.08 |
| Replication terminator protein | *Bacillus subtilis* |  | 25.98 |
| LCoR protein | *Homo sapiens* |  | 22.26 |
| Regulatory protein MNT | *Enterobacteria phage P22* |  | 21.14 |
| Talin-1 | *Mus musculus* |  | 18.94 |
| Acetyl-delta-toxin | *Staphylococcus aureus* |  | 16.20 |

Table S21. *Venustaconcha ellipsiformis* F*-ORF* function predictions

| **Hits** | **Species** | **Position** | **Probability** |
| --- | --- | --- | --- |
| **HHpred** | | | |
| TIGR04294 prepilin-type processing-associated H-X9-DG domain |  | 11-13 | 99.30 |
| TIGR03304 outer membrane insertion C-terminal signal |  | 25-29 | 99.16 |
| TIGR01167 LPXTG cell wall anchor domain |  | 19-34 | 98.97 |
| TIGR03057 X-X-X-Leu-X-X-Gly heptad repeats |  | 71-78 | 97.97 |
| TIGR03501 GlyGly-CTERM domain |  | 28-35 | 97.29 |
| TIGR00756 pentatricopeptide repeat domain |  | 7-25 | 95.33 |
| ComGC |  | 21-38 | 92.53 |
| VAMP-5_synaptobrevin |  | 13-44 | 81.17 |
| Protein-export membrane protein | *Bartonella henselae* | 11-87 | 81.16 |
| Transducer protein Htr37 | *Haloferax volcanii* | 1-40 | 79.70 |
| Transducer protein Htr36 | *Haloferax volcanii* | 3-40 | 73.90 |
| Stage III sporulation protein AF |  | 7-39 | 70.15 |
| General secretion pathway protein H | *Nostoc punctiforme* | 5-39 | 69.69 |
| Competence protein ComGC |  | 21-38 | 69.08 |
| CG11815-PA | *Drosophila melanogaster* | 53-75 | 68.40 |
| C-type LECtin family member (clec-35) | *Caenorhabditis elegans* | 13-86 | 66.92 |
| Protein involved in cis-Golgi membrane traffic | *Saccharomyces cerevisiae* | 2-40 | 66.39 |
| F08F8.8 | *Caenorhabditis elegans* | 6-40 | 66.33 |
| Methyl-accepting chemotaxis protein | *Beggiatoa sp. PS* | 10-35 | 65.39 |
| SecD-TM1 SecD export protein N-terminal TM region |  | 12-39 | 65.33 |
| Vesicle transport through interaction with t-SNAREs 1B homolog | *Mus musculus* | 6-40 | 65.33 |
| d.24.1 Pili subunits (54523) SCOP seed sequence |  | 20-40 | 63.63 |
| Vesicle transport through interaction with t-SNAREs 1B | *Homo sapiens* | 6-40 | 63.51 |
| Vesicle-associated membrane protein 5 (myobrevin) | *Homo sapiens* | 13-45 | 61.92 |
| Vesicle transport v-snare protein | *Schizosaccharomyces pombe* | 6-40 | 60.94 |
| Y57G11C.4 | *Caenorhabditis elegans* | 2-39 | 60.92 |
| Stage III sporulation protein AF |  | 1-39 | 60.78 |
| d.24.1 Pili subunits (54523) SCOP seed sequence |  | 20-41 | 59.91 |
| Syntaxin-like t-SNARE | *Saccharomyces cerevisiae* | 6-74 | 59.63 |
| VTI11; receptor | *Arabidopsis thaliana* | 6-40 | 59.48 |
| Related to VTI1 - v-SNARE |  | 6-40 | 59.10 |
| Methyl-accepting chemotaxis protein II | *Yersinia pestis* | 4-40 | 59.10 |
| CG3279-PA | *Drosophila melanogaster* | 6-52 | 58.98 |
| OapA_N: Opacity-associated protein A N-terminal motif |  | 16-37 | 58.44 |
| VTI12; SNARE binding / receptor | *Arabidopsis thaliana* | 2-40 | 58.30 |
| SYP123; t-SNARE | *Arabidopsis thaliana* | 5-42 | 57.83 |
| Prion-like-(Q/N-rich)-domain-bearing protein family member (pqn-34) | *Caenorhabditis elegans* | 35-80 | 54.60 |
| OapA_N Opacity-associated protein A N-terminal motif |  | 16-37 | 54.42 |
| B0272.2 | *Caenorhabditis elegans* | 6-40 | 52.87 |
| DevC protein | *Nostoc punctiforme* | 1-42 | 52.59 |
| Stage III sporulation protein AF |  | 7-39 | 52.31 |
| F41F3.3 | *Caenorhabditis elegans* | 18-37 | 52.22 |
| C-type LECtin family member (clec-25) | *Caenorhabditis elegans* | 19-83 | 51.25 |
| SYP124; t-SNARE | *Arabidopsis thaliana* | 14-40 | 50.99 |
| Integral membrane sensor signal transduction histidine kinase | *Nostoc punctiforme* | 5-41 | 50.79 |
| COLlagen family member (col-144) | *Caenorhabditis elegans* | 7-42 | 50.47 |
| Methyl-accepting chemotaxis protein III | *Escherichia coli* | 10-35 | 50.16 |
| v-SNARE protein involved in Golgi transpor | *Saccharomyces cerevisiae* | 2-35 | 49.57 |
| v-SNARE | *Saccharomyces cerevisiae* | 6-40 | 48.46 |
| Flagellar M-ring protein | *Bacillus subtilis* | 2-55 | 48.18 |
| DevC protein | *Nostoc punctiforme* | 3-41 | 47.10 |
| Resistance to inhibitors of cholinesterase 3 homolog | *Homo sapiens* | 17-81 | 45.89 |
| VTI13; SNARE binding / receptor | *Arabidopsis thaliana* | 6-40 | 45.23 |
| Enzyme inhibitor/ pectinesterase | *Arabidopsis thaliana* | 4-83 | 45.14 |
| Sulfate ABC transporter | *Nostoc punctiforme* | 9-35 | 44.93 |
| TonB family protein | *Nostoc punctiforme* | 14-57 | 43.89 |
| SQuaT family member (sqt-2) | *Caenorhabditis elegans* | 14-41 | 42.86 |
| T24B1.1 | *Caenorhabditis elegans* | 8-45 | 42.65 |
| CG4780-PA | *Drosophila melanogaster* | 6-42 | 42.35 |
| COLlagen family member (col-14) | *Caenorhabditis elegans* | 1-41 | 42.28 |
| Related to SNARE protein of Golgi compartment |  | 6-39 | 42.05 |
| T10E10.5 | *Caenorhabditis elegans* | 5-42 | 42.03 |
| DevC protein | *Nostoc punctiforme* | 5-41 | 41.86 |
| FER-1-LIKE |  | 16-43 | 41.78 |
| Type IV Pilin Pak | *Pseudomonas aeruginosa* | 20-39 | 41.43 |
| Transcriptional accessory factor Tex **(2)** | *Pseudomonas aeruginosa* | 55-75 | 41.42 |
| Syntaxin-related protein required for vacuolar assembly | *Saccharomyces cerevisiae* | 5-38 | 41.06 |
| Methyl-accepting chemotaxis protein | *Bacillus subtilis* | 5-41 | 40.98 |
| CG13581-PA | *Drosophila melanogaster* | 77-89 | 40.49 |
| Diffuse panbronchiolitis critical region 1 protein | *Homo sapiens* | 14-56 | 40.32 |
| Thiol-disulfide oxidoreductase | *Bacillus subtilis* | 18-55 | 39.81 |
| Alpha-disintegrin and metalloproteinase domain 7 | *Homo sapiens* | 21-84 | 39.42 |
| Fimbrial protein | *Dichelobacter nodosus* | 20-38 | 39.16 |
| **I-TASSER** | | | |
| Glycine betaine transporter BETP | *Corynebacterium sp.* |  | 0.575 |
| Cytochrome P450 130 | *Mycobacterium tuberculosis* |  | 0.567 |
| Transcription regulator, Crp family | *Thermus thermophilus* |  | 0.565 |
| DNA topoisomerase 2 | *Saccharomyces cerevisiae* |  | 0.556 |
| Virulence-associated V antigen | *Yersinia pestis* |  | 0.554 |
| Vitamin B12 import system permease protein btuC | *Escherichia coli* |  | 0.553 |
| Phase 1 flagellin | *Salmonella enterica* |  | 0.548 |
| **Predict Protein** | | | |
| Protein binding |  | 1,8,11,15,38,40-42,44,48,50,66,70,  71,73 |  |
| Mitochondrial membrane |  |  |  |
| **Atome2** | | | |
| Hepatitis B virus X-interacting protein | *Homo sapiens* |  | 72.60 |
| Prod 1 | *Notophthalmus viridescens* |  | 68.64 |
| Tumor necrosis factor receptor | *Homo sapiens* |  | 63.21 |
| V1V2 region of HIV-1 on 1FD6 scaffold | *Human immunodef. virus 1* |  | 57.65 |
| Troponin I, cardiac muscle | *Mus musculus* |  | 38.80 |
| Bone marrow stromal antigen 2 | *Homo sapiens* |  | 35.47 |
| Fimbrial protein | *Neisseria gonorrhoeae* |  | 29.39 |
| Photosystem Q(B) protein | *Thermosynechococcus elongates* |  | 27.56 |
| Fimbrial protein | *Pseudomonas aeruginosa* |  | 23.99 |
| Fimbrial protein | *Dichelobacter nodosus* |  | 23.00 |
| Neurotoxin | *Clostridium botulinum* |  | 13.59 |

Table S22. *Quadrula quadrula* F*-ORF* function predictions

| **Hits** | **Species** | **Position** | **Probability** |
| --- | --- | --- | --- |
| **HHpred** | | | |
| TIGR04294 prepilin-type processing-associated H-X9-DG domain |  | 1-4 | 99.37 |
| TIGR03304 outer membrane insertion C-terminal signal |  | 16-20 | 99.21 |
| TIGR01167 LPXTG cell wall anchor domain |  | 10-25 | 99.02 |
| TIGR03057 X-X-X-Leu-X-X-Gly heptad repeats |  | 62-69 | 97.98 |
| TIGR03501 GlyGly-CTERM domain |  | 19-26 | 97.38 |
| TIGR00756 pentatricopeptide repeat domain |  | 63-66 | 94.93 |
| ComGC |  | 12-29 | 94.12 |
| VAMP-5_synaptobrevin |  | 4-35 | 87.41 |
| Methyl-accepting chemotaxis protein | *Beggiatoa sp. PS* | 1-26 | 84.49 |
| Protein-export membrane protein | *Bartonella henselae* | 2-77 | 83.21 |
| C-type LECtin family member (clec-35) | *Caenorhabditis elegans* | 4-77 | 79.44 |
| ComGC Competence protein ComGC |  | 12-29 | 77.26 |
| SecD-TM1 SecD export protein N-terminal TM region |  | 3-30 | 77.09 |
| Pili subunits (54523) SCOP seed sequence: d2pila_ |  | 11-30 | 73.77 |
| Vesicle-associated membrane protein 5 (myobrevin) | *Homo sapiens* | 4-36 | 73.53 |
| Methyl-accepting chemotaxis sensory transducer | *Beggiatoa sp. PS* | 1-26 | 71.68 |
| d.24.1 Pili subunits (54523) SCOP seed sequence |  | 11-32 | 70.76 |
| Methyl-accepting chemotaxis protein III | *Escherichia coli K12* | 1-26 | 70.47 |
| CG11815-PA | *Drosophila melanogaster* | 44-66 | 69.18 |
| Syntaxin-like t-SNARE | *Saccharomyces cerevisiae* | 7-65 | 68.43 |
| C-type LECtin family member (clec-25) | *Caenorhabditis elegans* | 10-74 | 67.54 |
| SYP123; t-SNARE | *Arabidopsis thaliana* | 5-33 | 67.07 |
| SYP124; t-SNARE | *Arabidopsis thaliana* | 5-31 | 66.45 |
| OapA_N: Opacity-associated protein A N-terminal motif |  | 7-28 | 65.18 |
| Sensor signal transduction histidine kinase | *Beggiatoa sp. PS* | 1-31 | 63.30 |
| Diffuse panbronchiolitis critical region 1 protein | *Homo sapiens* | 5-47 | 62.48 |
| Spore_III_AF: Stage III sporulation protein AF |  | 2-30 | 62.09 |
| OapA_N Opacity-associated protein A N-terminal motif |  | 7-28 | 61.64 |
| F41F3.3 | *Caenorhabditis elegans* | 9-28 | 59.91 |
| SQuaT family member (sqt-2) | *Caenorhabditis elegans* | 5-32 | 57.86 |
| Plasma membrane t-SNARE | *Saccharomyces cerevisiae* | 7-26 | 56.77 |
| FER-1-LIKE |  | 7-34 | 56.30 |
| Methyl-accepting chemotaxis sensory transducer | *Beggiatoa sp. PS* | 1-26 | 55.82 |
| Cytochrome c family protein | *Beggiatoa sp. PS* | 1-34 | 55.53 |
| Vesicle transport through interaction with t-SNAREs 1B | *Homo sapiens* | 3-31 | 55.19 |
| CreD |  | 3-24 | 55.15 |
| Vesicle transport through interaction with t-SNAREs 1B homolog | *Mus musculus* | 3-31 | 54.58 |
| RCR |  | 9-26 | 54.56 |
| Pilin PilE | *Neisseria meningitidis* | 1-29 | 54.48 |
| Vesicle-associated membrane protein 1 isoform 1 | *Homo sapiens* | 4-31 | 54.42 |
| Methyl-accepting chemotaxis protein II | *Yersinia pestis CO92* | 1-32 | 54.19 |
| Prion-like-(Q/N-rich)-domain-bearing protein family member (pqn-34) | *Caenorhabditis elegans* | 26-71 | 54.00 |
| C-type LECtin family member (clec-32) | *Caenorhabditis elegans* | 3-74 | 53.81 |
| Type IV Pilin Pak | *Pseudomonas aeruginosa* | 11-30 | 53.69 |
| F08F8.8 | *Caenorhabditis elegans* | 3-31 | 53.66 |
| Stage III sporulation protein AF |  | 2-30 | 53.25 |
| Stage III sporulation protein AG |  | 7-25 | 53.24 |
| Sensor protein | *Nostoc punctiforme* | 3-32 | 52.91 |
| Protein involved in cis-Golgi membrane traffic; v-SNARE | *Saccharomyces cerevisiae* | 3-31 | 52.91 |
| COLlagen family member (col-176) | *Caenorhabditis elegans* | 3-33 | 52.78 |
| Syntaxin-related protein required for vacuolar assembly | *Saccharomyces cerevisiae* | 5-31 | 52.32 |
| C-type LECtin family member (clec-38) | *Caenorhabditis elegans* | 10-80 | 52.29 |
| TonB family protein | *Nostoc punctiforme* | 5-48 | 51.68 |
| Extracellular solute-binding protein | *Thermofilum pendens* | 3-26 | 51.66 |
| USE1-like protein |  | 9-26 | 51.50 |
| Thiol-disulfide oxidoreductase | *Bacillus subtilis* | 9-46 | 51.47 |
| Fimbrial protein (cell adhesion) | *Dichelobacter nodosus* | 11-29 | 51.31 |
| C-type LECtin family member (clec-27) | *Caenorhabditis elegans* | 10-74 | 51.13 |
| Enzyme inhibitor/ pectinesterase | *Arabidopsis thaliana* | 4-74 | 51.02 |
| Resistance to inhibitors of cholinesterase 3 homolog | *Homo sapiens* | 8-72 | 50.86 |
| Sialidase | *Haloferax volcanii* | 3-26 | 50.72 |
| Syntaxin 12 | *Mus musculus* | 5-30 | 50.68 |
| Use1 Membrane fusion protein Use1 |  | 9-26 | 50.43 |
| Target membrane receptor (t-SNARE) | *Saccharomyces cerevisiae* | 7-26 | 50.43 |
| CG3279-PA | *Drosophila melanogaster* | 3-31 | 50.13 |
| Fimbrial protein | *Dichelobacter nodosus* | 11-29 | 49.97 |
| Plasma membrane t-SNARE | *Saccharomyces cerevisiae* | 6-26 | 49.94 |
| SYP111; t-SNARE | *Arabidopsis thaliana* | 5-34 | 49.48 |
| Sensor protein | *Nostoc punctiforme* | 3-32 | 49.45 |
| Y57G11C.4 | *Caenorhabditis elegans* | 3-30 | 48.87 |
| VTI11; receptor | *Arabidopsis thaliana* | 3-31 | 48.51 |
| Alpha-disintegrin and metalloproteinase domain 7 | *Homo sapiens* | 12-75 | 48.44 |
| Use1: Membrane fusion protein Use1 |  | 9-26 | 48.26 |
| SYP21; t-SNARE | *Arabidopsis thaliana* | 5-30 | 47.91 |
| 4HB_MCP_1: Four helix bundle sensory module for signal transduction |  | 7-26 | 47.71 |
| CG31136-PA | *Drosophila melanogaster* | 7-32 | 47.53 |
| **PSIBLAST** | | | |
| Preprotein translocase subunit SecG | *Bergeyella zoohelcum* | 2-80 | 2.00e-04 |
| Leucine rich repeat protein **(2)** | *Leptospira kirschneri* | 19-63 | 3.00e-04, |
| Magnesium transporter MgtE **(2)** | *Thermus oshimai* | 7-49 | 7.00e-04 |
| Mg2+ transporter MgtE | *Thermus oshimai* | 7-49 | 7.00e-04 |
| Histidine kinase | *Paenibacillus larvae* | 1-26 | 8.00e-04 |
| Peptidase M15B and M15C DD-carboxypeptidase VanY/endolysin | *Paenibacillus sp. JDR-2* | 13-74 | 9.00e-04 |
| Histidine kinase | *Paenibacillus larvae* | 1-26 | 0.001 |
| Peptidase M15 | *Paenibacillus sp. JDR-2* | 13-74 | 0.001 |
| Calcium/proton exchanger **(3)** | *Cryptococcus gattii* | 26-78 | 0.001 |
| Calcium ion transporter | *Cryptococcus gattii* | 26-78 | 0.001 |
| Diaminopimelate epimerase | *Pseudomonas sp. RIT357* | 29-78 | 0.001 |
| Amino acid transporter | *Olleya marilimosa* | 14-49 | 0.002 |
| Sulfate transporter | *Bacillus cereus* | 3-37 | 0.002 |
| Serine/threonine protein kinase | *Vibrio harveyi* | 6-67 | 0.002 |
| Na(+)/H(+) antiporter NhaA | *Salinispora pacifica* | 13-75 | 0.002 |
| C4-dicarboxylate ABC transporter | *Chelatococcus sp. GW1* | 7-38 | 0.002 |
| Poly(glycerophosphate chain) D-alanine transfer protein | *Streptococcus parauberis* | 7-61 | 0.002 |
| D-alanyl-lipoteichoic acid biosynthesis protein DltD | *Streptococcus parauberis* | 7-61 | 0.002 |
| Sodium:proton antiporter | *Salinispora pacifica* | 13-75 | 0.002 |
| ATP-dependent DNA helicase RecQ | *Rhodopirellula europaea* | 37-76 | 0.002 |
| Transporter, MotA/TolQ/ExbB proton channel family protein | *Prevotella pleuritidis* | 11-78 | 0.003 |
| Sulfate transporter | *Bacillus cereus* | 3-40 | 0.003 |
| Bacterial membrane protein YfhO | *Microvirga lotononidis* | 5-73 | 0.003 |
| MULTISPECIES: C4-dicarboxylate ABC transporter | *Rhizobium* | 7-38 | 0.003 |
| XRE family transcriptional regulator | *Cyanothece sp. PCC 8801* | 23-49 | 0.003 |
| Leucine-rich repeat and death domain-containing protein | *Heterocephalus glaber* | 19-59 | 0.003 |
| Serine/threonine protein kinase **(2)** | *Vibrio harveyi* | 6-67 | 0.003 |
| Membrane protein, partial | *Streptomyces sp.* | 6-34 | 0.004 |
| Doublesex-and mab-3-related transcription factor 3 | *Strongyloides ratti* | 35-66 | 0.004 |
| Membrane protein, partial | *Streptomyces xanthophaeus* | 6-34 | 0.004 |
| General secretion pathway protein G | *Gallaecimonas xiamenensis* | 12-57 | 0.004 |
| Nicotinate (nicotinamide) nucleotide adenylyltransferase | *Cryptococcus gattii* | 27-74 | 0.004 |
| Bicarbonate transporter BicA | *Prochlorococcus marinus* | 10-61 | 0.004 |
| CoA-binding protein | *Halosarcina pallida* | 34-74 | 0.004 |
| Antisigma-factor antagonist, STAS | *Bacillus cereus Rock3-28* | 3-40 | 0.005 |
| Magnesium transporter MgtE | *Thermus yunnanensis* | 10-49 | 0.005 |
| **I-TASSER** | | | |
| A-type ATP synthase subunit E | *Methanocaldococcus jannaschii* |  | 1.18 |
| Fumarase C | *Escherichia coli* |  | 0.548 |
| Fumarate hydratase | *Homo sapiens* |  | 0.543 |
| Deoxyguanosinetriphosphate triphosphohydrolase | *Escherichia coli K-1* |  | 0.543 |
| Fumarate hydratase class II | *Rickettsia prowazekii* |  | 0.542 |
| Adenylosuccinate lyase | *Staphylococcus aureus* |  | 0.538 |
| Fumarate lyase | *Chelativorans sp. BNC1* |  | 0.538 |
| Fumarase Fum | *Mycobacterium marinum M* |  | 0.537 |
| Adenylosuccinate lyase | *Bacillus anthracis* |  | 0.537 |
| **Predict Protein** | | | |
| Protien binding |  | 1,13,39,40, 59,65,85 |  |
| Mitochondrial membrane |  |  |  |
| **Atome2** | | | |
| HIV-1 matrix protein **(2)** | *Human immunodef. virus 1* |  | 83.13 |
| Obscurin-like protein 1 | *Homo sapiens* |  | 41.74 |
| Photosystem Q(B) protein **(2)** | *Thermosynechococcus elongatus* |  | 37.59 |
| Photosystem II reaction center protein T | *Mastigocladus laminosus* |  | 27.60 |
| Sec-independent protein translocase protein tatAd | *Bacillus subtilis* |  | 27.54 |
| Fimbrial protein | *Neisseria gonorrhoeae* |  | 26.93 |
| Fimbrial protein | *Pseudomonas aeruginosa* |  | 23.40 |
| Vesicle-associated membrane protein 2 | *Rattus norvegicus* |  | 23.05 |
| Fimbrial protein | *Dichelobacter nodosus* |  | 22.81 |
| Defensin | *Caretta caretta* |  | 16.71 |

Table S23. *Pyganodon grandis* F*-ORF* function predictions

| **Hits** | **Species** | **Position** | **Probability** |
| --- | --- | --- | --- |
| **HHpred** | | | |
| TIGR01167 LPXTG cell wall anchor domain |  | 62-67 | 99.45 |
| TIGR03304 outer membrane insertion C-terminal signal |  | 1-6 | 99.34 |
| TIGR04294 prepilin-type processing-associated H-X9-DG domain |  | 26-29 | 99.27 |
| TIGR03057 X-X-X-Leu-X-X-Gly heptad repeats |  | 54-61 | 97.97 |
| TIGR03501 GlyGly-CTERM domain |  | 16-26 | 97.22 |
| TIGR00756 pentatricopeptide repeat domain |  | 46-53 | 94.27 |
| CG7685-PA | *Drosophila melanogaster* | 7-34 | 92.74 |
| Intra-Golgi v-SNARE | *Saccharomyces cerevisiae* | 2-34 | 89.49 |
| TMEM156: TMEM156 protein family |  | 9-35 | 76.75 |
| Conserved inner membrane protein | *Escherichia coli* | 5-37 | 75.88 |
| SrtB |  | 4-42 | 75.38 |
| LptF_YjgP LPS export ABC transporter permease LptF |  | 7-37 | 74.47 |
| GOLGI SNARE BET1-RELATED |  | 11-32 | 69.38 |
| Ceramidase |  | 11-52 | 69.27 |
| CG13969-PA | *Drosophila melanogaster* | 11-52 | 68.78 |
| Sensor histidine kinase | *Streptococcus pneumoniae* | 9-48 | 64.48 |
| LPS export ABC transporter permease LptG |  | 5-37 | 64.01 |
| Sensory box histidine kinase PhoR | *Staphylococcus aureus* | 9-44 | 63.97 |
| Nitric oxide reductase subunit C | *Pseudomonas aeruginosa* | 5-30 | 61.75 |
| Saliv_gland_allergen_Aed3 |  | 10-27 | 60.13 |
| Essential SNARE protein localized to the ER | *Saccharomyces cerevisiae* | 13-34 | 60.06 |
| T27F7.3a | *Caenorhabditis elegans* | 10-44 | 59.65 |
| CG11020-PA, isoform A | *Drosophila melanogaster* | 5-46 | 57.90 |
| Sterol reductase/lamin b receptor |  | 27-55 | 57.55 |
| Alkaline ceramidase 2 | *Mus musculus* | 11-52 | 56.62 |
| Dipeptide transport permease | *Pyrobaculum aerophilum* | 2-34 | 56.60 |
| GRP: Glycine rich protein family |  | 14-34 | 56.22 |
| Permease YjgP/YjgQ family protein | *Nostoc punctiforme* | 5-37 | 56.02 |
| Osm-9 & capsaicin receptor-related family (ocr-4) | *Caenorhabditis elegans* | 5-70 | 55.92 |
| ATBS14A; protein transporter | *Arabidopsis thaliana* | 11-32 | 55.52 |
| W02F12.2 | *Caenorhabditis elegans* | 11-61 | 54.80 |
| Related to YPC1 - Alkaline ceramidase |  | 11-52 | 53.56 |
| Human EMeRin homolog family member (emr-1) | *Caenorhabditis elegans* | 13-31 | 53.29 |
| GDSL family lipase | *Nitrosopumilus maritimus* | 1-38 | 52.36 |
| Alkaline ceramidase that also has reverse (CoA-independent) ceramide synthase function | *Saccharomyces cerevisiae* | 7-52 | 52.01 |
| Vesicle-associate membrane protein-associated protein |  | 12-34 | 51.58 |
| SVM protein signal sequence |  | 10-31 | 51.26 |
| GRP Glycine rich protein family |  | 13-34 | 51.08 |
| CbiN ABC-type cobalt transport system, periplasmic component |  | 9-44 | 49.52 |
| Peptidoglycan-associated lipoprotein Pal | *Yersinia pestis* | 8-27 | 47.95 |
| Lipoprotein required for capsular polysaccharide translocation through the outer membrane | *Escherichia coli* | 9-27 | 47.20 |
| Retinoblastoma-associated protein | *Homo sapiens* | 37-54 | 47.14 |
| Galactose-3-O-sulfotransferase 3 | *Homo sapiens* | 4-63 | 46.93 |
| H/K_exch_ATPase_C |  | 9-38 | 46.76 |
| Phytoceramidase, alkaline | *Homo sapiens* | 2-52 | 46.21 |
| DumPY: shorter than wild-type family member (dpy-5) | *Caenorhabditis elegans* | 9-49 | 46.06 |
| Y41C4A.19 | *Caenorhabditis elegans* | 2-54 | 45.83 |
| Secreted protein | *Beggiatoa sp. PS* | 9-57 | 45.68 |
| Protein transporter | *Arabidopsis thaliana* | 11-34 | 44.80 |
| Extracellular solute-binding protein | *Nostoc punctiforme* | 2-31 | 43.64 |
| **I-TASSER** | | | |
| Human cyclin B1 | *Homo sapiens* |  | 0.520 |
| G1/S-specific cyclin-D1 | *Homo sapiens* |  | 0.518 |
| V-cyclin | *Human herpesvirus 8* |  | 0.517 |
| G1/S-specific cyclin E1 | *Homo sapiens* |  | 0.517 |
| Cell division protein kinase 4 | *Homo sapiens* |  | 0.516 |
| Cell division protein kinase 2 | *Homo sapiens* |  | 0.515 |
| G2/mitotic-specific cyclin-B1 | *Homo sapiens* |  | 0.513 |
| Cyclin-C | *Homo sapiens* |  | 0.513 |
| **Predict Protein** | | | |
| Protein binding |  | 6,9,28,29,31,  33,35,38,40, 42,57,59,61,62,64 |  |
| Mitochondrial membrane |  |  |  |
| **Atome2** | | | |
| Colicin D (colicin D catalytic domain) | *Escherichia coli* |  | 87.77 |
| 14 kDa phosphohistidine phosphatase | *Homo sapiens* |  | 83.70 |
| Thrombin | *Homo sapiens* |  | 67.65 |
| Human beta2-Glycoprotein I | *Homo sapiens* |  | 66.28 |
| Calcium-gated potassium channel mthK | *Methanothermobacter thermautotrophicus* |  | 48.14 |
| Photosynthetic reaction center C subunit | *Thermochromatium tepidum* |  | 38.65 |
| CREB-binding protein | *Mus musculus* |  | 37.60 |
| Transcription factor Dp-1 | *Homo sapiens* |  | 36.50 |
| CPAP | *Danio rerio* |  | 27.81 |
| Vesicle-associated membrane protein 2 | *Rattus norvegicus* |  | 24.76 |
| Protein translocase subunit secA | *Bacillus subtilis* |  | 24.71 |
| Transient receptor potential cation channel subfamily V member 1 | *Rattus norvegicus* |  | 20.75 |
| Antibody fab fragment light chain | *Mus musculus* |  |  |

Table S24. *Inversidens japanensis* F*-ORF* function predictions

| **Hits** | **Species** | **Position** | **Probability** |
| --- | --- | --- | --- |
| **HHpred** | | | |
| TIGR01167 LPXTG cell wall anchor domain |  | 1-15 | 99.44 |
| TIGR03304 outer membrane insertion C-terminal signal |  | 3-5 | 99.36 |
| TIGR04294 prepilin-type processing-associated H-X9-DG domain |  | 13-15 | 99.23 |
| X-X-X-Leu-X-X-Gly heptad repeats |  | 57-65 | 97.91 |
| TIGR03501 GlyGly-CTERM domain |  | 2-13 | 97.58 |
| TIGR00756 pentatricopeptide repeat domain |  | 14-18 | 94.24 |
| L-lactate permease-related protein | *Neisseria meningitidis* | 1-31 | 81.61 |
| golgi phosphoprotein 4 | *Homo sapiens* | 4-18 | 80.40 |
| H/K_exch_ATPase_C |  | 3-15 | 80.09 |
| Md_memb_hyd |  | 5-24 | 74.97 |
| Cell division protein ZipA | *Yersinia pestis* | 2-18 | 72.32 |
| Cell division protein ZipA | *Escherichia coli* | 2-17 | 71.55 |
| Cell division protein ZipA | *Pseudomonas aeruginosa* | 2-18 | 70.81 |
| C-type LECtin family member (clec-62) | *Caenorhabditis elegans* | 1-19 | 70.72 |
| TGF-beta-activated kinase 1 and MAP3K7-binding PR | *Homo sapiens* | 40-59 | 69.82 |
| ABI gene family, member 3 (NESH) binding protein | *Homo sapiens* | 1-14 | 69.57 |
| Asialoglycoprotein receptor 1 | *Homo sapiens* | 1-14 | 69.16 |
| Melanocortin 3 receptor | *Homo sapiens* | 16-61 | 67.54 |
| Fibronectin-binding_protein_I_partial TQXA domain |  | 10-22 | 66.79 |
| T10G3.1 | *Caenorhabditis elegans* | 2-13 | 65.01 |
| Y111B2A.26 | *Caenorhabditis elegans* | 17-51 | 64.36 |
| Alpha-1,4-N-acetylglucosaminyltransferase | *Homo sapiens* | 1-15 | 61.44 |
| TIGR03778 VPDSG-CTERM protein sorting domain |  | 56-61 | 65.10 |
| CG33206-PB, isoform B | *Drosophila melanogaster* | 7-60 | 60.26 |
| Y41G9A.4a | *Caenorhabditis elegans* | 1-32 | 60.13 |
| Thrombospondin type 3 repeat-containing protein | *Nitrosopumilus maritimus* | 2-27 | 60.12 |
| Cell division protein ZipA |  | 1-21 | 59.36 |
| Neuropeptide-Like Protein family member (nlp-16) | *Caenorhabditis elegans* | 1-15 | 59.23 |
| Peptidyl-prolyl cis-trans isomerase | *Neisseria meningitidis* | 3-60 | 58.90 |
| CG12522-PA | *Drosophila melanogaster* | 2-51 | 58.86 |
| Cell division protein | *Yersinia pestis CO92* | 1-51 | 57.29 |
| TMEM52: Transmembrane 52 |  | 2-13 | 56.98 |
| ZK1010.5 | *Caenorhabditis elegans* | 2-16 | 56.32 |
| C-type lectin, superfamily member 14 isoform 2 | *Homo sapiens* | 2-18 | 56.21 |
| KdpC K+-transporting ATPase, c chain |  | 1-15 | 55.34 |
| Submaxillary gland androgen regulated protein 1 | *Mus musculus* | 1-11 | 54.45 |
| Transmembrane protein | *Mycobacterium tuberculosis* | 1-40 | 54.33 |
| Potassium-transporting ATPase subunit C; Reviewed |  | 1-15 | 54.00 |
| Alpha 1B-glycoprotein | *Homo sapiens* | 1-33 | 53.56 |
| KdpC: K+-transporting ATPase, c chain |  | 3-15 | 53.51 |
| CG33706-PA, isoform A | *Drosophila melanogaster* | 2-14 | 52.88 |
| Potassium-transporting ATPase C chain K+ |  | 1-15 | 52.85 |
| Potassium-transporting ATPase subunit C | *Nostoc punctiforme* | 3-15 | 52.73 |
| F13G3.12 | *Caenorhabditis elegans* | 13-19 | 52.66 |
| CG11709-PA | *Drosophila melanogaster* | 1-55 | 52.32 |
| Potassium-transporting ATPase subunit C | *Escherichia coli* | 2-15 | 51.89 |
| Potassium-transporting ATPase subunit C | *Nostoc punctiforme* | 2-15 | 51.32 |
| Potassium-transporting ATPase subunit C | *Mycobacterium tuberculosis* | 1-15 | 51.31 |
| Golgi phosphoprotein 4 | *Mus musculus* | 4-18 | 51.08 |
| ROK family transcriptional regulator | *Streptomyces coelicolor* | 2-35 | 50.72 |
| Cell envelope integrity inner membrane protein TolA | *Yersinia pestis* | 3-26 | 50.33 |
| Chymotrypsinogen B2 | *Homo sapiens* | 1-29 | 50.13 |
| Macrophage galactose N-acetyl-galactosamine specific lectin 2 | *Mus musculus* | 1-17 | 49.30 |
| CG9928-PA | *Drosophila melanogaster* | 1-15 | 49.29 |
| Potassium-transporting ATPase subunit C | *Yersinia pestis* | 2-15 | 48.97 |
| Ribonuclease, RNase A family, 2 (liver, eosinophil-derived neurotoxin) | *Homo sapiens* | 5-25 | 48.15 |
| K+-transporting ATPase, C subunit | *Staphylococcus aureus* | 2-15 | 47.95 |
| R09D1.5 | *Caenorhabditis elegans* | 2-19 | 47.79 |
| CD8 ALPHA CHAIN |  | 2-13 | 47.69 |
| P-type ATPase | *Frankia alni* | 2-15 | 47.63 |
| GRP: Glycine rich protein family |  | 1-52 | 47.53 |
| Outer membrane efflux protein | *Nostoc punctiforme* | 1-50 | 47.40 |
| Potassium-transporting ATPase subunit C | *Pseudomonas aeruginosa* | 2-15 | 47.35 |
| Cation transport system component | *Streptomyces coelicolor* | 3-15 | 46.51 |
| **I-TASSER** | | | |
| MSin3A-binding protein | *Mus musculus* |  | 1.03 |
| **Predict Protein** | | | |
| Protein binding |  | 4,15-19, 28,30,33, 35,45,47, 49,53,54, 56,58,62,65 |  |
| Secreted |  |  |  |
| **Atome2** | | | |
| Carboxypeptidase A2 | *Homo sapiens* |  | 69.57 |
| TraF protein | *Escherichia coli* |  | 61.48 |
| Nonstructural RNA-binding protien 34 | *Simian rotavirus* |  | 28.60-48.04 |
| Nucleoporin | *Mus musculus* |  | 47.00 |
| Dolichyl-diphosphooligosaccharide--protein glycosyltransferase subunit 4 | *Homo sapiens* |  | 41.53 |
| Chromo domain-containing protein 1 | *Saccharomyces cerevisiae* |  | 38.89 |
| Bone marrow stromal antigen 2 | *Homo sapiens* |  | 37.80 |
| Cytochrome b6 **(3)** | *Mastigocladus laminosus* |  | 29.46-34.43 |
| Presenilin-1 | *Homo sapiens* |  | 32.35 |
| Polyadenylate-binding protein 1 | *Homo sapiens* |  | 28.20 |
| Mitogen-activated protein kinase 14 | *Mus musculus* |  | 25.10 |

Table S25. *Utterbackia peninsularis* F*-ORF* function predictions

| **Hits** | **Species** | **Position** | **Probability** |
| --- | --- | --- | --- |
| **HHpred** | | | |
| TIGR01167 LPXTG cell wall anchor domain |  | 47-52 | 99.47 |
| TIGR04294 prepilin-type processing-associated H-X9-DG domain |  | 18-21 | 99.34 |
| TIGR03304 outer membrane insertion C-terminal signal |  | 27-28 | 99.28 |
| TIGR03057 X-X-X-Leu-X-X-Gly heptad repeats |  | 39-46 | 98.08 |
| TIGR03501 GlyGly-CTERM domain |  | 8-18 | 97.39 |
| TIGR00756 pentatricopeptide repeat domain |  | 31-38 | 94.79 |
| CG7685-PA | *Drosophila melanogaster* | 2-34 | 90.11 |
| CG11786-PA | *Drosophila melanogaster* | 1-33 | 83.58 |
| CbiN ABC-type cobalt transport system, periplasmic component |  | 1-29 | 79.63 |
| Human EMeRin homolog family member (emr-1) | *Caenorhabditis elegans* | 5-24 | 74.62 |
| CG13969-PA | *Drosophila melanogaster* | 3-37 | 74.59 |
| Ceramidase |  | 3-37 | 67.66 |
| Saliv_gland_allergen_Aed3 |  | 2-19 | 66.83 |
| GRP: Glycine rich protein family |  | 6-23 | 64.89 |
| Lipoprotein required for capsular polysaccharide translocation through the outer membrane | *Escherichia coli* | 1-19 | 62.25 |
| Intra-Golgi v-SNARE | *Saccharomyces cerevisiae* | 3-23 | 57.13 |
| Syntaxin-like t-SNARE | *Saccharomyces cerevisiae* | 8-58 | 56.27 |
| Syntaxin 5 | *Mus musculus* | 3-25 | 56.06 |
| Cysteine-type endopeptidase/ cysteine-type peptidase | *Arabidopsis thaliana* | 2-41 | 55.67 |
| GRP Glycine rich protein family |  | 5-23 | 55.06 |
| T27F7.3a | *Caenorhabditis elegans* | 2-29 | 54.72 |
| RCR |  | 8-23 | 54.67 |
| W02F12.2 | *Caenorhabditis elegans* | 3-46 | 54.24 |
| CG4214-PA, isoform A | *Drosophila melanogaster* | 6-25 | 54.12 |
| CG4214-PB, isoform B | *Drosophila melanogaster* | 6-25 | 54.12 |
| SYP31; t-SNARE | *Arabidopsis thaliana* | 3-24 | 53.28 |
| Retinoblastoma-associated protein | *Homo sapiens* | 22-39 | 53.01 |
| Alkaline ceramidase 2 | *Mus musculus* | 3-37 | 52.35 |
| MORN repeat protein | *Beggiatoa sp. PS* | 1-21 | 52.26 |
| Cancer susceptibility candidate 4 isoform 1 | *Mus musculus* | 4-32 | 52.12 |
| Golgi phosphoprotein 2 | *Homo sapiens* | 1-32 | 52.03 |
| Target membrane receptor (t-SNARE) | *Saccharomyces cerevisiae* | 3-24 | 51.69 |
| Sensor histidine kinase | *Streptococcus pneumoniae* | 1-35 | 51.24 |
| SYNtaxin family member (syn-3) | *Caenorhabditis elegans* | 8-25 | 51.20 |
| C46H11.8 | *Caenorhabditis elegans* | 6-20 | 50.34 |
| Golgi SNARE BET1-related |  | 3-23 | 49.96 |
| Sensory box histidine kinase PhoR | *Staphylococcus aureus* | 1-35 | 49.94 |
| Related to YPC1 - Alkaline ceramidase |  | 3-37 | 49.81 |
| P53-induced protein related |  | 4-33 | 48.53 |
| LCR32 | *Arabidopsis thaliana* | 1-18 | 48.29 |
| Sensor protein | *Nostoc punctiforme* | 2-35 | 47.97 |
| Cancer susceptibility candidate 4 isoform b | *Homo sapiens* | 4-32 | 47.39 |
| SVM protein signal sequence |  | 2-21 | 46.80 |
| Cancer susceptibility candidate 4 isoform a | *Homo sapiens* | 4-32 | 46.01 |
| Rhodanese-like protein | *Beggiatoa sp. PS* | 2-21 | 45.74 |
| LCR9 | *Arabidopsis thaliana* | 1-18 | 45.22 |
| Cell wall structural complex MreBCD transmembrane component MreC | *Escherichia coli* | 2-34 | 45.03 |
| Cancer susceptibility candidate 4 isoform 2 | *Mus musculus* | 4-32 | 44.69 |
| Alkaline ceramidase that also has reverse (CoA-independent) ceramide synthase activity | *Saccharomyces cerevisiae* | 3-37 | 44.61 |
| T01B10.5 | *Caenorhabditis elegans* | 9-67 | 44.41 |
| Pectinesterase/pectinesterase inhibitor | *Arabidopsis thaliana* | 2-21 | 44.24 |
| Conserved inner membrane protein | *Escherichia coli* | 1-32 | 44.17 |
| Y116A8C.44 | *Caenorhabditis elegans* | 10-21 | 44.15 |
| SYP32; t-SNARE | *Arabidopsis thaliana* | 3-25 | 44.03 |
| v-SNARE protein involved in Golgi transport, homolog of the mammalian protein GOS-28/GS28 | *Saccharomyces cerevisiae* | 7-29 | 43.63 |
| T19H12.3 | *Caenorhabditis elegans* | 6-21 | 43.56 |
| F08E10.7 | *Caenorhabditis elegans* | 6-34 | 43.53 |
| Alpha/beta hydrolase superfamily protein | *Lactobacillus casei* | 1-30 | 43.32 |
| CG4716-PB, isoform B | *Drosophila melanogaster* | 10-35 | 43.04 |
| F10B5.9 | *Caenorhabditis elegans* | 9-33 | 42.75 |
| Leukocyte surface antigen CD47 |  | 2-34 | 42.10 |
| COLlagen family member (col-102) | *Caenorhabditis elegans* | 4-39 | 41.52 |
| Diguanylate cyclase | *Nostoc punctiforme* | 2-35 | 41.51 |
| F58G1.5 | *Caenorhabditis elegans* | 8-55 | 41.39 |
| **Predict Protein** | | | |
| Protein binding |  | 1,2,41,50 |  |
| Mitochondrial membrane |  |  |  |
| **Atome2** | | | |
| Thrombin | *Homo sapiens* |  | 86.79 |
| Spindle pole body component SPC42 | *Saccharomyces cerevisiae* |  | 67.12 |
| Antitoxin RelB3 | *Methanocaldococcus jannaschii* |  | 62.24 |
| Antifreeze peptide SS-3 | *Myoxocephalus scorpius* |  | 55.05 |
| CREB-binding protein | *Mus musculus* |  | 50.10 |
| Antifreeze peptide SS-3 | *Myoxocephalus scorpius* |  | 45.83 |
| Oligomerization | *Homo sapiens* |  | 45.03 |
| BM2 protein | *Influenza B virus* |  | 42.29 |
| Importin subunit alpha-2 | *Mus musculus* |  | 41.49 |
| Protein transport protein SEC23 | *Saccharomyces cerevisiae* |  | 41.12 |
| CPAP | *Danio rerio* |  | 40.65 |
| Transcription factor Dp-1 | *Homo sapiens* |  | 36.18 |
| Protein translocase subunit secA | *Bacillus subtilis* |  | 23.61 |
| Argenine attenuator peptide | *Neurospora crassa* |  | 23.04 |
| Beta-hemolysin | *Staphylococcus aureus* |  | 13.11 |

Table S26. *Solenaia carinatus* F*-ORF* function predictions

| **Hits** | **Species** | **Position** | **Probability** |
| --- | --- | --- | --- |
| **HHpred** | | | |
| TIGR04294 prepilin-type processing-associated H-X9-DG domain |  | 2-9 | 99.37 |
| TIGR03304 outer membrane insertion C-terminal signal |  | 62-63 | 99.06 |
| TIGR01167 LPXTG cell wall anchor domain |  | 4-22 | 98.91 |
| TIGR03057 X-X-X-Leu-X-X-Gly heptad repeats |  | 4-7 | 97.69 |
| TIGR03501 GlyGly-CTERM domain |  | 7-17 | 96.90 |
| TIGR00756 pentatricopeptide repeat domain |  | 16-23 | 93.28 |
| 5-hydroxytryptamine (serotonin) receptor 1D | *Homo sapiens* | 33-70 | 92.61 |
| Light-harvesting complex subunits | *Rhodoblastus acidophilus* | 8-26 | 87.30, 87.07 |
| Light-harvesting complex subunits (56918) SCOP |  | 4-26 | 83.17 |
| Prenylated RAB acceptor 1-related |  | 6-26 | 79.07 |
| Light-harvesting protein B-800/850 |  | 5-26 | 74.63 |
| Integrin, beta-like 1 | *Mus musculus* | 1-28 | 73.53 |
| Light-harvesting complex subunits | *Rhodoblastus acidophilus* | 8-25 | 72.83 |
| LH1 beta polypeptide; photosynthesis |  | 5-26 | 70.95 |
| Light-harvesting complex subunits | *Rhodoblastus acidophilus* | 8-25 | 70.88 |
| P-loop containing nucleoside triphosphate hydrolases (52540) SCOP seed sequence: d1qhla_ |  | 48-56 | 70.54 |
| LH II, B800/850, light harvesting complex II |  | 5-26 | 69.61 |
| Rab acceptor 1 | *Homo sapiens* | 6-26 | 69.24 |
| Membrane protein | *Beggiatoa sp. PS* | 34-54 | 68.30 |
| Rab acceptor 1 | *Mus musculus* | 6-26 | 67.03 |
| Transmembrane protein HTP-1 related |  | 2-21 | 66.89 |
| CG1418-PA | *Drosophila melanogaster* | 6-26 | 65.31 |
| Dienelactone hydrolase | *Nostoc punctiforme* | 8-66 | 57.49 |
| PRA1 PRA1 family protein |  | 6-26 | 57.44 |
| ZK896.1 | *Caenorhabditis elegans* | 4-47 | 57.43 |
| Sterol carrier protein 2 isoform 3 precursor | *Homo sapiens* | 23-31 | 56.70 |
| MPI7 | *Arabidopsis thaliana* | 6-26 | 56.39 |
| Syntaxin-like t-SNARE | *Saccharomyces cerevisiae* | 8-66 | 56.24 |
| Protein localized to COPII vesicles | *Saccharomyces cerevisiae* | 6-26 | 55.8 |
| LH-1, light-harvesting protein B-880, beta chain | *Rhodospirillum rubrum* | 5-26 | 55.18 |
| Magnesium transporter | *Synechococcus sp.* | 9-28 | 55.18 |
| Flagellar motor protein MotS |  | 11-23 | 55.17 |
| CG10031-PA | *Drosophila melanogaster* | 1-21 | 54.86 |
| Integrin, beta-like 1 (with EGF-like repeat domains) | *Homo sapiens* | 1-24 | 54.45 |
| Phosphatidylserine decarboxylase | *Methanopyrus kandleri* | 1-26 | 53.64 |
| Light-harvesting protein B-880, beta chain |  | 5-26 | 53.58 |
| CG6339-PA | *Drosophila melanogaster* | 48-56 | 52.81 |
| Cell surface glycoprotein | *Methanosarcina mazei* | 1-63 | 51.14 |
| Excinuclease ATPase subunit | *Beggiatoa sp. PS* | 48-56 | 50.17 |
| Dopamine receptor D1A | *Mus musculus* | 1-26 | 50.08 |
| RADiation sensitivity abnormal/yeast RAD-related family member (rad-50) | *Caenorhabditis elegans* | 34-56 | 49.33 |
| Light-harvesting protein B-880, beta chain | *Rhodospirillum rubrum* | 5-26 | 49.09 |
| P-loop containing nucleoside triphosphate hydrolases (52540) SCOP |  | 48-56 | 48.83 |
| RAD50; ATP binding / nuclease/ zinc ion binding | *Arabidopsis thaliana* | 48-56 | 48.65 |
| ABC transporter ATP-binding protein | *Beggiatoa sp. PS* | 48-56 | 48.37 |
| Oligosaccharyltransferase subunit ost4p | *Saccharomyces cerevisiae* | 7-21 | 47.89 |
| Oligosaccharyltransferase subunit ost4p | *Saccharomyces cerevisiae* | 7-21 | 47.89 |
| Subunit of MRX complex | *Saccharomyces cerevisiae* | 48-56 | 47.83 |
| Glutamine ABC transporter (glutamine-binding protein) | *Bacillus subtilis* | 5-64 | 47.75 |
| UCP018933 |  | 47-69 | 47.22 |
| ABC transporter | *Beggiatoa sp. PS* | 48-56 | 46.29 |
| LHC Antenna complex alpha/beta subunit |  | 5-26 | 46.13 |
| RAD50 homolog isoform 1 | *Homo sapiens* | 48-56 | 45.17 |
| ATP-binding protein | *Beggiatoa sp. PS* | 48-56 | 44.98 |
| MotB flagellar motor protein MotB |  | 11-23 | 44.88 |
| F57C2.5 | *Caenorhabditis elegans* | 4-38 | 44.66 |
| Glycine rich protein family |  | 1-53 | 44.60 |
| UbiA prenyltransferase | *Nostoc punctiforme* | 9-31 | 44.58 |
| **BLASTP** | | | |
| Bifunctional 2',3'-cyclic nucleotide 2'-phosphodiesterase/3'-nucleotidase precursor protein |  | 12-81 |  |
| **I-TASSER** | | | |
| Pilin, type IV | *Thermus thermophilus* |  | 1.04, 1.07 |
| Anastral spindle 2, SAS 4 | *Drosophila melanogaster* |  | 1.00 |
| Phosphatidylinositol-4,5-bisphosphate 3-kinase catalytic subunit gamma isoform | *Homo sapiens* |  | 0.617 |
| Fructose 1,6-bisphosphatase/inositol monophosphatase | *Archaeoglobus fulgidus* |  | 0.617 |
| Inositol monophosphatase | *Zymomonas mobilis* |  | 0.606 |
| Pilin, type IV | *Thermus thermophilus* |  | 0.591 |
| Pseudopilin GspK | *Escherichia coli* |  | 0.589 |
| Fructose 1,6-bisphosphatase | *Pisum sativum* |  | 0.589 |
| Fimbrial protein | *Pseudomonas aeruginosa* |  | 0.588 |
| Xaa-Pro aminopeptidase 1 | *Homo sapiens* |  | 0.588 |
| Type IV pilin | *Pseudomonas aeruginosa* |  | 0.580 |
| **Predict Protein** | | | |
| Protein binding |  | 1,2,22,25,28, 32,34,37,57, 80,85-86 |  |
| Secreted |  |  |  |
| **Atome2** | | | |
| Major capsid protein (protein P3) | *Enterobacteria phage* |  | 80.01 |
| Importin alpha-1 subunit | *Homo sapiens* |  | 71.81 |
| Type II restriction enzyme HindIII | *Haemophilus influenzae* |  | 66.46 |
| AS-48 protein | *Enterococcus faecalis* |  | 63.35 |
| Stromal cell-derived factor 1 | *Homo sapiens* |  | 55.61 |
| Photosynthetic reaction center C subunit | *Thermochromatium tepidum* |  | 49.31 |
| Archaeal adhesion filament core | *Ignicoccus hospitalis* |  | 45.48 |
| Light-harvesting protein B-800/850, alpha chain | *Rhodoblastus acidophilus* |  | 42.22 |
| Light-harvesting protein B-880, beta chain | *Rhodospirillum rubrum* |  | 37.37 |
| Chromosome segregation protein smc | *Pyrococcus furiosus* |  | 31.93 |
| Phosphate starvation-inducible protein | *Corynebacterium glutamicum* |  | 31.63 |
| Guanylate kinase | *Coxiella burnetii* |  | 31.61 |
| Light harvesting complex II | *Phaeospirillum molischianum* |  | 31.23 |
| Chromosome segregation SMC protein | *Thermotoga maritima* |  | 30.86 |
| Cytochrome c oxidase, cbb3-type, subunit N | *Pseudomonas stutzeri* |  | 27.90 |
| Guanylate kinase | *Mus musculus* |  | 27.75 |
| Fructokinase | *Ruegeria sp. TM1040* |  | 27.69 |

Table S27. *Cumberlandia monodonta* F*-ORF* function predictions

| **Hits** | **Species** | **Position** | **Probability** |
| --- | --- | --- | --- |
| **HHpred** | | | |
| TIGR03304 outer membrane insertion C-terminal signal |  | 12-14 | 99.21 |
| TIGR04294 prepilin-type processing-associated H-X9-DG domain |  | 48-50 | 99.04 |
| TIGR01167 LPXTG cell wall anchor domain |  | 72-76 | 98.83 |
| TIGR03057 X-X-X-Leu-X-X-Gly heptad repeats |  | 18-27 | 97.70 |
| TIGR03501 GlyGly-CTERM domain |  | 4-15 | 96.86 |
| TIGR00756 pentatricopeptide repeat domain |  | 59-68 | 93.47 |
| F46H6.5 | *Caenorhabditis elegans* | 56-75 | 76.05 |
| UCP029505 |  | 6-16 | 69.88 |
| PEP-CTERM protein-sorting domain |  | 64-69 | 46.91 |
| TIGR04288 CGP-CTERM domain |  | 2-12 | 46.76 |
| Conserved inner membrane protein | *Escherichia coli* | 2-17 | 40.08 |
| COX7 |  | 2-16 | 36.69 |
| Conserved integral membrane protein | *Corynebacterium diphtheriae* | 3-28 | 34.75 |
| Y54E10BL.2 | *Caenorhabditis elegans* | 1-27 | 34.23 |
| Homodimeric domain of signal transducing histidine kinase (47384) SCOP seed sequence: d1joya_ |  | 36-42 | 33.23 |
| DumPY : shorter than wild-type family member (dpy-14) | *Caenorhabditis elegans* | 1-27 | 31.73 |
| T10E10.2 | *Caenorhabditis elegans* | 1-27 | 31.04 |
| DumPY : shorter than wild-type family member (dpy-2) | *Caenorhabditis elegans* | 1-14 | 30.61 |
| OSMotic avoidance abnormal family member (osm-10) | *Caenorhabditis elegans* | 28-51 | 29.76 |
| F46B6.10 | *Caenorhabditis elegans* | 6-89 | 29.15 |
| DumPY : shorter than wild-type family member (dpy-10) | *Caenorhabditis elegans* | 1-14 | 28.10 |
| COLlagen family member (col-84) | *Caenorhabditis elegans* | 1-14 | 28.10 |
| F38A3.1 | *Caenorhabditis elegans* | 1-27 | 27.34 |
| T10E10.1 | *Caenorhabditis elegans* | 1-27 | 27.33 |
| COLlagen family member (col-2) | *Caenorhabditis elegans* | 2-27 | 26.87 |
| COLlagen family member (col-36) | *Caenorhabditis elegans* | 1-27 | 26.73 |
| F15H10.1 | *Caenorhabditis elegans* | 1-27 | 26.57 |
| Nop10-like SnoRNP (144210) SCOP seed sequence: d2ey4e1 |  | 50-72 | 26.50 |
| ROLler: helically twisted, animals roll when moving family member (rol-1) | *Caenorhabditis elegans* | 1-14 | 26.24 |
| C34F6.3 | *Caenorhabditis elegans* | 1-27 | 25.86 |
| COLlagen family member (col-106) | *Caenorhabditis elegans* | 2-27 | 25.77 |
| CG13783-PA | *Drosophila melanogaster* | 10-28 | 24.23 |
| F15H10.2 |  | 1-27 | 24.06 |
| Methylene tetrahydromethanopterin dehydrogenase |  | 33-47 | 23.46 |
| fixS protein | *Neisseria meningitidis* | 6-19 | 23.35 |
| F11G11.12 | *Caenorhabditis elegans* | 1-27 | 23.32 |
| Chondrolectin precursor | *Homo sapiens* | 2-20 | 23.15 |
| F57B1.4 | *Caenorhabditis elegans* | 1-27 | 22.76 |
| T21B4.2 | *Caenorhabditis elegans* | 1-27 | 22.47 |
| Y69H2.14 | *Caenorhabditis elegans* | 1-27 | 22.39 |
| F57B1.3 | *Caenorhabditis elegans* | 1-27 | 22.39 |
| BLIstered cuticle family member (bli-2) | *Caenorhabditis elegans* | 1-27 | 22.37 |
| COLlagen family member (col-51) | *Caenorhabditis elegans* | 2-27 | 22.30 |
| ROLler: helically twisted, animals roll when moving family member (rol-8) | *Caenorhabditis elegans* | 1-27 | 22.10 |
| T10E10.6 | *Caenorhabditis elegans* | 1-27 | 22.08 |
| DumPY : shorter than wild-type family member (dpy-10) | *Caenorhabditis elegans* | 1-27 | 21.85 |
| Virus attachment protein globular domain (49835) SCOP seed sequence: d1h7za_ |  | 50-68 | 21.78 |
| Adenovirus fibre protein; cell receptor recognition, receptor | *Human adenovirus type 3* | 44-68 | 21.71 |
| COLlagen family member (col-165) | *Caenorhabditis elegans* | 1-27 | 21.58 |
| C44C10.1 | *Caenorhabditis elegans* | 1-27 | 21.26 |
| Photosystem II reaction centre X protein (PsbX) | *Synechococcus sp. CC9311* | 5-26 | 21.26 |
| COLlagen family member (col-110) | *Caenorhabditis elegans* | 2-27 | 20.99 |
| DumPY : shorter than wild-type family member (dpy-9) | *Caenorhabditis elegans* | 2-27 | 20.99 |
| DumPY : shorter than wild-type family member (dpy-3) | *Caenorhabditis elegans* | 1-27 | 20.77 |
| COLlagen family member (col-34) | *Caenorhabditis elegans* | 2-27 | 20.65 |
| FAD/NAD-linked reductases, dimerisation (C-terminal) domain (55424) SCOP seed sequence: d1d7ya3 |  | 10-26 | 20.64 |
| F17C11.3 | *Caenorhabditis elegans* | 2-27 | 20.63 |
| COLlagen family member (col-173) | *Caenorhabditis elegans* | 1-27 | 20.56 |
| Secreted protein | *Streptomyces coelicolor* | 4-19 | 20.37 |
| K08F4.5 | *Caenorhabditis elegans* | 2-21 | 20.33 |
| C34F6.2 | *Caenorhabditis elegans* | 2-27 | 20.30 |
| COLlagen family member (col-124) | *Caenorhabditis elegans* | 2-27 | 20.27 |
| F32G8.5 | *Caenorhabditis elegans* | 2-25 | 20.14 |
| **I-TASSER** | | | |
| Sec-independent protein translocase protein TatB | *Escherichia coli* |  | 1.13 |
| Sts-2 protein | *Mus musculus* |  | 0.501 |
| **Predict Protein** | | | |
| Protein binding |  | 1,2,52,62 |  |
| Mitochondrial membrane |  |  |  |
| **Atome2** | | | |
| Preprotein translocase SecA subunit | *Thermus thermophilus* |  | 72.89 |
| Deoxyribonuclease I | *Bos taurus* |  | 63.10 |
| FLAP endonuclease-1 protein | *Methanocaldococcus jannaschii* |  | 58.69 |
| E3 ubiquitin-protein ligase UBR2 | *Homo sapiens* |  | 48.38 |
| Potassium large conductance calcium-activated channel, subfamily M, beta member 2 | *Homo sapiens* |  | 45.89 |
| S-locus pollen protein | *Brassica rapa* |  | 43.02 |
| Regulatory protein SIR4 | *Saccharomyces cerevisiae* |  | 41.26 |
| mRNA 3'-end-processing protein RNA14 | *Kluyveromyces lactis* |  | 36.60 |
| Proliferating cell nuclear antigen | *Homo sapiens* |  | 35.73 |
| Protein (adenovirus fibre) | *Homo sapiens* |  | 33.59 |
| Fiber protein | *Human adenovirus 37* |  | 31.21 |
| Fiber protein | *Human adenovirus 2* |  | 30.90 |
| Adenovirus type 5 fiber protein | *Human adenovirus 5* |  | 30.46 |
| Fiber protein | *Human adenovirus 41* |  | 24.60 |
| Transmembrane protein 173 | *Homo sapiens* |  | 23.18 |
| Stimulator of interferon genes protein | *Homo sapiens* |  | 19.47 |
| Oncogene product P14TCL1 | *Homo sapiens* |  | 16.55 |
| HMTCP-1 | *Homo sapiens* |  | 13.22 |

Table S28. *Hyridella menziesii* F*-ORF* function predictions

| **Hits** | **Species** | **Position** | **Probability** |
| --- | --- | --- | --- |
| **HHpred** | | | |
| TIGR04294 prepilin-type processing-associated H-X9-DG domain |  | 80-85 | 99.25 |
| TIGR01167 LPXTG cell wall anchor domain |  | 8-35 | 99.10 |
| TIGR03304 outer membrane insertion C-terminal signal |  | 1-6 | 99.05 |
| TIGR03057 X-X-X-Leu-X-X-Gly heptad repeats |  | 8-12 | 97.66 |
| TIGR03501 GlyGly-CTERM domain |  | 23-35 | 96.93 |
| TIGR00756 pentatricopeptide repeat domain |  | 60-69 | 93.52 |
| MotB_plug Membrane MotB of proton-channel complex MotA/MotB. |  | 17-38 | 87.38 |
| Flagellar motor protein | *Bacillus subtilis* | 1-38 | 87.21 |
| CD274 antigen | *Homo sapiens* | 2-65 | 86.19 |
| Partially redundant sensor-transducer of the stress-activated PKC1-MPK1 signaling pathway | *Saccharomyces cerevisiae* | 6-37 | 85.09 |
| motB flagellar motor protein MotB |  | 17-38 | 84.85, 84.34 |
| MotB_plug: Membrane MotB of proton-channel complex MotA/MotB |  | 17-38 | 84.08 |
| motB flagellar motor protein MotB |  | 1-38 | 82.76 |
| Flagellar motor protein MotS |  | 5-38 | 82.25 |
| Glycophorin |  | 17-38 | 80.00 |
| Flagellar motor protein MotD |  | 1-38 | 79.60 |
| Flagellar motor protein MotB | *Escherichia coli* | 3-38 | 79.38 |
| Basigin | *Mus musculus* | 3-73 | 77.67 |
| MEGF11 protein | *Homo sapiens* | 8-37 | 77.66 |
| Cell division protein | *Yersinia pestis* | 6-40 | 77.15 |
| Transmembrane glycoprotein A33 precursor | *Homo sapiens* | 1-36 | 76.89 |
| Flagellar motor protein | *Yersinia pestis* | 5-38 | 76.41 |
| Leukocyte-associated immunoglobulin-like receptor 1 isoform b precursor | *Homo sapiens* | 5-43 | 76.34 |
| Flagellar motor protein | *Bacillus subtilis* | 17-38 | 76.34 |
| C35D10.1 | *Caenorhabditis elegans* | 14-42 | 76.14 |
| EGF-like-domain, multiple 9 | *Homo sapiens* | 2-37 | 76.07 |
| EGF-like-domain, multiple 9 | *Homo sapiens* | 2-37 | 76.07 |
| Carbamoyl-phosphate synthase L chain, ATP-binding | *Nostoc punctiforme* | 7-46 | 76.06 |
| MEGF10 protein | *Homo sapiens* | 2-37 | 75.94 |
| Golgi membrane protein with similarity to mammalian CASP | *Saccharomyces cerevisiae* | 10-38 | 75.88 |
| motB flagellar motor protein MotB |  | 17-38 | 75.30 |
| RIKEN cDNA 2900064A13 | *Mus musculus* | 14-39 | 75.22 |
| CG18146-PB, isoform B | *Drosophila melanogaster* | 17-37 | 74.16 |
| Syntaxin 7 | *Homo sapiens* | 11-39 | 73.60 |
| Glycoprotein A33 (transmembrane) | *Mus musculus* | 1-36 | 73.22 |
| CG31136-PA | *Drosophila melanogaster* | 17-37 | 71.97 |
| Chain length determinant protein | *Beggiatoa sp. PS* | 8-38 | 71.77 |
| Kin of IRRE-like 2 | *Mus musculus* | 18-86 | 71.74 |
| Neuregulin 4 | *Mus musculus* | 3-36 | 71.40 |
| motB flagellar motor protein MotB; Validated |  | 17-38 | 71.22 |
| Vesicle-associated membrane protein 1 isoform 1 | *Homo sapiens* | 17-38 | 71.16 |
| STL2P | *Arabidopsis thaliana* | 4-39 | 70.59 |
| Flagellar motor protein | *Yersinia pestis* | 17-39 | 70.30 |
| RCR |  | 18-38 | 68.98 |
| NHL12 | *Arabidopsis thaliana* | 18-56 | 68.76 |
| ZK353.4 | *Caenorhabditis elegans* | 12-34 | 68.51 |
| Flagellar motor protein | *Pseudomonas aeruginosa* | 17-38 | 67.85 |
| VAMP-5_synaptobrevin |  | 17-37 | 67.62 |
| T20D4.12 | *Caenorhabditis elegans* | 17-48 | 67.04 |
| CCAAT displacement protein isoform c | *Homo sapiens* | 3-39 | 66.72 |
| SIT: SHP2-interacting transmembrane adaptor protein, SIT |  | 18-42 | 66.62 |
| Endomucin |  | 8-42 | 66.54 |
| CCAAT displacement protein isoform b | *Homo sapiens* | 3-39 | 66.34 |
| Capsular polysaccharide biosynthesis protein Cap1A | *Staphylococcus aureus* | 11-39 | 66.20 |
| SYP61 | *Arabidopsis thaliana* | 11-36 | 65.62 |
| ATP binding / kinase/ protein kinase/ protein serine/threonine kinase/ protein-tyrosine kinase | *Arabidopsis thaliana* | 1-92 | 65.08 |
| Regulator of length of O-antigen component of lipopolysaccharide chains | *Escherichia coli* | 11-38 | 65.00 |
| Essential cell division protein | *Escherichia coli* | 6-40 | 64.98 |
| YLS9 | *Arabidopsis thaliana* | 7-56 | 64.93 |
| Integrin alpha-IIB | *Homo sapiens* | 8-40 | 64.81 |
| F11 receptor | *Mus musculus* | 18-65 | 64.72 |
| SIT SHP2-interacting transmembrane adaptor protein |  | 18-42 | 64.07 |
| Vesicle transport through interaction with t-SNAREs homolog 1A | *Mus musculus* | 17-39 | 63.82 |
| **I-TASSER** | | | |
| Type I restriction-modification system methyltransferase subunit | *Vibrio vulnificus* |  | 0.527 |
| Poly(ADP-ribose) glycohydrolase | *Rattus norvegicus* |  | 0.512 |
| Transporter | *Aquifex aeolicus* |  | 0.505 |
| Mre11 nuclease | *Pyrococcus furiosus* |  | 0.503 |
| **Predict Protein** | | | |
| Protein binding |  | 1,38-40, 54-56, 75-77, 79,81 |  |
| Secreted |  |  |  |
| **Atome2** | | | |
| Nucleoprotein | *Influenza A virus* |  | 80.49 |
| Tankyrase-1 | *Mus musculus* |  | 56.43 |
| Carboxypeptidase A1 | *Bos taurus* |  | 45.65 |
| Stromal cell-derived factor 1 | *Homo sapiens* |  | 42.76 |
| Integrin alpha-IIb **(3)** | *Homo sapiens* |  | 28.46 |
| Na, K-ATPase alpha subunit | *Squalus acanthias* |  | 33.92 |
| Integrin alpha-1 | *Homo sapiens* |  | 33.59 |
| HIG1 domain family member 1A | *Homo sapiens* |  | 33.37 |
| Sodium/potassium-transporting ATPase subunit alpha-1 | *Sus scrofa* |  | 32.09 |
| Pulmonary surfactant-associated polypeptide C | *Sus scrofa* |  | 31.12 |
| Phospholemman | *Homo sapiens* |  | 31.06 |
| Importin subunit alpha-2 | *Mus musculus* |  | 30.90 |
| T-cell surface glycoprotein CD4 | *Homo sapiens* |  | 30.38 |
| SERCA1a | *Oryctolagus cuniculus* |  | 29.56 |
| Potassium channel protein RCK4 | *Homo sapiens* |  | 29.40 |
| Vesicle-associated membrane protein 2 | *Rattus norvegicus* |  | 27.79 |
| Beta-type platelet-derived growth factor receptor | *Homo sapiens* |  | 27.49 |
| Integrin alpha-IIb light chain | *Homo sapiens* |  | 26.38 |

Table S29. *Lasmigona complanata* F*-ORF* function predictions

| **Hits** | **Species** | **Position** | **Probability** |
| --- | --- | --- | --- |
| **HHpred** | | | |
| TIGR01167 LPXTG cell wall anchor domain |  | 54-59 | 99.46 |
| TIGR04294 prepilin-type processing-associated H-X9-DG domain |  | 18-21 | 99.32 |
| TIGR03304 outer membrane insertion C-terminal signal |  | 34-35 | 99.27 |
| TIGR03057 X-X-X-Leu-X-X-Gly heptad repeats |  | 46-53 | 98.05 |
| TIGR03501 GlyGly-CTERM domain |  | 8-18 | 97.32 |
| TIGR00756 pentatricopeptide repeat domain |  | 38-45 | 94.58 |
| CG7685-PA | *Drosophila melanogaster* | 2-26 | 93.27 |
| Intra-Golgi v-SNARE, required for transport of proteins between an early and a later Golgi compartment | *Saccharomyces cerevisiae* | 3-26 | 84.46 |
| TMEM156: TMEM156 protein family **(2)** |  | 1-27 | 78.90, 78.70 |
| Golgi SNARE BET1-related |  | 3-24 | 71.61 |
| CG13969-PA | *Drosophila melanogaster* | 3-44 | 71.25 |
| Ceramidase |  | 3-44 | 70.74 |
| Sensor histidine kinase | *Streptococcus pneumoniae* | 1-40 | 69.88 |
| LptF_YjgP LPS export ABC transporter permease LptF |  | 1-29 | 67.65 |
| Sensory box histidine kinase PhoR | *Staphylococcus aureus* | 1-36 | 67.50 |
| Saliv_gland_allergen_Aed3 |  | 2-19 | 64.97 |
| Conserved inner membrane protein | *Escherichia coli* | 1-29 | 63.38 |
| T27F7.3a | *Caenorhabditis elegans* | 2-36 | 62.64 |
| GRP: Glycine rich protein family |  | 6-26 | 61.27 |
| Sterol reductase/lamin B receptor |  | 19-47 | 60.88 |
| Essential SNARE protein localized to the ER | *Saccharomyces cerevisiae* | 5-26 | 60.56 |
| Alkaline ceramidase 2 | *Mus musculus* | 3-44 | 58.05 |
| Vesicle-associated membrane-associated protein |  | 4-26 | 57.11 |
| Human EMeRin homolog family member (emr-1) | *Caenorhabditis elegans* | 6-23 | 56.93 |
| GRP Glycine rich protein family |  | 5-26 | 56.69 |
| CbiN ABC-type cobalt transport system, periplasmic component |  | 1-36 | 55.84 |
| W02F12.2 | *Caenorhabditis elegans* | 3-53 | 55.64 |
| Related to YPC1 - Alkaline ceramidase |  | 3-44 | 55.42 |
| Lipoprotein required for capsular polysaccharide translocation through the outer membrane | *Escherichia coli K12* | 1-19 | 54.31 |
| LptG_lptG LPS export ABC transporter permease LptG |  | 1-29 | 53.94 |
| CG11020-PA, isoform A | *Drosophila melanogaster* | 1-38 | 52.93 |
| CG3066-PD, isoform D | *Drosophila melanogaster* | 2-27 | 52.93 |
| SVM_signal: SVM protein signal sequence |  | 2-23 | 51.26 |
| DumPY: shorter than wild-type family member (dpy-5) | *Caenorhabditis elegans* | 1-41 | 50.82 |
| Alkaline ceramidase that also has reverse (CoA-independent) ceramide synthase activity | *Saccharomyces cerevisiae* | 3-44 | 49.99 |
| Retinoblastoma-associated protein | *Homo sapiens* | 29-46 | 49.30 |
| Protein transporter | *Arabidopsis thaliana* | 3-26 | 48.30 |
| Signal transduction histidine kinase | *Lactobacillus casei* | 2-36 | 46.70 |
| N-acylsphingosine amidohydrolase 3 | *Homo sapiens* | 3-44 | 45.65 |
| F59E11.5 | *Caenorhabditis elegans* | 2-31 | 44.97 |
| Ceramidase |  | 3-44 | 44.88 |
| Cytoplasmic membrane protein | *Bartonella henselae* | 1-34 | 44.46 |
| SYP125; t-SNARE | *Arabidopsis thaliana* | 6-29 | 44.28 |
| Rhodanese-like protein | *Beggiatoa sp. PS* | 2-22 | 43.79 |
| MORN repeat protein | *Beggiatoa sp. PS* | 1-21 | 43.59 |
| Protein containing DUF1239 | *Beggiatoa sp. PS* | 1-22 | 43.55 |
| Y41D4B.24 | *Caenorhabditis elegans* | 3-34 | 43.31 |
| Y110A7A.11 | *Caenorhabditis elegans* | 5-26 | 43.12 |
| COLlagen family member (col-102) | *Caenorhabditis elegans* | 1-46 | 42.45 |
| C46H11.8 | *Caenorhabditis elegans* | 6-20 | 42.31 |
| Vesicle-associated membrane protein | *Mus musculus* | 4-26 | 42.29 |
| SrtB |  | 1-34 | 42.20 |
| Urinary protein (RUP)/acrosomal protein SP-10 |  | 1-27 | 41.83 |
| ATCDS1; phosphatidate cytidylyltransferase | *Arabidopsis thaliana* | 45-77 | 41.81 |
| Temporarily Assigned Gene name family member (tag-254) | *Caenorhabditis elegans* | 6-21 | 41.51 |
| Golgi phosphoprotein 2 | *Homo sapiens* | 1-39 | 41.51 |
| RCR |  | 8-23 | 41.47 |
| **BLASTP** | | | |
| Membrane protein | *Enterococcus faecium* | 2-77 | 1.00e-06 |
| MULTISPECIES: membrane protein | *Enterococcus* | 4-77 | 5.00e-06 |
| Glycyl-tRNA synthetase subunit alpha | *Avibacterium sp.* | 23-77 | 2.00e-05 |
| Glycyl-tRNA synthetase subunit alpha | *Vibrio litoralis* | 23-77 | 2.00e-05 |
| COG0752 Glycyl-tRNA synthetase, alpha subunit | *uncultured bacterium B3TF_MPn_8* | 23-77 | 3.00e-05 |
| MULTISPECIES: glycyl-tRNA synthetase subunit alpha | *Vibrio* | 23-77 | 3.00e-05 |
| Glycyl-tRNA synthetase subunit alpha | *Vibrio caribbeanicus* | 23-77 | 3.00e-05 |
| Glycyl-tRNA synthetase, partial | *Vibrio campbellii* | 23-77 | 4.00e-05 |
| Deacylase | *Maribacter sp. HTCC2170* | 3-53 | 7.00e-05 |
| Glycyl-tRNA synthetase alpha chain | *Vibrio sp. JCM 19236* | 23-77 | 7.00e-05 |
| Lebocin-like antibacterial protein | *Heliothis virescens* | 6-76 | 2.00e-04 |
| 2-oxoglutarate dehydrogenase E2 | *Staphylococcus hominis* | 34-77 | 4.00e-04 |
| P2Y purinoceptor 1, partial | *Podiceps cristatus* | 1-49 | 5.00e-04 |
| Transporter | *Rickettsia typhi* | 8-44 | 5.00e-04 |
| Transporter | *Rickettsia prowazekii* | 8-44 | 6.00e-04 |
| P2Y purinoceptor 1, partial | *Gavia stellata* | 1-49 | 6.00e-04 |
| Chemotaxis protein | *Lactobacillus parafarraginis* | 15-74 | 6.00e-04 |
| Permease | *Rickettsia prowazekii* | 28-65 | 7.00e-04 |
| **I-TASSER** | | | |
| Fimbrial protein (Pilin) | *Peptoclostridium difficile* |  | 0.667 |
| Residues 29-152, plus four N-terminal residues from the expression construct | *Neisseria meningitidis* |  | 0.622 |
| Wnt inhibitor of Dorsal protein (N-terminal domain-linker) | *Drosophila melanogaster* |  | 0.618 |
| Cytochrome P450ERYF | *Saccharopolyspora erythraea* |  | 0.612 |
| Cytochrome P450 cypX | *Bacillus subtilis* |  | 0.607 |
| Inositol-1-monophosphatase | *Mycobacterium tuberculosis* |  | 0.606 |
| Cytochrome P450 119 | *Sulfolobus solfataricus* |  | 0.605 |
| Cytochrome P450 107B1 (P450CVIIB1) | *Streptomyces himastatinicus* |  | 0.604 |
| Oxy protein | *Actinoplanes teichomyceticus* |  | 0.602 |
| 367aa long hypothetical cytochrome P450 | *Sulfolobus tokodaii* |  | 0.600 |
| **Predict Protein** | | | |
| Protein binding |  | 1-2,5, 31-32, 34-35, 48,51,57 |  |
| Mitochondrial membrane |  |  |  |
| **Atome2** | | | |
| P fimbrial regulatory protein KS71A | *Escherichia coli* |  | 92.97 |
| Protein (neamatode anticoagulant protein C2) | *Ancylostoma caninum* |  | 62.39 |
| Herpes simplex virus protein ICP47 (active domain) | *Herpes simplex virus* |  | 46.61 |
| Polyribonucleotide nucleotidyltransferase | *Escherichia coli* |  | 42.79 |
| Neurotoxin BmP03 | *Mesobuthus martensii* |  | 41.75 |
| Calcium-gated potassium channel mthK | *Methanothermobacter thermautotrophicus* |  | 38.42 |
| CREB-binding protein | *Mus musculus* |  | 37.95 |
| Polyribonucleotide nucleotidyltransferase | *Escherichia coli* |  | 34.92 |
| Protein translocase subunit secA | *Thermotoga maritima* |  | 33.09 |
| Protein translocase subunit secA | *Bacillus subtilis* |  | 33.06 |
| Protein-export membrane protein secG | *Escherichia coli* |  | 31.50 |
| CPAP | *Danio rerio* |  | 26.35 |
| Transcription factor Dp-1 | *Homo sapiens* |  | 24.47 |
| FAB | *Mus musculus* |  | 16.70 |

Table S30. *Toxolasma lividus* F*-ORF* function predictions

| **Hits** | **Species** | **Position** | **Probability** |
| --- | --- | --- | --- |
| **HHpred** | | | |
| TIGR04294 prepilin-type processing-associated H-X9-DG domain |  | 34-36 | 99.23 |
| TIGR03304 outer membrane insertion C-terminal signal |  | 1-8 | 99.14 |
| TIGR01167 LPXTG cell wall anchor domain |  | 95-96 | 98.81 |
| TIGR03057 X-X-X-Leu-X-X-Gly heptad repeats |  | 18-22 | 97.99 |
| GlyGly-CTERM domain |  | 50-60 | 97.08 |
| TIGR00756 pentatricopeptide repeat domain |  | 30-49 | 94.54 |
| ComGC |  | 45-62 | 91.25 |
| Stage III sporulation protein AF |  | 18-63 | 80.41 |
| Protein-export membrane protein |  | 34-111 | 75.33 |
| Transducer protein Htr37 |  | 24-64 | 74.74 |
| Syntaxin-like t-SNARE |  | 37-98 | 70.94 |
| Mutants block sporulation after engulfment (stage III) |  | 18-63 | 70.72 |
| VAMP-5_synaptobrevin |  | 36-68 | 67.81 |
| CG11815-PA | *Drosophila melanogaster* | 77-99 | 66.44 |
| C-type LECtin family member (clec-35) | *Caenorhabditis elegans* | 28-110 | 65.05 |
| ComGC Competence protein ComGC |  | 45-62 | 62.86 |
| COLlagen family member (col-14) | *Caenorhabditis elegans* | 21-65 | 58.40 |
| Pili subunits (54523) SCOP seed sequence: d2pila_ |  | 4-62 | 56.94 |
| General secretion pathway protein H | *Nostoc punctiforme* | 28-63 | 56.08 |
| Methyl-accepting chemotaxis protein | *Beggiatoa sp. PS* | 33-59 | 55.50 |
| Stage III sporulation protein AF (Spore_III_AF) |  | 3-63 | 54.93 |
| Opacity-associated protein A N-terminal motif |  | 40-61 | 53.84 |
| Protein involved in cis-Golgi membrane traffic; v-SNARE | *Saccharomyces cerevisiae* | 25-64 | 53.77 |
| Pili subunits (54523) SCOP seed sequence: d1oqwa_ |  | 44-62 | 53.25 |
| F08F8.8 | *Caenorhabditis elegans* | 19-64 | 53.15 |
| DevC protein | *Nostoc punctiforme* | 24-66 | 52.26 |
| Transducer protein Htr36 | *Haloferax volcanii* | 26-64 | 50.87 |
| Prion-like-(Q/N-rich)-domain-bearing protein family member (pqn-34) | *Caenorhabditis elegans* | 59-104 | 50.27 |
| Flagellar M-ring protein | *Bacillus subtilis* | 25-79 | 49.97 |
| SecD-TM1 SecD export protein N-terminal TM region |  | 35-63 | 48.71 |
| C-type LECtin family member (clec-25) | *Caenorhabditis elegans* | 43-107 | 48.40 |
| Vesicle transport through interaction with t-SNAREs 1B | *Homo sapiens* | 25-64 | 48.33 |
| Y57G11C.4 | *Caenorhabditis elegans* | 25-63 | 47.57 |
| Methyl-accepting chemotaxis protein II | *Yersinia pestis* | 31-64 | 46.49 |
| Stage III sporulation protein AF (Spore_III_AF) |  | 30-63 | 46.41 |
| DevC protein | *Nostoc punctiforme* | 26-65 | 45.60 |
| T10E10.5 | *Caenorhabditis elegans* | 25-66 | 44.80 |
| CG3279-PA | *Drosophila melanogaster* | 25-76 | 44.73 |
| SYP123; t-SNARE | *Arabidopsis thaliana* | 25-66 | 43.93 |
| VTI12; SNARE binding/receptor | *Arabidopsis thaliana* | 25-64 | 43.41 |
| Protein export protein SecD | *Neisseria meningitidis* | 35-67 | 43.40 |
| VTI11; receptor | *Arabidopsis thaliana* | 25-64 | 42.17 |
| Vesicle-associated membrane protein 5 (myobrevin) | *Homo sapiens* | 36-69 | 41.51 |
| T24B1.1 | *Caenorhabditis elegans* | 31-69 | 41.28 |
| Vesicle transport v-snare protein | *Schizosaccharomyces pombe* | 25-64 | 41.03 |
| Intra-Golgi v-SNARE, required for transport of proteins between an early and a later Golgi compartment | *Saccharomyces cerevisiae* | 25-63 | 40.76 |
| F41F3.3 | *Caenorhabditis elegans* | 42-61 | 40.59 |
| TonB family protein | *Nostoc punctiforme 73102* | 37-81 | 40.54 |
| Related to VTI1 - v-SNARE: involved in Golgi retrograde protein traffic |  | 25-64 | 39.71 |
| Proline-rich region | *Synechococcus sp.* | 23-66 | 39.54 |
| Resistance to inhibitors of cholinesterase 3 homolog | *Homo sapiens* | 41-105 | 39.30 |
| Protein export protein SecD | *Pseudomonas aeruginosa* | 35-67 | 38.85 |
| Methyl-accepting chemotaxis protein III | *Escherichia coli* | 33-59 | 38.71 |
| Competence protein CglC | *Streptococcus pneumoniae* | 26-63 | 38.44 |
| COLlagen family member (col-77) | *Caenorhabditis elegans* | 25-66 | 37.87 |
| v-SNARE (vesicle specific SNAP receptor) | *Saccharomyces cerevisiae* | 19-64 | 37.76 |
| Transcriptional accessory factor Tex **(2)** | *Pseudomonas aeruginosa* | 79-99 | 37.60 |
| F0F1 ATP synthase subunit A | *Mycobacterium tuberculosis* | 18-67 | 37.34 |
| a disintegrin and metalloproteinase domain 7 | *Homo sapiens* | 45-108 | 37.11 |
| CG13581-PA | *Drosophila melanogaster* | 101-113 | 36.38 |
| Protein export protein SecD | *Escherichia coli* | 35-67 | 36.37 |
| SYP124; t-SNARE | *Arabidopsis thaliana* | 25-64 | 36.06 |
| Laeverin | *Homo sapiens* | 40-111 | 35.41 |
| COLlagen family member (col-174) | *Caenorhabditis elegans* | 25-66 | 35.28 |
| CG11500-PA | *Drosophila melanogaster* | 5-89 | 35.23 |
| SecD-TM1: SecD export protein N-terminal TM region |  | 36-67 | 34.96 |
| Multi-sensor signal transduction histidine kinase | *Nostoc punctiforme* | 21-64 | 34.75 |
| MacB_PCD MacB-like periplasmic core domain. |  | 32-66 | 34.43 |
| Type IV Pilin Pak | *Pseudomonas aeruginosa* | 44-62 | 34.26 |
| **I-TASSER** | | | |
| UNC-45 protein, SD10334p | *Drosophila melanogaster* |  | 0.512 |
| RCD1 required for cell differentiation1 homolog | *Homo sapiens* |  | 0.509 |
| Chloride intracellular channel exc-4 | *Caenorhabditis elegans* |  | 0.504 |
| Telomerase-binding protein EST1A (tetratricopeptide repeat, residues 580-1166) |  |  | 0.502 |
| Protein UNC-45 | *Caenorhabditis elegans* |  | 0.502 |
| Karyopherin alpha (armadillo domain) | *Saccharomyces cerevisiae* |  | 0.500 |
| **Predict Protein** | | | |
| Protein binding |  | 1,3,5,10,11,14-18,20-21,31, 35,38-39,64-66,68,70,72, 90,94-95,97 |  |
| Polynucleotide binding |  | 27 |  |
| Mitochondrial membrane |  |  |  |
| **Atome2** | | | |
| FLT3 ligand (receptor binding domain) | *Homo sapiens* |  | 99.28 |
| Protein parD | *Escherichia coli* |  | 59.11 |
| Intrinsic membrane protein pufX | *Rhodobacter sphaeroides* |  | 54.16 |
| Envelope protein E | *Dengue virus* |  | 46.45 |
| Neopetrosiamide A | *Neopetrosia sp.* |  | 38.08 |
| Laccase | *Rigidoporus microporus* |  | 30.09 |
| Laccase | *Botrytis aclada* |  | 25.58 |
| Ascorbate oxidase | *Cucurbita pepo* |  | 23.65 |
| Iron transport multicopper oxidase FET3 | *Saccharomyces cerevisiae* |  | 23.49 |
| Laccase 1 | *Coprinopsis cinerea* |  | 22.43 |
| Laccase | *Steccherinum ochraceum* |  | 22.40 |
| Fimbrial protein | *Neisseria gonorrhoeae* |  | 20.56 |
| Laccase-1 | *Melanocarpus albomyces* |  | 15.28 |

Table S31. *Margaritifera margaritifera* F*-ORF* function predictions

| **Hits** | **Species** | **Position** | **Probability** |
| --- | --- | --- | --- |
| **HHpred** | | | |
| TIGR03304 outer membrane insertion C-terminal signal |  | 23-24 | 99.14 |
| TIGR04294 prepilin-type processing-associated H-X9-DG domain |  | 44-49 | 99.11 |
| TIGR01167 LPXTG cell wall anchor domain |  | 32-48 | 98.87 |
| TIGR03057 X-X-X-Leu-X-X-Gly heptad repeats |  | 49-56 | 97.75 |
| TIGR03501 GlyGly-CTERM domain |  | 36-48 | 97.15 |
| TIGR00756 pentatricopeptide repeat domain |  | 16-23 | 93.83 |
| T24B1.1 | *Caenorhabditis elegans* | 21-52 | 84.13 |
| Occlusion-derived virus envelope protein ODV-E18 |  | 21-62 | 72.05 |
| d.24.1 Pili subunits (54523) SCOP seed sequence: d2pila_ |  | 31-50 | 68.15 |
| RCR |  | 32-50 | 65.14 |
| Cytochrome c550 | *Bacillus subtilis* | 27-61 | 62.79 |
| Occlusion-derived virus envelope protein ODV-E18 |  | 23-55 | 62.79 |
| Prion-like-(Q/N-rich)-domain-bearing protein family member (pqn-90) | *Caenorhabditis elegans* | 34-53 | 61.72 |
| General secretion pathway protein H | *Beggiatoa sp. PS* | 31-50 | 60.83 |
| CytB-hydogenase Ni/Fe-hydrogenase, b-type cytochrome subunit |  | 8-49 | 58.96 |
| Activated in Blocked Unfolded protein response family member (abu-1) | *Caenorhabditis elegans* | 34-53 | 58.69 |
| Alpha defensin |  | 39-50 | 58.16 |
| ComB |  | 6-50 | 57.29 |
| COLlagen family member (col-34) | *Caenorhabditis elegans* | 19-56 | 55.90 |
| Secreted protein | *Beggiatoa sp. PS* | 31-50 | 55.82 |
| COLlagen family member (col-93) | *Caenorhabditis elegans* | 19-56 | 54.87 |
| Serine protease inhibitor |  | 37-90 | 51.90 |
| Prion-like-(Q/N-rich)-domain-bearing protein family member (pqn-78) | *Caenorhabditis elegans* | 34-55 | 49.09 |
| Integral membrane protein | *Streptomyces coelicolor* | 1-55 | 47.76 |
| W06F12.2a | *Caenorhabditis elegans* | 10-52 | 47.72 |
| C17H11.6c | *Caenorhabditis elegans* | 8-59 | 47.62 |
| Methyl-CpG BinDing protein family member (mbd-2) | *Caenorhabditis elegans* | 9-21 | 47.44 |
| RCR |  | 32-50 | 46.40 |
| COLlagen family member (col-91) | *Caenorhabditis elegans* | 31-56 | 45.97 |
| Methyl-CpG binding domain protein 3-like 1 | *Mus musculus* | 9-27 | 44.23 |
| Pleiotrophin family member |  | 34-53 | 43.89 |
| F27E5.3 | *Caenorhabditis elegans* | 31-50 | 43.81 |
| F420-nonreducing hydrogenase II subunit cytochrome B | *Methanosarcina mazei* | 22-50 | 43.78 |
| Crumbs homolog 2 | *Homo sapiens* | 23-53 | 43.61 |
| General secretion pathway protein J | *Yersinia pestis* | 31-50 | 43.53 |
| F26B1.1 | *Caenorhabditis elegans* | 1-49 | 43.50 |
| Glycine rich protein family |  | 35-53 | 43.19 |
| COLlagen family member (col-94) | *Caenorhabditis elegans* | 19-56 | 43.14 |
| Activated in Blocked Unfolded protein response family member (abu-7) | *Caenorhabditis elegans* | 34-55 | 42.68 |
| TetraSPanin family member (tsp-14) | *Caenorhabditis elegans* | 33-109 | 42.50 |
| K08F4.5 | *Caenorhabditis elegans* | 31-50 | 42.44 |
| COLlagen family member (col-92) | *Caenorhabditis elegans* | 19-56 | 42.30 |
| Type II secretion system protein I. |  | 31-52 | 42.13 |
| COLlagen family member (col-139) | *Caenorhabditis elegans* | 19-56 | 42.09 |
| COLlagen family member (col-108) | *Caenorhabditis elegans* | 19-56 | 41.97 |
| F46H6.5 | *Caenorhabditis elegans* | 75-94 | 40.38 |
| COLlagen family member (col-102) | *Caenorhabditis elegans* | 31-56 | 40.22 |
| CG2040-PA, isoform A | *Drosophila melanogaster* | 19-45 | 40.06 |
| Prion-like-(Q/N-rich)-domain-bearing protein family member (pqn-91) | *Caenorhabditis elegans* | 34-55 | 39.77 |
| Type 4 fimbrial biogenesis protein FimT | *Pseudomonas aeruginosa PAO1* | 31-50 | 39.48 |
| T-cell receptor-associated transmembrane adapter 1 |  | 31-48 | 39.41 |
| **I-TASSER** | | | |
| TROPOMYOSIN | *Oryctolagus cuniculus* |  | 1.03 |
| Oligopeptidase | *Geobacillus sp. MO-1* |  | 0.532 |
| Glucose-6-phosphate isomerase | *Brucella melitensis* |  | 0.523 |
| Glucose-6-phosphate isomerase | *Vibrio cholerae* |  | 0.518 |
| Glucose-6-phosphate isomerase | *Plasmodium falciparum* |  | 0.518 |
| Glucose-6-phosphate isomerase | *Sus scrofa* |  | 0.518 |
| Cytochrome P450 107B1 (P450CVIIB1) | *Streptomyces himastatinicus* |  | 0.517 |
| Glucose-6-phosphate isomerase | *Escherichia coli* |  | 0.515 |
| Oligoendopeptidase F | *Geobacillus stearothermophilus* |  | 0.515 |
| Phosphoglucose isomerase | *Geobacillus stearothermophilus* |  | 0.512 |
| **Predict Protein** |  |  |  |
| Protein binding |  | 1,3,4,16, 18,20,24,29, 55,57,81,82 |  |
| Secreted |  |  |  |
| **Atome2** |  |  |  |
| Protein MXIG | *Shigella flexner* |  | 86.27 |
| Protein parD | *Escherichia coli* |  | 76.28 |
| ARF GTPase-activating protein GIT1 | *Rattus norvegicus* |  | 66.40 |
| NifU-like protein, mitochondrial | *Saccharomyces cerevisiae* |  | 43.28 |
| Photosynthetic reaction center C subunit | *Thermochromatium tepidum* |  | 39.97 |
| Lichenicidin VK21 A1 | *Bacillus licheniformis* |  | 38.81 |
| Collagen alpha 1 (heparin binding site) | *Gallus gallus* |  | 38.00 |
| Adenovirus fibre | *Human adenovirus 2* |  | 27.29 |
| Formate dehydrogenase, nitrate-inducible, major subunit | *Escherichia coli* |  | 26.46 |
| Fimbrial protein | *Dichelobacter nodosus* |  | 22.77 |
| Fimbrial protein | *Neisseria gonorrhoeae* |  | 22.40 |
| Fimbrial protein | *Pseudomonas aeruginosa* |  | 20.49 |
| Protein (LCA) | *Homo sapiens* |  | 18.92 |
| Fiber protein 2 | *Human adenovirus 41* |  | 18.06 |

Table S32. *Anodonta anatina* F*-ORF* function predictions

| **Hits** | **Species** | **Position** | **Probability** |
| --- | --- | --- | --- |
| **HHpred** | | | |
| TIGR01167 LPXTG cell wall anchor domain |  | 55-60 | 99.47 |
| TIGR04294 prepilin-type processing-associated H-X9-DG domain |  | 19-22 | 99.31 |
| TIGR03304 outer membrane insertion C-terminal signal |  | 35-36 | 99.24 |
| TIGR03057 X-X-X-Leu-X-X-Gly heptad repeats |  | 47-54 | 98.03 |
| TIGR03501 GlyGly-CTERM domain |  | 9-19 | 97.33 |
| TIGR00756 pentatricopeptide repeat domain |  | 26-46 | 94.32 |
| CG7685-PA | *Drosophila melanogaster* | 4-27 | 91.69 |
| Intra-Golgi v-SNARE | *Saccharomyces cerevisiae* | 2-27 | 86.02 |
| CG13969-PA | *Drosophila melanogaster* | 2-45 | 80.31 |
| TMEM156 protein family |  | 2-28 | 77.90 |
| Ceramidase |  | 3-45 | 74.10 |
| Peptidoglycan-associated lipoprotein Pal | *Yersinia pestis* | 1-20 | 69.76 |
| Saliv_gland_allergen_Aed3 |  | 3-20 | 69.53 |
| Retinoblastoma-associated protein | *Homo sapiens* | 30-47 | 68.54 |
| CG3066-PD, isoform D | *Drosophila melanogaster* | 3-28 | 68.32 |
| Alkaline ceramidase 2 | *Mus musculus* | 2-45 | 66.07 |
| Golgi SNARE BET1-related |  | 4-25 | 65.91 |
| W02F12.2 | *Caenorhabditis elegans* | 2-54 | 65.41 |
| ABC transporter, periplasmic amino acid-binding protein | *Bartonella henselae* | 1-23 | 63.51 |
| Undecaprenyl pyrophosphate phosphatase | *Escherichia coli* | 1-28 | 62.85 |
| Peptidoglycan-associated outer membrane lipoprotein | *Escherichia coli* | 1-20 | 62.73 |
| T27F7.3a | *Caenorhabditis elegans* | 3-37 | 62.11 |
| Cytochrome C-type biogenesis protein CcmE | *Pseudomonas aeruginosa* | 1-43 | 61.79 |
| Sterol reductase/lamin B receptor |  | 20-48 | 61.33 |
| Glycine rich protein family |  | 6-27 | 61.32 |
| SrtB |  | 1-35 | 61.18 |
| Human EMeRin homolog family member (emr-1) | *Caenorhabditis elegans* | 6-24 | 61.00 |
| Related to YPC1 - Alkaline ceramidase |  | 2-45 | 60.98 |
| Periplasmic heme chaperone | *Escherichia coli* | 1-43 | 60.72 |
| Protein transporter | *Arabidopsis thaliana* | 2-27 | 59.33 |
| Signal transduction histidine kinase | *Lactobacillus casei* | 3-37 | 59.20 |
| Essential SNARE protein localized to the ER | *Saccharomyces cerevisiae* | 6-27 | 58.72 |
| LPS export ABC transporter permease LptF |  | 4-30 | 58.57 |
| Syntaxin 5 | *Mus musculus* | 2-24 | 58.16 |
| Vesicle-associated membrane protein-associated protein |  | 5-27 | 57.33 |
| SYP31; t-SNARE | *Arabidopsis thaliana* | 2-24 | 57.00 |
| N-acylsphingosine amidohydrolase 3 | *Homo sapiens* | 2-45 | 55.58 |
| GRP Glycine rich protein family |  | 6-27 | 55.38 |
| Target membrane receptor (t-SNARE) | *Saccharomyces cerevisiae* | 2-24 | 55.04 |
| Alkaline ceramidase that also has reverse (CoA-independent) ceramide synthase activity | *Saccharomyces cerevisiae* | 2-45 | 54.77 |
| Soluble secreted antigen MPT53 precursor | *Mycobacterium tuberculosis* | 1-28 | 54.23 |
| Temporarily Assigned Gene name family member (tag-254) | *Caenorhabditis elegans* | 7-22 | 53.72 |
| Conserved inner membrane protein | *Escherichia coli* | 4-30 | 53.59 |
| Ceramidase |  | 2-45 | 52.04 |
| F59E11.5 | *Caenorhabditis elegans* | 3-32 | 51.37 |
| Cytochrome C-type protein NapC | *Beggiatoa sp. PS* | 2-36 | 50.69 |
| Syntaxin 7 | *Homo sapiens* | 2-24 | 50.07 |
| N-acylsphingosine amidohydrolase 3-like | *Homo sapiens* | 2-45 | 49.95 |
| Syntaxin-related protein required for vacuolar assembly | *Saccharomyces cerevisiae* | 2-24 | 49.79 |
| Y59E9AL.7 | *Caenorhabditis elegans* | 2-25 | 49.43 |
| Rhodanese-like protein | *Beggiatoa sp. PS* | 3-28 | 49.25 |
| PAP2 family protein | *Staphylococcus aureus* | 1-35 | 48.90 |
| Secreted protein | *Beggiatoa sp. PS* | 1-36 | 48.75 |
| Peptidoglycan associated lipoprotein OprL precursor | *Pseudomonas aeruginosa PAO1* | 1-20 | 48.67 |
| Sortase B | *Staphylococcus aureus* | 1-42 | 48.38 |
| SYP125; t-SNARE | *Arabidopsis thaliana* | 7-30 | 47.94 |
| Y41D4B.24 | *Caenorhabditis elegans* | 2-35 | 47.44 |
| CG14084-PB, isoform B | *Drosophila melanogaster* | 2-25 | 47.44 |
| **I-TASSER** | | | |
| Glucokinase regulatory protein | *Homo sapiens* |  | 0.522 |
| Glucokinase regulatory protein | *Xenopus laevis* |  | 0.517 |
| Cation exchanger YfkE | *Bacillus subtilis* |  | 0.512 |
| Pathogenicity island 1 effector protein | *Chromobacterium violaceum* |  | 0.509 |
| Unconventional myosin-Va | *Mus musculus* |  | 0.508 |
| Methane monooxygenase hydroxylase | *Methylosinus trichosporium* |  | 0.505 |
| Inositol-1-monophosphatase | *Mycobacterium tuberculosis* |  | 0.505 |
| **Predict Protein** | | | |
| Protein binding |  | 1-3,6, 32-33, 56-58 |  |
| Mitochondrial membrane |  |  |  |
| **Atome2** | | | |
| Transposon Tn557 toxic shock syndrome toxin-1 | *Staphylococcus aureus* |  | 78.60 |
| S67 | *Sicarius dolichocephalus* |  | 66.70 |
| BirA bifunctional protein | *Escherichia coli* |  | 61.21 |
| SERCA1a | *Oryctolagus cuniculus* |  | 55.57 |
| Sodium/potassium-transporting ATPase subunit alpha-1 | *Sus scrofa* |  | 51.63 |
| Antitoxin RelB3 | *Methanocaldococcus jannaschii* |  | 48.98 |
| Na, K-ATPase alpha subunit | *Squalus acanthias* |  | 46.29 |
| Transcription factor Dp-1 |  |  | 45.74 |
| Nicotinic acetylcholine receptor | *Torpedo californica* |  | 41.21 |
| CPAP | *Danio rerio* |  | 32.69 |
| Potassium-transporting ATPase alpha | *Sus scrofa* |  | 32.06 |
| Vesicle-associated membrane protein 2 | *Rattus norvegicus* |  | 8.39 |

Table S33. *Utterbackia imbecillis* H*-ORF* sequences function predictions

| **Hits** | **Species** | **Position** | **Probability** |
| --- | --- | --- | --- |
| **HHpred** | | | |
| TIGR04294 prepilin-type processing-associated H-X9-DG domain |  | 201-204 | 99.30 |
| TIGR03304 outer membrane insertion C-terminal signal |  | 49-52 | 99.27 |
| TIGR01167 LPXTG cell wall anchor domain |  | 75-77 | 98.89 |
| TIGR03057 X-X-X-Leu-X-X-Gly heptad repeats |  | 21-23 | 97.49 |
| TIGR03501 GlyGly-CTERM domain |  | 49-57 | 96.92 |
| CG12522-PA | *Drosophila melanogaster* | 77-147 | 97.70 |
| CG12522-PA | *Drosophila melanogaster* | 74-137 | 97.65 |
| G protein-coupled receptor 152 | *Homo sapiens* | 1-156 | 96.83 |
| Procyclic acidic repetitive protein (PARP) |  | 80-149 | 96.21 |
| R06C7.4 | *Caenorhabditis elegans* | 75-155 | 96.17 |
| K09E4.6 | *Caenorhabditis elegans* | 79-156 | 95.13 |
| TIGR00756 pentatricopeptide repeat domain |  | 14-21 | 94.13 |
| T14A8.2 | *Caenorhabditis elegans* | 34-155 | 95.01 |
| Related to CSR1 - phosphatidylinositol transfer protein |  | 72-153 | 94.46 |
| K09E4.6 | *Caenorhabditis elegans* | 88-156 | 94.22 |
| T06A4.1b | *Caenorhabditis elegans* | 73-153 | 93.81 |
| Armadillo repeat containing, X-linked 4 | *Homo sapiens* | 75-153 | 93.72 |
| DumPY: shorter than wild-type family member (dpy-10) | *Caenorhabditis elegans* | 1-66 | 93.60 |
| Protein with Tau-Like repeats family member (ptl-1) | *Caenorhabditis elegans* | 74-156 | 93.46 |
| Copper-binding protein | *Methanosarcina mazei* | 11-154 | 93.45 |
| Prolipoprotein diacylglyceryl transferase | *Mycobacterium tuberculosis* | 21-156 | 93.44 |
| Related to CSR1 - phosphatidylinositol transfer protein |  | 75-156 | 92.94 |
| T04F8.8 | *Caenorhabditis elegans* | 85-146 | 92.56 |
| F56B6.4a | *Caenorhabditis elegans* | 74-152 | 92.43 |
| Protein with Tau-Like repeats family member (ptl-1) | *Caenorhabditis elegans* | 75-153 | 92.40 |
| Armadillo repeat containing, X-linked 4 | *Homo sapiens* | 73-157 | 92.39 |
| F56B6.4a | *Caenorhabditis elegans* | 74-156 | 92.37 |
| Junctophilin 2 | *Mus musculus* | 74-212 | 92.12 |
| SphingoMyelin Synthase family member (sms-1) | *Caenorhabditis elegans* | 75-204 | 91.33 |
| Junctophilin 1 | *Mus musculus* | 74-212 | 91.12 |
| BLIstered cuticle family member (bli-2) | *Caenorhabditis elegans* | 12-67 | 91.06 |
| Solute carrier family 16, member 2 | *Homo sapiens* | 75-149 | 91.03 |
| CG1468-PA | *Drosophila melanogaster* | 71-156 | 90.90 |
| Solute carrier family 16, member 2 | *Homo sapiens* | 74-155 | 90.73 |
| CG12316-PA, isoform A | *Drosophila melanogaster* | 84-150 | 90.67 |
| CG12316-PB, isoform B | *Drosophila melanogaster* | 84-150 | 90.67 |
| Eukaryotic translation initiation factor 3, subunit 9 | *Mus musculus* | 76-156 | 90.65 |
| Prolipoprotein diacylglyceryl transferase | *Frankia alni* | 74-157 | 90.55 |
| Eukaryotic translation initiation factor 3, subunit 9 | *Mus musculus* | 69-156 | 90.48 |
| Junctophilin 1 | *Homo sapiens* | 71-212 | 90.44 |
| Adhesion exoprotein | *Lactobacillus casei* | 72-156 | 89.55 |
| F57B1.3 | *Caenorhabditis elegans* | 7-67 | 89.46 |
| Membralin isoform 1 | *Homo sapiens* | 7-156 | 89.25 |
| Protein with Tau-Like repeats family member (ptl-1) | *Caenorhabditis elegans* | 74-156 | 88.92 |
| Diacylglycerol kinase kappa | *Homo sapiens* | 73-156 | 88.91 |
| F47B8.5 | *Caenorhabditis elegans* | 74-155 | 88.65 |
| T10E10.5 | *Caenorhabditis elegans* | 12-67 | 88.21 |
| SQuaT family member (sqt-2) | *Caenorhabditis elegans* | 12-63 | 87.68 |
| CG1468-PA | *Drosophila melanogaster* | 74-156 | 87.29 |
| DumPY: shorter than wild-type family member (dpy-14) | *Caenorhabditis elegans* | 20-67 | 87.27 |
| Prolipoprotein diacylglyceryl transferase | *Frankia alni* | 74-153 | 87.09 |
| Diacylglycerol kinase kappa | *Homo sapiens* | 72-156 | 86.90 |
| V-set and immunoglobulin domain containing 1 | *Mus musculus* | 74-155 | 86.33 |
| Protein with Tau-Like repeats family member (ptl-1) | *Caenorhabditis elegans* | 75-156 | 85.78 |
| T10G3.1 | *Caenorhabditis elegans* | 73-154 | 85.56 |
| Adhesion exoprotein | *Lactobacillus casei* | 76-156 | 84.94 |
| R09F10.3 | *Caenorhabditis elegans* | 72-156 | 84.49 |
| F33A8.9 | *Caenorhabditis elegans* | 10-67 | 84.46 |
| COLlagen family member (col-110) | *Caenorhabditis elegans* | 21-68 | 84.41 |
| D1054.11 | *Caenorhabditis elegans* | 72-156 | 84.32 |
| Y54E10BL.2 | *Caenorhabditis elegans* | 22-67 | 84.00 |
| COLlagen family member (col-102) | *Caenorhabditis elegans* | 25-67 | 83.99 |
| PF70 protein | *Plasmodium falciparum* | 74-154 | 83.94 |
| C09F9.2 | *Caenorhabditis elegans* | 72-153 | 83.50 |
| F11G11.11 | *Caenorhabditis elegans* | 16-67 | 83.21 |
| **BLASTP, PSIBLAST** | | | |
| Bv80/Bb-1, partial | *Babesia bovis* | 77-152 | 2e-10 |
| S-layer protein precursor | *Bacillus thuringiensis* | 73-152 | 6e-09 |
| Cell surface protein, partial | *Bacillus thuringiensis* | 73-152 | 7e-09 |
| 85 kDa protein | *Babesia bovis* | 77-152 | 3e-08 |
| Bv80/Bb-1, partial **(3)** | *Babesia bovis* | 76-132 | 1e-07 |
| Cell surface protein **(2)** | *Bacillus thuringiensis* | 75-152 | 1e-07 |
| 85 kDa protein **(2)** | *Babesia bovis* | 76-182 | 1e-06 |
| LdOrf-129 peptide | *Lymantria dispar* | 74-144 | 2e-06 |
| ORF-132 protein | *Lymantria dispar* | 74-131 | 4e-06 |
| GH24581 | *Drosophila grimshawi* | 79-143 | 6e-05 |
| Type I restriction modification protein | *Mycoplasma pneumoniae* | 76-157 | 2e-04 |
| Restriction endonuclease, S subunit | *Mycoplasma pneumoniae* | 70-157 | 0.005 |
| Type I restriction modification protein | *Mycoplasma pneumoniae* | 70-157 | 0.005 |
| Restriction endonuclease, S subunit | *Mycoplasma pneumoniae* | 62-157 | 0.007 |
| **PSIBLAST** | | | |
| Protein B602L, partial | *Columba livia* | 76-154 | 6e-11 |
| Bv80, partial | *Babesia bovis* | 76-132 | 3e-08 |
| ORF-126 protein | *Lymantria dispar* | 72-140 | 4e-08 |
| B602L, partial **(2)** | *African swine fever virus* | 60-148 | 5e-08 |
| Central variable region protein **(2)** | *African swine fever virus* | 60-154 | 6e-08 |
| Central variable region protein | *African swine fever virus* | 60-130 | 7e-08 |
| pB602L | *African swine fever virus* | 60-132 | 8e-08 |
| Bv80, partial | *Babesia bovis* | 76-152 | 8e-08 |
| 9RL protein | *African swine fever virus* | 65-153 | 8e-08 |
| B602L, partial | *African swine fever virus* | 60-148 | 1e-07 |
| 9RL, partial **(3)** | *African swine fever virus* | 60-129 | 1e-07 |
| Bv80, partial **(3)** | *Babesia bovis* | 80-132 | 2e-07 |
| Response regulator receiver domain protein (CheY-like) | *Nodularia spumigena* | 70-136 | 2e-07 |
| B602L protein | *African swine fever virus* | 66-153 | 3e-07 |
| 9RL protein **(2)** | *African swine fever virus* | 60-154 | 3e-07 |
| U1 | *Hyposoter didymator ichnovirus* | 77-138 | 3e-07 |
| Pathway-specific nitrogen regulator | *Metarhizium guizhouense* | 68-132 | 4e-07 |
| 9RL protein | *African swine fever virus* | 65-136 | 4e-07 |
| B602L protein, partial **(3)** | *African swine fever virus* | 65-169 | 4e-07 |
| BV80 merozoite protein | *Babesia bovis* | 76-152 | 5e-07 |
| Translation initiation factor eIF2B | *Metarhizium robertsii* | 68-129 | 8e-06 |
| Pathway-specific nitrogen regulator, partial | *Metarhizium brunneum* | 68-129 | 9e-06 |
| Mucin | *Trichomonas vaginalis* | 71-140 | 1e-05 |
| Cell surface protein **(2)** | *Bacillus thuringiensis* | 23-152 | 1e-05 |
| S-layer protein | *Bacillus thuringiensis* | 23-152 | 1e-05 |
| Outer membrane autotransporter barrel domain-containing protein | *Escherichia coli* | 68-124 | 2e-05 |
| Central variable region protein | *African swine fever virus* | 60-134 | 2e-05 |
| Ribonuclease E | *Nitrincola lacisaponensis* | 77-146 | 4e-05 |
| Elicitin-like protein 6 precursor, partial | *Phytophthora medicaginis* | 74-150 | 1e-04 |
| Transcription factor IIIB 50 kDa subunit | *Xenopus tropicalis* | 76-130 | 5e-04 |
| Involucrin repeat protein | *Ophiostoma piceae* | 74-136 | 6e-04 |
| Peptidase | *Actinoplanes sp. SE50/110* | 77-127 | 6e-04 |
| Prokaryotic cytochrome b561 family protein | *Burkholderia pseudomallei* | 76-127 | 0.001 |
| B-type cytochrome | *Burkholderia pseudomallei* | 76-127 | 0.001 |
| Thylakoid rhodanese-like protein | *Medicago truncatula* | 73-132 | 0.001 |
| Cell division protein FtsK | *Carnobacterium sp. 17-4* | 79-179 | 0.003 |
| Autotransporter protein, partial | *Escherichia coli* | 73-122 | 0.003 |
| **I-TASSER** | | | |
| Survival motor neuron protein **(3)** | *Homo sapiens* |  | 1.14-1.81 |
| Type I hyperactive antifreeze protein | *Pseudopleuronectes americanus* |  | 2.21 |
| Myc box dependent interacting protein 1**(2)** | *Homo sapiens* |  | 1.00-1.90 |
| Accumulation associated protein **(3)** | *Staphylococcus epidermidis* |  | 1.13-1.97 |
| HIV-1 capsid | *Human immunodeficiency virus 1* |  | 0.513 |
| Gag Polyprotein | *Human immunodeficiency virus 1* |  | 0.510 |
| Capsid protein P24 | *Human immunodeficiency virus 2* |  | 0.504 |
| **Predict Protein** | | | |
| Protein binding |  | 1,221,222 |  |
| Secreted |  |  |  |
| Diacylglycerol kinase kappa **(3)** | *Homo sapiens* |  | 5e-04-0.002 |
| Proteoglycan 4 **(2)** | *Homo sapiens* |  | 0.17, 0.99 |
| Proteoglycan 4 **(9)** | *Mus musculus* |  | 1e-08- 0.002 |
| **Atome2** | | | |
| Lamin A/C | *Homo sapiens* |  | 92.75 |
| Protein bicaudal D | *Drosophila melanogaster* |  | 84.72 |
| 80 kDa MCM3-associated protein | *Homo sapiens* |  | 76.71 |
| Selenoprotein S | *Homo sapiens* |  | 72.30 |
| Heat shock protein | *Saccharomyces cerevisiae* |  | 50.07 |
| Nucleoprotein | *Andes virus* |  | 44.18 |
| Herpes simplex virus protein ICP47 | *Herpes simplex virus 1* |  | 37.48 |
| Cupiennin-1a | *Cupiennius salei* |  | 35.85 |
| RNA-binding protein 5 | *Homo sapiens* |  | 29.47 |

Table S34. *Margaritifera falcata* H*-ORF* sequences function predictions

| **Hits** | **Species** | **Position** | **Probability** |
| --- | --- | --- | --- |
| **HHpred** | | | |
| TIGR03304 outer membrane insertion C-terminal signal |  | 5-7 | 99.23 |
| TIGR04294 prepilin-type processing-associated H-X9-DG domain |  | 5-8 | 99.16 |
| TIGR01167 LPXTG cell wall anchor domain |  | 43-59 | 98.88 |
| TIGR03057 X-X-X-Leu-X-X-Gly heptad repeats |  | 60-67 | 97.66 |
| TIGR03501 GlyGly-CTERM domain |  | 47-59 | 96.99 |
| TIGR00756 pentatricopeptide repeat domain |  | 23-27 | 93.25 |
| T24B1.1 | *Caenorhabditis elegans* | 21-63 | 91.85 |
| Occlusion-derived virus envelope protein ODV-E18 |  | 33-73 | 74.97 |
| d.24.1 Pili subunits (54523) SCOP seed sequence: d1oqwa_ |  | 42-61 | 74.24 |
| Serine protease inhibitor |  | 48-107 | 71.24 |
| Chitin synthesis regulation, resistance to Congo red |  | 43-61 | 70.62 |
| Activator of basal transcription 1 | *Homo sapiens* | 5-33 | 69.40 |
| Occlusion-derived virus envelope protein ODV-E18 |  | 34-73 | 68.30 |
| CG17785-PA | *Drosophila melanogaster* | 16-65 | 68.04 |
| General secretion pathway protein H | *Beggiatoa sp. PS* | 42-61 | 67.36 |
| Prion-like-(Q/N-rich)-domain-bearing protein family member (pqn-90) | *Caenorhabditis elegans* | 45-64 | 63.72 |
| Secreted protein | *Beggiatoa sp. PS* | 42-61 | 61.88 |
| CG32708-PA | *Drosophila melanogaster* | 5-33 | 61.30 |
| Activator of basal transcription | *Mus musculus* | 5-33 | 59.95 |
| COLlagen family member (col-93) | *Caenorhabditis elegans* | 29-67 | 57.32 |
| Alpha defensin |  | 50-61 | 57.29 |
| COLlagen family member (col-34) | *Caenorhabditis elegans* | 30-67 | 56.89 |
| C17H11.6c | *Caenorhabditis elegans* | 20-70 | 56.74 |
| Cytochrome c550 | *Bacillus subtilis* | 38-72 | 56.20 |
| RCR |  | 43-61 | 52.93 |
| ComB |  | 18-61 | 52.89 |
| Prion-like-(Q/N-rich)-domain-bearing protein family member (pqn-2) | *Caenorhabditis elegans* | 45-66 | 52.79 |
| COLlagen family member (col-91) | *Caenorhabditis elegans* | 42-67 | 51.23 |
| UCP036704 |  | 29-35 | 51.11 |
| TATA-binding protein binding **(2)** | *Arabidopsis thaliana* | 5-33 | 50.96 |
| General secretion pathway protein J | *Yersinia pestis* | 42-61 | 50.70 |
| Prion-like-(Q/N-rich)-domain-bearing protein family member (pqn-78) | *Caenorhabditis elegans* | 45-66 | 50.59 |
| Essential nucleolar protein involved in pre-18S rRNA processing | *Saccharomyces cerevisiae* | 5-33 | 49.71 |
| CG6999-PA | *Drosophila melanogaster* | 5-33 | 49.32 |
| T-cell receptor-associates transmembrane adapter 1 |  | 42-59 | 49.10 |
| Thymidine kinase **(2)** | *Herpes virus* | 23-34 | 48.70 |
| K08F4.5 | *Caenorhabditis elegans* | 42-61 | 48.36 |
| Essential cell division protein | *Escherichia coli* | 36-85 | 48.20 |
| GRP: Glycine rich protein family |  | 46-64 | 47.31 |
| FAST kinase-like protein, subdomain 1 |  | 5-54 | 46.87 |
| COLlagen family member (col-94) | *Caenorhabditis elegans* | 29-67 | 46.72 |
| TIGR03544 DivIVA domain | *Bacillus subtilis* | 20-35 | 51.87 |
| Activated in Blocked Unfolded protein response family member (abu-7) | *Caenorhabditis elegans* | 45-66 | 45.83 |
| PulG Type II secretory pathway, pseudopilin PulG |  | 42-61 | 45.03 |
| COLlagen family member (col-139) | *Caenorhabditis elegans* | 30-67 | 44.54 |
| F27E5.3 | *Caenorhabditis elegans* | 42-61 | 44.44 |
| Light-harvesting complex subunits | *Rhodospirillum rubrum* | 39-63 | 43.91 |
| General secretion pathway protein G | *Beggiatoa sp. PS* | 42-61 | 43.24 |
| COLlagen family member (col-92) | *Caenorhabditis elegans* | 29-67 | 43.10 |
| General secretion pathway protein H | *Beggiatoa sp. PS* | 42-61 | 43.05 |
| CG32706-PA | *Drosophila melanogaster* | 5-33 | 42.51 |
| ABC transporter, permease protein | *Methanosarcina mazei* | 11-64 | 42.42 |
| Light-harvesting complex subunits | *Rhodospirillum rubrum* | 39-63 | 41.47 |
| Prion-like-(Q/N-rich)-domain-bearing protein family member (pqn-91) | *Caenorhabditis elegans* | 45-66 | 41.32 |
| PulG: Type II secretory pathway pseudopilin |  | 42-61 | 41.30 |
| F420-nonreducing hydrogenase, subunit cytochrome B | *Methanosarcina mazei* | 33-61 | 41.29 |
| EGF-like-domain, multiple 9 **(2)** | *Homo sapiens* | 45-62 | 40.73 |
| Y45G12B.2a | *Caenorhabditis elegans* | 37-62 | 40.38 |
| **I-TASSER** | | | |
| Tropomyosin | *Oryctolagus cuniculus* |  | 1.12 |
| Nck-associated protein 1 | *Homo sapiens* |  | 0.511 |
| **Predict Protein** | | | |
| Protein binding |  | 16,19,27,31,34-37,60,61,64,92 |  |
| Secreted |  |  |  |
| **Atome2** | | | |
| 39 kDa initiator binding protein | *Trichomonas vaginalis* |  | 67.83 |
| Octamer-binding transcription factor 1 | *Homo sapiens* |  | 59.87 |
| BA3-type cytochrome-c oxidase | *Thermus thermophilus* |  | 55.07 |
| Electron transfer flavoprotein-ubiquinone oxidoreductase | *Sus scrofa* |  | 50.03 |
| Degenerin mec-4 | *Caenorhabditis elegans* |  | 48.90 |
| Cytochrome b-c1 complex subunit 1, mitochondrial | *Saccharomyces cerevisiae* |  | 28.76 |
| Integrin alpha-IIb | *Homo sapiens* |  | 28.20 |
| Fimbrial protein | *Neisseria gonorrhoeae* |  | 23.71 |
| Fimbrial protein | *Pseudomonas aeruginosa* |  | 21.80 |
| Fimbrial protein | *Dichelobacter nodosus* |  | 21.07 |
| Cycloviolacin O14 | *Viola odorata* |  | 17.54 |
| Histone peptide | *Homo sapiens* |  | 15.68 |
| Cytochrome c oxidase subunit 1 | *Thermus thermophilus* |  | 13.57 |

Table S35. *Toxolasma lividus* H*-ORF* function predictions

| **Hits** | **Species** | **Position** | **Probability** |
| --- | --- | --- | --- |
| **HHpred** | | | |
| TIGR01167 LPXTG cell wall anchor domain |  | 24-41 | 99.01 |
| TIGR04294 prepilin-type processing-associated H-X9-DG domain |  | 134-135 | 98.98 |
| TIGR03304 outer membrane insertion C-terminal signal |  | 40-44 | 98.46 |
| TIGR03057 X-X-X-Leu-X-X-Gly heptad repeats |  | 189-194 | 97.44 |
| TIGR03501 GlyGly-CTERM domain |  | 33-44 | 96.69 |
| TIGR00756 pentatricopeptide repeat domain |  | 17-23 | 93.27 |
| CG17248-PA, isoform A | *Drosophila melanogaster* | 16-81 | 91.85 |
| CG17248-PC, isoform C | *Drosophila melanogaster* | 16-81 | 91.85 |
| CG17248-PE, isoform E | *Drosophila melanogaster* | 16-81 | 90.04 |
| CG17248-PB, isoform B | *Drosophila melanogaster* | 16-81 | 90.04 |
| G-protein-linked Acetylcholine Receptor family member (gar-1) | *Caenorhabditis elegans* | 6-52 | 84.25 |
| sensor protein | *Nostoc punctiforme* | 2-43 | 81.29 |
| cAMP responsive element binding protein 3-like 2 | *Mus musculus* | 8-50 | 80.14 |
| CG33517-PB, isoform B | *Drosophila melanogaster* | 6-52 | 78.13 |
| Vesicle-associated membrane protein 2 (synaptobrevin 2) | *Homo sapiens* | 16-43 | 78.05 |
| CG3856-PC, isoform C | *Drosophila melanogaster* | 8-52 | 77.12 |
| CG3856-PA, isoform A | *Drosophila melanogaster* | 8-52 | 77.12 |
| CG33517-PC, isoform C | *Drosophila melanogaster* | 8-52 | 76.96 |
| Histamine receptor-related G-protein coupled receptor |  | 8-52 | 76.91 |
| K08F4.5 | *Caenorhabditis elegans* | 20-43 | 76.75 |
| ATGLR2.4 | *Arabidopsis thaliana* | 4-39 | 76.65 |
| Septation ring formation regulator EzrA | *Staphylococcus aureus* | 20-42 | 76.59 |
| ABC Transporter | *Sulfolobus solfataricus* | 9-59 | 76.52 |
| C-type LECtin family member (clec-39) | *Caenorhabditis elegans* | 8-47 | 76.41 |
| G-protein-linked Acetylcholine Receptor family member (gar-2) | *Caenorhabditis elegans* | 3-52 | 75.76 |
| Syndecan 3 | *Mus musculus* | 10-42 | 75.43 |
| G-protein-linked Acetylcholine Receptor family member (gar-2) | *Caenorhabditis elegans* | 6-52 | 74.50 |
| Integral membrane sensor signal transduction histidine kinase | *Nostoc punctiforme* | 14-44 | 74.45 |
| F02E9.7 | *Caenorhabditis elegans* | 3-49 | 74.18 |
| Dentin matrix protein 1 | *Mus musculus* | 32-49 | 73.94 |
| COLlagen family member (col-77) | *Caenorhabditis elegans* | 8-42 | 73.16 |
| C17H11.6c | *Caenorhabditis elegans* | 1-52 | 73.01 |
| CG4356-PA, isoform A | *Drosophila melanogaster* | 6-63 | 72.19 |
| TonB family protein | *Nostoc punctiforme* | 1-45 | 71.31 |
| Membrane-bound protease FTSH (cell division protein) | *Mycobacterium tuberculosis* | 14-40 | 71.19 |
| DumPY: shorter than wild-type family member (dpy-2) | *Caenorhabditis elegans* | 4-42 | 70.87 |
| Histidine kinase | *Nitrosopumilus maritimus* | 13-42 | 70.83 |
| Y26D4A.6 | *Caenorhabditis elegans* | 28-49 | 70.46 |
| CG16720-PB, isoform B | *Drosophila melanogaster* | 8-52 | 69.15 |
| CG16720-PA, isoform A | *Drosophila melanogaster* | 8-52 | 69.15 |
| C49D10.10 | *Caenorhabditis elegans* | 14-48 | 68.66 |
| G-protein-linked Acetylcholine Receptor family member (gar-2) | *Caenorhabditis elegans* | 8-52 | 68.38 |
| Phosphonate ABC transporter | *Nostoc punctiforme* | 13-37 | 68.13 |
| T06E4.6 | *Caenorhabditis elegans* | 19-42 | 66.32 |
| Syntaxin |  | 8-41 | 66.31 |
| CG18208-PA | *Drosophila melanogaster* | 6-52 | 65.73 |
| Cuticle protein |  | 30-49 | 65.60 |
| LCR9 | *Arabidopsis thaliana* | 19-39 | 65.51 |
| Cation efflux system protein czcA-1 | *Synechococcus sp. CC9311* | 17-72 | 65.27 |
| Extracellular solute-binding protein | *Thermofilum pendens* | 14-38 | 64.33 |
| T06E4.8 | *Caenorhabditis elegans* | 14-61 | 64.21 |
| COLlagen family member (col-77) | *Caenorhabditis elegans* | 6-40 | 63.74 |
| Cytochrome c-550 | *Nostoc punctiforme* | 19-40 | 63.62 |
| DOPamine receptor family member (dop-3) | *Caenorhabditis elegans* | 1-52 | 63.59 |
| N-terminal TM domain of oligopeptide transport permease C |  | 30-69 | 63.54 |
| K08F4.5 | *Caenorhabditis elegans* | 26-47 | 62.68 |
| Structural constituent of cell wall | *Arabidopsis thaliana* | 4-71 | 62.58 |
| Glycophorin |  | 23-43 | 62.12 |
| Vesicle-associated membrane protein 8 | *Mus musculus* | 4-45 | 62.00 |
| High affinity copper uptake protein 1 | *Homo sapiens* | 32-43 | 61.97 |
| Cell-division initiation protein | *Bacillus subtilis* | 8-43 | 61.88 |
| C44C10.1 | *Caenorhabditis elegans* | 8-40 | 61.69 |
| COLlagen family member (col-173) | *Caenorhabditis elegans* | 5-44 | 60.97 |
| CG9778-PA | *Drosophila melanogaster* | 20-78 | 60.97 |
| Molybdate-binding protein | *Methanosarcina mazei* | 12-43 | 60.58 |
| Vesicle-associated membrane protein 1 isoform 1 | *Homo sapiens* | 16-43 | 60.32 |
| LCR5 | *Arabidopsis thaliana* | 19-39 | 60.27 |
| serine protease SplA | *Staphylococcus aureus* | 14-33 | 60.15 |
| RNase_BN |  | 6-44 | 60.11 |
| Activated in Blocked Unfolded protein response family member (abu-11) | *Caenorhabditis elegans* | 32-44 | 60.05 |
| Sulfate ABC transporter, periplasmic sulfate-binding protein | *Nostoc punctiforme* | 5-36 | 59.68 |
| Cholinergic receptor, muscarinic 3 | *Homo sapiens* | 8-52 | 59.63 |
| DOPamine receptor family member (dop-2) | *Caenorhabditis elegans* | 6-52 | 59.47 |
| DOPamine receptor family member (dop-3) | *Caenorhabditis elegans* | 10-52 | 59.43 |
| **I-TASSER** | | | |
| Survival motor neuron protein | *Homo sapiens* |  | 1.97, 1.36 |
| Type I hyperactive antifreeze protein | *Pseudopleuronectes americanus* |  | 2.63, 1.18 |
| Myc box dependent interacting protein 1 | *Homo sapiens* |  | 1.39 |
| Accumulation associated protein | *Staphylococcus epidermidis* |  | 1.65 |
| Major vault protein | *Rattus norvegicus* |  | 1.61 |
| Type I hyperactive antifreeze protein | *Pseudopleuronectes americanus* |  | 0.716 |
| Dynamin family protein | *Nostoc punctiforme* |  | 0.606 |
| Tyrosine-protein kinase Fes/Fps | *Homo sapiens* |  | 0.563 |
| Interferon-induced guanylate-binding protein 1 | *Homo sapiens* |  | 0.559 |
| BAI1-associated protein 2 isoform 1 | *Homo sapiens* |  | 0.546 |
| Phospholipase C beta | *Meleagris gallopavo* |  | 0.544 |
| Brain-specific angiogenesis inhibitor 1-associated protein 2-like protein 2 | *Mus musculus* |  | 0.539 |
| TcdA1 | *Photorhabdus luminescens* |  | 0.537 |
| **Predict Protein** | |  |  |
| Protein binding |  | 1-2 |  |
| Secreted |  |  |  |
| Cell surface protein **(5)** | *Bacillus cereus* |  | 1e-06-5e-05 |
| DNA-directed RNA polymerase II subunit RPB1 | *Homo sapiens* |  | 0.30 |
| DNA-directed RNA polymerase II subunit RPB1 | *Mus musculus* |  | 0.29 |
| Agglutinin receptor | *Streptococcus gordonii* |  | 0.28 |
| Muscle M-line assembly protein unc-89 | *Caenorhabditis elegans* |  |  |
| **Atome2** | | | |
| Glucose-1-phosphate thymidylyl transferase | *Pseudomonas aeruginosa* |  | 72.22 |
| VPU **(3)** | *Human immunodeficiency virus 1* |  | 33.16-27.03 |
| GDP-mannose mannosyl hydrolase | *Escherichia coli* |  | 30.48 |

Table S36. *Lasmigona compressa* H*-ORF* sequences function predictions

| **Hits** | **Species** | **Position** | **Probability** |
| --- | --- | --- | --- |
| **HHpred** | | | |
| TIGR04294 prepilin-type processing-associated H-X9-DG domain |  | 17-20 | 99.15 |
| TIGR01167 LPXTG cell wall anchor domain |  | 2-13 | 98.90 |
| TIGR03304 outer membrane insertion C-terminal signal |  | 23-24 | 98.77 |
| TIGR03057 X-X-X-Leu-X-X-Gly heptad repeats |  | 79-92 | 97.64 |
| TIGR03501 GlyGly-CTERM domain |  | 2-12 | 96.49 |
| TIGR00756 pentatricopeptide repeat domain |  | 14-34 | 93.03 |
| C53B4.8 | *Caenorhabditis elegans* | 75-198 | 87.53 |
| C53B4.8 | *Caenorhabditis elegans* | 52-196 | 86.53 |
| W02B8.6 | *Caenorhabditis elegans* | 53-198 | 71.92 |
| W02B8.6 | *Caenorhabditis elegans* | 76-198 | 70.34 |
| W02B8.3 | *Caenorhabditis elegans* | 51-204 | 56.63 |
| F32A11.7 | *Caenorhabditis elegans* | 30-198 | 56.42 |
| TonB family protein | *Nostoc punctiforme* | 2-25 | 50.10 |
| W02B8.3 | *Caenorhabditis elegans* | 51-198 | 49.98 |
| RNA binding/nucleic acid binding | *Arabidopsis thaliana* | 55-207 | 46.78 |
| W02B8.4 | *Caenorhabditis elegans* | 53-198 | 42.83 |
| Sterol reductase/lamin B receptor |  | 7-36 | 40.81 |
| Related to CHS7 - control of protein export from the ER (like chitin synthase III) |  | 3-27 | 40.45 |
| COLlagen family member (col-102) | *Caenorhabditis elegans* | 1-35 | 40.42 |
| Neuropeptide-Like Protein family member (nlp-16) | *Caenorhabditis elegans* | 1-17 | 40.23 |
| RNA binding/nucleic acid binding | *Arabidopsis thaliana* | 48-202 | 39.87 |
| Delta-notch-like EGF repeat-containing transmembrane | *Homo sapiens* | 1-101 | 39.77 |
| W02B8.4 | *Caenorhabditis elegans* | 76-196 | 38.59 |
| F19H8.4 | *Caenorhabditis elegans* | 75-196 | 35.09 |
| Defensin, beta 104B precursor **(2)** | *Homo sapiens* | 1-24 | 33.93 |
| Chs3p: Chitin synthase III catalytic subunit **(2)** |  | 3-34 | 33.08 |
| F58A4.1 | *Caenorhabditis elegans* | 2-10 | 29.62 |
| Y41C4A.19 | *Caenorhabditis elegans* | 1-35 | 27.84 |
| Phosphatidylinositol glycan, class B | *Homo sapiens* | 2-25 | 27.40 |
| Subunit X of cytochrome bc1 complex | *Saccharomyces cerevisiae* | 1-25 | 25.83 |
| CTAGE family, member 5 isoform 1 | *Homo sapiens* | 1-29 | 25.31 |
| Plasmodium falciparum S-antigen |  | 1-19 | 24.47 |
| RCR |  | 1-19 | 24.31 |
| B0379.7 | *Caenorhabditis elegans* | 2-19 | 24.10 |
| CG7685-PA | *Drosophila melanogaster* | 2-30 | 23.18 |
| CG8764-PA | *Drosophila melanogaster* | 2-25 | 22.39 |
| Sar8.2 family |  | 1-42 | 22.36 |
| Subunit 9 of the ubiquinol cytochrome-c reductase complex | *Saccharomyces cerevisiae* | 1-25 | 22.29 |
| Ergosterol biosynthesis ERG4/ERG24 family |  | 8-36 | 22.13 |
| C35A5.4 | *Caenorhabditis elegans* | 47-61 | 22.03 |
| Activated in Blocked Unfolded protein response family member (abu-11) | *Caenorhabditis elegans* | 5-29 | 21.87 |
| T26E3.1 | *Caenorhabditis elegans* | 1-11 | 21.62 |
| SRB6 |  | 11-30 | 21.50 |
| SH3 type 3 domain-containing protein | *Nostoc punctiforme* | 2-21 | 21.10 |
| DumPY: shorter than wild-type family member (dpy-5) | *Caenorhabditis elegans* | 1-30 | 21.03 |
| Transmembrane protein | *Mycobacterium tuberculosis* | 1-31 | 20.90 |
| Cytochrome b-c1 complex subunit 9 | *Saccharomyces cerevisiae* | 1-25 | 20.65 |
| PVC2 |  | 1-34 | 20.55 |
| C04H5.7 | *Caenorhabditis elegans* | 2-23 | 20.51 |
| F29B9.9 | *Caenorhabditis elegans* | 1-25 | 20.18 |
| Transcriptional regulator, XRE family | *Beggiatoa sp. PS* | 10-32 | 20.10 |
| Ubiquinol-cytochrome c reductase complex 7.2kDa protein isoform a | *Homo sapiens* | 2-25 | 20.01 |
| **BLASTP/PSIBLAST** |  |  |  |
| Viral protein TPX | *Histoplasma capsulatum* | 39-201 | 1e-10 |
| Proteoglycan | *Histoplasma capsulatum* | 32-199 | 6e-10, 5e-09 |
| Histone-lysine N-methyltransferase ATXR3 | *Medicago truncatula* | 77-199 | 1e-04 |
| Histone-lysine N-methyltransferase E(z) | *Medicago truncatula* | 77-199 | 1e-04 |
| Adhesin | *Rahnella aquatilis* | 43-192 | 2e-04 |
| BNIP2 motif containing molecule at the carboxyl terminal region 1-like protein | *Camelus ferus* | 35-191 | 3e-04 |
| Chitinase III | *Acanthocheilonema viteae* | 35-199 | 0.001 |
| **BLASTP** | | | |
| CRE-CLEC-85 protein | *Caenorhabditis remanei* | 41-207 | 0.033 |
| YadA domain-containing protein | *Rahnella sp. Y9602* | 49-192 | 0.075 |
| Phage protein | *Methanosarcina vacuolata* | 37-191 | 0.085 |
| GG21511 | *Drosophila erecta* | 49-200 | 0.13 |
| Glycosyl transferase family 1 | *Myxococcus sp.* | 72-197 | 0.67 |
| **PSIBLAST** | | | |
| Quinolinate phosphoribosyl transferase | *Burkholderia sp. MSh2* | 56-191 | 0.001 |
| **I-TASSER** | | | |
| Myeloma immunoglobulin D delta | *Homo sapiens* |  | 1.39 |
| Survival motor neuron protein | *Homo sapiens* |  | 2.59 |
| Type I hyperactive antifreeze protein | *Pseudopleuronectes americanus* |  | 2.47 |
| Myc box dependent interacting protein 1 | *Homo sapiens* |  | 1.28, 2.16 |
| Accumulation associated protein | *Staphylococcus sp.* |  | 2.24 |
| Long tail fiber protein P37 | *Enterobacteria phage T4* |  | 1.01 |
| Survival motor neuron protein | *Homo sapiens* |  | 0.805 |
| **Predict Protein** | | | |
| Protein binding |  | 1,186,187,206 |  |
| Secreted |  |  |  |
| DNA-directed RNA polymerase **(7)** | *Babesia bigemina* |  | 6e-20 |
| DNA-directed RNA polymerase **(6)** | *Phaeodactylum tricornutum* |  | 2e-20 |
| DNA-directed RNA polymerase **(9)** | *Phytophthora ramorum* |  | 4e-20 |
| Paternally-expressed gene 3 protein **(12)** | *Bos taurus* |  | 1e-08 |
| DNA-directed RNA polymerase II subunit RPB1 **(3)** | *Caenorhabditis elegans* |  | 8e-11 |
| DNA-directed RNA polymerase II subunit RPB1 **(6)** | *Homo sapiens* |  | 1e-09 |
| DNA-directed RNA polymerase II subunit RPB1 **(6)** | *Mus musculus* |  | 2e-09 |
| DNA-directed RNA polymerase **(2)** | *Aphanomyces astaci* |  | 1e-19 |
| **Atome2** | | | |
| Photosystem II: Subunit PsbA | *Thermosynechococcus vulcanus* |  | 75.97 |
| Cytosolic leucyl-tRNA synthetase | *Candida albicans* |  | 51.50 |
| Cytochrome b6 **(3)** | *Mastigocladus laminosus* |  | 37.79 |

Table S37. *Lasmigona subviridis* H*-ORF* sequences function predictions

| **Hits** | **Species** | **Position** | **Probability** |
| --- | --- | --- | --- |
| **HHpred** | | | |
| TIGR03304 outer membrane insertion C-terminal signal |  | 1-5 | 99.24 |
| TIGR04294 prepilin-type processing-associated H-X9-DG domain |  | 34-37 | 99.14 |
| TIGR01167 LPXTG cell wall anchor domain |  | 14-30 | 99.03 |
| TIGR03057 X-X-X-Leu-X-X-Gly heptad repeats |  | 95-101 | 97.61 |
| TIGR03501 GlyGly-CTERM domain |  | 14-24 | 96.98 |
| CG7685-PA | *Drosophila melanogaster* | 5-32 | 94.64 |
| TIGR00756 pentatricopeptide repeat domain |  | 31-51 | 92.73 |
| F32A11.7 | *Caenorhabditis elegans* | 53-187 | 93.21 |
| W02B8.6 | *Caenorhabditis elegans* | 60-188 | 93.18 |
| MoLTing defective family member (mlt-10) **(2)** | *Caenorhabditis elegans* | 65-188 | 92.62 |
| W02B8.4 | *Caenorhabditis elegans* | 53-188 | 91.89 |
| W02B8.6 | *Caenorhabditis elegans* | 66-188 | 91.77 |
| F32A11.7 | *Caenorhabditis elegans* | 66-186 | 91.00 |
| W02B8.4 | *Caenorhabditis elegans* | 66-188 | 88.18 |
| W02B8.3 | *Caenorhabditis elegans* | 60-188 | 84.25 |
| TMEM156 protein family |  | 7-33 | 83.15 |
| W02B8.3 | *Caenorhabditis elegans* | 103-188 | 80.80 |
| Conserved inner membrane protein | *Escherichia coli* | 1-35 | 80.68 |
| Sensory box histidine kinase PhoR | *Staphylococcus aureus* | 7-42 | 79.66 |
| SrtB |  | 5-40 | 78.93 |
| Sensor histidine kinase | *Streptococcus pneumoniae* | 7-42 | 78.59 |
| LptF_YjgP LPS export ABC transporter permease LptF. |  | 5-35 | 78.20 |
| Glycine rich protein family |  | 11-29 | 72.98 |
| CG11020-PA, isoform A | *Drosophila melanogaster* | 7-44 | 72.67 |
| CbiN ABC-type cobalt transport system, periplasmic component |  | 7-42 | 72.51 |
| Saliv_gland_allergen_Aed3 |  | 8-25 | 71.19 |
| Peptidoglycan-associated lipoprotein Pal | *Yersinia pestis* | 6-25 | 70.56 |
| Cytochrome c-type biogenesis protein cycj | *Bartonella henselae* | 3-48 | 69.67 |
| Glycine rich protein family |  | 11-32 | 69.14 |
| Urinary protein (RUP)/acrosomal protein SP-10. |  | 3-33 | 68.77 |
| DumPY: shorter than wild-type family member (dpy-5) | *Caenorhabditis elegans* | 7-47 | 68.71 |
| LPS export ABC transporter permease LptG. |  | 5-35 | 67.34 |
| TWiK family of potassium channels family member (twk-11) | *Caenorhabditis elegans* | 4-29 | 66.04 |
| CG13969-PA | *Drosophila melanogaster* | 9-50 | 65.81 |
| MORN repeat protein | *Beggiatoa sp. PS* | 7-31 | 64.41 |
| Signal transduction histidine kinase | *Lactobacillus casei* | 8-42 | 63.91 |
| Secreted protein | *Streptomyces coelicolor* | 12-30 | 63.54 |
| TonB family protein | *Nostoc punctiforme* | 9-36 | 63.16 |
| C53B4.8 | *Caenorhabditis elegans* | 65-185 | 62.28 |
| H/K_exch_ATPase_C |  | 7-36 | 62.23 |
| Lipoprotein required for capsular polysaccharide translocation through the outer membrane | *Escherichia coli* | 7-25 | 61.73 |
| R160.4 | *Caenorhabditis elegans* | 7-42 | 61.58 |
| SVM protein signal sequence |  | 8-29 | 61.27 |
| Nitric oxide reductase subunit C; metal-binding, membrane protein, immune system-oxidoreductas | *Pseudomonas aeruginosa* | 5-32 | 60.51 |
| COLlagen family member (col-102) | *Caenorhabditis elegans* | 7-52 | 60.47 |
| Cytoplasmic membrane protein | *Bartonella henselae* | 7-40 | 60.06 |
| CG3066-PD, isoform D | *Drosophila melanogaster* | 8-33 | 59.22 |
| Synoviolin 1 isoform a | *Homo sapiens* | 7-42 | 59.11 |
| Protein-export membrane protein | *Agrobacterium tumefaciens* | 6-30 | 58.68 |
| CG7875-PA | *Drosophila melanogaster* | 2-44 | 58.12 |
| Synoviolin 1 isoform b | *Homo sapiens* | 7-42 | 57.82 |
| C46H11.8 | *Caenorhabditis elegans* | 12-26 | 57.66 |
| Permease YjgP/YjgQ family protein | *Nostoc punctiforme* | 5-36 | 57.45 |
| RCR |  | 14-32 | 57.43 |
| F55A11.3 | *Caenorhabditis elegans* | 7-36 | 57.42 |
| **BLASTP/PSIBLAST** | | | |
| Herpes virus major outer envelope glycoprotein (BLLF1) | *Herpes virus* | 69-195 | 2.73e-03 |
| small proline-rich protein 3 | *Mus musculus* | 69-195 | 3e-04 |
| ARF GAP-like zinc finger-containing protein | *Trichomonas vaginalis* | 45-194 | 4e-04 |
| aggrecan core protein precursor | *Sus scrofa* | 57-192 | 8e-04 |
| viral protein TPX | *Histoplasma capsulatum* | 64-192 | 0.003 |
| **BLASTP** | | | |
| Proteoglycan **(3)** | *Histoplasma capsulatum* | 59-192 | 0.008-0.048 |
| Small proline-rich protein 3 **(2)** | *Rattus norvegicus* | 68-197 | 0.033 |
| FecR protein | *Cylindrospermum stagnale* | 53-195 | 0.065 |
| Peptidase S8 | *Actinobacillus capsulatus* | 71-193 | 0.10 |
| Aggrecan core protein | *Bos mutus* | 62-192 | 0.56 |
| CD5 antigen-like protein | *Chelonia mydas* | 54-191 | 1.00 |
| **I-TASSER** | | | |
| Chaperone protein PAPD | *Escherichia coli* |  | 1.13 |
| Survival motor neuron protein **(2)** | *Homo sapiens* |  | 1.89, 1.92 |
| Type I hyperactive antifreeze protein **(2)** | *Pseudopleuronectes americanus* |  | 1.77, 1.18 |
| Myc box dependent interacting protein 1 **(2)** | *Homo sapiens* |  | 1.04, 2.07 |
| Major capsid protein | *Synechococcus phage* |  | 1.66 |
| Type I hyperactive antifreeze protein | *Pseudopleuronectes americanus* |  | 0.767 |
| Dynamin family protein | *Nostoc punctiforme* |  | 0.600 |
| Phospholipase C beta | *Meleagris gallopavo* |  | 0.574 |
| LEOA | *Escherichia coli* |  | 0.573 |
| Interferon-induced guanylate-binding protein 1 | *Homo sapiens* |  | 0.571 |
| TcdA1 | *Photorhabdus luminescens* |  | 0.563 |
| Tyrosine-protein kinase Fes/Fps | *Homo sapiens* |  | 0.559 |
| RhUL123 | *Macacine herpesvirus 3* |  | 0.547 |
| 1-phosphatidylinositol 4,5-bisphosphate phosphodiesterase beta-3 | *Homo sapiens* |  | 0.545 |
| **Predict Protein** | |  | |
| Protein binding |  | 37,50,51, 63,124,151-153,169,171 |  |
| Cytoplasm |  |  |  |
| Merozoite surface protein 1 | *Plasmodium reichenowi* |  | 4e-18 |
| Protein piccolo **(5)** | *Rattus norvegicus* |  | 4e-04-0.35 |
| Merzoite surface protein 1 | *Plasmodium reichenowi* |  | 6e-22 |
| Cell surface glycoprotein 1 **(21)** | *Clostridium thermocellum* |  | 0.011-0.85 |
| **Atome2** | | | |
| Protein kinase BYR2 | *Schizosaccharomyces pombe* |  | 51.37 |
| Cell wall surface anchor family protein | *Streptococcus pneumoniae* |  | 46.70 |
| Apocytochrome f | *Chlamydomonas reinhardtii* |  | 33.57 |
| Cytochrome B6 **(3)** | *Mastigocladus laminosus* |  | 23.23-19.48 |
| Bone marrow stromal antigen 2 | *Homo sapiens* |  | 22.50 |
| 30S ribosomal protein S27E | *Archaeoglobus fulgidu* |  | 21.44 |
| SERCA1a | *Oryctolagus cuniculus* |  | 20.68 |
