# Supplementary material for: In silico analyses of mitochondrial ORFans in freshwater mussels (Bivalvia: Unionoida) provide a framework for future studies of their origin and function
Source: BMC Genomics. 2016 Aug 9;17:597. doi: 10.1186/s12864-016-2986-6 (PMC4979158; doi:10.1186/s12864-016-2986-6)

A. M vs M complete mitochondrial genomes

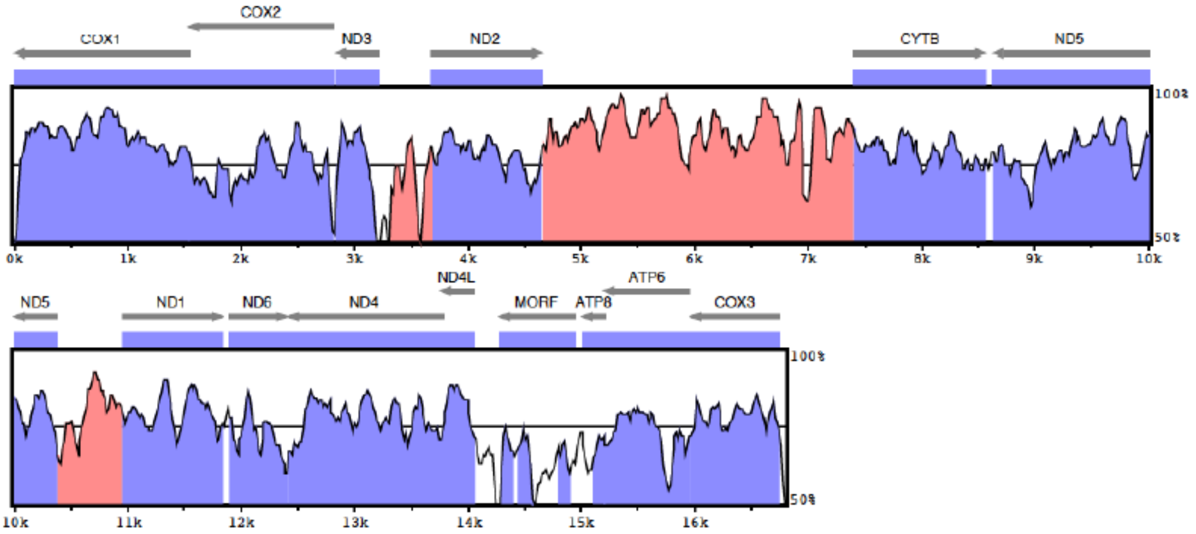

B. F vs F complete mitochondrial genomes

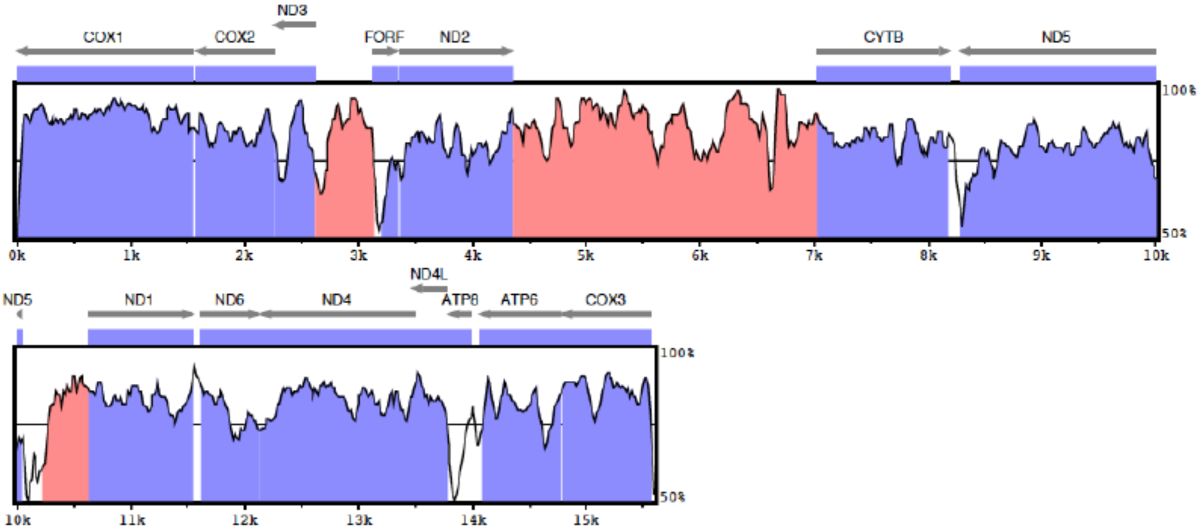

C. F vs H complete mitochondrial genomes

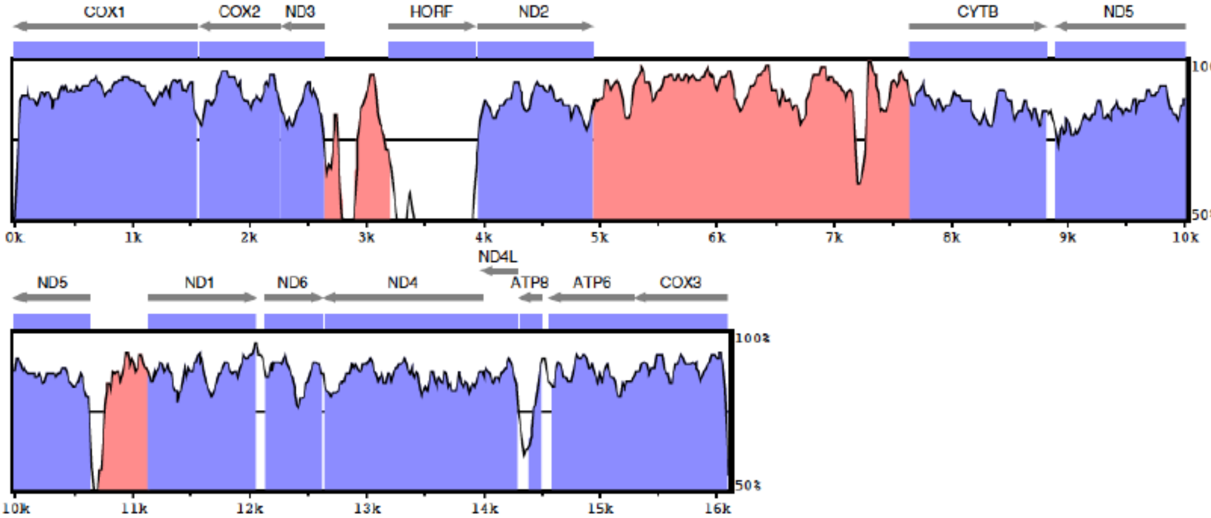

Supplement: Additional file 3: Figure S2. — Percentage of similarity between complete mitochondrial genomes of freshwater mussels with DUI. Each graph shows the percent of conservation between genomes at any given coordinate. The top and bottom percentage bounds are shown to the right of every row. The pink regions are conserved non-protein-coding sequences, the dark blue regions are protein-coding genes, the white regions are non-coding sequences. (A) M vs. M genome comparison between two closely related species (Utterbackia peninsularis and Pyganodon grandis, GenBank accession numbers HM856635 and FJ809754, respectively) showing that the M-ORF gene shows low level of sequence conservation compared to other protein-coding genes. (B) F vs. F genome comparison between two closely related species (U. peninsularis and P. grandis, GenBank accession numbers HM856636 and FJ809755, respectively) showing that the F-ORF gene shows low level of sequence conservation compared to other protein-coding genes. (C) F vs. H genome comparison between two closely related species (Utterbackia peninsularis and U. imbecillis, GenBank accession numbers HM856636 and HM856637, respectively) showing that the F-ORF/H-ORF gene region shows low level of sequence conservation compared to other protein-coding genes. (PDF 160 kb) [file 12864_2016_2986_MOESM3_ESM.pdf]
