# Supplementary material for: In silico analyses of mitochondrial ORFans in freshwater mussels (Bivalvia: Unionoida) provide a framework for future studies of their origin and function
Source: BMC Genomics. 2016 Aug 9;17:597. doi: 10.1186/s12864-016-2986-6 (PMC4979158; doi:10.1186/s12864-016-2986-6)
Supplement: Additional file 4: Figure S3. — Protein sequence alignment of Cumberlandia monodonta M-ORF and ATP8, along with ATP8 from the most diverse members of the Mt_ATP-synt_B superfamily (pfam02326). Homo sapiens ATP8 has also been included for comparison. The alignment was generated using T-COFFEE. The most conserved N-terminal domain, i.e. the best aligned portion, is in red; the rest of the sequences are rather badly aligned (in green). Consensus is shown and indicates good (red), intermediate (yellow), and bad alignment (green), and insertion/deletion (in blue). Cumberland, Cumberlandia monodonta; H_sapiens, Homo sapiens; Malawimonas, Malawimonas sp. (Excavate); Thraustoch, Thraustochytrium sp. (Stramenopiles); Mesostigma, Mesostigma sp. (Streptophyta); Reclinomon, Reclinomonas sp. (Protozoa); Porphyra, Porphyra sp. (Rhodophyta); Cyanidiosc, Cyanidioschyzon sp. (Rhodophyta); Pseudendoc, Pseudendoclonium sp. (Chlorophyta); Acanthamoe, Acanthamoeba sp. (Amoebozoa); Nephroselm, Nephroselmis sp. (Streptophyta). (PDF 236 kb) [file 12864_2016_2986_MOESM4_ESM.pdf]

```
T-COFFEE, Version_11.00.d625267 (2016-01-11 15:25:41 - Revision d625267 - Build 507)
Cedric Notredame
SCORE=696
*
 BAD AVG GOOD
*
Cumberland_atp8    :  58
Cumberland_Morf    :  60
H_sapiens_atp8     :  70
Malawimonasatp8    :  78
Thraustoch_atp8    :  77
Mesostigma_atp8    :  77
Reclinomon_atp8    :  76
Porphyra_atp8      :  72
Cyanidiosc_atp8    :  79
Pseudendoc_atp8    :  78
Acanthamoe_atp8    :  78
Nephroselm_atp8    :  77
cons               :  69

Cumberland_atp8    MPQ-FSP-MSWSVISLLVACHFLVIC-----VV---LWWLG-AG-------------G---YF
Cumberland_Morf    MKATLCKVIEFVLDNGWLCLFYFVLFMACSN-------VLWRVYKVRK-----GLY-K---KV
H_sapiens_atp8     MPO-LNT-TVWPTMITPMLLTLFLITOLKMLNTNYHLPPSPKPMKMKNYN-----------KP
Malawimonasatp8    MPQ-LDN-VTFLSQIFWCFITFSLLYFIVLKNI---LPNIAKVLKIRK-----KLF-D---YY
Thraustoch_atp8    MPQ-LDF-ITFTSQIFWLLIIFFIIYVFFNQSI---VPFLARVLKIRR-----KKS-FCNRKL
Mesostigma_atp8    MPQ-LDW-ITFLPQFFWVAVGFISFYIVCLRFF---LPRLARIFKVRN-----AKAYS---DV
Reclinomon_atp8    MPQ-LDK-VTFFSQYFWLLIFFLTFYFFVLKIV---LPTVVTIFKLRK-----KKL-E---AM
Porphyra_atp8__    MPQ-LDR-VIIFSQIFWLFLMFLVAYISYAHFV---LSNVLKIFLVRWWKLRKDIT-Q---VA
Cyanidiosc_atp8    MPQ-LDR-VIIVTQIFWLLLIMIVAYSFVIKRI---LPSSFRILKIRE-----NFI-K---DL
Pseudendoc_atp8    MPQ-LDT-LTYFTQFVFLLVSFIYIYYFVITYI---IPNTLTARKLRA-----KFN-S---QL
Acanthamoe_atp8    MPQ-LDK-LSFATQYFWLTLFFFGLYFLSVNFF---VILVFKNLKLRN-----IIY-K---IW
Nephroselm_atp8    MPQ-LDQ-VTFFSQFFWLCFFFFTFYAVLLKSF---LPKMNRILRFRK-----KIH-Q---AS

cons               *   :

Cumberland_atp8    --V---------------------SFFGEG--EVMLPLR-E-----FGFNVC-----------
Cumberland_Morf    -KRVI-------------------GVIWKP----------TAVS-------------------
H_sapiens_atp8     WEPKWTK-IC--S-LHSL---------------------------------------------
Malawimonasatp8    -N----------------------SLFNNIN--TI--NN-N-----Y---IVFKS-SDISNLF
Thraustoch_atp8    -TT---------------------AFFNENK--VL--FS-E-----YDSALVKSS-RSLNNLF
Mesostigma_atp8    -SSK--------------------RTKNKL--EPL---H-S-----FDSLLCNSL-EVSTGWI
Reclinomon_atp8    -ASEVE------------------QLKKEQ--SSI--LS-N-----YDSVLIDSF-SSSREIF
Porphyra_atp8__    LKSRLTK-LLIDSNSHSLREIYSAA---KSILLSLT--KKL-----FDSNLNNPKI--SLQDL
Cyanidiosc_atp8    -IL---------------------N-V---E--KL--NK-E-----ONTOL-KNT-IKLNOHY
Pseudendoc_atp8    -ESSKGL---LQV-PSEL---GGEAFIGELALESLT--SEA-----FTQLACKSAASTTVEDL
Acanthamoe_atp8    -YFFLYRFDYV-------------DYNNKH--KSL--VN-TSFSFIYYTLYINFFILTKINFI
Nephroselm_atp8    -SD---------------------SYVGEY--QSL--LQ-N-----YHTARIPEI-RTSTEHL

cons

Cumberland_atp8    ---------------------------------------------------------------
Cumberland_Morf    VKI--KKD------------K-------------VEKPKV--MEK---AK-------------
H_sapiens_atp8     -------------------------------------------P---------------PQS-
Malawimonasatp8    FII--NKNINDYNEIYK---NINNNSYISNNIRN-----------------------------
Thraustoch_atp8    VNH--NSSLQSWVSINKGLFFINN-NFLDTKFLS-----------------------------
Mesostigma_atp8    QES--REEASNWEKSN-YQWR-------------TNKK-S--LDQIKSSFQMLA--ENSHRR-
Reclinomon_atp8    NST--VSKSNKWIDES-AR-S-------------LNKTSL--LD-VNK--KYLNAINDINKE-
Porphyra_atp8__    NSLVLK-----VSLET-SL-----------------YSSK--SI---------------TKS-
Cyanidiosc_atp8    ------------INLIKNIILVQN-KFYDEILSNYYKIFI--KK-IN------------LKE-
Pseudendoc_atp8    NA--TN-KTGKHNIQS-AL-M-------------LHYTYA--LA---------------NKK-
Acanthamoe_atp8    FEK--TKS------------I-------------INKTQT--LNQ---SQRLLM--ISLLNN-
Nephroselm_atp8    SKV--LGNTRNWVEST-LL-Q-------------LNQTNEQNKQ-MNR--MYVRSIGEMTISQ

cons

Cumberland_atp8    ---------------------------------------------------------------
Cumberland_Morf    ------------------------------KGKKAAVSGK---VGKKSG-------------G
H_sapiens_atp8     ---------------------------------------------------------------
Malawimonasatp8    -------------------------------YS-----------LLNITRKLSKVITNGK---
Thraustoch_atp8    -------------------------------YL-----------ILIQSRF---FMVS-R---
Mesostigma_atp8    --------------------FLFSEDLNFLKGSKSPVTEK---IELGKIKYLRRSFT-----L
Reclinomon_atp8    -------------------------------YL-----------AFNKIK---SL---I----
Porphyra_atp8__    ---------------------------GIYSHW-----------T------------------
Cyanidiosc_atp8    ---------------------------TFFTYA-----------SFMQLYK---NLIQRYLIN
Pseudendoc_atp8    ---------------------------GLLNYE-----------L--------TA---A----
Acanthamoe_atp8    --------------------F--------LNSNSVDFVKKFQELNIDEI--------------
Nephroselm_atp8    TMMTTGFDLVFPPIQASQNTGVAKQ--KLYAQR-----------ILNRLK---KKATSR----

cons
```
