# Supplementary material for: In silico analyses of mitochondrial ORFans in freshwater mussels (Bivalvia: Unionoida) provide a framework for future studies of their origin and function
Source: BMC Genomics. 2016 Aug 9;17:597. doi: 10.1186/s12864-016-2986-6 (PMC4979158; doi:10.1186/s12864-016-2986-6)
Supplement: Additional file 5: Figure S4. — Protein sequence alignment of Cumberlandia monodonta F-ORF and NAD2. The alignment was generated using T-COFFEE. Consensus is shown and indicates identical (*) and similar (: and.) amino acids. Description of the data: Protein sequence alignment of Cumberlandia monodonta F-ORF and NAD2. (PDF 141 kb) [file 12864_2016_2986_MOESM5_ESM.pdf]

```
T-COFFEE, Version_11.00.d625267 (2016-01-11 15:25:41 - Revision d625267 - Build 507)
Cedric Notredame
SCORE=536
*
 BAD AVG GOOD
*
Cumberland_Forf     :  75
Cumberland_nad2     :  41
cons                :  53

Cumberland_Forf     MAIMT--LIILIPLSYLPLIWSNTDNLKTANN-------------------------------
Cumberland_nad2     MKSPHKLLFVLLMTTSTATVLSSSNWLVTWIGLEINMIGFIPLMYYKTTANESETAVKYLIPQ

cons                *      *::*:  :  . : *.:: * *  .


Cumberland_Forf     -------------------L-KMKPIAHDLKPSKHPTSN------------------------
Cumberland_nad2     SLGSTMFITSALISHHSDNIQSLMPIAMCLKLGAAPFHFWFPAVMSGLSLTPAFILLTWQKIA

cons                                   : .: ***  ** .  *


Cumberland_Forf     -------------------------------------------------------IT------
Cumberland_nad2     PIFAISSMNPTLITKILPVAGISALWGGIGGLNQTDVRLLLSYSSIAHTGWMLASISAPSEVL

cons                                                                       *:


Cumberland_Forf     ----------------KPQPNDTQTSNEHSPNTYKPKKSKA----------------------
Cumberland_nad2     TLYLMTYVMINTSLFIALLQNSVKLHKQLFPSSQKPYESFLLAITILSLGGLPPLTGFIMKLL

cons                                    *..:  ::  *.: ** :*


Cumberland_Forf     -------------------------------STNLTNDKPNATKEP-----------------
Cumberland_nad2     VLMFSEAKMIIMMTLILGAIMSLYYYLTLTFSPLLSLHKMPISQLKLITSASTSFLLSQILPL

cons                                               *. *: .*   ::


Cumberland_Forf     -------
Cumberland_nad2     SLLLFFF

cons
```
