# Supplementary figures and images for: In silico analyses of mitochondrial ORFans in freshwater mussels (Bivalvia: Unionoida) provide a framework for future studies of their origin and function
Source: BMC Genomics. 2016 Aug 9;17:597. doi: 10.1186/s12864-016-2986-6 (PMC4979158; doi:10.1186/s12864-016-2986-6)

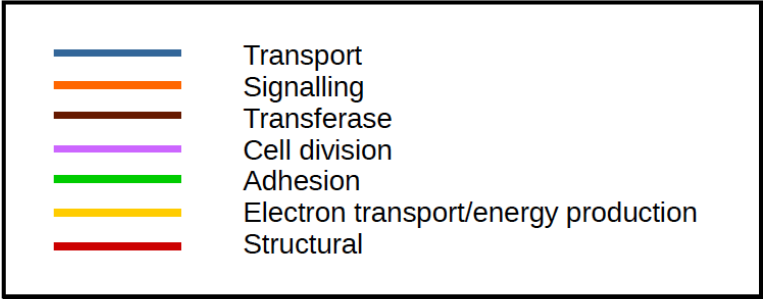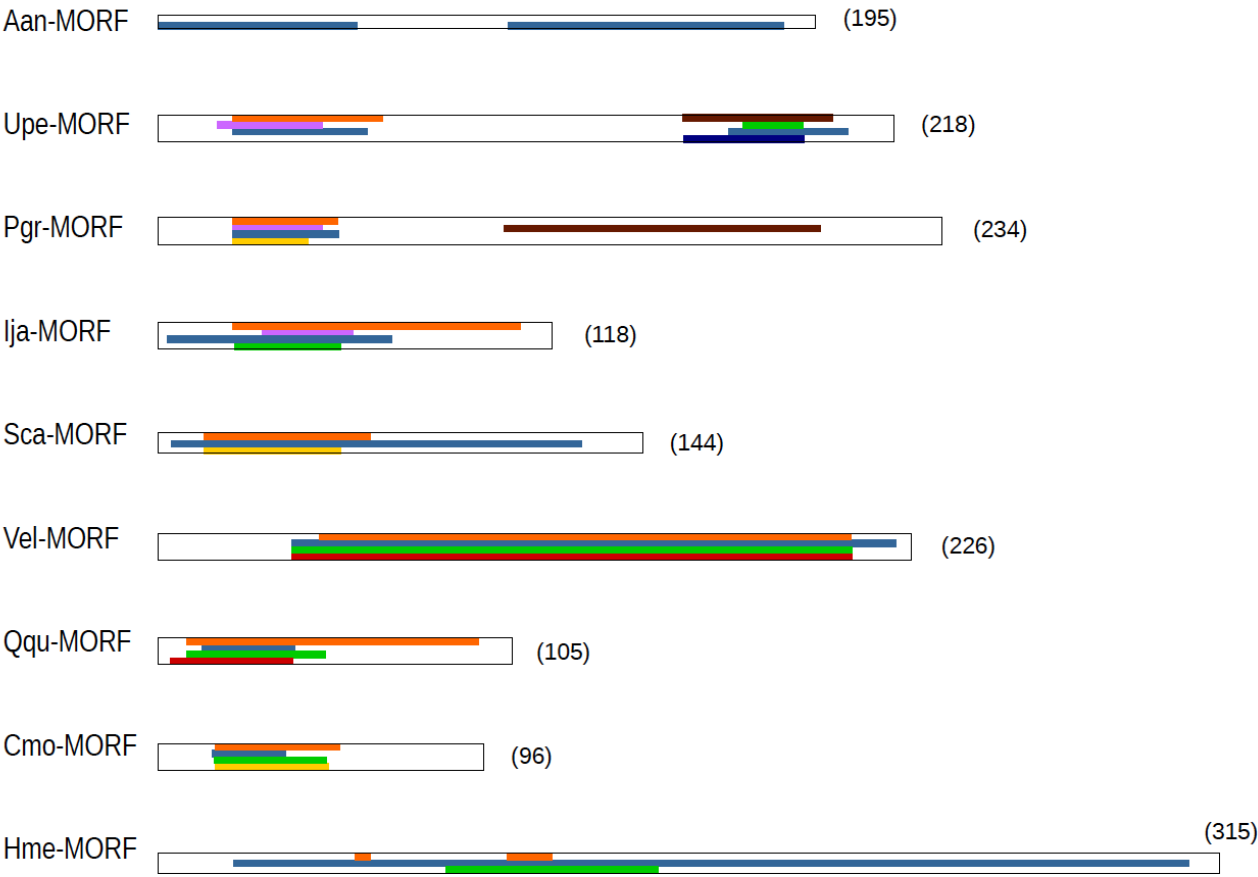

A

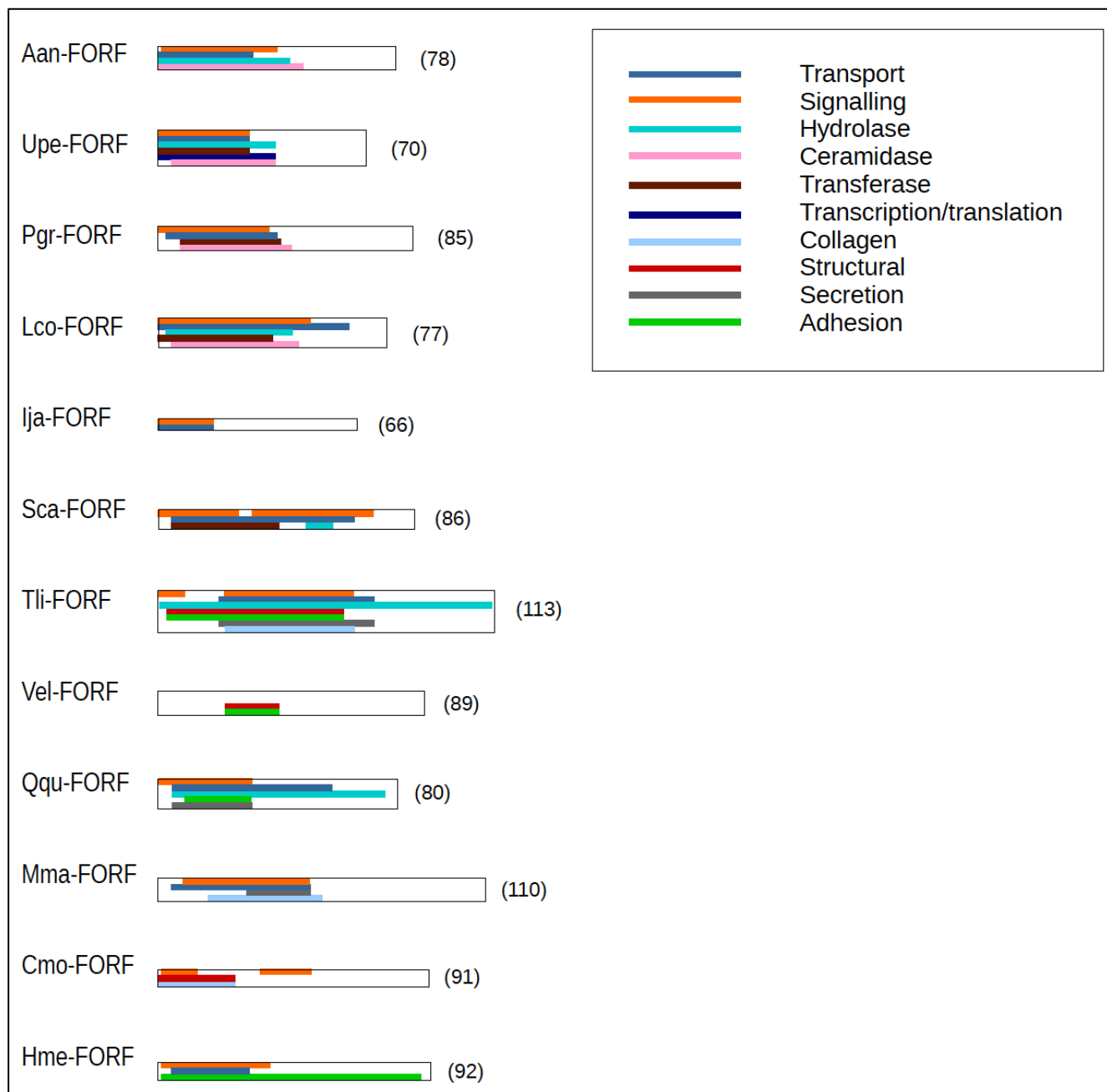

B

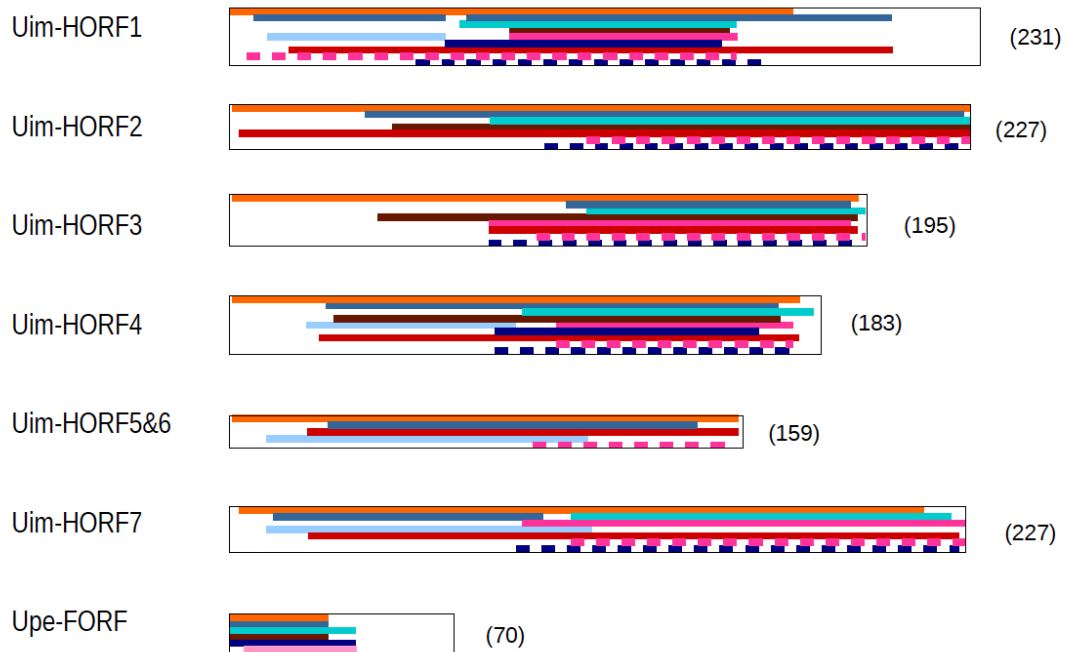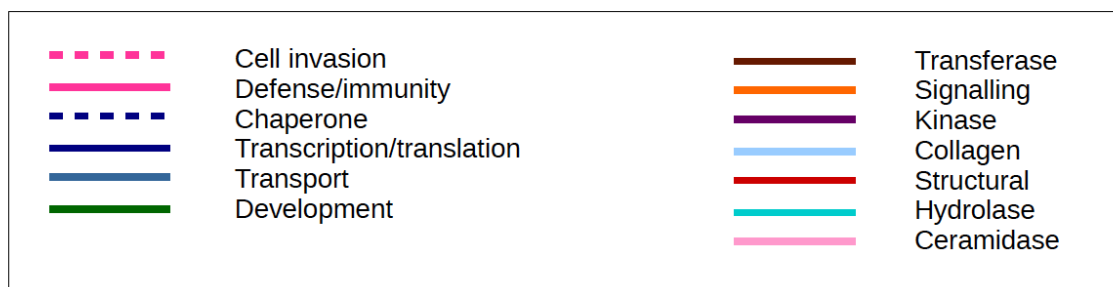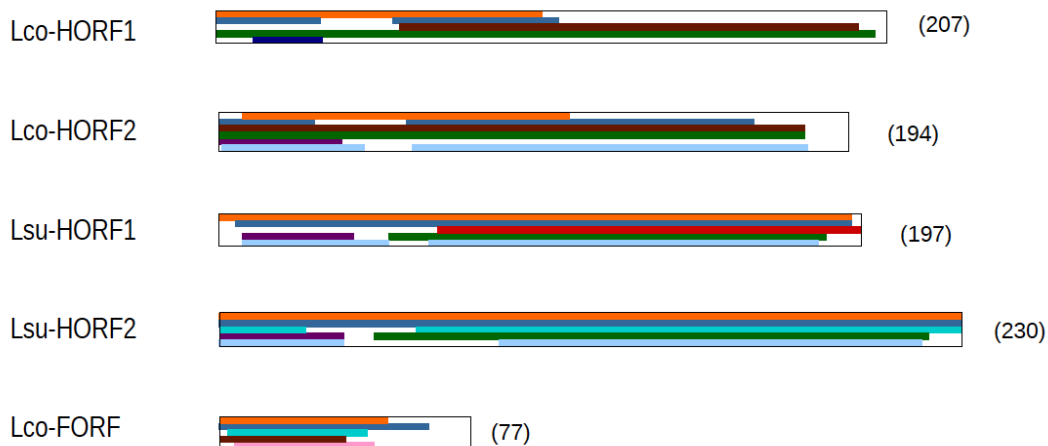

C

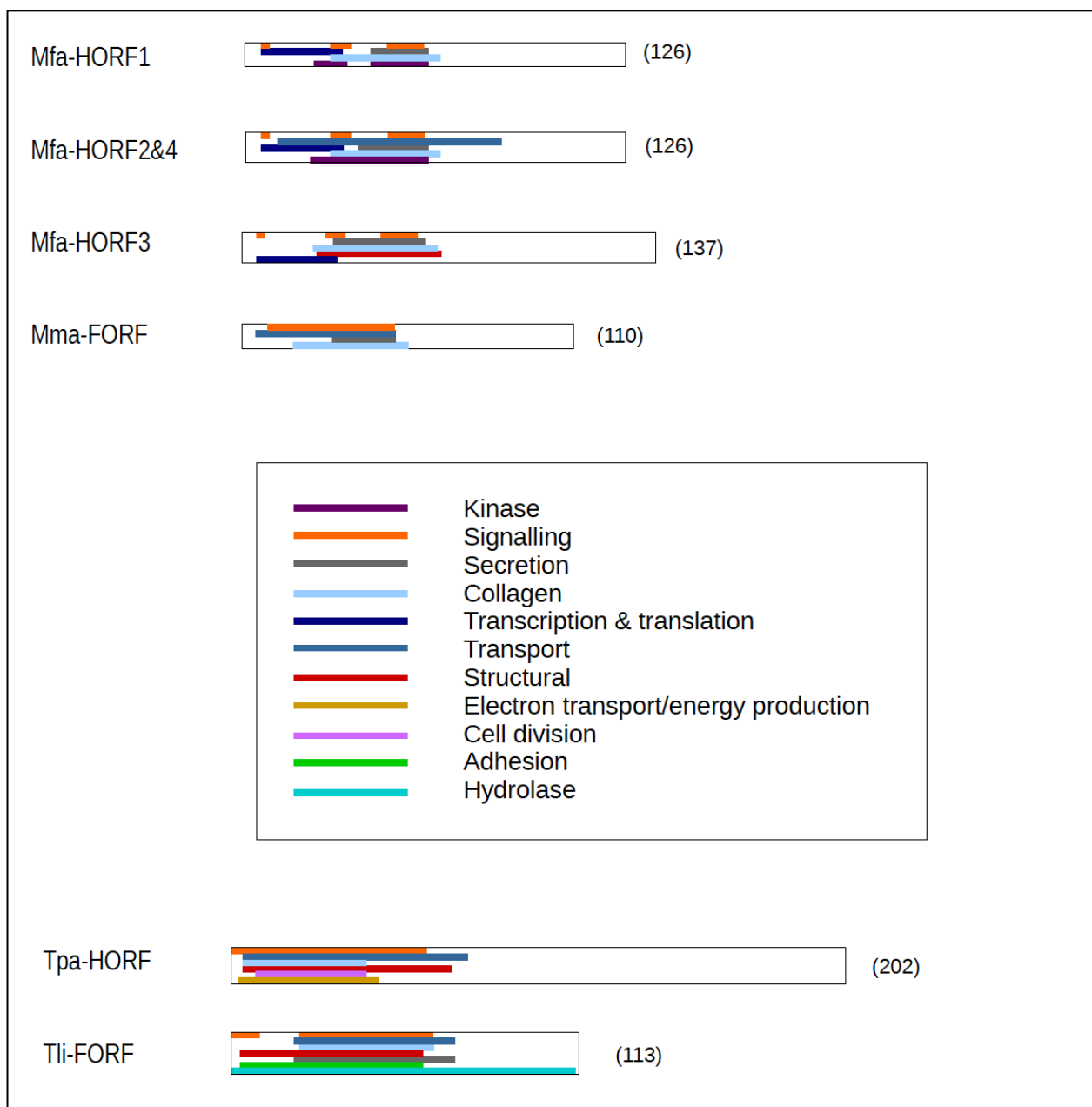

D

Supplement: Additional file 6: Figure S5. — Position of frequently recurring functions in HHpred and BLAST hits for (a) M-ORFs, (b) F-ORFs, and (c) and (d) H-ORFs. Hits with positions were grouped into categories and traced together, showing hot spots of functionality. Protein length in amino acids is indicated in parentheses. (PDF 301 kb) [file 12864_2016_2986_MOESM6_ESM.pdf]
